# The effects of *Thymus capitatus* essential oil topical application on milk quality: a systems biology approach

**DOI:** 10.1038/s41598-025-88168-0

**Published:** 2025-02-07

**Authors:** Ralph Nehme, Chiara Gini, Elise Vanbergue, Sergine Even, Filippo Biscarini, Sonia Andrés, Lucie Rault, Faustine Noel, Valerie Hardit, Said Bouhallab, David M. Pereira, Riadh Ksouri, Philippe Roussel, Secundino López, Paola Cremonesi, Bianca Castiglioni, Donatella Caruso, Fiorenza Faré, Manuela Fontana, Fabrizio Ceciliani, Latifa Abdennebi-Najar

**Affiliations:** 1 Quality and Health Department, IDELE Institute, 149 Rue de Bercy, 75595 Paris CEDEX 12, France; 2 INRAE, Institut Agro, STLO, 35042 Rennes, France; 3 Department of Veterinary Medicine and Animal Sciences, Università degli Studi di Milano, Via dell’università 6, Lodi, Italy; 4 Department of Bioinformatics, Biostatistics, Genomics, Institute of Agricultural Biology and Biotechnology, IBBA-CNR, Milan, Italy; 5 Instituto de Ganadería de Montaña (CSIC-Universidad de León, Finca Marzanas s/n, 24346 Grulleros, Spain; 6 REQUIMTE/LAQV Laboratory of Pharmacognosy, Department of Chemistry Faculty of Pharmacy, University of Porto, R Jorge Viterbo Ferreir 228, 4050-313 Porto, Portugal; 7 Laboratory of Aromatic and Medicinal Plants, Biotechnology Center of Borj-Cédria, BP 901, 2050 Hammam-Lif, Tunisia; 8 Departamento de Producción Animal, Universidad de León, 24007 León, Spain; 9 Department of Pharmacological and Biomolecular Sciences, Università degli Studi di Milano, 20133 Milan, Italy; 10 UNITECH OMICs Platform, Università degli Studi di Milano, 20133 Milano, Italy; 11 Centre de Recherche Saint-Antoine (CRSA), Sorbonne University, INSERM UMR_S_938, 75571 Paris Cedex 12, France

**Keywords:** Milk quality, Essential oil, *Thymus capitatus*, Microbiota, Food safety, Biological techniques, Microbiology, Systems biology

## Abstract

Essential oils (EO) are known for their antibacterial and anti-inflammatory properties and can be used as an alternative to reduce the reliance on antimicrobials in dairy cattle. While many studies have explored the beneficial properties of EO in vitro, their effects on milk quality and milk microbiota, when applied directly to the udder skin, remain relatively unknown. This study aimed to investigate the impact of *Thymus capitatus* essential oil (TCEO), known for its high antibacterial and antioxidant properties, on milk microbiota using 16S rRNA sequencing, the lipidomic profile via liquid chromatography-quadrupole time-of-flight mass spectrometry, udder skin microbiota, and inflammatory biomarkers of dairy cows at the end of lactation. Sixteen-quarters of 12 Holstein cows were selected, and TCEO was topically applied to the udder skin twice a day for 7 days. Milk was collected aseptically on days 0, 7, 21, and 28 before morning farm milking. The results showed no significant changes in microbiota composition after the EO treatment in alpha and beta diversity or taxonomical composition at the phylum and genus levels. TCEO induced limited changes in the milk lipidome, primarily affecting diacylglycerols at T21. The treatment did not affect inflammatory biomarkers, milk sensory properties, or quality. Our study is the first to demonstrate that a local application of 10% TCEO on cow’s quarters does not significantly alter milk quality or microbiota composition in milk and skin. More studies should be conducted to ensure the safe use of TCEO in dairy cows and explore its potential benefits on antibiotic-resistant bacteria as an alternative or support for antibiotic therapy.

**Supplementary Information:**

The online version contains supplementary material available at 10.1038/s41598-025-88168-0.

## Background

Antimicrobial resistance (AMR) is a significant threat to humans and animals. In 2019 alone, AMR was responsible for 4.95 million deaths. By 2050, it is expected to prematurely kill 10 million people annually (nearly matching the rate of cancer deaths in 2020), resulting in an economic output cost of 3.4 trillion USD^1^. Although often unavoidable, antibiotic-based therapy is not always efficient, and the excessive use of antibiotics leads to the development of AMR within the bacterial community^2^. Therefore, alternative strategies and therapeutic approaches are required to reduce the use of antibiotics in humans and animals. Herbal medicine, including crude herbs, herbal compounds, herbal crude extracts, and herbal effective secondary metabolites such as essential oils (EO), are promising alternatives to antibiotics in humans^3^ and animal husbandry^4^. In recent decades, EOs, obtained through several plant extraction processes, have become a preferred therapy for replacing antibiotics and chemicals because of their excellent therapeutic effectiveness, low toxicity, minor adverse effects, and less possibility of developing drug resistance^4,5^.

Ethnoveterinary use of medicinal plants is already ongoing: for instance, 75% of small rural farms in the Eastern Cape Province of South Africa already use plants or herbal remedies to treat livestock^6^. This trend is expanding in developing countries, such as Ethiopia and Nepal, as well as in developed countries, like Spain and Switzerland, among others^7^. The demand for these natural products is also increasing, especially in organic farms^8^, which currently occupy 1.6% of the world’s agricultural lands, corresponding to 76.4 million hectares and a global market that reached 125 billion euros in 2021^9^.

Several EOs, like *Angelica sinensis, Gaultheria procumbens, Glycyrrhiza uralensis,* and *Thymus vulgaris*, which are commercialized as intramammary infusions (PhytoMast^®^^10^) or for external use (Master Mint^®^) have been already included in animal husbandry routines^11^. Other EOs, such as those extracted from *Lavandula angustifolia* and *Origanum vulgare,* were studied for their effects via intramammary infusion and/or external application at the quarter and demonstrated antibacterial activities against *Staphylococcus* spp, and *Streptococcus* spp^12,13^. The EOs from *Origanum vulgare* and *Satureja montana* were investigated for their effects on milk, by determining their antibacterial and antioxidant activity, as well *as Fumaria indica*, *Nepata cataria,* and *Adiantum capillus*, with promising results^14,15^.

To the best of the authors’ knowledge, the EO’s effects on milk microbiological content and quality are limitedly known. This gap should be filled as lawmakers’ organizations advocate responses to develop adequate regulations. The impact of EO at the system’s biology level, including the microbiota and the metabolome/lipidome, remains equally undisclosed^16^.

*Thymus capitatus* is a Mediterranean endemic, perennial shrub and ornamental plant that belongs to the *Lamiaceae* family^17^. Also known as *Satureja capitata L., Coridothymus capitatus (L.) Rchb. f., Thymus capitatus (L.) Hoffmanns. & Link, Thymus marinosci Ten*., Spanish oregano or *Thymbra capitata* (L.), *Thymus capitatus* has been investigated for its essential oil’s antioxidant, antimicrobial, and antiviral properties^18–26^ and its anti-inflammatory effects. It has been reported that *Thymus capitatus EO* (TCEO) can inhibit the expression of inflammatory cytokines, iNOS, and COX-2, as well as suppress the production of neutrophil elastase and the synthesis of PGE2^27–29^. A previous study has already demonstrated its in vitro anti-bacterial properties^24^. More recently, evidence of in vitro immunomodulatory activity on THP-1 Cells has also been provided^30^. To our knowledge, no data specifically addressing the effects of TCEO on dairy cows currently exist. Given the widespread use of essential oils in the animal sector, it is urgent to conduct further studies to assess their safety at a systems biology level.

Our study aimed to determine the impact of TCEO on the milk and udder skin of cows with subclinical mastitis. We characterized the skin microbiome and analyzed milk production, quality, and lipidome. Additionally, we evaluated the effects of subclinical mastitis on milk sensory properties and measured critical inflammatory markers. For that purpose, mammary glands were topically treated with TCEO at the end of the lactation period. This stage is crucial for udder health as it undergoes involution at the beginning of the dry period, along with tissue remodeling and repair. Ensuring optimal mammary gland health at this stage is pivotal for successful lactation and achieving high milk yields in subsequent periods. It is important to note that, at this stage, antibiotic treatment is primarily used on cows.

## Methods

### Experimental design

#### Evaluation of the TCEO

The antibacterial activity of TCEO^31^ was compared in vitro with that of nine essential oils from *Laurus nobilis, Nigella sativa, Origanum majorana, Salvia officinalis, Rosmarinus officinalis, Pelargonium graveolens, Coriandrum sativum, Artemisia herba alba*, and *Juniperus oxycedrus* against *Escherichia coli* (*E. coli*) and *Staphylococcus aureus* (*S. aureus*) previously isolated from an infected cow udder. The antibacterial activity test was performed according to Vuddhakul et al. (2007) with non-relevant modifications^31^. The protocol was repeated 5 times with disks impregnated with Colistin, Amoxycillin, Ampicillin, and Cephalexin. The statistically significant differences between EO were carried out by one-way analysis of variance at a 95% confidence level (*p* ≤ 0.05). Furthermore, Tukey’s honest significance test was used.

#### Animal selection and collection of milk samples

Twelve Holstein cows were selected in the INRAE experimental dairy farm of Méjusseaume (Le Rheu, Brittany, France). The animals were evaluated as clinically healthy at the veterinary visit. They presented no clinical signs of infection nor clinical mastitis and received no antibiotic treatment for at least 6 months before enrolment. The recruited animals were at the end of the lactation period. The animals were divided into two groups (Control and Treated), homogenous per cows’ numbers and parity. Cows had permanent access to pasture, and their diet was composed of 65% corn silage, 12.5% soybean 48 and 12.5% energy concentrate (made by a mix of cereals, predominantly corn), and 10% alfalfa ad libitum (proportions are reported in % of dry matter). Milk was collected twice a day in a rotary milking parlor. Supplementary Table S1 presents the samples included in the experimental design, reporting animal parity, average somatic cell count (SCC), and microbiological results of the milk samples at the beginning of the study*.*

#### Application of TCEO

A 10% TCEO was applied by skin massage to enhance the penetration of active compounds^32^. The mixture was prepared by dissolving 0.5 g of TCEO in 4.5 g of milking grease and stored in single-dose containers at 4 °C. This concentration was selected after testing various percentages (2.5%, 5%, 7.5%, and 10%) on cow udders, with no observed local or systemic reactions (data not shown). It was applied to the udders of the Treated group (6 cows, 8 quarters) twice daily after each milking for one week. The Control group (6 cows, 8 quarters) received only milking grease massages.

#### Milk samples collection

Milk samples were aseptically collected on days 0, 7, 21, and 28 (T0, T7, T21, T28) before morning milking. After discarding the first three foremilk streams, teats were disinfected with 70% alcohol^33^. Part of the milk was immediately analyzed for somatic cell count (SCC), bacteriology, fat and protein content, casein micelles, and sensory properties. Viscosity, IL-8, acute phase proteins (haptoglobin and lactoferrin), microbiota, and lipidome analyses were performed on milk samples stored at − 80 °C.

#### Skin sample collection for microbiota determination

The selected quarters were wiped with a wet tissue and then dried. Before milking and using a skin swab (R. Langenbrinck GmbH, Emmendingen, Germany), an area of 1 cm^2^ of the quarter was rubbed by slightly rotating the swab through an angle of 45° for 10 s. The skin microbiota was collected twice on T0, before TCEO application (T0A), after TCEO application (T0B), and once on T7.

### Part 1: Microbiota analysis

#### Bacteriological analysis

Bacteriological cultures were performed according to the National Mastitis Council guidelines (https://www.nmconline.org/nmc-protocols-guidelines-and-procedures/). A composite sample of 10 ml of milk from the Treated udders was collected in a labeled aseptic container at T0, 7, 21, and 28. Cultures were incubated for 24 h at 37 °C under aerobic conditions on blood agar (Columbia Agar containing 5% defibrinated sheep blood) for 48 h at 37 °C under aerobic conditions. Gram stain, coagulase, catalase, and oxidase assays were conducted on positive cultures. *Staphylococcus* spp. Covidiase detection was performed using rabbit plasma^34^.

#### DNA extraction and 16S rRNA-gene sequencing of milk and skin microbiota.

DNA from milk samples was extracted using the Cremonesi et al.^35^ protocol, while DNA from skin swabs was extracted with the QIAamp PowerFecal Pro DNA Kit, with slight modifications. Skin swabs were processed in a PowerBead Pro Tube with CD1 solution and vortexed on a TissueLyser II instrument. Blank controls were included in both cases. DNA quality and quantity were measured with a NanoDrop ND-1000 spectrophotometer (NanoDrop Technologies, Wilmington, DE, USA). Sequencing of both milk and skin samples targeted the V3-V4 regions of the 16S rRNA gene using specific primers (forward: 5'-CCTACGGGNGGCWGCAG-3', reverse: 5'-GACTACHVGGGTATCTAATCC-3')^36^. The 16S rRNA gene raw sequences obtained were deposited in UNIMI Dataverse (https://doi.org/10.13130/RD_UNIMI/GMPFOB, https://doi.org/10.13130/RD_UNIMI/OPMKOC).

#### Bioinformatic processing and statistical analysis of milk and skin microbiota

After demultiplexing, paired-end reads from 16S rRNA-gene sequencing were quality-checked using the software package MultiQC^37^. Subsequently, analyses were performed using QIIME 1.9^38^, retrieved as a container used through Singularity^39^ from the Docker hub (https://hub.docker.com/r/fischuu/qiime-1.9.1). The following steps and parameters were previously described by Biscarini et al.^40^.

#### Alpha- and beta-diversity of milk and skin swab samples

The milk and skin microbial diversity was assessed within- (alpha diversity) and across- (beta diversity) samples with the adaptations required by the dataset as previously described in Ranilla et al.^41^. Briefly, filtered and normalized OTU tables (at the OTU level) were used to estimate all alpha (using Chao1, ACE, Shannon, Simpson and InvSimpson, Fisher, and Shannon) and beta diversity (Bray–Curtis dissimilarities) indices. OTU counts, and diversity indices were corrected for a baseline set on the first time point (T0) before statistical analysis, considering that samples at T0 cannot be affected by TCEO as no treatment was applied to quarters assigned to the Treated or Control groups.

#### Statistical models for milk and skin microbiota analysis

Differences between groups (Treated vs. Control) along time points in terms of OTU abundances and diversity indices were evaluated with the three following linear models:

1 $${\text{y}}_{{{\text{ik}}}} = \mu + {\text{ time point}}_{{\text{k}}} + {\text{ e}}_{{{\text{ik}}}} ,$$

2 $${\text{y}}_{{{\text{ij}}}} = \mu + {\text{ treatment}}_{{\text{j}}} + {\text{ e}}_{{{\text{ij}}}} ,$$

3 $${\text{y}}_{{{\text{ikj}}}} = \mu + {\text{ time point}}_{{\text{k}}} + {\text{ treatment}}_{{\text{j}}} + {\text{ e}}_{{{\text{ikj}}}}$$

where y_ikj_ is the abundance (counts) or index value for each taxonomy (OTU) and alpha and beta diversity metric per quarter i in time point k and treatment j; time point_k_ is the effect of the categorical variable time point (4 classes); treatment is the effect of the categorical variable treatment (2 classes); e_kj_ are the residuals of the model. Model (1) focuses on the influence of time on the milk and skin microbiota, while model (2) focuses on the effect of treatments. Model (3) considers both time and treatment effects, allowing for an adjusted assessment of OTU counts and alpha diversity index values in the milk and skin microbiota.

### Part 2: Milk quality

#### Fat and protein content

Composite (four glands) milk samples (50 ml) from the whole milk were collected twice a week for 2 weeks prior and 5 weeks after the first application of T0 during the morning milking to determine the proteins and fats content using MilkoscanTM (Foss electric, Hillerød, Denmark).

#### Hydrodynamic diameters of casein micelles

Particle size analysis of casein micelles was performed using Zetasizer Nano ZS (Malvern Panalytical, Malvern, UK). Six fresh milk samples were chosen randomly at T21 (3 from Treated quarters and 3 from the Control group). The samples were first skimmed by centrifugation at 4 °C and filtered (0.8 µm pore size), then diluted 1/100 (v/v) in protein-free ultrafiltration permeate of the same milk. The refractive index used was 1.57, and the absorption index was 0.001^42^. Every measurement was repeated 6 times.

#### Milk viscosity

The viscosity measurements were carried out on skimmed (by centrifugation at 4 °C) thawed milk at T0, 7, 21, and 28 from 4 randomly chosen samples (2 from the Control group and 2 from the Treated group) according to the method of Doudiès et al.^42^. Flow measurements were performed using a Low Shear 400 viscometer (Lamy Rheology, Champagne au Mont d’Or, France) using a Couette geometry (inner and outer radii = 5.5 and 6.0 mm respectively, shear rate = 2–120 s − 1) at 20 °C.

#### Sensory analysis

A panel of experts conducted a sensory evaluation of milk to assess its organoleptic qualities, following NF ISO 8586-2 and NF ISO 22,935 standards. The evaluation was performed on both fresh and pasteurized milk collected on T7. Pasteurization involved heating the milk to 100 °C for 5 min, and it was served at 40–50 °C. Visual and olfactory characteristics were evaluated for fresh and pasteurized milk, while taste was assessed only for the pasteurized milk. Panelists rated the intensity of odors and aromas on a scale from 0 to 10, with 0 indicating no perception of TCEO and 10 indicating a very strong perception according to the process described by Listrat et al.^43^.

### Part 3: Lipidomic analysis

#### Preparation of milk samples for lipidomic analysis: lipid extraction

Two aliquots (100 µL) from each sample were added with internal standards (Splash™ Lipidomix^®^ Internal Standards Avanti^®^ Polar and a mix of 13C-Palmitic and 13C-Linoleic Acids at 50 µg/mL), extracted and separated as previously described^44^. Electrospray ionization and mass spectrometer conditions were set as presented in Supplementary Table S2.

#### Lipid data processing and statistical analysis

Data are expressed as the ratio of analyte to an internal standard area (1-phenoxy-2-propanol), and fatty acids are expressed as ng/mL of milk. Data processing was done using the untargeted data processing program MSDIAL (v. 4.24) with LipidBlast database (v. 68), as previously described by Ceciliani et al.^45^. This database contains 81 lipid classes, 377,313 molecules, and 554,041 spectra in positive polarity, and 94 lipid classes, 356,477 molecules, and 792,757 spectra in negative polarity. Statistical analysis was carried out using the MetabolAnalyst 5.0 Webtool, as previously described^45^. The analysis did not include variables containing more than 20% of missing values (i.e., values less than LOD)^46^. The Principal Component Analysis (PCA), the Partial Least-Squares Discriminant Analysis (PLS-DA), and the volcano plots and heatmaps were generated using the MetaboAnalyst 5.0 web tool.

### Part 4: milk inflammatory parameters

#### Analysis of acute phase proteins (APP) and interleukin 8 in milk

Haptoglobin (Hp) and Lactoferrin concentration were measured using an in-house developed ELISA as previously described^47^. The inflammatory cytokine Interleukin 8 (IL-8) dosage in milk was carried out according to Roussel et al.^48^. Statistical analyses were performed using GraphPad Prism version 9.0.0 for Windows (GraphPad Software, San Diego, California USA, www.graphpad.com).

#### Somatic cells count

Composite (four glands) milk samples (50 ml) from the whole milk were collected twice a week for 5 weeks prior and 5 weeks after the first application of T0 during the morning milking to perform the SCC using the flow cytometer Fossomatic (Foss electric, Hillerød, Denmark).

#### Statistical analysis

All data were expressed as mean ± SEM. Values of *p* < 0.05 were considered statistically significant. For the sensory analysis test, the significance levels were determined using the Student’s *t*-test. For the other milk quality parameters (Fat and protein content, hydrodynamic diameters of casein micelles, and milk viscosity) one way ANOVA was used.

For the somatic cell count, the levels of significance were determined using the Type III ANOVA. For the IL-8 measurements, one-way ANOVA was used. For the other milk inflammatory parameters (APP and LF) two way ANOVA was used.

## Results

The first part of the study looked at the microbial communities (routine bacteriological analysis) in the skin and milk after TCEO treatment over a set period. The second part focused on whether the topical application of TCEO affected milk quality (composition, protein and fat content, hydrodynamic sizes of casein micelles, viscosity, and sensory attributes). Finally, we presented related inflammatory parameters, SCC, IL-8, and APP content.

### Evaluation of TCEO

**Fig. 1:**
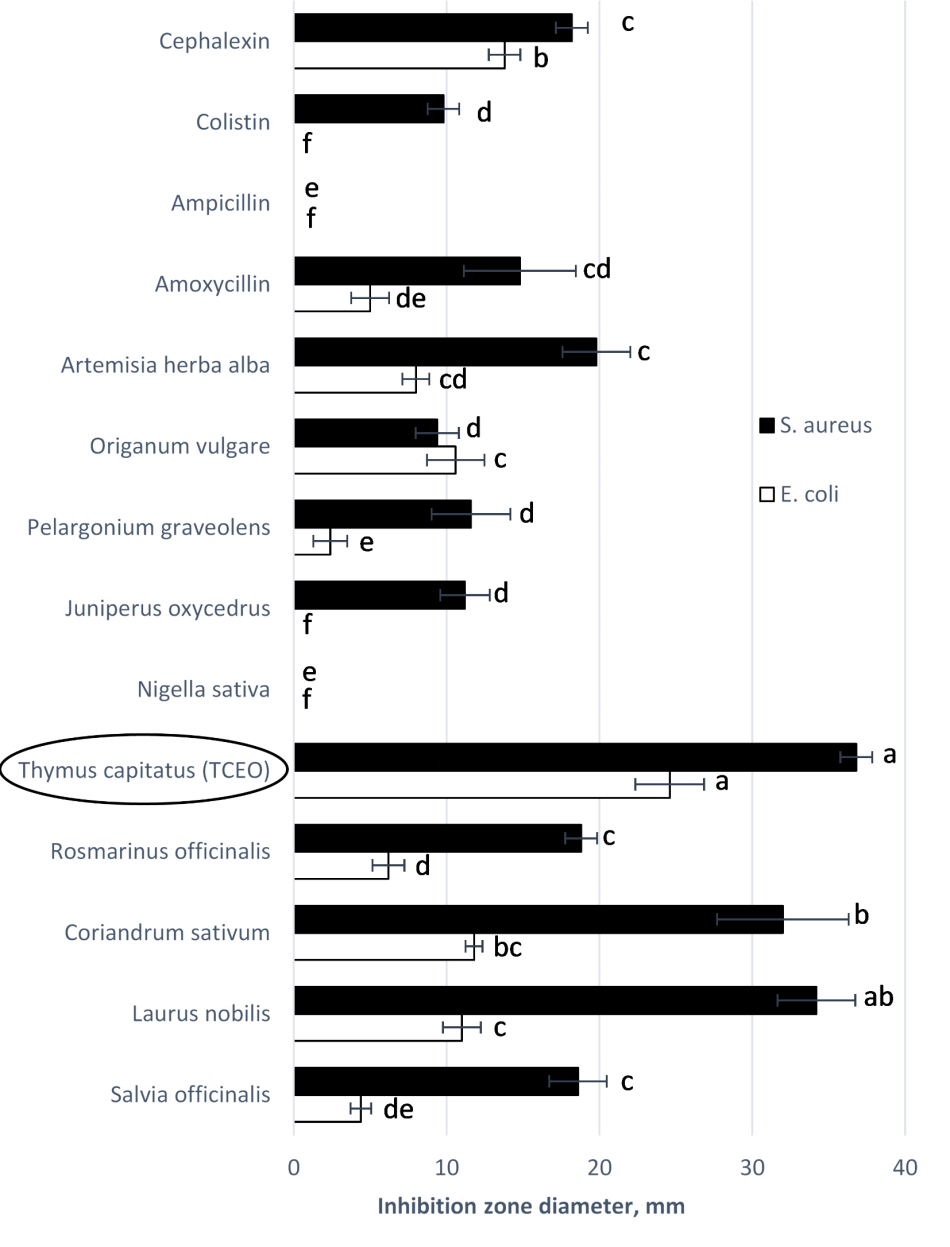
Inhibition zone diameter of the 9 EO and 4 antibiotics (Amoxycillin, ampicillin, colistin, and cephalexin) against *E.coli* and *S. aureus*. Values are the average diameter of six replicates’ inhibitory zone (mm) ± SD. Different letters in the column mean the inhibition zone is significantly different (*P* < 0.05). The diameter of the paper disk (6 mm) is excluded.

Figure 1 shows the robust antibacterial efficacy of TCEO against *E. coli* and *S. aureus*, as evidenced by the substantial zone of inhibition measuring 25.3 mm and 36.8 mm, respectively. These values were found to be significantly higher compared to several other tested components (*P* = 0.05). Consequently, TCEO was chosen as the optimal candidate for further investigation in the in vitro experiments.

### Part 1: microbiota

#### Bacteriological Analysis of Milk Samples

Table 1 presents the microbiological content of composite milk during the study and the total colony counts (cfu/mL). On day 28, no bacteria were identified in the milk of 4 quarters, 2 in each group. There were no statistically significant changes related to the TCEO application.

**Table 1:** Evolution of the bacteria species in milk from Control (CT) and Treated (TR) groups.

Quarter	Identification of the quarter	Day 0 (cfu/mL)	Day 7 (cfu/mL)	Day 21 (cfu/mL)	Day 28 (cfu/mL)
Control	CT1A	Corynebacterium spp. (> 1 600)	Corynebacterium spp. (> 1 600)	Corynebacterium spp. (> 1 600)	Corynebacterium spp. (330)
	CT1B	Corynebacterium spp. (> 1 600)	Corynebacterium spp. (> 1 600)	Corynebacterium spp. (> 1 600)	Corynebacterium spp. (330)
	CT2A	CNS (> 1 600)	CNS (> 1 600)	CNS (> 1 600)	CNS (330)
	CT2B	Microccocus spp. (> 1 600)	Sterile	Sterile	Sterile
	CT3	CNS (> 1 600)	CNS (> 1 600)	CNS (> 1 600)	CNS (> 1 600)
	CT4	CNS (> 1 600)	CNS (> 1 600)	CNS (> 1 600)	CNS (> 1 600)
	CT5	CNS (> 1 600)	CNS (> 1 600)	Sterile	Sterile
	CT6	CNS (> 1 600)	CNS (> 1 600)	CNS (> 1 600)	CNS (330)
Treated	TR1A	CNS (> 1 600)	CNS (> 1 600)	CNS (> 1 600)	CNS (> 1 600)
	TR1B	CNS (1 600)	Sterile	CNS (> 1 600)	Sterile
	TR2A	CNS (830)	CNS (332)	CNS (500)	CNS (332)
	TR2B	CNS (124)	CNS (> 1 600)	CNS (216)	CNS (34)
	TR3	CNS (> 1 600)	CNS (> 1 600)	CNS (> 1 600)	CNS (> 1 600)
	TR4	CNS (> 1 600)	CNS (> 1 600)	CNS (> 1 600)	CNS (> 1 600)
	TR5	CNS (1 600)	CNS (> 1 600)	CNS (> 1 600)	CNS (1 330)
	TR6	CNS (580)	CNS (332)	CNS (> 1 600)	CNS Sterile

The number of colonies is determined by total colony counts (cfu/mL). CNS: coagulase-negative Staphylococci. The notation “ > 1600 cfu/mL” indicates that the number of colonies in the Petri dish could not be quantified due to their high count.

#### Microbiota analysis

This section of the study analyzed changes in milk microbiota using meta-transcriptomics. It also discusses alterations in skin microbiota following topical treatment with TCEO. The phyla definitions are based on the Silva database 132, which does not reflect the latest bacterial nomenclature updates. As a result, terms such as Bacillus are classified under Firmicutes, Pseudomonadota under Proteobacteria, Actinomycetota under Actinobacteria, and Bacteroidota under Bacteroidetes.

#### Sequencing metrics and characterization of milk core microbiota at phylum and genus levels

- Sequencing metrics

Sequencing the V3-V4 regions of the bacterial 16S rRNA gene from 64 milk samples yielded 2,871,971 assembled reads. After filtering, 2,122,199 sequences remained, with an average retention rate of 77.4%. The Control group averaged 33,159 sequences per sample, while the Treated group had 33,302. Initially, 6,874 OTUs were identified, but after filtering out low-count OTUs, 1,880 distinct OTUs remained.

- Milk core microbiota characterization

Three main phyla were detected in the milk microbiota and were shared within all the samples: Actinomycetota (formerly named Actinobacteria, 11.7%), Bacillota (formerly named Firmicutes, 29.25%), and Pseudomonadota (formerly named Proteobacteria, 58.95%). At the genus level, 4 genera were detected in the milk microbiota, including *Cutibacterium* (10,1%), *Halomonas* (8.31%), *Methylobacterium* (49.33%), and *Pseudomonas* (27.78%). The remaining 4.48% was composed of the "uncultured or unknown" group, which was artificially composed for the aim of the statistical analysis, including all the genera that the database (SILVA v. 132) retrieved as “uncultured” or “uncultured bacterium” or “Other” or “uncultured organism” and similar.

#### Alpha- and beta-diversities in milk microbiota

The estimated alpha diversity indices for describing the richness, diversity, and evenness of the milk microbiota between the two experimental groups are reported in Supplementary Table S3.

**Fig. 2:**
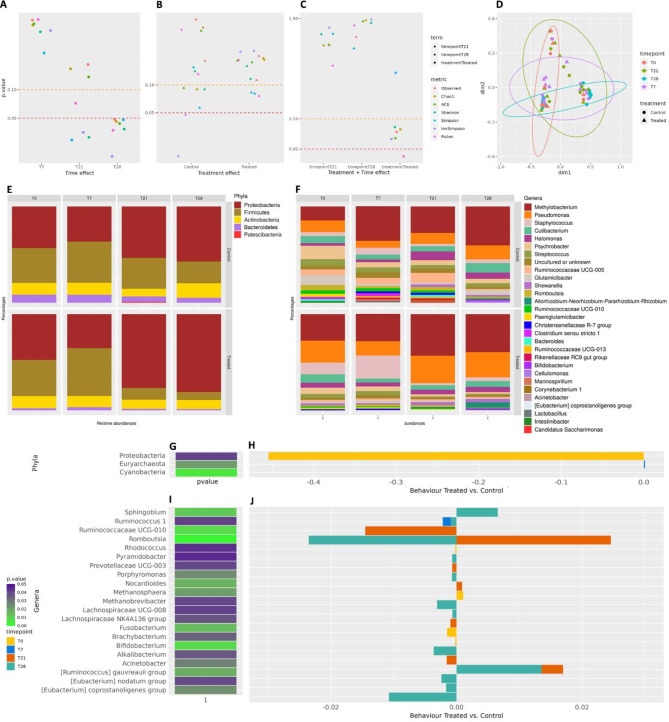
The milk microbiota analysis. (**A**) Scatterplots of *P*-values from the model (1) evaluating the effects of treatment per time; (**B**) Scatterplots of *P*-values from the model (2) the effect of time points within treatment; (**C**) Scatterplots of P-values from the model (3), or combined model. In (**A**), (**B**), and (**C**), Scatterplots refer to milk alpha-diversity indices (corrected for baseline). The dashed line in red represents the significance threshold equal to 0.05, and in orange, it is equal to 0.10. (**D**) MDS plot of Bray–Curtis dissimilarities based on the OTU table from the milk microbiota. The shape of data points represents the two groups of quarters (Control vs. Treated with EO). Colours represent the different time points instead. (**E**) Boxplots of the distribution of phylum relative abundance higher than 1% in the milk microbiota (all 64 samples together). (**F**) Boxplots of the distribution of genera relative abundance higher than 1% in the milk microbiota. Significantly abundant taxa in milk microbiota at phyla level: (**G**) Heatmap of the significance of taxa relative abundance in the milk microbiota at phyla level. (**H**) Bar plot representing the behavior of the significantly different abundant phyla along time points: the scale in the x-axis is the ratio of Treated against Controls. Please note that the scale of the graph does not make Proteobacteria clear. Significantly abundant taxa in milk microbiota at genera level: (**I**) heatmap of the significance of taxa relative abundance in the milk microbiota at the genera level. (**J**) Bar plot representing the behaviour of the significantly different abundant genera along time points: the scale in the x-axis is the ratio of Treated against Controls. The definition at the phyla level originates from Silva v. 132, which did not integrate some updates about bacterial nomenclature. Therefore, readers will find, e.g., Bacillus as Firmicutes, Pseudomonadota as Proteobacteria, Actinomycetota as Actinobacteria, and Bacteroidota as Bacteroidetes.

Once corrected to a baseline equal to T0 (milk samples collected before treatment application), alpha indices were compared in 3 different linear models to assess their behavior: i) over time points (within the group, Eq. 1), ii) between treatments (within time point, Eq. 2) and iii) between treatments accounting for time (Eq. 3) as described in the Materials section. Figure 2 reports the scatterplots of i) the significance (P) of treatment at T7, T21, and T28 (Fig. 2A), ii) the significance (P) of the time point within treatment (Fig. 2B), iii) the significance (P) of time point and treatment from the model (3) (Fig. 2C), for the different alpha diversity indices.

From model (1), the effect of time is significant: three indices (Shannon, Simpson, and InvSimpson) have a significance of *P* < 0.05 on T21, and 6 indices in the significance range between 0.05–0.10 (Shannon, Obseved_otus, Simpson, Fisher, ACE, and Chao1) plus one index with a *P* < 0.05 in T28 (InvSimpson). From model (2), significance clusters are similar for Treated and Control groups, with 1 index (InvSimpson) belonging to T21, statistically significant in the 0.05–0.10. From model (3), treatment has a clear effect over time points, with six diversity indices found with P lower than 0.05 (Observed_OTUs, Chao1, ACE, Shannon, InvSimpson, and Fisher) for the Treated group compared with the Control, influenced by time effect.

The relationships between samples were assessed based on Bray–Curtis dissimilarities from the beta diversity analysis. Figure 2D shows the distribution of samples along the first two dimensions from the multidimensional scaling (MDS) of Bray–Curtis dissimilarities: a clustering was observed between time points (*P* = 0.01211), while no clear clustering by treatment nor by animal or quarter was detected (*P* = 0.09531, *P* = 0.06507 and *P* = 0.16199 from PERMANOVA between treatment, time, animals and quarters respectively, 999 permutations).

#### Effects of the treatments and time points on milk microbiota at phyla and genera level

Five main phyla were found in the milk microbiota with a relative abundance higher than 1% along different time points (Fig. 2E): Firmicutes, Proteobacteria, Actinobacteria, Bacteoridetes, and Patescibacteria. On the other hand, thirty-four main genera were found in the milk microbiota with a relative abundance higher than 1% along time points (Fig. 2F). Only 3 phyla (Proteobacteria, Euryarchaota, and Cyanobacteria) were found to significantly differ between treatments along time points, interestingly decreasing all at T28 in the Treated group as compared to the Control group (Fig. 2G,H).

Twenty-one genera were found to differ significantly between treatments within time points. In particular, the genus *Sphingobium* and *Acinetobacter* were increased in the Treated group compared to the Control group at T21 and T28, respectively (Fig. 2I, J). Interestingly, the genus *Romboutsia* has a bimodal trend: it decreases in the Treated group compared to the Control group at T21 but increases at T28 (Supplementary Table S4). The microbial milk community behaved significantly differently along two or more time points and described their behavior in the Treated group compared with the Control.

#### Sequencing metrics of skin swab samples and alpha and beta diversity

**Fig. 3:**
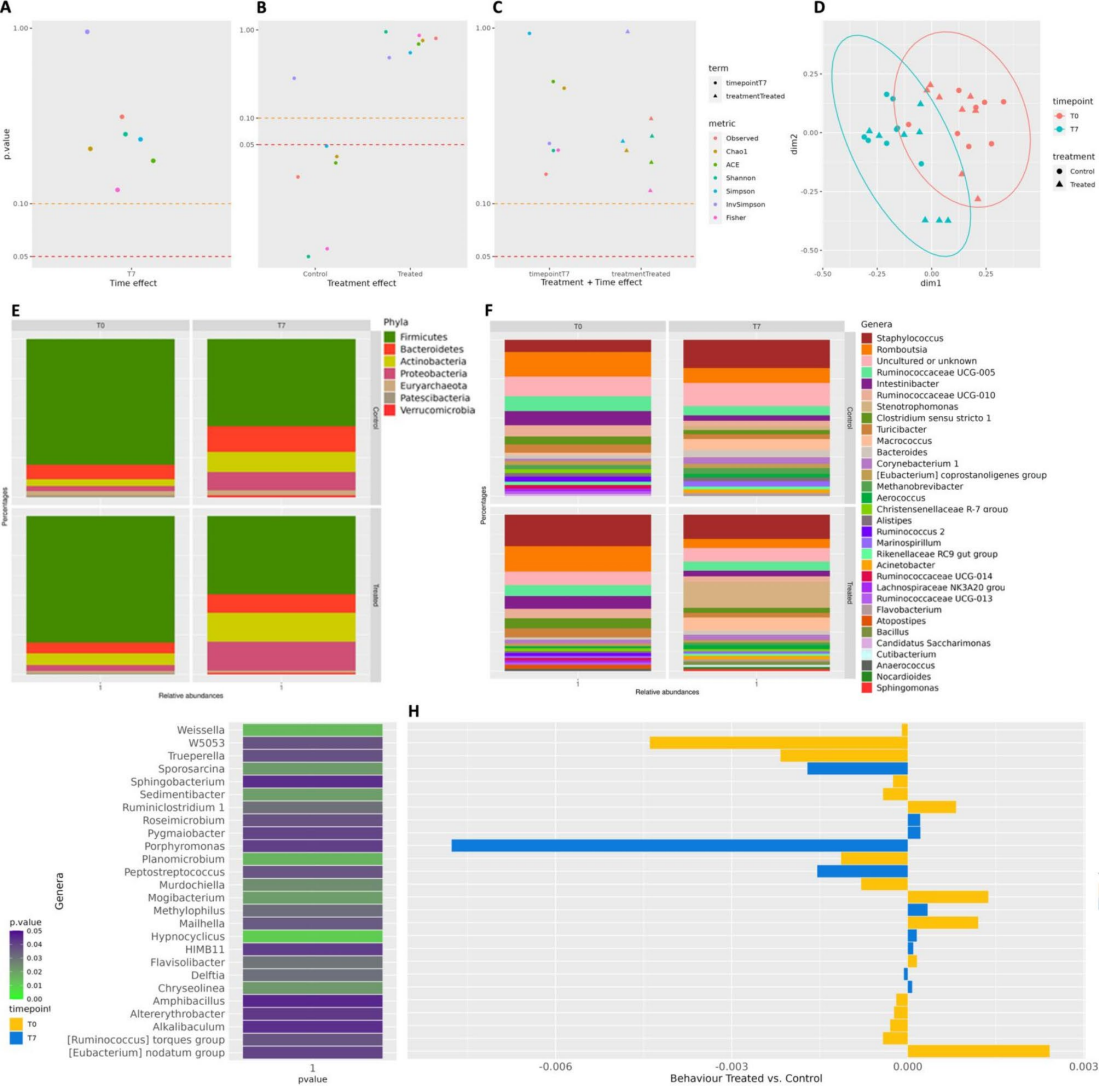
The skin microbiota analysis. (**A**) Scatterplots of Ps from the model (1) evaluating the effects of treatment per time; (**B**) Scatterplots of Ps from the model (2) the effect of time points within treatment; (**C**) Scatterplots of Ps from the model (3), or combined model. In (**A**), (**B**), and (**C**), Scatterplots refer to skin alpha-diversity indices (corrected for baseline). The dashed line in red represents the P threshold equal to 0.05, and in orange, it is equal to 0.10. (**D**) MDS plot of Bray–Curtis dissimilarities based on the OTU table from the milk microbiota. The shape of data points represents the two groups of quarters (Control vs. Treated with EO). Colours represent the different time points instead. (**E**) Boxplots of the distribution of phylum relative abundance higher than 1% in the skin microbiota (all 64 samples together). (**F**) Boxplots of the distribution of genera relative abundance higher than 1% in the skin microbiota (all 64 samples together). Significantly abundant taxa in milk microbiota: (**G**) heatmap of the P of taxa relative abundance in the milk microbiota at phyla level, (**H**) Bar plot representing the behaviour of the significantly different abundant phyla along time points. The definition at the phyla level originates from Silva 132, which did not integrate some updates about bacterial nomenclature. Therefore, readers will find, e.g., Bacillus as Firmicutes, Pseudomonadota as Proteobacteria, Actinomycetota as Actinobacteria, and Bacteroidota as Bacteroidetes.

Sequencing the V3-V4 regions of the bacterial 16S rRNA gene of the 32 udder skin swabs samples produced 675,938 assembled reads (joined R1-R2 paired-end reads). After quality filtering, 105 300 sequences were removed, leaving 570 638 sequences for subsequent analyses (85.4% average retention rate, maximum 98.1%, minimum 57.6%). On average, the Control group had 17 421 (± 9 085) sequences per sample and 17 832 (± 9 029) in the Treated group. The initial number of OTUs identified was 11,255; after filtering out OTUs with less than 10 counts in at least 2 samples, 3326 distinct OTUs were left. Figure 3 reports the scatterplots of i) the significance (P) of treatment at T7, (Fig. 3A), ii) the significance (P) of the time within treatments (Fig. 3B), iii) the significance (P) of time and treatments from the model (3) (Fig. 3C), for the different alpha diversity indices. Details about indices are reported in Supplementary Table S5.

From model (1), the effect of time appears not significant when comparing T7 against T0, which is used as the baseline. On the contrary, from the model (2), it is possible to see that, considering treatments only, Control groups show significance while the Treated group did not (6 indices in *P* < 0.05: Observed_OTUs, Chao1, ACE, Shannon, Simpson, and Fisher). From model (3), it was possible to observe that combining time and treatment effect, no significance is evidenced, suggesting that the significance in Fig. 3A is strictly related to treatment. Bray–Curtis dissimilarities method was used to assess the relationship between samples for the beta-diversity analysis (Fig. 3D): results showed that samples tend to cluster significantly by time point, while not per treatment nor when considering time and treatment effect together (*p* = 0.001 for time, 0.311 for treatment and 0.18 for time and treatment combined effect, from PERMANOVA, 999 permutations).

#### Effects of the treatments and time points on skin microbiota at phyla and genera level

Figure 3E summarizes the skin microbiota at the phyla and genera level, showing that Seven main phyla were found in the skin microbiota with a relative abundance higher than 1% (Firmicutes, Proteobacteria, Actinobacteria, Bacteoridetes, Euryarchaota, Verrucomicrobia, and Patescibacteria. For the main genera, 31 were found in the skin microbiota with a relative abundance higher than 1% along time points (Fig. 3F) among them 26 genera significantly differed between treatments along time points, (Supplementary Table S6) (Fig. 3G,H).

### Part 2: Milk quality

#### Composition, physical, and organoleptic properties

**Fig. 4:**
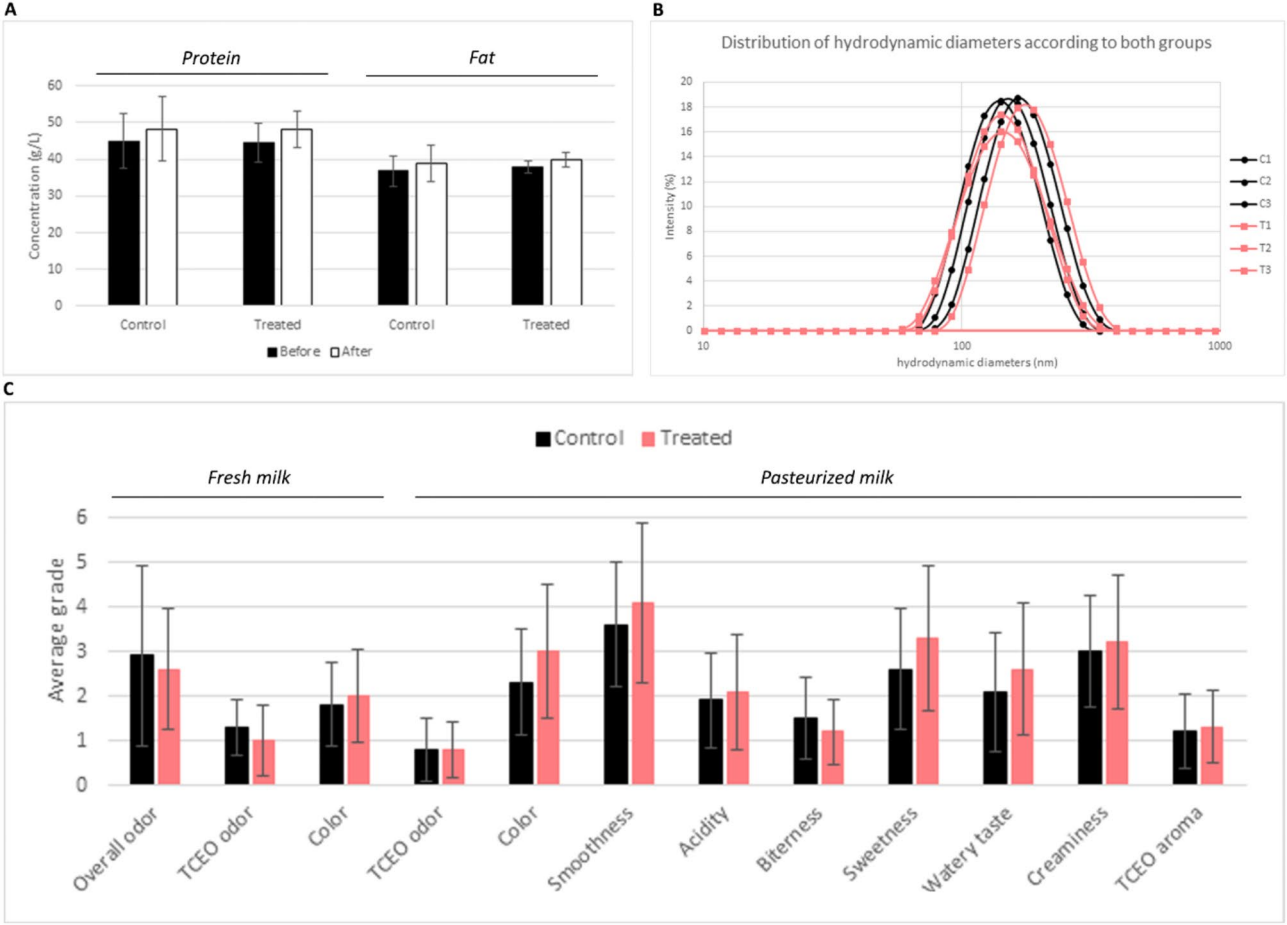
The milk quality evaluation. (**A**) Evolution of protein and fat content in the milk before and after the treatment for both groups. (**B**) Distribution of the hydrodynamic diameters of casein micelle in milk in both groups (C for the Control group and T for the treatment group). Each curve corresponds to 1 sampled quarter (3 per condition). (**C**) Distribution of the average grades attributed by the panel of experts to fresh and pasteurized milk from Control and Treated cows. No significant differences were observed between the 2 groups of samples (*P* = 0.05).

The overall composition of milk and its sensory properties were not affected by the treatment with TCEO (Fig. 4A,C). The average hydrodynamic diameter of the casein micelles varied between 140 and 170 nm, and the viscosity ranged from 2.2 to 3.4 mPa∙s at 20 °C (Fig. 4B) without any difference between the two groups (*p* = 0.05).

### Part 3: lipidomic analysis

#### Lipidome profile and analysis

The untargeted lipidome in milk was determined following a liquid chromatography-quadrupole time-of-flight mass spectrometry approach. We identified i2450 lipid species. Only the ones over a cut-off of 5000 (684 species) were considered for further analysis. The lipids were classified into 18 classes, namely acyl steryl glycosides (ASG [10 species]), N-Acylethanolamine (NAE [25 species]), triacylglycerols (TAG [23 species]), diacylglycerols (DAG [97 species]), monoacylglycerols (MAG [12 species]), phosphatidylserine (PS [48 species]), sphingomyelin (SM [52 species]), phosphatidylinositol (PI [35 species]), phosphatidylethanolamine (PE [128 species]), phosphatidylcholine (PC [82 species]), lysophosphatidylethanolamine (LPE [14 species]), lysophosphatidylcholine (LPC [10 species]), lysophosphatidylinositol (LPI [3 species]), ceramides (Cer [27 species]), hexosylceramides (HexCer [35 species]), galactosylceramide sulfate (SHexCer [18 species]), acylcarnitines (AcCarn [5 species]) and fatty acids (FA [60 species]). A list of the species is also reported in Table 2.

**Table 2:** Number of species detected in milk from subclinical mastitis.

Class	Acronym	Number of species
Acyl steryl glycosides	ASG	10
N-Acylethanolamine	NAE	25
Triacylglycerols	TG	23
Diacylglycerols	DG	97
Monoacylglycerols	MG	12
Phosphatidylserine	PS	48
Sphingomyelin	SM	52
Phosphatidylinositol	PI	35
Phosphatidylethanolamine	PE	128
Phosphatidylcholine	PC	82
Lysophosphatidylethanolamine	LPE	14
Lysophosphatidylcholine	LPC	10
Lysophosphatidylinositol	LPI	3
Ceramides	Cer	27
Hexosylceramides	HexCer	35
Galactosylceramide sulfate	SHexCer	18
Acylcarnitines	AcCarn	5
Fatty acids	FA	60

Milk lipid primary function and significance description have been previously reviewed^49^.

The list of lipids identified in milk samples is presented in Supplementary Table S7.

https://unimibox.unimi.it/index.php/s/zPws6CxNWRYFXTd

**Fig. 5:**
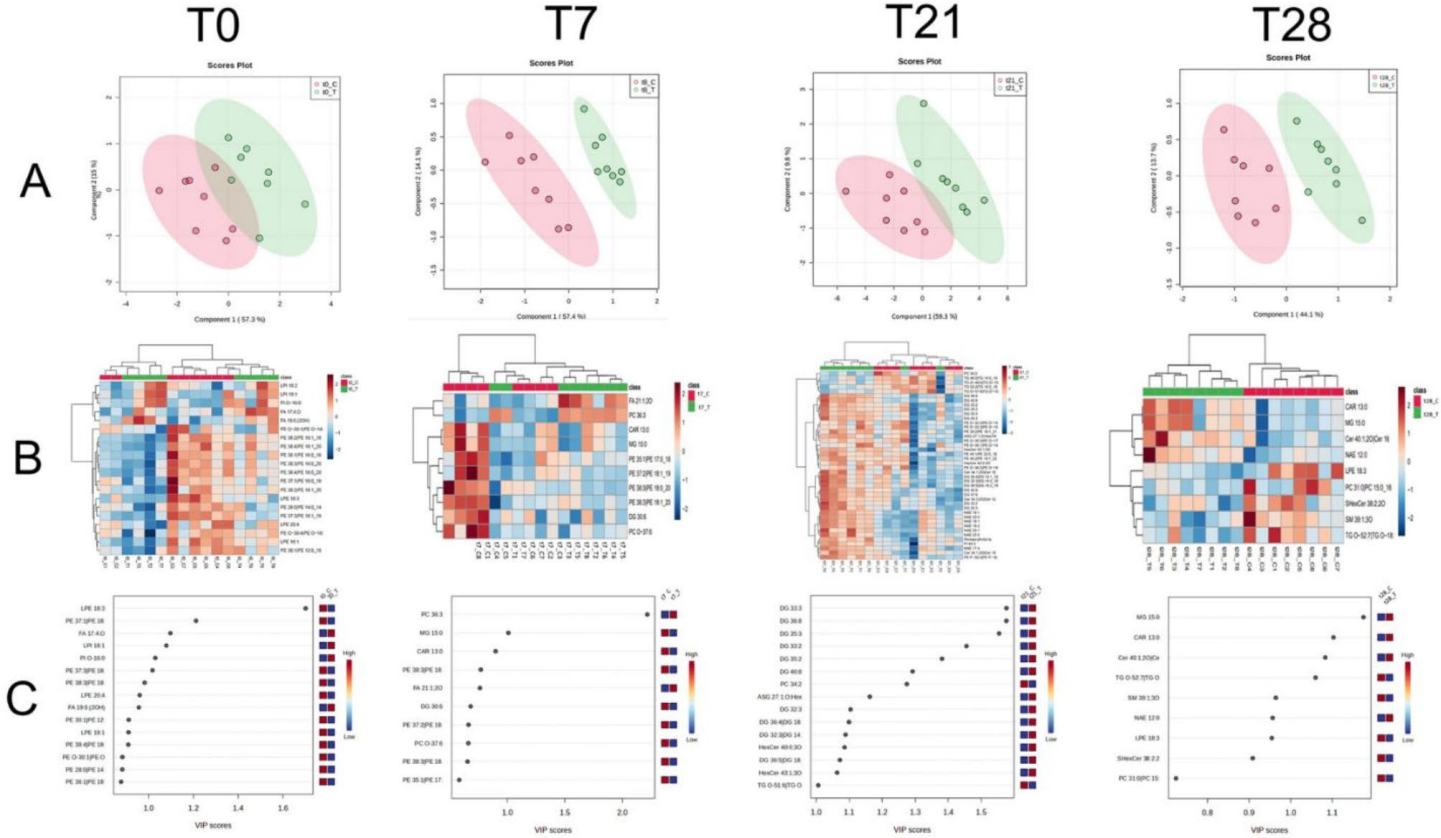
The metabolome analysis of milk. Panel A Partial Least Squares—Discriminant Analysis (PLS-DA). Panel B: Heat maps that include the important features identified as statistically significant (*P* < 0.05) after a t-test. Panel C: the most important lipids shown to be differentially abundant between treated and untreated quarters were as variable importance in the projection (VIP) score.

Figure 5 presents the Partial Least Squares—Discriminant Analysis (PLS-DA) that measured the difference in the milk lipidome from Treated and non-treated mammary gland quarters along the time points T0, T7, T21, and T28 (Panel A). The results showed that the scatters of the two groups were limitedly separated across PC1 and PC2 at T0, indicating a quite homogeneity of the two groups. The effect of the treatment became more evident with time, as presented in panel A. Panel B shows the Heat maps that include the important features identified as statistically significant (*p* < 0.05) after a t-test. The most critical lipids, shown to be differentially abundant between Treated and Control quarters, were presented in panel C as variable importance in the projection (VIP) score.

At T7, most of the lipids that changed between EO-treated and Control groups (8 out of 10) had a lower concentration in the milk of Treated cows. Of the ten lipids whose abundance changed, the most noticeable feature is an increase of PC 36:3. At T21, 15 lipids changed their abundance: two decreased, and 13 increased with treatment. Most changes were reported in the family of diglycerides, namely DG 33:3, DG 36:8, DG 35:3, DG 33:2, DG 35.2, DG 40:8, and PC 34:2 (VIP score > 1). At 28, the effects of TCEO on milk lipidome were limited to only 9 lipids (four increased and five decreased their abundance), the most interesting feature being MG 15:0, which exhibited an increased abundance after TCEO.

### Part 4: Milk inflammatory parameters

#### SCC of milk after TCEO treatment

The changes in somatic cells in composite milk after TCEO are presented in Supplementary Figure S8. No statistically significant differences were detected after TCEO treatment (*P* = 0.05).

#### Milk concentration of IL-8 and the acute phase proteins Lf and Hp

The concentration of IL-8 was not homogeneous at T0. Only 2 milk samples from the Control and 3 from the Treated groups contained a concentration of IL-8 higher than 50 pg/mL. Nevertheless, no significant (*p* = 0.05) difference was observed between the two groups for this parameter.

**Fig. 6:**
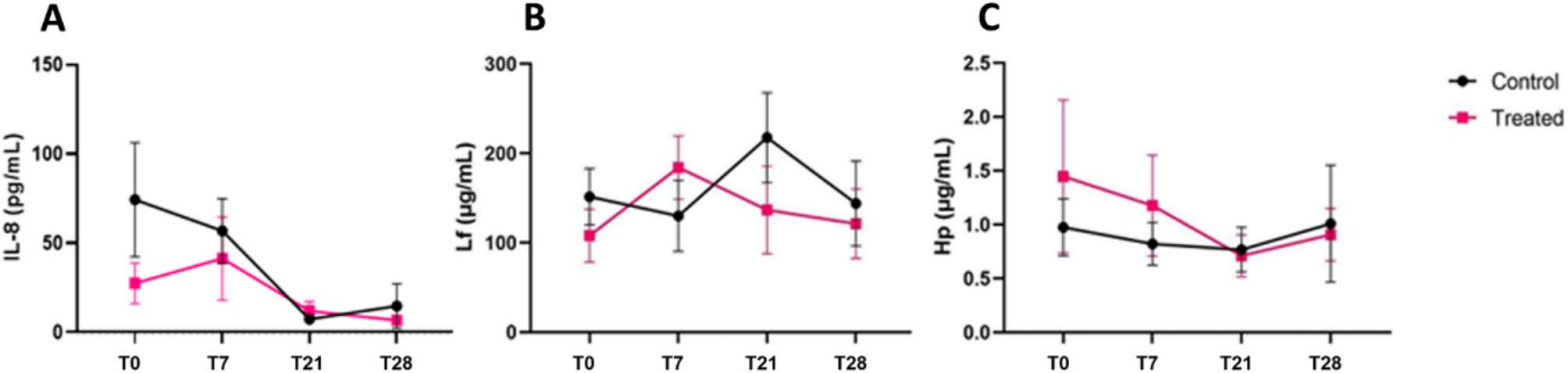
The milk inflammatory parameters. (**A**) Time course of the serum concentrations (means + SEM) of Interleukine 8 (IL-8)*. (**B**) Time course of the serum concentrations (means + SEM) of acute phase proteins Lactoferrin (LF)*. (**C**) Time course of the serum concentrations (means + SEM) of acute phase proteins Haptoglobin (Hp)*. *In quarter milk all the 4 time points (T0, T7, T21, and T28).

Lactoferrin (Lf) concentrations ranged between 143 and 217 μg/mL (Fig. 6B), and a significant interaction between treatment and time points was found (*p* = 0.0002), indicating an effect of TCO on Lf. For Haptoglobin (Hp), the values ranged from 0.7 to 1.4 μg/mL for the two groups (Fig. 4C) without any significant difference between the treatments (*p* = 0.05).

## Discussion

The present study investigated the impact of topical treatment with TCEO on the mammary gland of healthy cows at the end of the lactation period, which is one of the most delicate phases of the life cycle of a cow and its udder due to the involution and remodeling of the gland to quiescence. The effects of this treatment were unraveled using a systems biology approach. The changes in milk and skin microbiota were determined by microbiological and uncultured microbiota analysis by NGS. The study further included milk quality investigation in terms of composition, sensory analysis, viscosity, the hydrodynamic diameter of casein micelle evaluation, lipidomic profiling, and measurements of the significant markers of inflammation in milk.

The first part of the study investigated the potential changes in milk culturable microbial content and the uncultured bacterial population of milk and skin after TCEO treatment. The treatment did not statistically affect the milk’s culturable microbial content significantly. Although these results were inconsistent with the in vitro studies that show antibacterial properties of TCEO^10,12,13,16–19,50,51^, it can be hypothesized that the surface treatment was less effective than the direct treatment in the teats previously cited in the literature. Different theories could justify this event: i) the oxidation and degradation of TCEO during the massage negatively affecting the TCEO antibacterial properties^44^, or ii) the concentration of TCEO that penetrated the quarter and arrived at the cistern was below the minimum inhibitory concentration (MIC) of the bacteria, or iii) TCEO was captured by the milk’s fat. However, it is interesting that results from the culturable microbial analysis and microbiota study were coherent, as the CNS signaled in the first analysis were then easily visible between the genera found in the microbiota analysis (Fig. 2F). On the other hand, metagenomics analysis showed that the investigated treatment induced limited changes in the uncultured milk microbiota: the significance in alpha diversity indexes referred, in fact, to time (Fig. 2A, 3 indices statistically significant at T21, and 6 indices with P between 0.05 and 0.1, plus 1 index with P lower than 0.05 at T28) and to group-related time effect (Fig. 2C, 6 indices statistically significant for treatment), suggesting that changes were related to time effect within the milk ecosystem, rather than to a treatment effect.

Similarly, the species diversity between the two communities, described by Bray–Curtis dissimilarities analysis and statistically tested with PERMANOVA, showed significance only when related to time. The milk microbiota confirmed the substantial lack of significant changes between the two groups at both phyla and genus levels. Milk microbiota composition at the phylum level was consistent with previous reports^52^, with no significant treatment-related changes. However, it is possible to observe little changes at the genus level, mainly at T21 and T28. At T21, a decrease of *Eubacterium coprostanoligenes* and *Romboutsia* was found, and an increase of *Acinetobacter*, among others, in the TCEO-treated groups compared to control groups. The TCEO at T28 featured an opposite trend of *Romboutsia*, which increased and decreased the amount of *Ruminococcus 1*. *Acinetobacter* amount further increased: the Acinetobacter genus is related to clinical mastitis^53^, but still, small signals in inflammatory parameters were found, thus suggesting that the increase in the abundance of this genus has no practical consequences on the health status of the mammary gland. Its presence could be interesting due to its influence on cheese production, as it is reported to influence, as an external contaminant linked with silage, the cheese sensory characteristics^54^.

Similarly, *Brachybacterium*, *Corynebacterium,* and *Brevibacterium* were found to be initially lower in the Treated group but to increase to a non-significant difference between the two treated groups in the following time points. This behavior from these genera, which were previously signaled in the literature for their benefit on milk and cheese production, suggests that EO treatment does not influence them. On the contrary, they seem advantaged, guaranteeing healthy dairy production. Coherently, this beneficial status could favor the use of milk for cheese production, as EO has already been reported as an effective preservative against *Listeria monocytogenes* spoilage^55–57^.

Similarly, changes in the uncultured bacterial population diversity at the skin level were limited, as shown by the statistically significant differences of alpha diversity indices only when related to treatment (Fig. 3B) but not when related to time nor when considering time and treatment effects together. It is important to consider that the application method could have influenced the skin microbiota more than the treatment. Bacterial species diversity between the two groups was equally significant only when related to and was not affected by the treatment. Changes at phylum and genus levels between the two groups were similarly limited: phyla did not present any significant change. The Genera that was found increased in the treated vs. control group at T7 and did not reveal potential harmful species, in agreement with previous results reporting no damaging effect of the EO^58,59^. In conclusion, these results show that TCEO udder application did not induce significant changes in the uncultured microbiota in milk and udder skin.

The second part of the study evaluated the effects of TCEO application on milk quality, including milk composition and sensory analysis. Concerning the physical properties of milk, the average casein micelle size and milk viscosity are, among others, critical physical parameters that affect the processing of milk into other milk-based products (cheese, yogurt, etc.). No significant difference (*P* > 0.05) was highlighted after the TCEO application, suggesting that TCEO did not interact with milk casein biosynthesis, which is known to influence milk viscosity. The viscosity values (from 2.2 to 3.4 mPa∙s at 20 °C) are slightly higher than that classically reported for skimmed bovine milk^60^. The high measured viscosity of some samples was attributed to the presence of coagulated residual fat particles that did not completely dissolve in milk upon thawing^61^.

Consistently, with the limited changes reported for milk quality and microbial content, untargeted lipidome, to which the third part of the study was dedicated, was limitedly affected by TCEO. As described for the microbiota, most differences were detected at T21 and T28, suggesting that TCEO may delay some slight changes mainly limited to diacylglycerols. Diacylglycerols are important milk components, accounting for about 2% of the milk fraction^62^. Diacylglycerols provide a crucial intermediate element before the final step of triglyceride synthesis in the biosynthesis of milk triglycerides. This step is catalyzed by the enzyme diacylglycerol acyl-CoA acyltransferase 1 (DGAT1), a promising candidate gene for milk production traits. It would be interesting to ascertain if EO affected the enzyme’s activity. It has been shown that feeding with EO induced changes in other species’ Triacylglycerol (TAG) metabolism, such as Japanese quails^63^. Further studies are required to ascertain if skin treatment with EO may fulfill the same function in the ruminant mammary gland.

As for completeness, this study evaluated milk inflammatory parameters in its fourth part: TCEO treatment didn’t affect the inflammation process, showing any changes in IL-8, Hp, and Lf.

## Limitations

The authors acknowledge that the study included some unforeseen limitations, which should be considered while improving this pilot study to a more extensive one. Firstly, the size of the animal groups was limited, although aligned with similar studies^45,64–66^ as intended by a pilot study, but can be further increased to better mitigate the limitations. The authors acknowledge that the low numbers considered, although sufficient for the statistical analysis, can weaken the analysis outcomes, particularly given the preliminary nature of this study. Furthermore, the concentration of TCEO or its constituents in the milk was not analyzed, which represents an improvement for further studies. Lastly, the effect of TCEO massage on SCC was assessed at the udder level rather than on the individual quarter, as it is good practice in this kind of study. Future studies can include improvements to ameliorate the overall results, starting from these reported limitations.

## Conclusions

TCEO topical application on the udder did not modify milk microbiota. At the concentration of 10% TCEO, no significant change was evidenced in skin microbiota or milk lipidomic. In addition, no modifications were observed in milk composition or physicochemical properties, and somewhat similar levels of inflammatory markers were detected in both Treated and Control animal milk quarters. These results suggest that *Thymus capitatus* at the concentration of 10% is safe and has no side effects on milk when used in dairy farms as an antimicrobial alternative. These effects must be considered preliminary since a more focused study on the single-quarter milk quality is required to attest to the safety of TCEO in healthy dairy cows.

For future perspective, given the fact that the long-term impacts of EOs on gut microbiome development, particularly in early life, remain less explored, EO supplementation’s ability to modulate microbial colonization to enhance animal immunity and protect against mastitis through early nutrition in dairy cows^67^ could offer another strategy to improving cow’s mammary health. This can make EO supplementation a valuable tool in shaping the milk production sector’s future sustainability.

## Electronic supplementary material

Below is the link to the electronic supplementary material.

Supplementary Material 1

Supplementary Material 2

Supplementary Material 3

Supplementary Material 4

Supplementary Material 5

Supplementary Material 6

Supplementary Material 7

Supplementary Material 8

## Data Availability

The milk and skin swab 16S rRNA gene sequencing raw data generated and analysed during the current study are available in the UNIMI Dataverse repository (Milk dataset: 10.13130/RD_UNIMI/GMPFOB, Skin dataset: 10.13130/RD_UNIMI/OPMKOC).

## Abbreviations

AMR: Antimicrobial resistance
APP: Acute phase proteins
CNS: Coagulase-negative staphylococci
CT: Control
EO: Essential oils
HP: Haptoglobin
IL-8: Interleukin 8
Lf: Lactoferrin
MDS: Multidimensional scaling
P: Significance
SCC: Somatic cell count
TCEO: *Thymus capitatus* essential oils
TR: Treated
OUT: Operational taxonomic unit
