# Supplementary material for: The effects of Thymus capitatus essential oil topical application on milk quality: a systems biology approach
Source: Sci Rep. 2025 Feb 7;15:4627. doi: 10.1038/s41598-025-88168-0 (PMC11805959; doi:10.1038/s41598-025-88168-0)
Supplement: Supplementary file 1 — Supplementary Material 1 [file 41598_2025_88168_MOESM1_ESM.docx]

**Supplementary Table S1**. Table of data regarding animals selected for this study. Cows were identified with the code CT or TR depending on the belonging to the control or treated group respectively, followed by an increasing group-dependent numbering. In the cases of two quarters considered on the same animal, the samples were differentiated adding a letter to the ID (A and B). CNS: coagulase-negative Staphylococci. The notation "> 1600 cfu/mL" indicates that the number of colonies in the Petri dish could not be quantified due to their very high count. SCC corresponds to the somatic cell count in milk.

| **Group** | Control | | | | | | | | Treated | | | | | | | |
| --- | --- | --- | --- | --- | --- | --- | --- | --- | --- | --- | --- | --- | --- | --- | --- | --- |
| **Animal ID** | CT1A | CT1B | CT2A | CT2B | CT3 | CT4 | CT5 | CT6 | TR1A | TR1B | TR 2A | TR 2B | TR3 | TR4 | TR5 | TR6 |
| **Parity** | 2 | | 2 | | 2 | 4 | 3 | 2 | 3 | | 4 | | 2 | 1 | 4 | 3 |
| **Average daily production in liters** | 25.3 | | 14.1 | | 16 | 18.1 | 21.5 | 21.8 | 17.2 | | 13.9 | | 21.9 | 21.2 | 24.6 | 16.5 |
| **Composite SCC × 10^3^ per ml** | 276 | | 84 | | 133 | 145 | 166 | 139 | 159 | | 120 | | 215 | 140 | 197 | 140 |
| **Microbiological content, number (cfu/ml)** | Corynebacterium, > 600 | Corynebacterium, >1600 | CNS, >1600 | *Microccocus spp*, 1600 | CNS, >1600 | CNS, >1600 | CNS, >1600 | CNS, >1600 | CNS, >1600 | CNS, 1600 | CNS, 830 | CNS, 124 | CNS, >1600 | CNS, >1600 | CNS, 1600 | CNS, 580 |
