# Supplementary material for: The effects of Thymus capitatus essential oil topical application on milk quality: a systems biology approach
Source: Sci Rep. 2025 Feb 7;15:4627. doi: 10.1038/s41598-025-88168-0 (PMC11805959; doi:10.1038/s41598-025-88168-0)
Supplement: Supplementary file 2 — Supplementary Material 2 [file 41598_2025_88168_MOESM2_ESM.docx]

**Supplementary Table S2:** ESI and mass spectrometer parameters.

| **Item** | **Lipidomic IDA POS** | **Lipidomic IDA NEG** |
| --- | --- | --- |
| Ionization | POS | NEG |
| Source temperature | 350 °C | 350 °C |
| Curtain Gas (CUR) | 35 | 35 |
| GS 1 | 55 | 55 |
| GS 2 | 65 | 65 |
| Ion Spray Voltage | 4500 V | -4500 V |
| Declustering Potential (DP) | 50 V | -50 V |
| Collision Energy | 35V | -35V |
| Collision Energy Spread | 15 | 15 |
| TOF MS Mass Range | 140-1500 Da | 140-1500 Da |
| IDA acquisition Mass Range | 50-1500 Da | 50-1500 Da |
| Top N | 18 | 10 |

CUR (Curtain Gas) refers to the gas flow between the curtain plate and the orifice.

DP (Declustering Potential) provides a measure of the voltage applied to the orifice to minimize the solvent clusters that may remain on the sample ions after they enter the vacuum chamber

GS1(Nebulizer Gas): is the gas used for droplet formation.

GS2 (Heating Gas) promotes desolvation and supports the ion evaporation process to condense the charged analyte and produce gas phase ions.

IS (IonSpray Voltage) s the voltage applied between the needle and orifice plate, creating strong electric field that pulls the formed ions into the analyzer.

CE (Collision Energy) is the potential difference between Q0 and Q2 (collision cell). The collision energy (CE) and the collision energy spread define the fragmentation pattern of a peptide at a given charge state
