# Supplementary material for: The effects of Thymus capitatus essential oil topical application on milk quality: a systems biology approach
Source: Sci Rep. 2025 Feb 7;15:4627. doi: 10.1038/s41598-025-88168-0 (PMC11805959; doi:10.1038/s41598-025-88168-0)
Supplement: Supplementary file 3 — Supplementary Material 3 [file 41598_2025_88168_MOESM3_ESM.docx]

**Supplementary Table S3**: The estimated alpha diversity indices for describing the richness, diversity, and evenness of the milk microbiota within the two experimental groups per index per time point of milk samples

| **Group** | **Time point** | **Observed** | **Chao1** | **Ace** | **Shannon** | **Simpson** | **Invsimpson** | **Fisher** |
| --- | --- | --- | --- | --- | --- | --- | --- | --- |
| Control | T0 | 470.2 | 563.2 | 552.1 | 4.9 | 0.9 | 50.8 | 128.1 |
|  | T7 | 374.8 | 461.4 | 452.7 | 4.5 | 0.9 | 43.9 | 100.0 |
|  | T21 | 291.2 | 361.2 | 347.6 | 3.4 | 0.8 | 12.1 | 69.8 |
|  | T28 | 203.3 | 248.3 | 243.2 | 3.1 | 0.8 | 12.0 | 43.1 |
| Treated | T0 | 410.3 | 504.9 | 496.5 | 4.5 | 0.9 | 40.1 | 107.9 |
|  | T7 | 330.8 | 402.8 | 395.5 | 3.9 | 0.8 | 32.0 | 85.8 |
|  | T21 | 385.6 | 481.6 | 479.2 | 4.5 | 0.9 | 39.6 | 115.6 |
|  | T28 | 400.8 | 466.4 | 464.4 | 4.3 | 0.9 | 37.8 | 98.9 |
