# Supplementary material for: The effects of Thymus capitatus essential oil topical application on milk quality: a systems biology approach
Source: Sci Rep. 2025 Feb 7;15:4627. doi: 10.1038/s41598-025-88168-0 (PMC11805959; doi:10.1038/s41598-025-88168-0)
Supplement: Supplementary file 4 — Supplementary Material 4 [file 41598_2025_88168_MOESM4_ESM.docx]

**Supplementary Table S6**: List of the genera belonging to the skin microbial community.

| **Genus** | **Timepoint** | **P** | **Treated versus Control behaviour ratio** |
| --- | --- | --- | --- |
| Weissella | T0 | 0,01 | -0,00010 |
| Planomicrobium | T0 | 0,02 | -0,00114 |
| Sedimentibacter | T0 | 0,02 | -0,00042 |
| Mogibacterium | T0 | 0,02 | 0,00137 |
| Murdochiella | T0 | 0,02 | -0,00079 |
| Flavisolibacter | T0 | 0,03 | 0,00016 |
| Ruminiclostridium 1 | T0 | 0,03 | 0,00082 |
| Mailhella | T0 | 0,04 | 0,00120 |
| [Ruminococcus] torques group | T0 | 0,04 | -0,00042 |
| W5053 | T0 | 0,04 | -0,00439 |
| Trueperella | T0 | 0,04 | -0,00217 |
| [Eubacterium] nodatum group | T0 | 0,04 | 0,00242 |
| Altererythrobacter | T0 | 0,04 | -0,00023 |
| Alkalibaculum | T0 | 0,04 | -0,00030 |
| Sphingobacterium | T0 | 0,04 | -0,00025 |
| Hypnocyclicus | T7 | 0,01 | 0,00015 |
| Sporosarcina | T7 | 0,02 | -0,00171 |
| Chryseolinea | T7 | 0,02 | 0,00008 |
| Delftia | T7 | 0,03 | -0,00007 |
| Methylophilus | T7 | 0,03 | 0,00034 |
| Peptostreptococcus | T7 | 0,04 | -0,00154 |
| Roseimicrobium | T7 | 0,04 | 0,00021 |
| Pygmaiobacter | T7 | 0,04 | 0,00022 |
| Porphyromonas | T7 | 0,04 | -0,00777 |
| HIMB11 | T7 | 0,04 | 0,00009 |
