# Supplementary material for: The effects of Thymus capitatus essential oil topical application on milk quality: a systems biology approach
Source: Sci Rep. 2025 Feb 7;15:4627. doi: 10.1038/s41598-025-88168-0 (PMC11805959; doi:10.1038/s41598-025-88168-0)
Supplement: Supplementary file 5 — Supplementary Material 5 [file 41598_2025_88168_MOESM5_ESM.docx]

**Supplementary Table S4**: List of the genera belonging to the microbial milk community. In bold the genera presenting changes in more than 1 time point.

| **Genus** | **Timepoint** | **P** | **Treated versus Control behaviour ratio** |
| --- | --- | --- | --- |
| *Fusobacterium* | T0 | 1,39E+14 | -1,51E+14 |
| *Methanosphaera* | T0 | 2,08E+14 | 1,06E+14 |
| *Brachybacterium* | T0 | 3,39E+14 | -1,91E+14 |
| *Rhodococcus* | T0 | 4,43E+14 | -1,79E+14 |
| ***Ruminococcus 1*** | **T7** | **4,07E+14** | **-1,28E+13** |
| ***Romboutsia*** | **T21** | **8,50E+14** | **2,46E+14** |
| *Ruminococcaceae UCG-010* | T21 | 7,63E+14 | -1,45E+14 |
| *Nocardioides* | T21 | 1,67E+14 | 8,91E+14 |
| ***Acinetobacter*** | **T21** | **2,72E+14** | **3,44E+14** |
| *Alkalibacterium* | T21 | 3,61E+14 | -1,57E+14 |
| *Lachnospiraceae NK4A136 group* | T21 | 3,68E+14 | -9,76E+14 |
| *Prevotellaceae UCG-003* | T21 | 3,95E+14 | -6,92E+14 |
| ***Romboutsia*** | **T28** | **2,56E+13** | **-2,35E+14** |
| *Bifidobacterium* | T28 | 8,56E+14 | -3,65E+13 |
| *Sphingobium* | T28 | 1,16E+14 | 6,58E+14 |
| ***Ruminococcus 1*** | **T28** | **1,33E+14** | **-9,19E+14** |
| ***Acinetobacter*** | **T28** | **1,58E+14** | **1,35E+14** |
| *[Ruminococcus] gauvreauii group* | T28 | 1,73E+14 | -2,41E+14 |
| *[Eubacterium] coprostanoligenes group* | T28 | 2,41E+14 | -1,08E+14 |
| *Porphyromonas* | T28 | 2,51E+14 | -7,34E+14 |
| *[Eubacterium] nodatum group* | T28 | 3,85E+14 | -1,66E+14 |
| *Methanobrevibacter* | T28 | 3,96E+14 | -3,10E+14 |
| *Lachnospiraceae UCG-008* | T28 | 4,07E+14 | -6,54E+14 |
| *Pyramidobacter* | T28 | 4,49E+14 | -6,98E+14 |
