# Supplementary material for: The effects of Thymus capitatus essential oil topical application on milk quality: a systems biology approach
Source: Sci Rep. 2025 Feb 7;15:4627. doi: 10.1038/s41598-025-88168-0 (PMC11805959; doi:10.1038/s41598-025-88168-0)
Supplement: Supplementary file 6 — Supplementary Material 6 [file 41598_2025_88168_MOESM6_ESM.docx]

**Supplementary Table S5**: Alpha diversity values per index per time point for the microbiota of udder skin samples

| **Treatment** | **Time point** | **Observed^[[1]](#footnote-1)^** | **Chao1^[[2]](#footnote-2)^** | **Ace** | **Shannon^[[3]](#footnote-3)^** | **Simpson^[[4]](#footnote-4)^** | **Invsimpson^[[5]](#footnote-5)^** | **Fisher** |
| --- | --- | --- | --- | --- | --- | --- | --- | --- |
| Control | T0 | 1230.75 | 1586.1 | 1625.0 | 5.3 | 0.9 | 44.8 | 354.4 |
| Control | T7 | 1517.4 | 1872.8 | 1920.5 | 5.7 | 0.9 | 69.1 | 464.1 |
| Treated | T0 | 1122.6 | 1509.6 | 1534.2 | 5.3 | 0.9 | 57.8 | 342.4 |
| Treated | T7 | 1236.4 | 1461.2 | 1469.6 | 5.4 | 0.9 | 67.4 | 336.4 |

1. The total number of unique species observed in a sample. [↑](#footnote-ref-1)
2. Estimates the total number of species in a community, especially when rare species are underrepresented in the sample [↑](#footnote-ref-2)
3. Measures both richness and evenness. A higher Shannon index indicates a community with more species and a more even distribution of individuals among those species [↑](#footnote-ref-3)
4. Focuses on the dominance of the most common species. A lower Simpson index suggests a more diverse community with fewer dominant species. [↑](#footnote-ref-4)
5. The inverse of the Simpson index, providing a measure of diversity that increases with the number of species and their evenness. [↑](#footnote-ref-5)
