# Supplementary material for: The effects of Thymus capitatus essential oil topical application on milk quality: a systems biology approach
Source: Sci Rep. 2025 Feb 7;15:4627. doi: 10.1038/s41598-025-88168-0 (PMC11805959; doi:10.1038/s41598-025-88168-0)
Supplement: Supplementary file 7 — Supplementary Material 7 [file 41598_2025_88168_MOESM7_ESM.docx]

| **Supplementary Table S7: Lipid that changed their abundance in a significant way for more than 10 folds**  **Values are given as fold changes as compared to internal standards. The list of abbreviations is at the end of the table** | | | | | | | | | |
| --- | --- | --- | --- | --- | --- | --- | --- | --- | --- |
|  | | | | | | | | | |
|  |  |  |  |  |  |  |  |  |  |
| **Metabolyte name** | **t0_C1** | **t0_C2** | **t0_C3** | **t0_C4** | **t0_C5** | **t0_C6** | **t0_C7** | **t0_C8** | **average value** |
| **Acyl sterol glycoside** |  |  |  |  |  |  |  |  |  |
| ASG 27:1;O;Hex;FA 2:0 | 102 | 135 | 105 | 388 | 315 | 313 | 182 | 152 | 211 |
| ASG 28:1;O;Hex;FA 14:0 | 846 | 243 | 8029 | 1546 | 929 | 535 | 1326 | 449 | 1738 |
| ASG 28:1;O;Hex;FA 16:0 | 845 | 160 | 8023 | 1230 | 806 | 354 | 1082 | 327 | 1603 |
| ASG 28:1;O;Hex;FA 18:0 | 474 | 116 | 5715 | 965 | 329 | 318 | 866 | 176 | 1120 |
| ASG 28:1;O;Hex;FA 20:1 | 522 | 310 | 4976 | 1167 | 990 | 609 | 1635 | 372 | 1322 |
| ASG 28:2;O;Hex;FA 14:0 | 1459 | 496 | 9936 | 2284 | 2174 | 1462 | 2799 | 1345 | 2744 |
| ASG 28:2;O;Hex;FA 16:0 | 787 | 275 | 5357 | 1547 | 763 | 697 | 1718 | 528 | 1459 |
| ASG 28:2;O;Hex;FA 16:1 | 501 | 102 | 2473 | 678 | 936 | 624 | 670 | 259 | 780 |
| ASG 28:2;O;Hex;FA 20:1 | 332 | 116 | 2117 | 557 | 378 | 298 | 616 | 261 | 584 |
| ASG 29:2;O;Hex;FA 20:5 | 506 | 666 | 2075 | 1148 | 1501 | 1178 | 1241 | 2010 | 1291 |
|  |  |  |  |  |  |  |  |  |  |
| Carnitines |  |  |  |  |  |  |  |  |  |
| CAR 13:0 | 214 | 1006 | 3962 | 1084 | 1309 | 1705 | 1436 | 509 | 1403 |
| CAR 21:2 | 1744 | 1472 | 12555 | 3304 | 4898 | 3469 | 4321 | 2044 | 4226 |
| CAR 21:4 | 297 | 1743 | 1921 | 2184 | 1469 | 2769 | 1427 | 997 | 1601 |
| CAR 4:0 | 1435 | 5953 | 12056 | 9699 | 10679 | 11883 | 10954 | 4246 | 8363 |
| CAR 5:0 | 819 | 3504 | 16500 | 15230 | 14823 | 11472 | 10280 | 6464 | 9887 |
| **Ceramides** |  |  |  |  |  |  |  |  |  |
| Cer 12:0;2O/25:0 | 2717 | 2828 | 24995 | 3476 | 7147 | 8322 | 5187 | 3355 | 7253 |
| Cer 12:2;2O/19:4 | 544 | 307 | 5905 | 758 | 849 | 864 | 2089 | 613 | 1491 |
| Cer 13:2;2O/30:3 | 3542 | 2907 | 4022 | 3680 | 3577 | 2746 | 1974 | 2847 | 3162 |
| Cer 20:2;4O|Cer 12:2;3O/8:0;(2OH) | 1738 | 4471 | 10016 | 2444 | 5933 | 4394 | 9151 | 2439 | 5073 |
| Cer 24:3;3O|Cer 15:3;2O/9:0;O | 884 | 1633 | 4398 | 1527 | 2129 | 1944 | 1844 | 1162 | 1940 |
| Cer 32:0;2O|Cer 16:0;2O/16:0 | 568 | 437 | 940 | 549 | 578 | 500 | 556 | 391 | 565 |
| Cer 32:1;2O|Cer 16:1;2O/16:0 | 420 | 329 | 985 | 455 | 420 | 586 | 585 | 530 | 539 |
| Cer 33:1;2O|Cer 17:1;2O/16:0 | 258 | 146 | 759 | 216 | 288 | 271 | 372 | 220 | 316 |
| Cer 33:1;4O|Cer 18:0;3O/15:1;(2OH) | 1249 | 2167 | 5569 | 1514 | 3084 | 2039 | 3469 | 1226 | 2540 |
| Cer 34:1;2O|Cer 18:1;2O/16:0 | 1983 | 1578 | 3903 | 2672 | 2595 | 2295 | 2371 | 3434 | 2604 |
| Cer 34:1;3O|Cer 19:0;2O/15:1;O | 2803 | 1849 | 4029 | 2037 | 1588 | 1843 | 1834 | 1779 | 2220 |
| Cer 34:3;2O|Cer 12:2;2O/22:1 | 739 | 846 | 4184 | 1742 | 3503 | 1668 | 1842 | 1631 | 2020 |
| Cer 36:0;3O|Cer 18:0;2O/18:0;O | 8651 | 11595 | 32554 | 11752 | 16065 | 13254 | 17377 | 8181 | 14929 |
| Cer 36:1;3O|Cer 19:0;2O/17:1;O | 527 | 477 | 933 | 423 | 440 | 458 | 461 | 289 | 501 |
|  |  |  |  |  |  |  |  |  |  |
| Cer 36:3;4O|Cer 19:2;3O/17:1;(2OH) | 670 | 885 | 2289 | 979 | 1048 | 1433 | 1193 | 610 | 1138 |
| Cer 37:0;2O|Cer 19:0;2O/18:0 | 435 | 852 | 1206 | 297 | 1112 | 658 | 1268 | 89 | 740 |
| Cer 38:0;4O | 494 | 726 | 2584 | 616 | 2055 | 1113 | 2064 | 476 | 1266 |
| Cer 39:0;3O|Cer 18:0;2O/21:0;O | 3356 | 4185 | 14014 | 3348 | 8345 | 6741 | 9212 | 2350 | 6444 |
| Cer 40:0;2O|Cer 16:0;2O/24:0 | 938 | 1095 | 1883 | 906 | 1415 | 913 | 853 | 484 | 1061 |
| Cer 40:1;2O|Cer 16:1;2O/24:0 | 1750 | 2153 | 7086 | 1836 | 2600 | 2294 | 3194 | 1584 | 2812 |
| Cer 41:1;2O|Cer 18:1;2O/23:0 | 980 | 1187 | 3101 | 1164 | 1750 | 1382 | 1696 | 916 | 1522 |
| Cer 42:1;2O|Cer 18:1;2O/24:0 | 1048 | 1429 | 4267 | 1256 | 2162 | 1731 | 1984 | 1747 | 1953 |
| Cer 44:2;4O|Cer 28:1;3O(FA 16:0) | 1029 | 704 | 3743 | 1837 | 1409 | 1398 | 1579 | 1635 | 1667 |
|  |  |  |  |  |  |  |  |  |  |
|  |  |  |  |  |  |  |  |  |  |
|  |  |  |  |  |  |  |  |  |  |
| **Diacylglycerols** |  |  |  |  |  |  |  |  |  |
| DG 16:0 | 603 | 779 | 5396 | 2259 | 3651 | 1618 | 1449 | 1362 | 2140 |
| DG 18:0 | 3189 | 2163 | 12642 | 8002 | 9225 | 5573 | 4727 | 5070 | 6324 |
| DG 20:0 | 15030 | 7424 | 48648 | 44970 | 34224 | 21303 | 13268 | 27213 | 26510 |
| DG 21:0 | 321 | 312 | 2197 | 737 | 1169 | 643 | 658 | 604 | 830 |
| DG 22:0 | 5903 | 3285 | 32856 | 22223 | 18524 | 10289 | 8743 | 14160 | 14498 |
| DG 22:0|DG 10:0_12:0 | 223 | 173 | 2980 | 801 | 846 | 599 | 1117 | 800 | 942 |
| DG 22:0|DG 8:0_14:0 | 328 | 267 | 3455 | 973 | 1221 | 864 | 1220 | 839 | 1146 |
| DG 22:1 | 3066 | 2208 | 23637 | 10646 | 14362 | 7037 | 5044 | 7995 | 9249 |
| DG 22:1|DG 8:0_14:1 | 3373 | 2157 | 21863 | 10945 | 11700 | 6974 | 4201 | 7975 | 8648 |
| DG 24:0 | 3535 | 2005 | 33111 | 8301 | 8692 | 7381 | 10952 | 6115 | 10012 |
| DG 24:0|DG 10:0_14:0 | 1429 | 933 | 14235 | 3354 | 3093 | 2683 | 4216 | 2899 | 4105 |
| DG 24:0|DG 8:0_16:0 | 1746 | 1019 | 13778 | 5020 | 4419 | 3425 | 3748 | 2889 | 4505 |
| DG 24:1 | 3706 | 2007 | 31676 | 12976 | 13387 | 7382 | 5881 | 8374 | 10674 |
| DG 24:1|DG 10:0_14:1 | 2065 | 1273 | 16420 | 7198 | 6721 | 4175 | 3041 | 5141 | 5754 |
| DG 24:2 | 270 | 245 | 1538 | 924 | 1169 | 488 | 680 | 653 | 746 |
| DG 24:3 | 137 | 134 | 966 | 454 | 619 | 380 | 365 | 434 | 436 |
| DG 25:0|DG 10:0_15:0 | 505 | 262 | 4567 | 732 | 734 | 894 | 1431 | 515 | 1205 |
| DG 26:0|DG 12:0_14:0 | 5292 | 3080 | 35854 | 9780 | 11110 | 7621 | 10938 | 9507 | 11648 |
| DG 26:1 | 2975 | 1687 | 29230 | 8816 | 9005 | 5984 | 5622 | 5938 | 8657 |
| DG 26:1|DG 8:0_18:1 | 2486 | 1245 | 24524 | 6488 | 6517 | 4451 | 3528 | 4616 | 6732 |
| DG 26:2 | 306 | 258 | 2105 | 824 | 1099 | 610 | 833 | 597 | 829 |
| DG 26:2|DG 8:0_18:2 | 248 | 211 | 1825 | 800 | 996 | 537 | 697 | 574 | 736 |
| DG 26:3 | 107 | 83 | 1053 | 244 | 474 | 260 | 271 | 344 | 354 |
| DG 27:0 | 572 | 456 | 5347 | 747 | 1533 | 1113 | 1799 | 507 | 1509 |
| DG 27:5 | 493 | 426 | 5523 | 710 | 968 | 1253 | 2157 | 555 | 1511 |
| DG 28:0|DG 12:0_16:0 | 12045 | 8913 | 80953 | 25313 | 29949 | 25232 | 27124 | 19505 | 28629 |
| DG 28:1 | 4063 | 3485 | 41288 | 11080 | 19209 | 12499 | 12793 | 8674 | 14136 |
| DG 28:1|DG 10:0_18:1 | 6657 | 3805 | 62699 | 19801 | 18848 | 14476 | 13198 | 14949 | 19304 |
| DG 28:2 | 1694 | 1542 | 15264 | 4662 | 5768 | 4089 | 4328 | 2552 | 4987 |
| DG 28:2|DG 14:1_14:1 | 2091 | 1131 | 15123 | 5786 | 5729 | 3535 | 4125 | 3988 | 5189 |
| DG 28:3 | 197 | 172 | 2183 | 656 | 767 | 544 | 701 | 620 | 730 |
| DG 28:3|DG 10:0_18:3 | 233 | 146 | 2005 | 676 | 754 | 654 | 702 | 587 | 720 |
| DG 30:0 | 391 | 418 | 2476 | 712 | 919 | 810 | 990 | 530 | 906 |
| DG 30:0|DG 14:0_16:0 | 53092 | 42943 | 246204 | 82550 | 130694 | 94637 | 92635 | 47958 | 98839 |
| DG 30:1|DG 16:0_14:1 | 17762 | 10858 | 125561 | 38644 | 65653 | 41007 | 27111 | 25678 | 44034 |
| DG 30:2 | 794 | 688 | 7929 | 1539 | 3091 | 2006 | 2712 | 900 | 2457 |
| DG 30:2|DG 12:0_18:2 | 1471 | 735 | 10899 | 3159 | 3869 | 2481 | 2969 | 2416 | 3500 |
| DG 30:2|DG 14:1_16:1 | 1265 | 853 | 10237 | 4246 | 3877 | 2436 | 2587 | 2277 | 3472 |
| DG 30:3|DG 12:0_18:3 | 436 | 289 | 4152 | 1031 | 1127 | 1043 | 972 | 930 | 1247 |
| DG 30:5 | 113 | 325 | 776 | 165 | 704 | 560 | 645 | 144 | 429 |
| DG 30:6 | 2682 | 2329 | 27906 | 3288 | 7216 | 5372 | 13426 | 2461 | 8085 |
| DG 30:7 | 334 | 315 | 3144 | 485 | 935 | 761 | 1388 | 327 | 961 |
| DG 31:7 | 18344 | 21157 | 132310 | 24832 | 61982 | 46340 | 78035 | 19093 | 50261 |
| DG 31:8 | 1007 | 932 | 9113 | 1512 | 3079 | 2142 | 5078 | 850 | 2964 |
| DG 32:1|DG 14:0_18:1 | 66172 | 57325 | 347869 | 117878 | 155537 | 128568 | 84553 | 67517 | 128177 |

| DG 32:2 | 3379 | 3550 | 20449 | 6778 | 14510 | 8508 | 8412 | 4645 | 8779 |
| --- | --- | --- | --- | --- | --- | --- | --- | --- | --- |
| DG 32:2|DG 14:1_18:1 | 10233 | 5149 | 54207 | 21678 | 23663 | 17187 | 14297 | 11980 | 19799 |
| DG 32:3 | 511 | 863 | 3640 | 1010 | 2410 | 1925 | 1747 | 886 | 1624 |
| DG 32:3|DG 14:0_18:3 | 1442 | 964 | 10416 | 3225 | 3492 | 3118 | 3969 | 2424 | 3631 |
| DG 32:6 | 3177 | 4538 | 30997 | 3901 | 10629 | 7791 | 18292 | 3072 | 10300 |
| DG 32:7 | 777 | 858 | 9436 | 1200 | 2915 | 1921 | 3297 | 620 | 2628 |
| DG 33:2 | 748 | 693 | 2999 | 979 | 1547 | 1125 | 1619 | 704 | 1302 |
| DG 33:3 | 452 | 430 | 2533 | 446 | 908 | 740 | 899 | 332 | 843 |
| DG 33:6 | 28081 | 55986 | 168497 | 35467 | 128211 | 75652 | 104490 | 28624 | 78126 |
| DG 33:8 | 3009 | 2250 | 25392 | 5146 | 9687 | 6111 | 12952 | 3069 | 8452 |
| DG 33:9 | 492 | 459 | 5395 | 863 | 1439 | 1438 | 3292 | 886 | 1783 |
| DG 34:0 | 563 | 680 | 4436 | 845 | 1445 | 1220 | 1097 | 534 | 1352 |
| DG 34:0|DG 16:0_18:0 | 5492 | 11428 | 43370 | 8318 | 16975 | 14335 | 13323 | 7847 | 15136 |
| DG 34:1|DG 16:0_18:1 | 96495 | 102997 | 511166 | 144851 | 272736 | 211217 | 140254 | 149289 | 203626 |
| DG 34:2 | 431 | 478 | 1060 | 362 | 660 | 496 | 619 | 436 | 568 |
| DG 34:2|DG 16:0_18:2 | 35775 | 23058 | 203218 | 67004 | 91922 | 69543 | 54337 | 45418 | 73784 |
| DG 34:3|DG 16:0_18:3 | 3349 | 2537 | 25318 | 8093 | 10168 | 10769 | 7484 | 6723 | 9305 |
| DG 34:6 | 4019 | 6868 | 30728 | 5224 | 14091 | 11206 | 22706 | 5241 | 12510 |
| DG 34:7 | 1690 | 2015 | 15956 | 2755 | 6283 | 3856 | 7282 | 1962 | 5225 |
| DG 35:2 | 677 | 573 | 3236 | 696 | 1387 | 854 | 1191 | 682 | 1162 |
| DG 35:3 | 364 | 368 | 1919 | 458 | 921 | 599 | 946 | 303 | 735 |
| DG 35:8 | 8688 | 11455 | 59514 | 12858 | 36871 | 21624 | 33075 | 10132 | 24277 |
| DG 35:9 | 1494 | 1999 | 27707 | 2546 | 6394 | 4603 | 13078 | 1660 | 7435 |
| DG 36:1|DG 18:0_18:1 | 3671 | 7794 | 78467 | 7896 | 17173 | 18864 | 14583 | 7815 | 19533 |
| DG 36:2|DG 18:1_18:1 | 21468 | 32187 | 319581 | 41007 | 86527 | 111111 | 49569 | 51704 | 89144 |
| DG 36:3|DG 18:1_18:2 | 10702 | 7828 | 96914 | 16917 | 26364 | 22630 | 17259 | 11456 | 26259 |
| DG 36:4|DG 18:1_18:3 | 1908 | 1525 | 18472 | 3135 | 5181 | 5487 | 4030 | 2926 | 5333 |
| DG 36:5|DG 16:0_20:5 | 403 | 332 | 2555 | 983 | 1226 | 1183 | 960 | 629 | 1034 |
| DG 36:5|DG 18:2_18:3 | 452 | 267 | 3326 | 1021 | 1375 | 1271 | 1324 | 809 | 1231 |
| DG 36:7 | 2211 | 4028 | 15021 | 3333 | 11932 | 6149 | 8640 | 2866 | 6772 |
| DG 36:8 | 273 | 346 | 1982 | 315 | 876 | 558 | 886 | 185 | 678 |
| DG 37:9 | 2880 | 3357 | 31561 | 5210 | 12020 | 9465 | 17919 | 4079 | 10811 |
| DG 38:5|DG 16:0_22:5 | 3794 | 2485 | 31982 | 6613 | 9703 | 10223 | 9159 | 6113 | 10009 |
| DG 38:6 | 228 | 218 | 1788 | 266 | 707 | 463 | 908 | 170 | 594 |
| DG 40:6|DG 18:1_22:5 | 989 | 934 | 7449 | 1526 | 2462 | 2029 | 2222 | 1235 | 2356 |
| DG 40:8 | 396 | 518 | 2190 | 420 | 1445 | 819 | 1268 | 304 | 920 |
| DG 41:10 | 486 | 1131 | 2003 | 1135 | 4159 | 2050 | 2376 | 947 | 1786 |
| DG 41:11 | 294 | 489 | 1988 | 417 | 1280 | 835 | 1177 | 332 | 852 |
| DG 41:5 | 1478 | 3076 | 7521 | 1966 | 7421 | 3683 | 4715 | 1978 | 3980 |
| DG 41:6 | 11579 | 40951 | 35257 | 10388 | 55019 | 33285 | 42970 | 14181 | 30454 |
| DG 43:11 | 432 | 1151 | 2884 | 825 | 2751 | 1886 | 1995 | 658 | 1573 |
| DG 43:3 | 498 | 713 | 2343 | 561 | 1790 | 1090 | 1217 | 482 | 1087 |
| DG 43:4 | 1360 | 2613 | 7612 | 1724 | 5741 | 3462 | 5308 | 1443 | 3658 |
| DG 43:6 | 10165 | 29079 | 28722 | 12555 | 39639 | 22307 | 35961 | 11588 | 23752 |
| DG 43:7 | 10683 | 34334 | 56023 | 13017 | 50158 | 29478 | 40362 | 16375 | 31304 |
| DG 44:7 | 809 | 546 | 2661 | 1176 | 1120 | 1174 | 1188 | 1200 | 1234 |

| DG 44:9 | 1163 | 584 | 1699 | 3203 | 1571 | 1476 | 819 | 3722 | 1779 |
| --- | --- | --- | --- | --- | --- | --- | --- | --- | --- |
| DG 46:6 | 4458 | 4289 | 4061 | 4173 | 3640 | 4061 | 3467 | 3562 | 3964 |
| DG 47:7 | 8982 | 25801 | 25316 | 13115 | 44669 | 21595 | 27487 | 9875 | 22105 |
| DG 47:8 | 4437 | 12483 | 18783 | 6523 | 22408 | 11223 | 15832 | 7355 | 12380 |
| DG 51:7 | 2216 | 3561 | 11055 | 3221 | 8265 | 5099 | 5654 | 2868 | 5242 |
| DG 51:8 | 1199 | 1597 | 6433 | 1869 | 3537 | 2311 | 3745 | 1441 | 2767 |
| **Free Fatty Acida** |  |  |  |  |  |  |  |  |  |
| FA 12:0 | 346 | 485 | 1210 | 612 | 1553 | 752 | 611 | 1547 | 889 |
| FA 14:0 | 4408 | 4225 | 6667 | 6043 | 5713 | 4300 | 5581 | 8743 | 5710 |
| FA 14:1 | 503 | 295 | 471 | 1639 | 1925 | 456 | 365 | 2477 | 1016 |
| FA 15:0 | 400 | 496 | 810 | 624 | 1218 | 594 | 713 | 866 | 715 |
| FA 15:4 | 1187 | 1897 | 3383 | 1190 | 6625 | 3119 | 4835 | 1922 | 3020 |
| FA 16:0 | 229588 | 283787 | 307622 | 259415 | 416309 | 291008 | 354480 | 328317 | 308816 |
| FA 16:0;(2OH) | 221 | 243 | 418 | 251 | 620 | 271 | 533 | 550 | 388 |
| FA 16:0;O | 1089 | 1563 | 1801 | 1625 | 4167 | 2033 | 2137 | 3893 | 2289 |
| FA 16:1 | 5005 | 2452 | 4959 | 14138 | 8998 | 5268 | 3515 | 23197 | 8442 |
| FA 16:1;O | 1125 | 1732 | 2133 | 1353 | 3459 | 2102 | 2202 | 2652 | 2095 |
| FA 17:0 | 666 | 797 | 1346 | 721 | 1889 | 908 | 1452 | 1075 | 1107 |
| FA 17:1 | 122 | 101 | 356 | 253 | 223 | 119 | 170 | 532 | 234 |
| FA 17:4;O | 110 | 194 | 122 | 100 | 207 | 253 | 235 | 262 | 186 |
| FA 18:0 | 241747 | 337649 | 382899 | 272841 | 498900 | 324024 | 430605 | 325665 | 351791 |
| FA 18:0;(2OH) | 1674 | 2224 | 2502 | 1835 | 3573 | 2295 | 3108 | 2591 | 2475 |
| FA 18:0;O | 1801 | 2270 | 5036 | 5551 | 14329 | 4023 | 5394 | 11593 | 6250 |
| FA 18:1 | 21420 | 18736 | 70927 | 49622 | 82093 | 43095 | 37439 | 87166 | 51312 |
| FA 18:1;2O | 279 | 245 | 588 | 462 | 1058 | 671 | 798 | 1182 | 660 |
| FA 18:1;O | 1378 | 1547 | 2454 | 3013 | 7825 | 2852 | 2940 | 8294 | 3788 |
| FA 18:2 | 1254 | 959 | 3297 | 3363 | 4282 | 2790 | 2852 | 5063 | 2983 |
| FA 18:2;O | 1180 | 1144 | 1517 | 1245 | 2468 | 2032 | 1612 | 3489 | 1836 |
| FA 18:3 | 1031 | 513 | 2419 | 3484 | 3190 | 2327 | 1539 | 8387 | 2861 |
| FA 18:3;4O | 373 | 538 | 849 | 469 | 1063 | 535 | 1031 | 680 | 692 |
| FA 18:3;O | 189 | 98 | 487 | 215 | 237 | 322 | 152 | 547 | 281 |
| FA 19:0 | 150 | 370 | 352 | 209 | 448 | 162 | 174 | 324 | 274 |
| FA 19:0;(2OH) | 786 | 662 | 529 | 684 | 530 | 691 | 756 | 773 | 676 |
| FA 19:1 | 82 | 72 | 241 | 169 | 336 | 169 | 87 | 249 | 176 |
| FA 19:1;2O | 215 | 346 | 598 | 348 | 1240 | 676 | 1087 | 498 | 626 |
| FA 19:1;O | 278 | 230 | 755 | 609 | 1294 | 309 | 180 | 2600 | 782 |
| FA 20:0 | 3135 | 4200 | 4513 | 3128 | 6308 | 3469 | 4850 | 2967 | 4071 |
| FA 20:1 | 224 | 173 | 332 | 369 | 446 | 440 | 401 | 707 | 386 |
| FA 20:3 | 144 | 87 | 467 | 253 | 382 | 404 | 419 | 614 | 346 |
| FA 20:4 | 1122 | 418 | 1478 | 1958 | 1660 | 6629 | 1724 | 3855 | 2356 |
| FA 20:5 | 360 | 421 | 678 | 431 | 575 | 1844 | 671 | 764 | 718 |
| FA 21:0 | 277 | 513 | 691 | 330 | 533 | 401 | 773 | 287 | 476 |
| FA 21:1;2O | 183 | 267 | 237 | 235 | 252 | 194 | 498 | 312 | 272 |
| FA 22:0 | 1250 | 1658 | 1792 | 1282 | 2008 | 1369 | 1775 | 1383 | 1565 |
| FA 22:1 | 60 | 123 | 197 | 115 | 341 | 214 | 236 | 178 | 183 |
| FA 22:5 | 534 | 377 | 2384 | 770 | 1150 | 1097 | 1161 | 2335 | 1226 |

| FA 22:6 | 693 | 645 | 1112 | 993 | 1154 | 747 | 1330 | 2395 | 1134 |
| --- | --- | --- | --- | --- | --- | --- | --- | --- | --- |
| FA 23:0 | 699 | 1111 | 1274 | 692 | 1581 | 705 | 1952 | 1044 | 1132 |
| FA 23:1 | 34 | 61 | 203 | 64 | 132 | 66 | 184 | 305 | 131 |
| FA 24:0 | 4944 | 7757 | 8675 | 4868 | 9888 | 5996 | 10407 | 6494 | 7379 |
| FA 25:0 | 1806 | 3125 | 3286 | 1610 | 4360 | 2548 | 4618 | 2138 | 2936 |
| FA 26:0 | 10293 | 14792 | 19257 | 9617 | 20676 | 12107 | 22485 | 11828 | 15132 |
| FA 26:1;O | 549 | 672 | 951 | 465 | 849 | 527 | 783 | 695 | 686 |
| FA 27:0 | 3520 | 4643 | 7337 | 3482 | 4135 | 2539 | 5611 | 3038 | 4288 |
| FA 27:1;O | 270 | 171 | 293 | 203 | 349 | 266 | 310 | 242 | 263 |
| FA 28:0 | 15531 | 16928 | 27390 | 11652 | 21713 | 13574 | 27626 | 12734 | 18393 |
| FA 29:0 | 3886 | 3634 | 6937 | 3363 | 3955 | 3198 | 6431 | 3341 | 4343 |
| FA 30:0 | 25823 | 14629 | 43961 | 20450 | 20266 | 14700 | 27009 | 14917 | 22719 |
| FA 31:0 | 2323 | 1874 | 4240 | 1654 | 2389 | 1872 | 4069 | 1789 | 2526 |
| FA 32:0 | 6534 | 4528 | 14052 | 4780 | 4660 | 4492 | 10273 | 4193 | 6689 |
| FA 33:0 | 666 | 454 | 1246 | 548 | 275 | 506 | 1198 | 439 | 667 |
| FA 34:0 | 1166 | 1039 | 2553 | 1017 | 1161 | 614 | 2012 | 756 | 1290 |
| FA 34:8 | 1085 | 226 | 2219 | 886 | 426 | 544 | 692 | 496 | 822 |
| FA 42:5 | 575580 | 722764 | 1268786 | 553471 | 1013630 | 541144 | 946879 | 388997 | 751406 |
| FA 42:6 | 519 | 650 | 842 | 492 | 968 | 639 | 1104 | 656 | 734 |
| FA 44:5 | 174508 | 222495 | 480905 | 156123 | 331628 | 177608 | 310429 | 103032 | 244591 |
| FA 44:6 | 364 | 427 | 796 | 428 | 428 | 394 | 523 | 347 | 463 |
| **Dihexosylceramides** |  |  |  |  |  |  |  |  |  |
| Hex2Cer 32:1;2O|Hex2Cer 16:1;2O/16:0 | 189 | 176 | 300 | 193 | 261 | 175 | 151 | 147 | 199 |
| Hex2Cer 33:1;2O|Hex2Cer 17:1;2O/16:0 | 155 | 178 | 283 | 165 | 252 | 169 | 160 | 151 | 189 |
| Hex2Cer 34:0;2O | 576 | 473 | 1418 | 704 | 737 | 685 | 644 | 550 | 724 |
| Hex2Cer 34:1;2O|Hex2Cer 18:1;2O/16:0 | 5093 | 3151 | 8157 | 6939 | 4813 | 4137 | 2615 | 5056 | 4995 |
| Hex2Cer 36:1;2O|Hex2Cer 18:1;2O/18:0 | 120 | 116 | 309 | 146 | 136 | 173 | 114 | 118 | 154 |
| Hex2Cer 38:1;2O|Hex2Cer 16:1;2O/22:0 | 657 | 651 | 1544 | 517 | 742 | 610 | 701 | 496 | 740 |
| Hex2Cer 39:0;2O|Hex2Cer 17:0;2O/22:0 | 975 | 1204 | 1110 | 493 | 762 | 706 | 550 | 376 | 772 |
| Hex2Cer 39:1;2O|Hex2Cer 16:1;2O/23:0 | 982 | 917 | 1578 | 589 | 1054 | 877 | 861 | 426 | 911 |
| Hex2Cer 40:1;2O|Hex2Cer 18:1;2O/22:0 | 1802 | 1994 | 3350 | 1567 | 2488 | 1931 | 2100 | 1818 | 2131 |
| Hex2Cer 40:2;2O|Hex2Cer 16:1;2O/24:1 | 118 | 122 | 190 | 101 | 92 | 90 | 60 | 78 | 106 |
| Hex2Cer 41:1;2O|Hex2Cer 18:1;2O/23:0 | 909 | 973 | 1981 | 792 | 1361 | 1069 | 862 | 1007 | 1119 |
| Hex2Cer 42:1;2O|Hex2Cer 18:1;2O/24:0 | 604 | 1241 | 1425 | 778 | 1277 | 1190 | 956 | 997 | 1058 |
| Hex2Cer 42:2;2O|Hex2Cer 18:1;2O/24:1 | 232 | 245 | 487 | 208 | 350 | 353 | 212 | 225 | 289 |
| HexCer 33:1;2O|HexCer 17:1;2O/16:0 | 480 | 406 | 1224 | 609 | 646 | 406 | 663 | 316 | 594 |
| HexCer 34:0;2O|HexCer 18:0;2O/16:0 | 356 | 295 | 485 | 372 | 397 | 139 | 226 | 236 | 313 |
| HexCer 34:1;2O | 1161 | 1152 | 1277 | 1918 | 1199 | 662 | 914 | 889 | 1147 |
| HexCer 34:1;2O|HexCer 18:1;2O/16:0 | 1509 | 1399 | 2070 | 2110 | 1864 | 856 | 1585 | 1434 | 1603 |
| HexCer 34:1;3O|HexCer 18:1;2O/16:0;O | 391 | 296 | 1076 | 1078 | 927 | 533 | 487 | 698 | 686 |
| HexCer 38:1;2O | 296 | 242 | 347 | 183 | 326 | 119 | 132 | 111 | 220 |
| HexCer 39:1;3O | 619 | 309 | 1320 | 1160 | 1299 | 543 | 556 | 901 | 838 |
| HexCer 40:0;3O | 268 | 63 | 332 | 550 | 101 | 101 | 24 | 302 | 218 |
| HexCer 40:1;2O | 2614 | 1255 | 4818 | 3438 | 1423 | 1086 | 1246 | 2166 | 2256 |
| HexCer 40:1;2O|HexCer 18:1;2O/22:0 | 815 | 991 | 1795 | 461 | 1291 | 488 | 682 | 612 | 892 |
| HexCer 40:1;3O | 1226 | 824 | 2556 | 3525 | 2356 | 1362 | 810 | 2155 | 1852 |

| HexCer 41:1;2O | 2225 | 1595 | 3245 | 2450 | 2065 | 1249 | 2114 | 1919 | 2108 |
| --- | --- | --- | --- | --- | --- | --- | --- | --- | --- |
| HexCer 41:1;2O|HexCer 18:1;2O/23:0 | 2163 | 3127 | 10169 | 3191 | 7782 | 3806 | 7038 | 3012 | 5036 |
| HexCer 41:1;3O | 1841 | 784 | 3866 | 6345 | 2953 | 1742 | 1236 | 4369 | 2892 |
| HexCer 42:1;2O | 4192 | 1825 | 8464 | 5747 | 3022 | 1428 | 2551 | 2896 | 3766 |
| HexCer 42:1;3O | 1310 | 705 | 2939 | 4047 | 3568 | 2372 | 825 | 4086 | 2482 |
| HexCer 43:1;3O | 427 | 81 | 974 | 997 | 265 | 305 | 149 | 716 | 489 |
| HexCer 49:5;4O | 2973 | 3355 | 4168 | 1907 | 1427 | 1447 | 1485 | 1061 | 2228 |
| HexCer 51:9;3O | 601 | 471 | 340 | 128 | 300 | 326 | 200 | 84 | 306 |
| HexCer 53:9;3O | 632 | 740 | 677 | 439 | 747 | 500 | 205 | 314 | 532 |
| **Lysophophatidylcholines** |  |  |  |  |  |  |  |  |  |
| LPC 14:0 | 601 | 1006 | 2268 | 1336 | 1699 | 1499 | 2177 | 2280 | 1608 |
| LPC 15:0 | 594 | 1100 | 1882 | 1390 | 1282 | 1221 | 1568 | 1317 | 1294 |
| LPC 16:0 | 4728 | 5767 | 16324 | 13526 | 10818 | 12267 | 11218 | 21441 | 12011 |
| LPC 17:0 | 197 | 234 | 799 | 393 | 402 | 406 | 553 | 414 | 425 |
| LPC 18:0 | 799 | 1098 | 2911 | 1858 | 1760 | 2360 | 2092 | 2318 | 1900 |
| LPC 18:1 | 2133 | 2481 | 8858 | 4782 | 4142 | 4003 | 3584 | 5854 | 4480 |
| LPC 18:2 | 504 | 1117 | 3140 | 1598 | 2173 | 1546 | 1904 | 1366 | 1669 |
| LPC 26:0 | 399 | 379 | 761 | 459 | 533 | 444 | 520 | 428 | 490 |
| LPC 28:7 | 191 | 286 | 923 | 495 | 783 | 461 | 574 | 171 | 485 |
| **Lysophosphatidylethanolamines** |  |  |  |  |  |  |  |  |  |
| LPE 16:0 | 1445 | 1194 | 2401 | 3145 | 2981 | 2278 | 1595 | 3501 | 2318 |
| LPE 16:1 | 314 | 184 | 298 | 838 | 523 | 389 | 197 | 545 | 411 |
| LPE 18:0 | 1215 | 1004 | 3294 | 2533 | 2021 | 1676 | 1533 | 3512 | 2098 |
| LPE 18:1 | 7004 | 5567 | 16246 | 14215 | 16148 | 12353 | 8626 | 11797 | 11494 |
| LPE 18:2 | 1493 | 1379 | 2248 | 3146 | 3067 | 1990 | 2403 | 2906 | 2329 |
| LPE 18:3 | 280 | 295 | 538 | 558 | 973 | 519 | 486 | 926 | 572 |
| LPE 20:4 | 255 | 288 | 348 | 407 | 330 | 239 | 193 | 410 | 309 |
| **Lysophosphatidylinositols** |  |  |  |  |  |  |  |  |  |
| LPI 18:0 | 3670 | 2198 | 4256 | 1624 | 1897 | 1722 | 2274 | 3176 | 2602 |
| LPI 18:1 | 2705 | 1364 | 2575 | 1457 | 1779 | 1788 | 1650 | 6006 | 2416 |
| LPI 18:2 | 375 | 264 | 556 | 217 | 456 | 386 | 504 | 408 | 396 |
| **Monoacylglycerols** |  |  |  |  |  |  |  |  |  |
| MG 15:0 | 143 | 845 | 3153 | 762 | 1039 | 1665 | 1168 | 403 | 1147 |
| MG 17:3 | 1305 | 5669 | 9892 | 1205 | 5445 | 2718 | 5650 | 1028 | 4114 |
| MG 17:4 | 1495 | 3817 | 11379 | 2666 | 6616 | 4773 | 7723 | 2167 | 5080 |
| MG 18:0 | 1487 | 3323 | 10086 | 2548 | 7667 | 4747 | 5487 | 1495 | 4605 |
| MG 19:3 | 433 | 1399 | 2780 | 536 | 1834 | 802 | 1611 | 329 | 1215 |
| MG 21:4 | 1234 | 925 | 10039 | 1790 | 2455 | 2420 | 2898 | 1331 | 2887 |
| MG 22:1 | 551 | 852 | 4382 | 933 | 1890 | 1408 | 1662 | 541 | 1527 |
| MG 24:2 | 388 | 423 | 1102 | 367 | 506 | 462 | 539 | 235 | 503 |
| MG 30:7 | 3616 | 6268 | 39414 | 8274 | 8270 | 11658 | 16712 | 6339 | 12569 |
| MG 32:8 | 634 | 598 | 4470 | 935 | 1383 | 1204 | 1826 | 714 | 1470 |
| MG 34:8 | 1447 | 1774 | 9451 | 2110 | 4617 | 3407 | 5328 | 1411 | 3693 |
| MG 36:6 | 717 | 373 | 4611 | 1145 | 1015 | 748 | 1346 | 448 | 1300 |
|  |  |  |  |  |  |  |  |  |  |
| **N-acyl ethanolamines** |  |  |  |  |  |  |  |  |  |

| NAE 12:0 | 1806 | 2975 | 8629 | 3072 | 4420 | 3557 | 5510 | 2126 | 4012 |
| --- | --- | --- | --- | --- | --- | --- | --- | --- | --- |
| NAE 13:1 | 1993 | 7206 | 21847 | 6379 | 18918 | 9680 | 19052 | 2900 | 10997 |
| NAE 14:1 | 6277 | 13341 | 37672 | 11340 | 24055 | 17134 | 21357 | 5237 | 17052 |
| NAE 15:1 | 1078 | 1725 | 4814 | 1704 | 2607 | 1916 | 2774 | 959 | 2197 |
| NAE 16:1 | 334962 | 544694 | 1152220 | 415465 | 604337 | 524570 | 685033 | 309652 | 571367 |
| NAE 16:2 | 742394 | 1237382 | 2734902 | 932770 | 1338825 | 1189687 | 1587999 | 753360 | 1314665 |
| NAE 16:3 | 264634 | 564471 | 1500262 | 526279 | 796901 | 605113 | 974071 | 329015 | 695093 |
| NAE 16:4 | 22422 | 46691 | 137850 | 37426 | 71985 | 45744 | 95966 | 27344 | 60678 |
| NAE 17:4 | 2333 | 5364 | 18617 | 4642 | 10652 | 8269 | 11768 | 2248 | 7987 |
| NAE 18:1 | 18541 | 21539 | 41053 | 17803 | 18797 | 19415 | 21958 | 15251 | 21795 |
| NAE 18:2 | 25200 | 33155 | 60952 | 24906 | 27186 | 28094 | 34229 | 21527 | 31906 |
| NAE 18:3 | 1689 | 3728 | 11453 | 3180 | 5434 | 4244 | 6391 | 1873 | 4749 |
| NAE 18:4 | 54643 | 117858 | 330746 | 86001 | 163021 | 133162 | 191729 | 45837 | 140375 |
| NAE 18:5 | 72676 | 170936 | 467885 | 137319 | 268255 | 190538 | 290773 | 72799 | 208898 |
| NAE 20:1 | 2175 | 2682 | 6914 | 2878 | 3617 | 3038 | 4139 | 1789 | 3404 |
| NAE 20:2 | 7408 | 7409 | 14804 | 6534 | 6356 | 9324 | 7663 | 5134 | 8079 |
| NAE 20:4 | 4874 | 6203 | 13597 | 4794 | 5690 | 6352 | 7632 | 3579 | 6590 |
| NAE 20:5 | 3285 | 4514 | 11330 | 3782 | 4883 | 5182 | 6303 | 2557 | 5230 |
| NAE 22:3 | 733 | 1295 | 2763 | 1396 | 2072 | 1795 | 1665 | 865 | 1573 |
| NAE 22:4 | 1251 | 1128 | 3433 | 1386 | 1060 | 1617 | 1296 | 1076 | 1531 |
| NAE 22:5 | 1124 | 1642 | 3527 | 1357 | 2022 | 2193 | 2131 | 881 | 1860 |
| NAE 24:4 | 589 | 1447 | 3742 | 1169 | 2157 | 1754 | 2592 | 489 | 1743 |
| NAE 5:0 | 773 | 4563 | 17197 | 3833 | 6925 | 6909 | 8536 | 2482 | 6402 |
| NAE 6:0 | 6605 | 36269 | 100793 | 52257 | 54305 | 66322 | 46107 | 10588 | 46656 |
| NAE 7:0 | 56 | 240 | 856 | 320 | 880 | 512 | 561 | 119 | 443 |
| **Phosphatidylcholines** |  |  |  |  |  |  |  |  |  |
| PC 24:0 | 154 | 117 | 779 | 493 | 481 | 498 | 521 | 499 | 443 |
| PC 26:0|PC 12:0_14:0 | 635 | 715 | 3001 | 2128 | 1849 | 1643 | 1930 | 2743 | 1831 |
| PC 27:0|PC 12:0_15:0 | 163 | 182 | 737 | 377 | 372 | 331 | 429 | 455 | 381 |
| PC 28:0 | 374 | 335 | 1266 | 728 | 746 | 680 | 647 | 659 | 679 |
| PC 28:0|PC 14:0_14:0 | 20704 | 15120 | 45509 | 40548 | 25894 | 28691 | 23410 | 46994 | 30859 |
| PC 28:1 | 196 | 217 | 825 | 449 | 589 | 292 | 373 | 506 | 431 |
| PC 29:0|PC 14:0_15:0 | 4268 | 2982 | 10405 | 6641 | 5259 | 6346 | 5257 | 8237 | 6174 |
| PC 30:0|PC 14:0_16:0 | 403758 | 286596 | 818698 | 643039 | 559586 | 528264 | 426905 | 586902 | 531718 |
| PC 30:1|PC 14:0_16:1 | 3904 | 2534 | 8414 | 9455 | 6577 | 4767 | 3381 | 8521 | 5944 |
| PC 30:3 | 322 | 367 | 1413 | 652 | 810 | 739 | 560 | 599 | 683 |
| PC 31:0|PC 15:0_16:0 | 17372 | 16162 | 42068 | 33983 | 40448 | 34423 | 28591 | 54376 | 33428 |
| PC 31:1 | 1419 | 904 | 4219 | 2092 | 2165 | 2459 | 1113 | 1942 | 2039 |
| PC 31:1|PC 15:0_16:1 | 1301 | 943 | 2782 | 2246 | 1888 | 1458 | 1393 | 1872 | 1735 |
| PC 32:0 | 7616 | 4314 | 12901 | 14328 | 14088 | 11211 | 8570 | 17058 | 11261 |
| PC 32:0|PC 16:0_16:0 | 320959 | 237848 | 855380 | 673999 | 715442 | 560233 | 536726 | 736771 | 579670 |
| PC 32:1 | 2294 | 1330 | 3012 | 4002 | 2652 | 2543 | 1445 | 4048 | 2666 |
| PC 32:1|PC 14:0_18:1 | 157133 | 96756 | 242853 | 280966 | 209334 | 160859 | 102681 | 229236 | 184977 |
| PC 32:2|PC 16:1_16:1 | 5190 | 3707 | 10263 | 9359 | 6082 | 6167 | 6141 | 6630 | 6692 |
| PC 32:3|PC 14:0_18:3 | 463 | 381 | 1285 | 706 | 738 | 817 | 755 | 980 | 766 |
| PC 32:3|PC 14:1_18:2 | 8765 | 4519 | 11928 | 15945 | 8234 | 9423 | 6140 | 15856 | 10101 |

| PC 33:0|PC 16:0_17:0 | 7588 | 7123 | 26393 | 11840 | 16145 | 16151 | 12542 | 13569 | 13919 |
| --- | --- | --- | --- | --- | --- | --- | --- | --- | --- |
| PC 33:1|PC 15:0_18:1 | 16996 | 13434 | 40868 | 27596 | 25519 | 25839 | 19985 | 32942 | 25397 |
| PC 33:2 | 1291 | 836 | 4633 | 1834 | 1724 | 2088 | 1865 | 1426 | 1962 |
| PC 34:0|PC 16:0_18:0 | 38888 | 33158 | 204401 | 73927 | 83749 | 85327 | 81780 | 138066 | 92412 |
| PC 34:1 | 22900 | 15698 | 51270 | 30180 | 26083 | 28342 | 18465 | 30321 | 27907 |
| PC 34:1|PC 16:0_18:1 | 758352 | 618631 | 2221089 | 1048349 | 1204497 | 1121708 | 909671 | 1087480 | 1121222 |
| PC 34:2 | 3900 | 3514 | 9394 | 4808 | 5129 | 5907 | 4368 | 4157 | 5147 |
| PC 34:2|PC 15:1_19:1 | 1048 | 945 | 3184 | 1491 | 1527 | 1359 | 1421 | 1225 | 1525 |
| PC 34:2|PC 16:0_18:2 | 136049 | 111882 | 233789 | 224331 | 226285 | 184522 | 148642 | 204483 | 183748 |
| PC 34:3|PC 16:0_18:3 | 20299 | 12570 | 49558 | 46414 | 35796 | 36419 | 22470 | 54201 | 34716 |
| PC 34:4|PC 17:2_17:2 | 258 | 208 | 780 | 612 | 663 | 446 | 474 | 680 | 515 |
| PC 35:0|PC 17:0_18:0 | 1130 | 1462 | 6999 | 2046 | 2749 | 2716 | 2577 | 2710 | 2799 |
| PC 35:1|PC 17:0_18:1 | 6025 | 6195 | 21324 | 6657 | 10741 | 9373 | 9053 | 12002 | 10171 |
| PC 35:2|PC 17:1_18:1 | 6350 | 3226 | 17013 | 7341 | 6145 | 5836 | 5057 | 6639 | 7201 |
| PC 35:3 | 912 | 648 | 3004 | 1547 | 1256 | 1331 | 1161 | 1330 | 1399 |
| PC 36:0|PC 18:0_18:0 | 1056 | 1481 | 9017 | 1646 | 2444 | 3210 | 3144 | 3282 | 3160 |
| PC 36:1 | 639 | 482 | 2270 | 1005 | 952 | 918 | 1232 | 1617 | 1139 |
| PC 36:1|PC 18:0_18:1 | 117472 | 90712 | 489275 | 178012 | 183352 | 156798 | 185450 | 300119 | 212649 |
| PC 36:2 | 1558 | 2177 | 4247 | 2062 | 2924 | 2665 | 2017 | 2691 | 2542 |
| PC 36:2|PC 18:1_18:1 | 116794 | 116241 | 270714 | 129251 | 159666 | 161063 | 102482 | 174027 | 153780 |
| PC 36:3 | 343 | 402 | 746 | 421 | 404 | 632 | 417 | 419 | 473 |
| PC 36:3|PC 18:1_18:2 | 61818 | 46664 | 148546 | 89620 | 82524 | 80049 | 86317 | 103690 | 87404 |
| PC 36:4 | 529 | 416 | 1672 | 938 | 825 | 842 | 707 | 1108 | 880 |
| PC 36:4|PC 18:2_18:2 | 18210 | 11711 | 68656 | 35990 | 25250 | 28433 | 26890 | 45010 | 32519 |
| PC 36:5|PC 16:0_20:5 | 3454 | 2466 | 8111 | 7108 | 5796 | 5235 | 4386 | 5753 | 5289 |
| PC 36:5|PC 18:2_18:3 | 998 | 899 | 2792 | 1989 | 1875 | 1829 | 1952 | 2276 | 1826 |
| PC 36:6 | 95 | 76 | 318 | 157 | 246 | 216 | 224 | 280 | 201 |
| PC 37:1|PC 19:0_18:1 | 423 | 537 | 1708 | 573 | 660 | 563 | 589 | 770 | 728 |
| PC 37:2|PC 18:1_19:1 | 1014 | 913 | 3388 | 976 | 982 | 1162 | 1116 | 1170 | 1340 |
| PC 38:1|PC 20:0_18:1 | 1225 | 1048 | 2494 | 1430 | 1422 | 1862 | 1338 | 1818 | 1580 |
| PC 38:2 | 879 | 794 | 2549 | 1235 | 1296 | 1240 | 1186 | 1371 | 1319 |
| PC 38:2|PC 20:0_18:2 | 2937 | 2429 | 4349 | 2525 | 2841 | 4285 | 2628 | 4825 | 3352 |
| PC 38:3 | 1276 | 1098 | 5032 | 1732 | 2137 | 2231 | 2522 | 2658 | 2336 |
| PC 38:3|PC 20:0_18:3 | 1105 | 575 | 2330 | 1210 | 1033 | 1769 | 1111 | 1833 | 1371 |
| PC 38:4 | 1433 | 1171 | 5767 | 2183 | 3453 | 2645 | 2974 | 3273 | 2863 |
| PC 38:4|PC 18:1_20:3 | 2882 | 1910 | 6818 | 3562 | 3183 | 3295 | 3770 | 4769 | 3774 |
| PC 38:5|PC 16:0_22:5 | 6532 | 4731 | 15474 | 10437 | 9629 | 10116 | 9481 | 18899 | 10662 |
| PC 38:6|PC 19:3_19:3 | 1419 | 893 | 4808 | 2738 | 2174 | 2374 | 2132 | 3104 | 2455 |
| PC 38:7 | 310 | 269 | 1012 | 514 | 621 | 508 | 576 | 598 | 551 |
| PC 40:3|PC 25:1_15:2 | 515 | 379 | 936 | 434 | 471 | 1092 | 660 | 1020 | 688 |
| PC 40:5|PC 18:0_22:5 | 681 | 603 | 2195 | 826 | 1107 | 1169 | 1665 | 1791 | 1255 |
| PC 40:6 | 1219 | 888 | 4749 | 1678 | 1857 | 1918 | 1742 | 3410 | 2183 |
| PC O-29:1 | 338 | 286 | 1113 | 574 | 497 | 546 | 576 | 523 | 557 |
| PC O-31:0 | 6406 | 5176 | 13877 | 11072 | 9415 | 8715 | 4642 | 8297 | 8450 |
| PC O-31:1 | 1525 | 1090 | 4015 | 2331 | 2355 | 2238 | 2107 | 3136 | 2350 |

| PC O-31:6 | 103 | 123 | 296 | 257 | 231 | 153 | 185 | 227 | 197 |
| --- | --- | --- | --- | --- | --- | --- | --- | --- | --- |
| PC O-33:2 | 1841 | 1333 | 5482 | 2250 | 2295 | 2683 | 4038 | 2298 | 2778 |
| PC O-33:3 | 1181 | 803 | 2476 | 1840 | 1719 | 1759 | 1753 | 1838 | 1671 |
| PC O-33:6 | 327 | 304 | 929 | 882 | 959 | 700 | 526 | 625 | 656 |
| PC O-35:6 | 753 | 613 | 1907 | 2237 | 2035 | 1203 | 1203 | 1770 | 1465 |
| PC O-35:9 | 1686 | 898 | 2064 | 2860 | 1677 | 1714 | 1196 | 2846 | 1868 |
| PC O-37:6 | 214 | 207 | 839 | 403 | 630 | 506 | 463 | 470 | 467 |
| PC O-38:10 | 908 | 789 | 3347 | 1181 | 1566 | 1255 | 1667 | 1548 | 1533 |
| PC O-39:0 | 663 | 1152 | 1263 | 852 | 1865 | 1103 | 900 | 1242 | 1130 |
| PC O-39:10 | 3213 | 2480 | 5689 | 3620 | 3812 | 3481 | 2643 | 4234 | 3647 |
| PC O-33:4 | 6176 | 2795 | 6164 | 14404 | 7565 | 6190 | 3830 | 18654 | 8222 |
| **Phosphatidylethanolamines** |  |  |  |  |  |  |  |  |  |
| PE 28:0|PE 14:0_14:0 | 1202 | 1168 | 1634 | 1542 | 1517 | 1387 | 1357 | 1355 | 1395 |
| PE 30:0|PE 14:0_16:0 | 3710 | 3863 | 6850 | 4737 | 5530 | 7507 | 5326 | 3124 | 5081 |
| PE 30:1|PE 12:0_18:1 | 1649 | 1573 | 2853 | 2890 | 2738 | 2158 | 1428 | 2572 | 2232 |
| PE 31:0|PE 15:0_16:0 | 587 | 473 | 1351 | 748 | 927 | 1193 | 747 | 543 | 821 |
| PE 31:1|PE 15:0_16:1 | 238 | 101 | 311 | 377 | 360 | 303 | 326 | 310 | 291 |
| PE 32:0|PE 16:0_16:0 | 6523 | 5916 | 11904 | 11467 | 12874 | 12312 | 10325 | 7455 | 9847 |
| PE 32:1|PE 14:0_18:1 | 22305 | 22745 | 37452 | 44662 | 56866 | 46442 | 29110 | 32500 | 36510 |
| PE 32:1|PE 16:0_16:1 | 6624 | 5064 | 9214 | 13239 | 11377 | 8300 | 5841 | 10068 | 8716 |
| PE 32:2 | 466 | 411 | 1301 | 777 | 856 | 634 | 705 | 565 | 714 |
| PE 32:2|PE 14:0_18:2 | 3352 | 3141 | 4212 | 5397 | 5325 | 4233 | 4501 | 4871 | 4379 |
| PE 32:3|PE 14:0_18:3 | 178 | 139 | 150 | 310 | 484 | 306 | 135 | 512 | 277 |
| PE 33:0|PE 16:0_17:0 | 812 | 597 | 1837 | 1146 | 1368 | 1720 | 966 | 785 | 1154 |
| PE 33:1|PE 15:0_18:1 | 5061 | 5259 | 10914 | 7753 | 10928 | 11525 | 7149 | 6860 | 8181 |
| PE 34:0|PE 16:0_18:0 | 3588 | 3209 | 11257 | 4753 | 6171 | 7385 | 5237 | 5153 | 5844 |
| PE 34:1 | 3642 | 3283 | 7562 | 5834 | 6939 | 5054 | 5622 | 4713 | 5331 |
| PE 34:1|PE 16:0_18:1 | 199672 | 198135 | 361120 | 308141 | 409085 | 349915 | 280235 | 268352 | 296832 |
| PE 34:2 | 1125 | 992 | 1924 | 1888 | 1992 | 1370 | 1597 | 1579 | 1558 |
| PE 34:2|PE 16:0_18:2 | 81871 | 76838 | 123116 | 138299 | 178894 | 139310 | 122664 | 122885 | 122985 |
| PE 34:3|PE 16:0_18:3 | 6830 | 7779 | 11100 | 12642 | 22100 | 18094 | 14187 | 19287 | 14002 |
| PE 34:3|PE 16:1_18:2 | 2496 | 1693 | 4037 | 6829 | 6924 | 4371 | 3306 | 4595 | 4281 |
| PE 34:4|PE 14:0_20:4 | 464 | 320 | 660 | 965 | 850 | 785 | 803 | 937 | 723 |
| PE 34:4|PE 16:1_18:3 | 299 | 225 | 266 | 1026 | 930 | 611 | 449 | 1067 | 609 |
| PE 35:0|PE 17:0_18:0 | 344 | 306 | 1123 | 319 | 384 | 561 | 313 | 210 | 445 |
| PE 35:1|PE 17:0_18:1 | 9354 | 9622 | 27191 | 12040 | 15261 | 19134 | 15848 | 12452 | 15113 |
| PE 35:2|PE 17:1_18:1 | 7697 | 7636 | 17969 | 11354 | 15844 | 13585 | 11671 | 11052 | 12101 |
| PE 35:3|PE 17:1_18:2 | 746 | 730 | 1822 | 1447 | 1792 | 1499 | 1269 | 1770 | 1384 |
| PE 36:0|PE 18:0_18:0 | 296 | 266 | 1344 | 799 | 399 | 758 | 623 | 899 | 673 |
| PE 36:1|PE 18:0_18:1 | 160562 | 143985 | 474992 | 200092 | 280718 | 269192 | 276299 | 251598 | 257180 |
| PE 36:2 | 1669 | 1304 | 6122 | 2997 | 3115 | 2916 | 2452 | 4023 | 3075 |
| PE 36:2|PE 18:1_18:1 | 441710 | 444512 | 1185790 | 636788 | 920999 | 917110 | 696837 | 637336 | 735135 |
| PE 36:3 | 1505 | 1273 | 4164 | 2328 | 2795 | 2637 | 2541 | 1841 | 2385 |
| PE 36:3;2O|PE 14:0_22:3;2O | 1291 | 892 | 2060 | 1077 | 1425 | 1522 | 1044 | 991 | 1288 |
| PE 36:3|PE 18:1_18:2 | 101556 | 106158 | 260586 | 167116 | 243063 | 234627 | 212515 | 172743 | 187295 |
| PE 36:4|PE 16:0_20:4 | 10903 | 9962 | 20989 | 20658 | 29397 | 22686 | 19989 | 21156 | 19467 |

| PE 36:4|PE 18:1_18:3 | 19481 | 19919 | 47977 | 40423 | 58324 | 56285 | 47903 | 57009 | 43415 |
| --- | --- | --- | --- | --- | --- | --- | --- | --- | --- |
| PE 36:5|PE 16:0_20:5 | 2478 | 2636 | 4311 | 4418 | 6549 | 5910 | 4290 | 5242 | 4479 |
| PE 36:5|PE 18:2_18:3 | 819 | 1040 | 1681 | 2165 | 2376 | 2310 | 2478 | 3543 | 2051 |
| PE 37:1|PE 18:0_19:1 | 1907 | 1703 | 4625 | 1588 | 1915 | 2096 | 2151 | 1856 | 2230 |
| PE 37:2|PE 18:1_19:1 | 3860 | 3273 | 10447 | 4322 | 6223 | 6366 | 4627 | 4325 | 5430 |
| PE 37:3|PE 18:1_19:2 | 834 | 685 | 1647 | 1125 | 1385 | 1434 | 1472 | 1278 | 1232 |
| PE 38:1|PE 20:0_18:1 | 2369 | 1912 | 7148 | 1827 | 2244 | 2942 | 2433 | 2080 | 2870 |
| PE 38:2|PE 18:1_20:1 | 4856 | 3787 | 10390 | 4867 | 6662 | 6901 | 4382 | 4754 | 5825 |
| PE 38:3|PE 18:0_20:3 | 7913 | 7259 | 19614 | 9754 | 12998 | 14771 | 15239 | 15271 | 12852 |
| PE 38:3|PE 18:1_20:2 | 1542 | 1493 | 4011 | 1886 | 2882 | 3334 | 2816 | 3037 | 2625 |
| PE 38:4|PE 18:0_20:4 | 9104 | 8477 | 27349 | 14851 | 20958 | 19936 | 18233 | 21782 | 17586 |
| PE 38:4|PE 18:1_20:3 | 7855 | 7748 | 19153 | 11793 | 15187 | 21901 | 14147 | 16405 | 14274 |
| PE 38:5|PE 16:0_22:5 | 15164 | 14642 | 33747 | 24558 | 33919 | 35048 | 31127 | 33467 | 27709 |
| PE 38:5|PE 18:1_20:4 | 4470 | 2583 | 9792 | 8560 | 5682 | 6513 | 5890 | 10922 | 6802 |
| PE 38:6 | 957 | 696 | 2770 | 2078 | 1776 | 1679 | 1481 | 1936 | 1672 |
| PE 39:1|PE 21:0_18:1 | 915 | 536 | 1407 | 551 | 350 | 797 | 600 | 457 | 702 |
| PE 39:2|PE 18:1_21:1 | 801 | 603 | 1636 | 821 | 1239 | 1178 | 496 | 717 | 936 |
| PE 40:1|PE 22:0_18:1 | 2586 | 1610 | 6523 | 1193 | 957 | 2385 | 1313 | 966 | 2192 |
| PE 40:2|PE 18:1_22:1 | 630 | 375 | 1066 | 555 | 689 | 765 | 455 | 483 | 627 |
| PE 40:4|PE 18:0_22:4 | 1124 | 720 | 3223 | 1199 | 1246 | 1495 | 1402 | 3391 | 1725 |
| PE 40:5|PE 18:0_22:5 | 8726 | 7657 | 23838 | 9805 | 14171 | 13231 | 18463 | 19475 | 14421 |
| PE 40:6|PE 18:1_22:5 | 3917 | 2521 | 9832 | 4170 | 4134 | 4012 | 3718 | 6350 | 4832 |
| PE 41:1|PE 23:0_18:1 | 541 | 278 | 1410 | 220 | 206 | 597 | 533 | 173 | 495 |
| PE 44:10|PE 22:5_22:5 | 2726 | 1450 | 6901 | 2851 | 4496 | 2989 | 3080 | 3733 | 3528 |
| PE O-19:0_18:1 | 893 | 520 | 2427 | 843 | 430 | 302 | 288 | 405 | 764 |
| PE O-30:1|PE O-14:1_16:0 | 369 | 361 | 621 | 286 | 196 | 414 | 565 | 327 | 393 |
| PE O-31:1|PE O-15:1_16:0 | 1181 | 1213 | 1888 | 1070 | 1187 | 2076 | 1691 | 1174 | 1435 |
| PE O-31:2|PE O-15:1_16:1 | 634 | 457 | 968 | 1215 | 1353 | 934 | 797 | 852 | 901 |
| PE O-32:1|PE O-16:1_16:0 | 345 | 323 | 633 | 440 | 199 | 463 | 475 | 293 | 396 |
| PE O-32:2|PE O-14:1_18:1 | 2778 | 2580 | 4264 | 4322 | 4619 | 4709 | 4132 | 4567 | 3997 |
| PE O-32:3|PE O-14:1_18:2 | 916 | 951 | 1784 | 1401 | 1522 | 1349 | 2037 | 1320 | 1410 |
| PE O-33:2|PE O-15:1_18:1 | 5771 | 4825 | 10277 | 7415 | 7573 | 9141 | 9650 | 8278 | 7866 |
| PE O-33:3|PE O-15:1_18:2 | 2603 | 2082 | 5668 | 3799 | 4533 | 4785 | 4524 | 3698 | 3962 |
| PE O-33:4|PE O-15:1_18:3 | 305 | 285 | 767 | 550 | 418 | 847 | 816 | 841 | 604 |
| PE O-34:2|PE O-16:1_18:1 | 18109 | 18072 | 35259 | 22971 | 28422 | 29494 | 22726 | 27877 | 25366 |
| PE O-34:3|PE O-16:1_18:2 | 8715 | 9225 | 18206 | 12231 | 14948 | 14372 | 14414 | 14898 | 13376 |
| PE O-34:4|PE O-16:1_18:3 | 1939 | 1625 | 3583 | 3281 | 3539 | 3671 | 2719 | 4553 | 3114 |
| PE O-35:1 | 3264 | 2415 | 9001 | 4571 | 6434 | 5858 | 4997 | 3149 | 4961 |
| PE O-35:2|PE O-17:1_18:1 | 2657 | 2341 | 5231 | 3604 | 3366 | 4304 | 2590 | 3975 | 3509 |
| PE O-35:3|PE O-17:1_18:2 | 1443 | 1444 | 3094 | 2035 | 2500 | 2297 | 2042 | 1795 | 2081 |
| PE O-35:3|PE O-17:2_18:1 | 509 | 455 | 914 | 651 | 331 | 796 | 627 | 619 | 613 |
| PE O-35:5|PE O-15:1_20:4 | 795 | 516 | 1352 | 1042 | 974 | 1050 | 1592 | 1004 | 1041 |
| PE O-35:6|PE O-15:1_20:5 | 283 | 156 | 398 | 306 | 256 | 306 | 481 | 325 | 314 |
| PE O-36:2|PE O-18:0_18:2 | 1205 | 1258 | 4181 | 602 | 589 | 5488 | 343 | 979 | 1831 |
| PE O-36:2|PE O-18:1_18:1 | 5879 | 4901 | 9830 | 7151 | 8676 | 7890 | 4838 | 6506 | 6959 |

| PE O-36:3|PE O-18:1_18:2 | 764 | 775 | 1322 | 490 | 451 | 1236 | 948 | 1364 | 919 |
| --- | --- | --- | --- | --- | --- | --- | --- | --- | --- |
| PE O-36:3|PE O-18:2_18:1 | 6530 | 5542 | 10959 | 7851 | 8953 | 8133 | 6411 | 8308 | 7836 |
| PE O-36:4|PE O-18:2_18:2 | 2865 | 2267 | 4421 | 3425 | 3650 | 5061 | 3915 | 4259 | 3733 |
| PE O-36:5|PE O-16:1_20:4 | 3029 | 2324 | 5391 | 2902 | 3114 | 3658 | 3865 | 4037 | 3540 |
| PE O-36:5|PE O-18:2_18:3 | 498 | 365 | 778 | 593 | 682 | 868 | 617 | 887 | 661 |
| PE O-36:6|PE O-16:1_20:5 | 1265 | 1029 | 1922 | 1234 | 1224 | 1955 | 1336 | 1572 | 1442 |
| PE O-38:4|PE O-18:1_20:3 | 698 | 623 | 877 | 869 | 740 | 893 | 532 | 826 | 757 |
| PE O-38:5|PE O-18:1_20:4 | 1457 | 997 | 3003 | 1481 | 1170 | 2014 | 1625 | 1858 | 1701 |
| PE O-38:6|PE O-16:1_22:5 | 2936 | 2100 | 4930 | 2207 | 2637 | 3234 | 4478 | 4072 | 3324 |
| PE O-40:6|PE O-18:1_22:5 | 1053 | 700 | 2067 | 763 | 650 | 1512 | 1210 | 1700 | 1207 |
| PE P-30:1|PE P-14:0_16:1 | 285 | 217 | 1160 | 486 | 508 | 380 | 500 | 309 | 480 |
| PE P-31:0|PE P-15:0_16:0 | 585 | 476 | 1708 | 402 | 735 | 707 | 964 | 475 | 756 |
| PE P-32:1|PE P-14:0_18:1 | 1085 | 1058 | 4440 | 1754 | 1949 | 1781 | 2066 | 1151 | 1911 |
| PE P-32:2|PE P-14:0_18:2 | 535 | 428 | 1597 | 692 | 761 | 831 | 791 | 581 | 777 |
| PE P-33:1|PE P-15:0_18:1 | 1058 | 844 | 2693 | 1308 | 1353 | 1859 | 2163 | 1471 | 1594 |
| PE P-33:2|PE P-15:0_18:2 | 494 | 462 | 1488 | 916 | 880 | 956 | 1107 | 701 | 875 |
| PE P-33:3|PE P-15:0_18:3 | 447 | 306 | 1515 | 789 | 578 | 677 | 600 | 800 | 714 |
| PE P-34:1|PE P-16:0_18:1 | 7055 | 4502 | 19337 | 8292 | 8332 | 7697 | 8229 | 8240 | 8960 |
| PE P-34:2|PE P-16:0_18:2 | 2083 | 1695 | 4939 | 2994 | 3166 | 2965 | 2544 | 2776 | 2895 |
| PE P-34:3|PE P-16:0_18:3 | 584 | 472 | 1280 | 960 | 1041 | 813 | 770 | 1177 | 887 |
| PE P-36:1|PE P-18:0_18:1 | 4494 | 4205 | 17661 | 5081 | 9523 | 6777 | 9559 | 3686 | 7623 |
| PE P-36:2|PE P-18:0_18:2 | 1463 | 1367 | 5564 | 2194 | 2903 | 2068 | 2916 | 1455 | 2491 |
| PE P-36:2|PE P-18:1_18:1 | 1383 | 1233 | 3921 | 2059 | 2393 | 1797 | 2237 | 1783 | 2101 |
| PE P-36:3|PE P-16:0_20:3 | 826 | 598 | 1853 | 1132 | 1100 | 1092 | 1175 | 1023 | 1100 |
| PE P-36:3|PE P-18:1_18:2 | 876 | 636 | 1710 | 1170 | 1345 | 1244 | 1183 | 1025 | 1149 |
| PE P-36:4|PE P-16:0_20:4 | 650 | 440 | 1608 | 592 | 577 | 862 | 879 | 772 | 797 |
| **Phosphatidylinositols** |  |  |  |  |  |  |  |  |  |
| PI 30:0 | 997 | 567 | 2124 | 1233 | 1224 | 782 | 697 | 1249 | 1109 |
| PI 30:0|PI 14:0_16:0 | 1091 | 640 | 2241 | 1478 | 1298 | 848 | 744 | 1334 | 1209 |
| PI 32:0|PI 16:0_16:0 | 7024 | 4916 | 8183 | 9097 | 8548 | 5969 | 6091 | 8524 | 7294 |
| PI 32:1|PI 14:0_18:1 | 3760 | 2103 | 5269 | 5980 | 6134 | 3996 | 3546 | 5346 | 4517 |
| PI 33:1 | 888 | 436 | 1513 | 1342 | 1608 | 709 | 900 | 1490 | 1111 |
| PI 34:0|PI 16:0_18:0 | 6425 | 3912 | 18172 | 7285 | 8871 | 6978 | 7021 | 9364 | 8504 |
| PI 34:1|PI 16:0_18:1 | 39176 | 24219 | 53910 | 45621 | 55272 | 38626 | 35633 | 47493 | 42494 |
| PI 34:2|PI 16:1_18:1 | 8054 | 4807 | 9194 | 11304 | 11646 | 6949 | 6828 | 10559 | 8668 |
| PI 34:3 | 410 | 210 | 498 | 641 | 637 | 417 | 335 | 1038 | 523 |
| PI 35:2 | 1222 | 654 | 1679 | 1376 | 1244 | 826 | 1147 | 1342 | 1186 |
| PI 36:0 | 1962 | 1406 | 6704 | 1866 | 2063 | 2293 | 1136 | 1491 | 2365 |
| PI 36:1|PI 18:0_18:1 | 131112 | 84326 | 311631 | 113908 | 156954 | 130666 | 106232 | 124997 | 144978 |
| PI 36:2 | 1165 | 583 | 2348 | 862 | 1539 | 1131 | 801 | 979 | 1176 |
| PI 36:2|PI 18:1_18:1 | 63012 | 42898 | 117629 | 58852 | 76239 | 59270 | 68431 | 69212 | 69443 |
| PI 36:3|PI 18:1_18:2 | 12994 | 7491 | 20119 | 14321 | 16646 | 10641 | 12843 | 18182 | 14155 |
| PI 36:4|PI 16:0_20:4 | 3451 | 1985 | 3766 | 4125 | 4498 | 3163 | 4514 | 3497 | 3625 |
| PI 36:4|PI 18:2_18:2 | 752 | 214 | 1018 | 1126 | 1309 | 898 | 757 | 2177 | 1031 |
| PI 36:5 | 1099 | 791 | 1343 | 1559 | 1343 | 1302 | 1320 | 1646 | 1300 |

| PI 37:1 | 2175 | 1024 | 3998 | 1473 | 1694 | 1065 | 1250 | 1472 | 1769 |
| --- | --- | --- | --- | --- | --- | --- | --- | --- | --- |
| PI 37:2 | 1699 | 962 | 2852 | 1361 | 1867 | 1092 | 1138 | 1495 | 1558 |
| PI 38:1 | 2502 | 1258 | 3658 | 1567 | 2519 | 1821 | 1045 | 2344 | 2089 |
| PI 38:2 | 6279 | 3637 | 10529 | 3626 | 5470 | 4803 | 3721 | 5249 | 5414 |
| PI 38:3|PI 18:0_20:3 | 15832 | 10565 | 38214 | 12098 | 16419 | 20124 | 19985 | 13297 | 18317 |
| PI 38:4|PI 18:0_20:4 | 18222 | 12385 | 50561 | 16863 | 24607 | 23627 | 26613 | 15740 | 23577 |
| PI 38:4|PI 18:1_20:3 | 5412 | 3564 | 7251 | 5391 | 6425 | 6500 | 5680 | 6793 | 5877 |
| PI 38:5|PI 18:0_20:5 | 9134 | 6369 | 17418 | 7996 | 9770 | 10819 | 8974 | 9401 | 9985 |
| PI 38:5|PI 18:1_20:4 | 7560 | 4401 | 12133 | 9341 | 11491 | 8961 | 7959 | 8333 | 8773 |
| PI 38:6 | 1479 | 553 | 1920 | 1986 | 1693 | 1754 | 1199 | 2206 | 1599 |
| PI 40:3 | 3369 | 4911 | 5339 | 2605 | 3475 | 5020 | 3621 | 1425 | 3721 |
| PI 40:6 | 402 | 157 | 819 | 521 | 290 | 365 | 189 | 718 | 433 |
| PI 42:10 | 2604 | 1525 | 5481 | 2710 | 3848 | 2675 | 3160 | 2393 | 3049 |
| **Phosphatidylserins** |  |  |  |  |  |  |  |  |  |
| PS 32:0 | 454 | 351 | 1006 | 562 | 643 | 627 | 623 | 387 | 582 |
| PS 32:1|PS 16:0_16:1 | 1801 | 1075 | 731 | 1622 | 1159 | 972 | 523 | 748 | 1079 |
| PS 34:1|PS 16:0_18:1 | 23492 | 19003 | 26855 | 17202 | 25180 | 24634 | 17818 | 16198 | 21298 |
| PS 34:2|PS 16:0_18:2 | 10442 | 6996 | 9553 | 8061 | 8851 | 7819 | 7242 | 6501 | 8183 |
| PS 34:3|PS 16:0_18:3 | 855 | 442 | 756 | 920 | 661 | 713 | 420 | 806 | 697 |
| PS 35:1|PS 18:0_17:1 | 6296 | 4549 | 11495 | 4637 | 5514 | 6418 | 4911 | 4984 | 6100 |
| PS 35:2|PS 17:0_18:2 | 410 | 275 | 1288 | 538 | 558 | 843 | 714 | 382 | 626 |
| PS 36:0|PS 18:0_18:0 | 37466 | 27000 | 78053 | 32024 | 37396 | 40164 | 39199 | 38053 | 41169 |
| PS 36:1|PS 18:0_18:1 | 240766 | 158299 | 470765 | 165810 | 228676 | 204417 | 196558 | 226646 | 236492 |
| PS 36:2|PS 18:1_18:1 | 128310 | 90260 | 244198 | 104149 | 137342 | 120492 | 124159 | 130000 | 134864 |
| PS 36:3|PS 18:0_18:3 | 2785 | 2007 | 8847 | 4435 | 5382 | 4325 | 4247 | 5967 | 4749 |
| PS 36:3|PS 18:1_18:2 | 18808 | 13166 | 34194 | 19543 | 25232 | 22911 | 21271 | 29171 | 23037 |
| PS 36:4|PS 18:1_18:3 | 2079 | 1344 | 3004 | 2334 | 2226 | 2121 | 2131 | 3523 | 2345 |
| PS 36:4|PS 18:2_18:2 | 279 | 249 | 577 | 360 | 522 | 362 | 417 | 443 | 401 |
| PS 37:0 | 79 | 72 | 250 | 97 | 146 | 110 | 116 | 104 | 122 |
| PS 37:2 | 198 | 191 | 555 | 185 | 311 | 262 | 253 | 229 | 273 |
| PS 38:1|PS 20:0_18:1 | 8831 | 5996 | 25042 | 5288 | 8366 | 9110 | 5787 | 5955 | 9297 |
| PS 38:2|PS 18:1_20:1 | 6394 | 4041 | 12746 | 4050 | 4377 | 5486 | 3968 | 4247 | 5664 |
| PS 38:3 | 35300 | 21021 | 19662 | 25506 | 20252 | 12394 | 13883 | 27804 | 21978 |
| PS 38:3|PS 18:0_20:3 | 19229 | 13464 | 36543 | 14875 | 18230 | 17979 | 18146 | 23142 | 20201 |
| PS 38:4|PS 18:1_20:3 | 3400 | 2198 | 5835 | 2089 | 2780 | 4259 | 3099 | 3807 | 3433 |
| PS 38:5|PS 16:0_22:5 | 3333 | 1814 | 2935 | 1805 | 1660 | 2131 | 2161 | 2331 | 2271 |
| PS 39:1|PS 21:0_18:1 | 4619 | 3502 | 14256 | 3888 | 5153 | 5235 | 4099 | 4951 | 5713 |
| PS 39:2|PS 18:1_21:1 | 3231 | 2243 | 6773 | 3169 | 4805 | 3491 | 2802 | 3596 | 3764 |
| PS 40:2 | 3064 | 2947 | 8463 | 2159 | 3219 | 2977 | 2327 | 3092 | 3531 |
| PS 40:2|PS 22:0_18:2 | 4021 | 3051 | 10789 | 2577 | 3591 | 3783 | 2661 | 3527 | 4250 |
| PS 40:3 | 3128 | 1430 | 3955 | 2812 | 1248 | 1030 | 911 | 2528 | 2130 |
| PS 40:4|PS 18:0_22:4 | 4228 | 2657 | 11946 | 4188 | 5244 | 3599 | 3810 | 8241 | 5489 |
| PS 40:5|PS 18:0_22:5 | 32164 | 21059 | 77937 | 30340 | 39020 | 29828 | 37717 | 54537 | 40325 |
| PS 40:6|PS 18:1_22:5 | 4452 | 2971 | 8811 | 4646 | 5743 | 3684 | 6315 | 8806 | 5678 |
| PS 41:2 | 1949 | 949 | 4079 | 1451 | 1246 | 1478 | 543 | 1838 | 1692 |
| PS 42:1|PS 24:0_18:1 | 2112 | 1076 | 5967 | 1007 | 1356 | 1420 | 1027 | 1306 | 1909 |
| **Sulfatides** |  |  |  |  |  |  |  |  |  |
|  |  |  |  |  |  |  |  |  |  |

| SHexCer 35:0;3O | 2285 | 2127 | 5823 | 2074 | 4973 | 3945 | 4360 | 5736 | 3915 |
| --- | --- | --- | --- | --- | --- | --- | --- | --- | --- |
| SHexCer 36:4;2O | 1435 | 2482 | 5053 | 2231 | 6558 | 4103 | 5054 | 3090 | 3751 |
| SHexCer 38:2;2O | 284 | 253 | 322 | 291 | 264 | 295 | 362 | 389 | 307 |
| SHexCer 38:7;3O | 395 | 487 | 1024 | 412 | 908 | 618 | 684 | 957 | 686 |
| SHexCer 39:5;3O | 875 | 1217 | 2381 | 945 | 2152 | 1509 | 2060 | 2021 | 1645 |
| SHexCer 39:6;3O | 479 | 597 | 1255 | 441 | 1146 | 579 | 572 | 1253 | 790 |
| SHexCer 39:7;3O | 3027 | 5227 | 7948 | 6432 | 13981 | 7603 | 9236 | 8253 | 7713 |
| SHexCer 39:8;3O | 1557 | 1948 | 2664 | 3203 | 5206 | 2790 | 3236 | 3798 | 3050 |
| SHexCer 40:2;2O | 1649 | 2505 | 5770 | 2265 | 4951 | 3161 | 3070 | 4521 | 3486 |
| SHexCer 40:3;3O | 546 | 745 | 4101 | 1575 | 3197 | 2631 | 2488 | 3840 | 2390 |
| SHexCer 40:8;3O | 267 | 392 | 1096 | 294 | 672 | 473 | 920 | 962 | 635 |
| SHexCer 40:9;3O | 794 | 1252 | 2076 | 634 | 2769 | 1469 | 2043 | 1954 | 1624 |
| SHexCer 41:8;3O | 8434 | 16304 | 32229 | 14686 | 39566 | 24020 | 28372 | 19610 | 22903 |
| SHexCer 42:2;2O | 181 | 167 | 368 | 211 | 196 | 171 | 368 | 406 | 259 |
| SHexCer 42:4;3O | 1699 | 2338 | 9785 | 3492 | 6295 | 4031 | 7287 | 9721 | 5581 |
| SHexCer 43:0;3O | 1070 | 706 | 1572 | 2323 | 774 | 451 | 778 | 2709 | 1298 |
| SHexCer 44:2;2O | 1195 | 1541 | 3656 | 1314 | 3079 | 1719 | 2261 | 1730 | 2062 |
| SHexCer 46:2;2O | 4805 | 7559 | 14073 | 6895 | 14352 | 9176 | 9217 | 10966 | 9630 |
| **Sphingomyelins** |  |  |  |  |  |  |  |  |  |
|  |  |  |  |  |  |  |  |  |  |
| SM 30:0;2O|SM 22:0;2O/8:0 | 970 | 975 | 1131 | 795 | 918 | 840 | 869 | 648 | 893 |
| SM 30:1;2O|SM 16:1;2O/14:0 | 803 | 754 | 1801 | 739 | 870 | 823 | 1450 | 1065 | 1038 |
| SM 31:0;2O|SM 19:0;2O/12:0 | 238 | 272 | 528 | 265 | 264 | 273 | 263 | 251 | 294 |
| SM 31:1;2O|SM 17:1;2O/14:0 | 871 | 733 | 1847 | 864 | 979 | 724 | 995 | 985 | 1000 |
| SM 32:0;2O | 997 | 809 | 1289 | 1251 | 1058 | 934 | 746 | 1185 | 1034 |
| SM 32:0;2O|SM 22:0;2O/10:0 | 35736 | 25290 | 40200 | 45952 | 34933 | 30561 | 21014 | 43556 | 34655 |
| SM 32:1;2O | 180 | 248 | 523 | 486 | 730 | 403 | 347 | 970 | 486 |
| SM 32:1;2O|SM 16:1;2O/16:0 | 51321 | 36913 | 92761 | 77401 | 61175 | 53462 | 55959 | 87208 | 64525 |
| SM 33:0;2O | 3980 | 2698 | 6052 | 5660 | 4525 | 3456 | 2378 | 6077 | 4353 |
| SM 33:1;2O | 653 | 526 | 1097 | 1034 | 891 | 782 | 750 | 1289 | 878 |
| SM 33:1;2O|SM 17:1;2O/16:0 | 29585 | 19324 | 57637 | 49854 | 38032 | 33102 | 29860 | 62375 | 39971 |
| SM 34:0;2O | 34510 | 25897 | 41623 | 71733 | 53517 | 32807 | 17199 | 76703 | 44249 |
| SM 34:1;2O | 16571 | 7875 | 16393 | 41557 | 19602 | 16506 | 9637 | 59337 | 23435 |
| SM 34:1;2O|SM 18:1;2O/16:0 | 315728 | 233139 | 627091 | 687147 | 581907 | 439893 | 361278 | 855957 | 512767 |
| SM 34:1;3O | 387 | 289 | 1116 | 1288 | 1141 | 846 | 569 | 1935 | 946 |
| SM 34:2;2O|SM 25:2;2O/9:0 | 2334 | 1797 | 5340 | 5270 | 3716 | 3677 | 3275 | 7617 | 4128 |
| SM 35:0;2O | 655 | 381 | 1449 | 976 | 817 | 799 | 614 | 1049 | 842 |
| SM 35:1;2O|SM 16:1;2O/19:0 | 6812 | 4079 | 15604 | 11759 | 7643 | 7510 | 5532 | 13495 | 9054 |
| SM 35:1;2O|SM 18:1;2O/17:0 | 2893 | 2301 | 8589 | 4493 | 4926 | 3528 | 4326 | 5419 | 4559 |
| SM 35:2;2O | 422 | 511 | 1275 | 629 | 793 | 605 | 647 | 474 | 670 |
| SM 35:2;2O|SM 21:1;2O/14:1 | 292 | 307 | 1125 | 442 | 625 | 421 | 440 | 499 | 519 |
| SM 35:2;3O | 1060 | 927 | 3202 | 1771 | 1708 | 1333 | 1541 | 1275 | 1602 |
| SM 36:0;2O | 1830 | 1622 | 6266 | 2061 | 2751 | 1896 | 1807 | 1823 | 2507 |
| SM 36:1;2O|SM 15:0;2O/21:1 | 11227 | 6778 | 26366 | 17567 | 14495 | 12380 | 11632 | 22412 | 15357 |
| SM 36:2;2O|SM 19:1;2O/17:1 | 2544 | 1440 | 6160 | 4392 | 2998 | 2725 | 2633 | 5512 | 3550 |
| SM 37:0;2O | 1352 | 1425 | 3325 | 1533 | 1870 | 1358 | 1356 | 1239 | 1682 |

| SM 37:1;2O | 603 | 605 | 2269 | 653 | 1003 | 901 | 861 | 893 | 974 |
| --- | --- | --- | --- | --- | --- | --- | --- | --- | --- |
| SM 38:0;2O|SM 16:0;2O/22:0 | 13517 | 13641 | 22413 | 13471 | 18633 | 13278 | 12708 | 6548 | 14276 |
| SM 38:1;2O|SM 16:1;2O/22:0 | 89648 | 72386 | 209020 | 97243 | 113336 | 86343 | 111968 | 99127 | 109884 |
| SM 38:2;2O|SM 19:2;2O/19:0 | 513 | 555 | 1535 | 876 | 1148 | 989 | 1034 | 747 | 925 |
| SM 39:0;2O | 37149 | 46405 | 50023 | 31657 | 43631 | 29728 | 30354 | 28001 | 37119 |
| SM 39:1;2O|SM 17:1;2O/22:0 | 1858 | 1580 | 3267 | 1731 | 1639 | 2142 | 1270 | 1939 | 1928 |
| SM 39:1;3O | 4315 | 5270 | 9475 | 5123 | 9170 | 6109 | 7641 | 8835 | 6992 |
| SM 39:2;2O|SM 19:1;2O/20:1 | 995 | 1130 | 2752 | 1611 | 1892 | 1641 | 1697 | 1588 | 1663 |
| SM 40:0;2O | 10291 | 16969 | 17239 | 10419 | 22680 | 11208 | 9506 | 9986 | 13537 |
| SM 40:1;2O | 2968 | 3182 | 4759 | 3830 | 4921 | 3815 | 3508 | 6930 | 4239 |
| SM 40:1;2O|SM 16:1;2O/24:0 | 165419 | 175372 | 332803 | 204799 | 308495 | 229216 | 231414 | 290708 | 242278 |
| SM 40:1;2O|SM 18:1;2O/22:0 | 2520 | 2308 | 5560 | 2509 | 3277 | 2995 | 1547 | 2038 | 2844 |
| SM 40:1;3O | 412 | 477 | 1656 | 1272 | 1839 | 1312 | 588 | 1278 | 1104 |
| SM 40:2;2O|SM 18:1;2O/22:1 | 12567 | 9172 | 24129 | 14111 | 13263 | 31371 | 13800 | 13954 | 16546 |
| SM 40:8;2O | 381 | 659 | 2282 | 544 | 1758 | 795 | 2011 | 510 | 1117 |
| SM 41:0;2O | 2837 | 4660 | 5133 | 4446 | 8365 | 3853 | 3866 | 5883 | 4880 |
| SM 41:1;2O|SM 18:1;2O/23:0 | 17244 | 30099 | 44305 | 26691 | 64624 | 32292 | 37429 | 41581 | 36783 |
| SM 41:2;2O|SM 18:1;2O/23:1 | 3535 | 4352 | 7718 | 5627 | 8434 | 6424 | 5754 | 9720 | 6446 |
| SM 42:1;2O|SM 18:1;2O/24:0 | 27458 | 58373 | 61820 | 64100 | 108183 | 81199 | 60791 | 132304 | 74278 |
| SM 42:2;2O | 259 | 321 | 356 | 390 | 525 | 476 | 329 | 709 | 421 |
| SM 42:2;2O|SM 18:1;2O/24:1 | 9593 | 10733 | 17914 | 16629 | 20116 | 18124 | 13711 | 27821 | 16830 |
| SM 42:2;3O | 164 | 135 | 510 | 273 | 364 | 269 | 391 | 364 | 309 |
| SM 42:3;2O | 420 | 414 | 1102 | 822 | 856 | 870 | 748 | 721 | 744 |
| SM 43:2;2O|SM 18:1;2O/25:1 | 783 | 1235 | 1348 | 1188 | 2333 | 1314 | 785 | 1886 | 1359 |
| SM 44:8;2O | 267 | 535 | 494 | 166 | 641 | 157 | 330 | 161 | 344 |
| **Triacylglycerols** |  |  |  |  |  |  |  |  |  |
| TG 46:0|TG 14:0_16:0_16:0 | 26836 | 15897 | 12141 | 11043 | 25377 | 12625 | 11794 | 19274 | 16873 |
| TG 48:1|TG 16:0_16:0_16:1 | 77888 | 63033 | 85788 | 59184 | 85649 | 80382 | 50173 | 96767 | 74858 |
| TG 49:3|TG 15:0_16:0_18:3 | 25207 | 9933 | 21392 | 20587 | 19449 | 24844 | 13766 | 41733 | 22114 |
| TG 50:2|TG 16:0_16:0_18:2 | 58869 | 35719 | 82987 | 34630 | 48932 | 52616 | 42789 | 67434 | 52997 |
| TG 51:2|TG 15:0_18:1_18:1 | 14330 | 7224 | 15410 | 5579 | 11578 | 14429 | 8944 | 16965 | 11807 |
| TG 51:4|TG 15:0_16:0_20:4 | 11621 | 7060 | 12702 | 9919 | 9786 | 19412 | 12030 | 34232 | 14595 |
| TG 52:2|TG 16:0_18:1_18:1 | 54517 | 43882 | 70769 | 28893 | 53195 | 79342 | 31833 | 72486 | 54365 |
| TG 52:3|TG 16:0_18:1_18:2 | 1418857 | 576936 | 1325880 | 710517 | 1295824 | 1388101 | 837574 | 1893982 | 1180959 |
| TG 53:4|TG 17:1_18:1_18:2 | 19415 | 5274 | 42231 | 9439 | 6218 | 11574 | 9352 | 15168 | 14834 |
| TG 54:6|TG 18:1_18:2_18:3 | 26411 | 11363 | 35633 | 15011 | 14071 | 28095 | 22730 | 48388 | 25213 |
| TG 56:6|TG 16:0_18:1_22:5 | 26963 | 7928 | 55819 | 14415 | 12489 | 23426 | 16350 | 26651 | 23005 |
| TG 57:6|TG 17:0_18:1_22:5 | 1204 | 469 | 2531 | 562 | 846 | 1517 | 804 | 1531 | 1183 |
| TG 10:0_10:0_20:5 | 26129 | 32653 | 161521 | 45174 | 109364 | 82631 | 91055 | 38295 | 73353 |
| TG 8:0_8:0_16:4 | 491 | 460 | 3878 | 881 | 1939 | 1496 | 2047 | 646 | 1480 |
| TG O-42:5|TG O-20:5_11:0_11:0 | 1163 | 1261 | 987 | 586 | 1713 | 1568 | 974 | 1716 | 1246 |
| TG O-49:6|TG O-15:4_17:1_17:1 | 3910 | 2863 | 2269 | 1995 | 3792 | 2534 | 2185 | 3928 | 2934 |
| TG O-51:6|TG O-15:4_17:0_19:2 | 2542 | 1586 | 960 | 1151 | 1929 | 1380 | 1529 | 2156 | 1654 |
| TG O-51:7|TG O-15:4_15:0_21:3 | 8305 | 7246 | 9915 | 6658 | 10235 | 8941 | 6358 | 10341 | 8500 |
| TG O-52:7|TG O-18:4_16:0_18:3 | 1638 | 1154 | 1472 | 873 | 1900 | 1676 | 1216 | 2248 | 1522 |

| TG O-54:8|TG O-15:4_18:1_21:3 | 1496 | 1116 | 1796 |  | 724 |  | 1952 | 2158 | 1190 | 2312 | 1593 |
| --- | --- | --- | --- | --- | --- | --- | --- | --- | --- | --- | --- |
| TG O-55:9|TG O-13:1_21:4_21:4 | 2939 | 2016 | 2644 |  | 1547 |  | 3985 | 3474 | 2296 | 5248 | 3019 |
| TG O-57:10|TG O-15:0_21:5_21:5 | 1425 | 1087 | 2036 |  | 649 |  | 1972 | 2354 | 1559 | 2813 | 1737 |
| TG O-57:9|TG O-15:1_21:4_21:4 | 1939 | 1057 | 2343 |  | 857 |  | 2154 | 2524 | 1183 | 2432 | 1811 |
| **Others** |  |  |  |  |  |  |  |  |  |  |  |
| CoQ8 | 1087 | 837 | 790 |  | 2089 |  | 2007 | 1181 | 523 | 2539 | 1382 |
| DGCC 17:2_22:6 | 1535 | 1351 | 4818 |  | 2157 |  | 2167 | 2004 | 2253 | 5077 | 2670 |
| DGGA 36:2|DGGA 18:1_18:1 | 689 | 1149 | 2802 |  | 1994 |  | 4380 | 2693 | 3087 | 3695 | 2561 |
| DGTS 21:0 | 284 | 275 | 1424 |  | 371 |  | 643 | 466 | 681 | 235 | 547 |
| LPA 16:3 | 1428 | 1494 | 2444 |  | 1286 |  | 2250 | 2372 | 2358 | 1228 | 1858 |
| LPE O-19:4 | 369 | 636 | 2324 |  | 584 |  | 1725 | 1057 | 1233 | 401 | 1041 |
| LPE-N (FA)33:2|LPE-N (FA 15:0)18:2 | 830 | 848 | 1019 |  | 1339 |  | 1663 | 1467 | 1340 | 1289 | 1224 |
| LPG O-13:0 | 471 | 618 | 824 |  | 491 |  | 749 | 769 | 611 | 317 | 606 |
| NAGly 9:0;O | 432 | 755 | 1771 |  | 932 |  | 1902 | 1668 | 2142 | 437 | 1255 |
| Pentaerythritol tetrakis(3,5-di-tert-butyl-4-hydro | 5641 | 10304 | 20252 |  | 6684 |  | 15657 | 14402 | 14211 | 3357 | 11313 |
| PI O-16:0 | 2085 | 1648 | 1509 |  | 1315 |  | 1325 | 1257 | 2250 | 3968 | 1920 |
| PI-Cer 38:3;2O | 572 | 343 | 1267 |  | 754 |  | 692 | 752 | 858 | 802 | 755 |
| PMeOH 40:4|PMeOH 20:2_20:2 | 412 | 94 | 1469 |  | 505 |  | 188 | 292 | 541 | 96 | 450 |
| SL 12:0;O/26:0 | 4496 | 4323 | 9326 |  | 5301 |  | 6965 | 4933 | 4317 | 4338 | 5500 |
| SL 12:1;O/32:0 | 3724 | 4002 | 11482 |  | 4289 |  | 6525 | 6274 | 8114 | 3203 | 5952 |
| ST 27:1;O;S - Cholesterol sulfate | 665 | 787 | 1382 |  | 1394 |  | 1978 | 1878 | 1163 | 2349 | 1449 |
| **Control T8 Metabolyte species** | **t8_C1** | **t8_C2** | **t8_C3** | **t8_C4** |  | **t8_C5** |  | **t8_C6** | **t8_C7** | **t8_C8** | **Average value** |
| **Acyl sterol glycoside** |  |  |  |  |  |  |  |  |  |  |  |
| ASG 27:1;O;Hex;FA 2:0 | 404 | 583 | 589 |  | 671 |  | 820 | 727 | 407 | 208 | 551 |
| ASG 28:1;O;Hex;FA 14:0 | 966 | 224 | 446 |  | 4500 |  | 3462 | 612 | 999 | 1058 | 1533 |
| ASG 28:1;O;Hex;FA 16:0 | 1106 | 90 | 196 |  | 4171 |  | 2664 | 530 | 797 | 996 | 1319 |
| ASG 28:1;O;Hex;FA 18:0 | 1059 | 132 | 321 |  | 3712 |  | 2831 | 417 | 568 | 857 | 1237 |
| ASG 28:1;O;Hex;FA 20:1 | 1139 | 112 | 286 |  | 3413 |  | 2472 | 440 | 624 | 926 | 1177 |
| ASG 28:2;O;Hex;FA 14:0 | 2338 | 303 | 437 |  | 9241 |  | 5522 | 1220 | 1596 | 2053 | 2839 |
| ASG 28:2;O;Hex;FA 16:0 | 1216 | 174 | 431 |  | 4975 |  | 3419 | 713 | 857 | 898 | 1585 |
| ASG 28:2;O;Hex;FA 16:1 | 772 | 64 | 167 |  | 2906 |  | 2424 | 251 | 379 | 349 | 914 |
| ASG 28:2;O;Hex;FA 20:1 | 476 | 151 | 164 |  | 2079 |  | 1760 | 218 | 298 | 476 | 703 |
| ASG 29:2;O;Hex;FA 20:5 | 647 | 450 | 1285 |  | 2311 |  | 1459 | 614 | 650 | 1566 | 1123 |
| **Carnitines** |  |  |  |  |  |  |  |  |  |  |  |
| CAR 13:0 | 3172 | 1556 | 1684 |  | 1318 |  | 2557 | 988 | 691 | 1884 | 1731 |
| CAR 21:2 | 4956 | 1617 | 2295 |  | 11151 |  | 8041 | 3346 | 2972 | 3537 | 4739 |
| CAR 21:4 | 2386 | 3753 | 2845 |  | 1080 |  | 1094 | 1203 | 470 | 897 | 1716 |
| CAR 4:0 | 6377 | 9364 | 28310 |  | 5401 |  | 5676 | 2419 | 1818 | 5784 | 8144 |
| CAR 5:0 | 3834 | 5225 | 41759 |  | 6031 |  | 10254 | 3760 | 1624 | 8242 | 10091 |
| **Ceramides** |  |  |  |  |  |  |  |  |  |  |  |
| Cer 12:0;2O/25:0 | 5790 | 2573 | 4384 |  | 7415 |  | 6760 | 2571 | 2381 | 4158 | 4504 |
| Cer 12:2;2O/19:4 | 1294 | 427 | 748 |  | 2115 |  | 1548 | 1111 | 1342 | 1053 | 1205 |
| Cer 13:2;2O/30:3 | 3568 | 1885 | 1358 |  | 4075 |  | 3245 | 2220 | 2627 | 3870 | 2856 |
| Cer 20:2;4O|Cer 12:2;3O/8:0;(2OH) | 5049 | 4012 | 4442 |  | 3448 |  | 4456 | 1395 | 778 | 2922 | 3313 |

| Cer 24:3;3O|Cer 15:3;2O/9:0;O | 2210 | 1539 | 1906 | 1827 | 2689 | 916 | 661 | 1359 | 1638 |
| --- | --- | --- | --- | --- | --- | --- | --- | --- | --- |
| Cer 32:0;2O|Cer 16:0;2O/16:0 | 862 | 501 | 480 | 1203 | 831 | 327 | 435 | 549 | 649 |
| Cer 32:1;2O|Cer 16:1;2O/16:0 | 721 | 476 | 406 | 743 | 668 | 478 | 635 | 500 | 578 |
| Cer 33:1;2O|Cer 17:1;2O/16:0 | 357 | 131 | 153 | 306 | 322 | 124 | 194 | 119 | 213 |
| Cer 33:1;4O|Cer 18:0;3O/15:1;(2OH) | 3311 | 1600 | 2310 | 4713 | 3999 | 2383 | 2089 | 2999 | 2926 |
| Cer 34:1;2O|Cer 18:1;2O/16:0 | 3056 | 1641 | 1508 | 5361 | 4325 | 2214 | 2035 | 4062 | 3025 |
| Cer 34:1;3O|Cer 19:0;2O/15:1;O | 2111 | 1493 | 1475 | 3078 | 3967 | 2066 | 1265 | 2226 | 2210 |
| Cer 34:3;2O|Cer 12:2;2O/22:1 | 1309 | 913 | 1586 | 5868 | 3116 | 1645 | 1296 | 1639 | 2172 |
| Cer 36:0;3O|Cer 18:0;2O/18:0;O | 16791 | 12528 | 13843 | 19893 | 18259 | 8207 | 7289 | 12713 | 13690 |
| Cer 36:1;3O|Cer 19:0;2O/17:1;O | 603 | 399 | 342 | 752 | 897 | 556 | 386 | 592 | 566 |
| Cer 36:3;4O|Cer 19:2;3O/17:1;(2OH) | 1386 | 945 | 1129 | 1815 | 1459 | 974 | 845 | 1234 | 1223 |
| Cer 37:0;2O|Cer 19:0;2O/18:0 | 2138 | 1255 | 1433 | 2629 | 1859 | 624 | 673 | 1388 | 1500 |
| Cer 38:0;4O | 2304 | 1130 | 1621 | 2353 | 1764 | 963 | 617 | 1638 | 1549 |
| Cer 39:0;3O|Cer 18:0;2O/21:0;O | 12076 | 8240 | 11162 | 13885 | 10687 | 6435 | 4613 | 7148 | 9281 |
| Cer 40:0;2O|Cer 16:0;2O/24:0 | 1310 | 1032 | 748 | 1783 | 1062 | 505 | 559 | 669 | 959 |
| Cer 40:1;2O|Cer 16:1;2O/24:0 | 2693 | 1781 | 1834 | 3095 | 3430 | 1353 | 1653 | 1793 | 2204 |
| Cer 41:1;2O|Cer 18:1;2O/23:0 | 1667 | 1220 | 1029 | 2465 | 1736 | 769 | 994 | 1006 | 1361 |
| Cer 42:1;2O|Cer 18:1;2O/24:0 | 2040 | 1106 | 1139 | 3281 | 3506 | 1578 | 1473 | 1769 | 1987 |
| Cer 44:2;4O|Cer 28:1;3O(FA 16:0) | 1094 | 358 | 671 | 4319 | 3060 | 856 | 767 | 1212 | 1542 |
| **Diacylglycerols** |  |  |  |  |  |  |  |  |  |
| DG 16:0 | 1685 | 899 | 995 | 3076 | 4180 | 1078 | 708 | 1332 | 1744 |
| DG 18:0 | 5331 | 2425 | 1940 | 8857 | 10905 | 4712 | 2612 | 3569 | 5044 |
| DG 20:0 | 19921 | 6881 | 6684 | 50760 | 58579 | 21015 | 10815 | 16265 | 23865 |
| DG 21:0 | 634 | 254 | 431 | 1382 | 1457 | 452 | 389 | 673 | 709 |
| DG 22:0 | 10825 | 3534 | 4713 | 31635 | 31079 | 11617 | 8148 | 10677 | 14028 |
| DG 22:0|DG 10:0_12:0 | 687 | 310 | 598 | 1422 | 1337 | 910 | 843 | 931 | 880 |
| DG 22:0|DG 8:0_14:0 | 755 | 314 | 563 | 1315 | 1503 | 891 | 780 | 928 | 881 |
| DG 22:1 | 3803 | 1338 | 1989 | 12546 | 12643 | 3735 | 2409 | 3209 | 5209 |
| DG 22:1|DG 8:0_14:1 | 3703 | 1471 | 2049 | 11240 | 12260 | 4362 | 2700 | 3071 | 5107 |
| DG 24:0 | 7533 | 3141 | 5539 | 15187 | 14315 | 8479 | 8562 | 11591 | 9293 |
| DG 24:0|DG 10:0_14:0 | 2428 | 1217 | 2223 | 5903 | 5208 | 3528 | 3349 | 3885 | 3468 |
| DG 24:0|DG 8:0_16:0 | 3309 | 1134 | 2266 | 8147 | 8413 | 3276 | 3102 | 3115 | 4095 |
| DG 24:1 | 5583 | 1919 | 2924 | 16597 | 18749 | 6034 | 4051 | 5726 | 7698 |
| DG 24:1|DG 10:0_14:1 | 3335 | 1175 | 1865 | 8320 | 10083 | 3322 | 2370 | 3498 | 4246 |
| DG 24:2 | 507 | 242 | 322 | 1232 | 1052 | 433 | 423 | 404 | 577 |
| DG 24:3 | 294 | 235 | 307 | 697 | 815 | 279 | 216 | 438 | 410 |
| DG 25:0|DG 10:0_15:0 | 1070 | 299 | 530 | 1127 | 1651 | 912 | 918 | 799 | 913 |
| DG 26:0|DG 12:0_14:0 | 10042 | 4274 | 6632 | 26225 | 18771 | 10318 | 8429 | 15870 | 12570 |
| DG 26:1 | 5104 | 2137 | 2921 | 14586 | 15461 | 5275 | 4350 | 6494 | 7041 |
| DG 26:1|DG 8:0_18:1 | 3640 | 1296 | 1755 | 12175 | 10893 | 4027 | 3480 | 4651 | 5240 |
| DG 26:2 | 546 | 215 | 443 | 1703 | 1396 | 456 | 488 | 659 | 738 |
| DG 26:2|DG 8:0_18:2 | 500 | 275 | 394 | 1166 | 1098 | 417 | 474 | 650 | 622 |
| DG 26:3 | 163 | 96 | 218 | 539 | 495 | 174 | 243 | 350 | 285 |
| DG 27:0 | 1677 | 768 | 1183 | 2723 | 2224 | 1191 | 826 | 2291 | 1610 |
| DG 27:5 | 1203 | 588 | 1055 | 1809 | 1333 | 1017 | 1425 | 1261 | 1211 |
| DG 28:0|DG 12:0_16:0 | 33656 | 15371 | 23530 | 70629 | 56375 | 34722 | 21387 | 42036 | 37213 |

| DG 28:1 | 12210 | 4920 | 9092 | 27738 | 27705 | 9470 | 6745 | 17151 | 14379 |
| --- | --- | --- | --- | --- | --- | --- | --- | --- | --- |
| DG 28:1|DG 10:0_18:1 | 13116 | 4259 | 7825 | 36808 | 36225 | 15137 | 12632 | 18561 | 18070 |
| DG 28:2 | 3628 | 1758 | 2407 | 10003 | 9797 | 2842 | 2314 | 4310 | 4632 |
| DG 28:2|DG 14:1_14:1 | 3419 | 1585 | 1765 | 11262 | 10735 | 3558 | 2904 | 4344 | 4947 |
| DG 28:3 | 448 | 199 | 356 | 1297 | 1031 | 486 | 524 | 960 | 663 |
| DG 28:3|DG 10:0_18:3 | 370 | 201 | 408 | 1061 | 896 | 383 | 454 | 766 | 567 |
| DG 30:0 | 914 | 313 | 602 | 2533 | 1553 | 622 | 631 | 1332 | 1063 |
| DG 30:0|DG 14:0_16:0 | 123755 | 61431 | 75544 | 231900 | 196400 | 111203 | 66159 | 117957 | 123044 |
| DG 30:1|DG 16:0_14:1 | 37280 | 15160 | 22272 | 117940 | 97607 | 33684 | 20916 | 48589 | 49181 |
| DG 30:2 | 2467 | 931 | 1573 | 5841 | 5265 | 1715 | 1347 | 3023 | 2770 |
| DG 30:2|DG 12:0_18:2 | 3007 | 1007 | 1599 | 8753 | 7590 | 2608 | 2351 | 3845 | 3845 |
| DG 30:2|DG 14:1_16:1 | 2294 | 1060 | 1607 | 11905 | 10405 | 3324 | 2283 | 3684 | 4570 |
| DG 30:3|DG 12:0_18:3 | 728 | 273 | 564 | 2243 | 2101 | 738 | 641 | 1690 | 1122 |
| DG 30:5 | 576 | 468 | 559 | 764 | 704 | 264 | 220 | 515 | 509 |
| DG 30:6 | 8067 | 3357 | 5505 | 8987 | 10611 | 5026 | 6625 | 6797 | 6872 |
| DG 30:7 | 859 | 364 | 619 | 1675 | 1682 | 503 | 653 | 852 | 901 |
| DG 31:7 | 58758 | 23157 | 36217 | 71843 | 67282 | 28028 | 23470 | 42917 | 43959 |
| DG 31:8 | 2815 | 875 | 1236 | 3835 | 3810 | 1259 | 1839 | 1691 | 2170 |
| DG 32:1|DG 14:0_18:1 | 121992 | 60249 | 83561 | 271295 | 242067 | 123082 | 57340 | 102115 | 132713 |
| DG 32:2 | 10021 | 4232 | 6556 | 18944 | 16893 | 5353 | 3423 | 9611 | 9379 |
| DG 32:2|DG 14:1_18:1 | 16859 | 6062 | 7809 | 58870 | 52916 | 14054 | 10434 | 15623 | 22828 |
| DG 32:3 | 1558 | 882 | 1373 | 3374 | 2774 | 922 | 445 | 2153 | 1685 |
| DG 32:3|DG 14:0_18:3 | 2943 | 1419 | 1678 | 7900 | 6189 | 2504 | 2132 | 4032 | 3599 |
| DG 32:6 | 11747 | 6169 | 10683 | 12944 | 12070 | 6167 | 7982 | 11144 | 9863 |
| DG 32:7 | 3513 | 1203 | 1779 | 4094 | 4279 | 1335 | 1378 | 2399 | 2497 |
| DG 33:2 | 2111 | 932 | 1634 | 3575 | 1958 | 980 | 796 | 1416 | 1675 |
| DG 33:3 | 1135 | 401 | 803 | 1440 | 877 | 418 | 325 | 730 | 766 |
| DG 33:6 | 139532 | 89428 | 106705 | 138040 | 89935 | 56206 | 50621 | 86349 | 94602 |
| DG 33:8 | 7983 | 2495 | 4659 | 17170 | 15978 | 4858 | 4929 | 6673 | 8093 |
| DG 33:9 | 1412 | 412 | 1074 | 2225 | 1999 | 778 | 1040 | 1479 | 1302 |
| DG 34:0 | 1063 | 522 | 629 | 1536 | 1286 | 570 | 446 | 630 | 835 |
| DG 34:0|DG 16:0_18:0 | 15544 | 11753 | 18034 | 22638 | 16338 | 9160 | 5615 | 23265 | 15293 |
| DG 34:1|DG 16:0_18:1 | 203933 | 112721 | 148372 | 490846 | 344932 | 115238 | 82298 | 188031 | 210796 |
| DG 34:2 | 542 | 463 | 519 | 585 | 817 | 305 | 261 | 397 | 486 |
| DG 34:2|DG 16:0_18:2 | 69724 | 20748 | 31655 | 178548 | 142290 | 46789 | 31461 | 63355 | 73071 |
| DG 34:3|DG 16:0_18:3 | 10487 | 3630 | 6129 | 27344 | 19523 | 7777 | 5172 | 12176 | 11530 |
| DG 34:6 | 12224 | 8642 | 13282 | 14784 | 12381 | 7921 | 8354 | 13675 | 11408 |
| DG 34:7 | 5484 | 2637 | 3764 | 8785 | 7983 | 3024 | 3253 | 5549 | 5060 |
| DG 35:2 | 1529 | 644 | 970 | 2210 | 1685 | 726 | 692 | 1255 | 1214 |
| DG 35:3 | 818 | 316 | 597 | 1271 | 939 | 434 | 303 | 684 | 670 |
| DG 35:8 | 27827 | 12984 | 19315 | 51150 | 45181 | 13166 | 12415 | 25476 | 25939 |
| DG 35:9 | 6003 | 2377 | 4581 | 9313 | 8463 | 3243 | 3755 | 6267 | 5500 |
| DG 36:1|DG 18:0_18:1 | 13159 | 8505 | 16907 | 10036 | 11237 | 6526 | 5925 | 17229 | 11190 |
| DG 36:2|DG 18:1_18:1 | 60316 | 27416 | 43833 | 93225 | 84125 | 25754 | 21407 | 47736 | 50477 |
| DG 36:3|DG 18:1_18:2 | 16607 | 6880 | 10216 | 41108 | 36562 | 10873 | 7844 | 16779 | 18358 |
| DG 36:4|DG 18:1_18:3 | 4464 | 1898 | 2872 | 8149 | 6878 | 2680 | 2343 | 5416 | 4338 |

| DG 36:5|DG 16:0_20:5 | 1099 | 350 | 636 | 2146 | 2014 | 1004 | 565 | 1277 | 1136 |
| --- | --- | --- | --- | --- | --- | --- | --- | --- | --- |
| DG 36:5|DG 18:2_18:3 | 894 | 363 | 531 | 1937 | 1541 | 822 | 754 | 1114 | 994 |
| DG 36:7 | 8840 | 4993 | 5774 | 13474 | 11219 | 4542 | 3656 | 8826 | 7666 |
| DG 36:8 | 830 | 401 | 646 | 1115 | 563 | 285 | 234 | 679 | 594 |
| DG 37:9 | 8443 | 3541 | 7473 | 15341 | 13843 | 5648 | 6243 | 13080 | 9201 |
| DG 38:5|DG 16:0_22:5 | 6791 | 2819 | 4898 | 16322 | 13624 | 7907 | 4645 | 10875 | 8485 |
| DG 38:6 | 936 | 315 | 590 | 1330 | 975 | 462 | 307 | 686 | 700 |
| DG 40:6|DG 18:1_22:5 | 2012 | 929 | 1229 | 4042 | 2884 | 1492 | 1032 | 2108 | 1966 |
| DG 40:8 | 1242 | 545 | 1016 | 1411 | 1222 | 521 | 402 | 999 | 920 |
| DG 41:10 | 1725 | 1183 | 1072 | 4283 | 3141 | 861 | 672 | 2083 | 1877 |
| DG 41:11 | 767 | 449 | 620 | 1157 | 955 | 523 | 352 | 862 | 711 |
| DG 41:5 | 4411 | 2915 | 4285 | 7210 | 5713 | 2555 | 1824 | 4098 | 4126 |
| DG 41:6 | 42179 | 38522 | 36707 | 35035 | 18443 | 17907 | 10004 | 24939 | 27967 |
| DG 43:11 | 1942 | 1075 | 1524 | 1905 | 1820 | 884 | 668 | 1353 | 1396 |
| DG 43:3 | 1263 | 733 | 1036 | 1731 | 1432 | 565 | 533 | 1070 | 1045 |
| DG 43:4 | 4488 | 3179 | 4322 | 5866 | 4899 | 2156 | 1787 | 4562 | 3907 |
| DG 43:6 | 23485 | 19503 | 33771 | 31013 | 28085 | 17903 | 11944 | 28040 | 24218 |
| DG 43:7 | 21143 | 22284 | 28201 | 21879 | 15431 | 10000 | 7757 | 17754 | 18056 |
| DG 44:7 | 1007 | 679 | 864 | 1917 | 1812 | 911 | 1236 | 1194 | 1202 |
| DG 44:9 | 540 | 477 | 545 | 2174 | 1994 | 943 | 1602 | 2281 | 1320 |
| DG 46:6 | 4393 | 4561 | 3642 | 4034 | 4443 | 3946 | 4066 | 5253 | 4292 |
| DG 47:7 | 14367 | 13485 | 15616 | 27311 | 18309 | 9089 | 11570 | 9793 | 14942 |
| DG 47:8 | 9492 | 9994 | 10870 | 19944 | 17677 | 9198 | 7549 | 10930 | 11957 |
| DG 51:7 | 5915 | 3146 | 3644 | 9766 | 6352 | 3153 | 2205 | 4692 | 4859 |
| DG 51:8 | 2707 | 1433 | 1894 | 4268 | 3393 | 1470 | 1189 | 2150 | 2313 |
| **Free Fatty Acids** |  |  |  |  |  |  |  |  |  |
| FA 12:0 | 751 | 665 | 652 | 1602 | 1355 | 1012 | 811 | 972 | 977 |
| FA 14:0 | 4954 | 3962 | 3380 | 7267 | 6201 | 6582 | 5752 | 6156 | 5532 |
| FA 14:1 | 457 | 280 | 328 | 2594 | 2020 | 1205 | 407 | 441 | 966 |
| FA 15:0 | 702 | 436 | 913 | 1285 | 1140 | 533 | 528 | 760 | 787 |
| FA 15:4 | 3872 | 3454 | 5679 | 6475 | 3912 | 1849 | 1646 | 4754 | 3955 |
| FA 16:0 | 476825 | 396412 | 468393 | 654118 | 513183 | 430985 | 385983 | 535128 | 482628 |
| FA 16:0;(2OH) | 595 | 408 | 538 | 717 | 612 | 338 | 242 | 645 | 512 |
| FA 16:0;O | 1880 | 1577 | 2914 | 4882 | 4368 | 2134 | 1924 | 2606 | 2786 |
| FA 16:1 | 2544 | 1722 | 2540 | 15027 | 17636 | 9200 | 3958 | 3354 | 6997 |
| FA 16:1;O | 2732 | 1997 | 3234 | 4636 | 3703 | 2115 | 1726 | 2770 | 2864 |
| FA 17:0 | 1373 | 1113 | 1786 | 2218 | 1549 | 1198 | 1171 | 1634 | 1505 |
| FA 17:1 | 114 | 23 | 175 | 563 | 511 | 144 | 111 | 192 | 229 |
| FA 17:4;O | 338 | 424 | 1701 | 580 | 1231 | 459 | 528 | 551 | 726 |
| FA 18:0 | 578111 | 452326 | 516969 | 720173 | 552789 | 441034 | 410858 | 640687 | 539118 |
| FA 18:0;(2OH) | 2463 | 2257 | 3657 | 3364 | 2977 | 2003 | 1286 | 2948 | 2619 |
| FA 18:0;O | 3177 | 2407 | 4558 | 10145 | 8866 | 3855 | 2443 | 4038 | 4936 |
| FA 18:1 | 29316 | 16419 | 32610 | 90138 | 89179 | 41770 | 25662 | 36360 | 45182 |
| FA 18:1;2O | 396 | 413 | 946 | 1267 | 1176 | 452 | 306 | 651 | 701 |
| FA 18:1;O | 1849 | 1099 | 2861 | 6303 | 6210 | 2466 | 1433 | 2627 | 3106 |
| FA 18:2 | 1413 | 835 | 1638 | 4313 | 4513 | 2279 | 1859 | 2348 | 2400 |

| FA 18:2;O | 1785 | 1367 | 2264 | 3696 | 3651 | 1879 | 1352 | 2392 | 2298 |
| --- | --- | --- | --- | --- | --- | --- | --- | --- | --- |
| FA 18:3 | 647 | 359 | 634 | 3479 | 3408 | 1802 | 1181 | 1355 | 1608 |
| FA 18:3;4O | 581 | 637 | 1495 | 1065 | 889 | 552 | 516 | 886 | 828 |
| FA 18:3;O | 174 | 176 | 513 | 484 | 294 | 227 | 205 | 338 | 301 |
| FA 19:0 | 298 | 154 | 259 | 562 | 513 | 284 | 201 | 313 | 323 |
| FA 19:0;(2OH) | 901 | 711 | 1106 | 725 | 1184 | 906 | 969 | 874 | 922 |
| FA 19:1 | 92 | 29 | 52 | 347 | 277 | 172 | 76 | 84 | 141 |
| FA 19:1;2O | 696 | 634 | 1478 | 1201 | 808 | 392 | 377 | 914 | 812 |
| FA 19:1;O | 192 | 35 | 78 | 803 | 816 | 252 | 39 | 137 | 294 |
| FA 20:0 | 5567 | 3564 | 5071 | 7744 | 5005 | 3797 | 4328 | 7502 | 5322 |
| FA 20:1 | 370 | 192 | 251 | 777 | 1415 | 382 | 306 | 608 | 538 |
| FA 20:3 | 104 | 53 | 157 | 501 | 523 | 303 | 212 | 225 | 260 |
| FA 20:4 | 982 | 278 | 898 | 3090 | 2339 | 1361 | 1196 | 908 | 1382 |
| FA 20:5 | 431 | 453 | 714 | 654 | 1777 | 528 | 196 | 442 | 650 |
| FA 21:0 | 743 | 371 | 483 | 946 | 550 | 457 | 469 | 684 | 588 |
| FA 21:1;2O | 166 | 280 | 344 | 304 | 345 | 193 | 238 | 330 | 275 |
| FA 22:0 | 2447 | 1525 | 2037 | 2972 | 2562 | 1666 | 1744 | 2845 | 2225 |
| FA 22:1 | 142 | 166 | 307 | 195 | 203 | 91 | 57 | 238 | 175 |
| FA 22:5 | 615 | 271 | 871 | 937 | 1343 | 925 | 621 | 1197 | 848 |
| FA 22:6 | 1074 | 1165 | 2088 | 1910 | 1188 | 1119 | 673 | 1160 | 1297 |
| FA 23:0 | 1716 | 844 | 1275 | 2489 | 1648 | 947 | 822 | 2055 | 1474 |
| FA 23:1 | 58 | 108 | 69 | 270 | 190 | 75 | 91 | 30 | 111 |
| FA 24:0 | 10630 | 5792 | 8283 | 13699 | 11200 | 5543 | 5976 | 12593 | 9214 |
| FA 25:0 | 3859 | 2736 | 4084 | 6320 | 4628 | 2488 | 2269 | 4913 | 3912 |
| FA 26:0 | 21949 | 12984 | 17631 | 27645 | 24395 | 11416 | 12497 | 27096 | 19452 |
| FA 26:1;O | 1217 | 873 | 1080 | 1472 | 1409 | 754 | 831 | 1089 | 1091 |
| FA 27:0 | 7146 | 4256 | 5350 | 9935 | 7259 | 4247 | 3328 | 7836 | 6169 |
| FA 27:1;O | 192 | 318 | 421 | 701 | 488 | 232 | 273 | 593 | 402 |
| FA 28:0 | 35568 | 18479 | 25538 | 41089 | 33150 | 18143 | 16635 | 36842 | 28180 |
| FA 29:0 | 8915 | 4935 | 7515 | 10780 | 9265 | 4988 | 4564 | 10429 | 7674 |
| FA 30:0 | 51912 | 21918 | 28150 | 70842 | 50274 | 26320 | 21445 | 43963 | 39353 |
| FA 31:0 | 7668 | 3522 | 4787 | 9180 | 6438 | 2960 | 2985 | 7920 | 5683 |
| FA 32:0 | 20357 | 9303 | 12459 | 26295 | 20020 | 8337 | 7282 | 17412 | 15183 |
| FA 33:0 | 2221 | 1439 | 1634 | 3146 | 2497 | 1110 | 1018 | 2735 | 1975 |
| FA 34:0 | 5440 | 2138 | 3766 | 7171 | 4517 | 1880 | 1652 | 5080 | 3956 |
| FA 34:8 | 1181 | 222 | 207 | 1890 | 2115 | 1310 | 1085 | 761 | 1096 |
| FA 42:5 | 2167737 | 816085 | 1695161 | 2866243 | 1840283 | 564773 | 686766 | 1513386 | 1518804 |
| FA 42:6 | 1529 | 794 | 1731 | 3068 | 1206 | 687 | 681 | 1157 | 1357 |
| FA 44:5 | 707319 | 220152 | 524604 | 1549678 | 601861 | 184601 | 220370 | 443305 | 556486 |
| FA 44:6 | 1074 | 497 | 1005 | 3247 | 1180 | 492 | 503 | 825 | 1103 |
| **Dihexosylceramides** |  |  |  |  |  |  |  |  |  |
| Hex2Cer 32:1;2O|Hex2Cer 16:1;2O/16:0 | 238 | 218 | 141 | 175 | 222 | 113 | 187 | 153 | 181 |
| Hex2Cer 33:1;2O|Hex2Cer 17:1;2O/16:0 | 201 | 142 | 114 | 264 | 182 | 126 | 155 | 173 | 170 |
| Hex2Cer 34:0;2O | 751 | 482 | 293 | 977 | 891 | 531 | 549 | 539 | 627 |
| Hex2Cer 34:1;2O|Hex2Cer 18:1;2O/16:0 | 4083 | 3268 | 1659 | 6929 | 6043 | 3941 | 4318 | 4051 | 4286 |
| Hex2Cer 36:1;2O|Hex2Cer 18:1;2O/18:0 | 138 | 149 | 86 | 200 | 163 | 97 | 160 | 100 | 137 |

| Hex2Cer 38:1;2O|Hex2Cer 16:1;2O/22:0 | 773 | 688 | 418 | 805 | 752 | 394 | 737 | 427 | 624 |
| --- | --- | --- | --- | --- | --- | --- | --- | --- | --- |
| Hex2Cer 39:0;2O|Hex2Cer 17:0;2O/22:0 | 942 | 1070 | 565 | 896 | 715 | 403 | 566 | 303 | 682 |
| Hex2Cer 39:1;2O|Hex2Cer 16:1;2O/23:0 | 1149 | 1062 | 628 | 746 | 1144 | 818 | 1001 | 601 | 894 |
| Hex2Cer 40:1;2O|Hex2Cer 18:1;2O/22:0 | 2319 | 1970 | 1366 | 2750 | 2585 | 1495 | 1737 | 1918 | 2018 |
| Hex2Cer 40:2;2O|Hex2Cer 16:1;2O/24:1 | 118 | 91 | 46 | 110 | 92 | 64 | 140 | 37 | 87 |
| Hex2Cer 41:1;2O|Hex2Cer 18:1;2O/23:0 | 1671 | 1700 | 1423 | 952 | 1785 | 684 | 1135 | 1704 | 1382 |
| Hex2Cer 42:1;2O|Hex2Cer 18:1;2O/24:0 | 1448 | 1675 | 1035 | 1495 | 1278 | 742 | 814 | 1185 | 1209 |
| Hex2Cer 42:2;2O|Hex2Cer 18:1;2O/24:1 | 315 | 301 | 248 | 125 | 184 | 266 | 187 | 168 | 224 |
|  |  |  |  |  |  |  |  |  |  |
| **hexosylceramides** |  |  |  |  |  |  |  |  |  |
| HexCer 33:1;2O|HexCer 17:1;2O/16:0 | 564 | 380 | 462 | 1133 | 1006 | 364 | 371 | 478 | 595 |
| HexCer 34:0;2O|HexCer 18:0;2O/16:0 | 435 | 314 | 220 | 642 | 523 | 118 | 236 | 276 | 346 |
| HexCer 34:1;2O | 1058 | 996 | 898 | 1863 | 1665 | 399 | 828 | 950 | 1082 |
| HexCer 34:1;2O|HexCer 18:1;2O/16:0 | 1520 | 1285 | 1126 | 3503 | 2699 | 781 | 1110 | 1338 | 1670 |
| HexCer 34:1;3O|HexCer 18:1;2O/16:0;O | 397 | 188 | 274 | 1626 | 1197 | 398 | 372 | 605 | 632 |
| HexCer 38:1;2O | 203 | 137 | 273 | 135 | 304 | 66 | 187 | 69 | 172 |
| HexCer 39:1;3O | 532 | 602 | 378 | 1536 | 1095 | 330 | 510 | 505 | 686 |
| HexCer 40:0;3O | 142 | 93 | 77 | 318 | 390 | 77 | 111 | 131 | 167 |
| HexCer 40:1;2O | 2028 | 1466 | 1179 | 3599 | 2918 | 812 | 1513 | 1438 | 1869 |
| HexCer 40:1;2O|HexCer 18:1;2O/22:0 | 428 | 902 | 984 | 1192 | 1190 | 302 | 702 | 359 | 757 |
| HexCer 40:1;3O | 1065 | 740 | 796 | 2434 | 3316 | 725 | 779 | 1540 | 1424 |
| HexCer 41:1;2O | 2126 | 1770 | 1514 | 2579 | 3307 | 836 | 1303 | 1374 | 1851 |
| HexCer 41:1;2O|HexCer 18:1;2O/23:0 | 6195 | 3504 | 5229 | 9889 | 8565 | 2852 | 3779 | 5854 | 5733 |
| HexCer 41:1;3O | 1030 | 749 | 1007 | 5218 | 5361 | 1282 | 965 | 2363 | 2247 |
| HexCer 42:1;2O | 2849 | 2189 | 1451 | 6070 | 7296 | 1425 | 2663 | 1599 | 3193 |
| HexCer 42:1;3O | 1038 | 784 | 1018 | 4774 | 4509 | 1261 | 838 | 2593 | 2102 |
| HexCer 43:1;3O | 361 | 84 | 250 | 1208 | 776 | 245 | 290 | 258 | 434 |
| HexCer 49:5;4O | 2448 | 2193 | 718 | 1503 | 894 | 935 | 1060 | 1173 | 1365 |
| HexCer 51:9;3O | 837 | 739 | 162 | 171 | 416 | 55 | 552 | 214 | 393 |
| HexCer 53:9;3O | 984 | 963 | 457 | 904 | 652 | 261 | 361 | 396 | 622 |
| **Lysophophatidylcholines** |  |  |  |  |  |  |  |  |  |
| LPC 14:0 | 1587 | 1420 | 1691 | 1724 | 3120 | 1076 | 960 | 3614 | 1899 |
| LPC 15:0 | 1258 | 1157 | 1336 | 1404 | 1793 | 677 | 618 | 1529 | 1222 |
| LPC 16:0 | 9258 | 6912 | 7239 | 15499 | 25198 | 7047 | 5651 | 24991 | 12724 |
| LPC 17:0 | 355 | 246 | 298 | 419 | 608 | 202 | 223 | 653 | 376 |
| LPC 18:0 | 1020 | 1108 | 1004 | 2411 | 3218 | 994 | 1373 | 4196 | 1915 |
| LPC 18:1 | 2978 | 2401 | 3712 | 5508 | 7204 | 1954 | 2022 | 4892 | 3834 |
| LPC 18:2 | 1419 | 1266 | 1882 | 2104 | 2670 | 691 | 587 | 1658 | 1535 |
| LPC 26:0 | 468 | 399 | 477 | 644 | 669 | 385 | 395 | 446 | 485 |
| LPC 28:7 | 558 | 361 | 775 | 645 | 434 | 99 | 101 | 430 | 425 |
| **Lysophosphatidylethanolamines** |  |  |  |  |  |  |  |  |  |
| LPE 16:0 | 1665 | 1755 | 1048 | 4962 | 5207 | 2190 | 2492 | 3977 | 2912 |
| LPE 16:1 | 400 | 255 | 143 | 1139 | 1045 | 458 | 259 | 308 | 501 |
| LPE 18:0 | 1404 | 1132 | 894 | 2897 | 3493 | 1458 | 1744 | 4844 | 2233 |
| LPE 18:1 | 6950 | 5959 | 5467 | 19285 | 18647 | 9805 | 6461 | 9761 | 10292 |
| LPE 18:2 | 1632 | 1362 | 1332 | 4000 | 4802 | 1973 | 1873 | 2729 | 2463 |
| LPE 18:3 | 428 | 243 | 499 | 949 | 1128 | 497 | 279 | 914 | 617 |
| LPE 20:4 | 191 | 246 | 125 | 297 | 454 | 377 | 188 | 268 | 268 |

| LPI 18:0 | 1559 | 1041 | 1287 | 2178 | 2679 | 1316 | 4931 | 1947 | 2117 |
| --- | --- | --- | --- | --- | --- | --- | --- | --- | --- |
| LPI 18:1 | 1493 | 1186 | 977 | 3149 | 3504 | 801 | 5190 | 2215 | 2314 |
| LPI 18:2 | 387 | 341 | 328 | 751 | 611 | 170 | 794 | 467 | 481 |
| **Monoacylglycerols** |  |  |  |  |  |  |  |  |  |
| MG 15:0 | 3132 | 1209 | 1444 | 1259 | 2252 | 664 | 615 | 1543 | 1515 |
| MG 17:3 | 5305 | 2283 | 3524 | 3882 | 3269 | 1452 | 802 | 3356 | 2984 |
| MG 17:4 | 4807 | 4284 | 4568 | 3491 | 5299 | 1408 | 686 | 3367 | 3489 |
| MG 18:0 | 5067 | 4010 | 6122 | 6774 | 4979 | 2040 | 1844 | 4868 | 4463 |
| MG 19:3 | 987 | 773 | 1110 | 1575 | 891 | 346 | 257 | 893 | 854 |
| MG 21:4 | 2757 | 1067 | 1553 | 4900 | 3361 | 1802 | 1823 | 2255 | 2440 |
| MG 22:1 | 1346 | 1000 | 1367 | 2430 | 1891 | 881 | 612 | 1162 | 1336 |
| MG 24:2 | 489 | 351 | 427 | 711 | 683 | 325 | 243 | 405 | 454 |
| MG 30:7 | 7358 | 5464 | 8222 | 18574 | 18389 | 9849 | 8684 | 8095 | 10579 |
| MG 32:8 | 1303 | 605 | 897 | 2144 | 1688 | 881 | 1031 | 1101 | 1206 |
| MG 34:8 | 4319 | 1779 | 2643 | 6283 | 5285 | 2072 | 1833 | 3360 | 3447 |
| MG 36:6 | 1137 | 516 | 640 | 1975 | 1973 | 1083 | 1016 | 1135 | 1184 |
| **N-acyl ethanolamines** |  |  |  |  |  |  |  |  |  |
| NAE 12:0 | 5781 | 4007 | 5187 | 5397 | 5681 | 2202 | 1833 | 3919 | 4251 |
| NAE 13:1 | 11355 | 10502 | 12747 | 8456 | 9056 | 2341 | 1824 | 5247 | 7691 |
| NAE 14:1 | 15453 | 15055 | 15709 | 14408 | 19076 | 5009 | 4115 | 10904 | 12466 |
| NAE 15:1 | 2352 | 2063 | 2480 | 1919 | 2623 | 813 | 596 | 1674 | 1815 |
| NAE 16:1 | 552886 | 530657 | 565760 | 431353 | 769426 | 209601 | 135534 | 351987 | 443400 |
| NAE 16:2 | 1336273 | 1224177 | 1313104 | 971744 | 1887703 | 491721 | 283866 | 879639 | 1048528 |
| NAE 16:3 | 770399 | 652210 | 762035 | 672478 | 801889 | 259746 | 212844 | 534461 | 583258 |
| NAE 16:4 | 58723 | 47714 | 54227 | 40277 | 64906 | 16473 | 9467 | 37641 | 41179 |
| NAE 17:4 | 9113 | 6933 | 7308 | 6695 | 7832 | 2392 | 2106 | 6047 | 6053 |
| NAE 18:1 | 19304 | 18243 | 17039 | 16475 | 32237 | 9368 | 5701 | 12853 | 16402 |
| NAE 18:2 | 28488 | 27595 | 27938 | 22664 | 45438 | 11280 | 6518 | 17908 | 23479 |
| NAE 18:3 | 4494 | 3754 | 5221 | 4434 | 5447 | 1783 | 1438 | 3731 | 3788 |
| NAE 18:4 | 178640 | 139859 | 148562 | 104028 | 200919 | 47802 | 30165 | 113246 | 120403 |
| NAE 18:5 | 276582 | 214240 | 252956 | 202310 | 297989 | 84512 | 60523 | 216097 | 200651 |
| NAE 20:1 | 5285 | 2640 | 3279 | 3767 | 5636 | 1525 | 1166 | 2355 | 3207 |
| NAE 20:2 | 9020 | 5933 | 6607 | 6475 | 12006 | 4181 | 2259 | 5111 | 6449 |
| NAE 20:4 | 6828 | 5802 | 5378 | 4048 | 10384 | 2765 | 1724 | 4442 | 5171 |
| NAE 20:5 | 5688 | 4448 | 5012 | 4253 | 7696 | 1996 | 1378 | 3996 | 4308 |
| NAE 22:3 | 2265 | 1860 | 2367 | 2458 | 2478 | 1119 | 865 | 1709 | 1890 |
| NAE 22:4 | 2951 | 1312 | 1808 | 1562 | 2850 | 880 | 529 | 1123 | 1627 |
| NAE 22:5 | 2357 | 1637 | 1773 | 1968 | 3032 | 1080 | 529 | 1387 | 1720 |
| NAE 24:4 | 1873 | 1849 | 2184 | 2075 | 1824 | 626 | 542 | 1397 | 1546 |
| NAE 5:0 | 5178 | 5328 | 14401 | 3265 | 4039 | 879 | 976 | 4660 | 4841 |
| NAE 6:0 | 37318 | 46386 | 81229 | 18399 | 22324 | 5620 | 4228 | 17199 | 29088 |
| NAE 7:0 | 844 | 804 | 994 | 598 | 1062 | 276 | 77 | 519 | 647 |
| **Phosphatidylcholines** |  |  |  |  |  |  |  |  |  |
| PC 24:0 | 361 | 285 | 366 | 871 | 842 | 341 | 475 | 737 | 535 |
| PC 26:0|PC 12:0_14:0 | 1152 | 907 | 1355 | 2809 | 2892 | 1452 | 1716 | 3190 | 1934 |

| PC 27:0|PC 12:0_15:0 | 270 | 184 | 246 | 606 | 519 | 311 | 350 | 806 | 412 |
| --- | --- | --- | --- | --- | --- | --- | --- | --- | --- |
| PC 28:0 | 683 | 496 | 444 | 982 | 960 | 661 | 423 | 906 | 695 |
| PC 28:0|PC 14:0_14:0 | 25858 | 18864 | 20497 | 44512 | 42146 | 35381 | 30510 | 46504 | 33034 |
| PC 28:1 | 335 | 212 | 339 | 765 | 703 | 372 | 298 | 618 | 455 |
| PC 29:0|PC 14:0_15:0 | 4732 | 3153 | 3816 | 7785 | 9108 | 6000 | 5599 | 7405 | 5950 |
| PC 30:0|PC 14:0_16:0 | 435289 | 310381 | 346923 | 850580 | 801054 | 544167 | 487621 | 578815 | 544354 |
| PC 30:1|PC 14:0_16:1 | 4894 | 3130 | 3008 | 12237 | 11876 | 6482 | 4217 | 6929 | 6597 |
| PC 30:3 | 545 | 269 | 446 | 990 | 818 | 464 | 365 | 648 | 568 |
| PC 31:0|PC 15:0_16:0 | 18451 | 15425 | 24472 | 50703 | 47715 | 26464 | 26022 | 48312 | 32195 |
| PC 31:1 | 1423 | 884 | 1209 | 3145 | 2695 | 1763 | 1479 | 1981 | 1822 |
| PC 31:1|PC 15:0_16:1 | 1472 | 978 | 894 | 3547 | 2933 | 1662 | 1334 | 1736 | 1819 |
| PC 32:0 | 6370 | 4228 | 7002 | 24670 | 22614 | 11136 | 10836 | 17497 | 13044 |
| PC 32:0|PC 16:0_16:0 | 344521 | 246223 | 375041 | 1149151 | 1024539 | 505000 | 502096 | 796305 | 617859 |
| PC 32:1 | 2097 | 1079 | 1033 | 4478 | 5226 | 1905 | 1583 | 2547 | 2494 |
| PC 32:1|PC 14:0_18:1 | 144440 | 84814 | 75396 | 384180 | 354930 | 163577 | 115709 | 159291 | 185292 |
| PC 32:2|PC 16:1_16:1 | 5842 | 3739 | 3793 | 9434 | 10092 | 6150 | 7603 | 5341 | 6499 |
| PC 32:3|PC 14:0_18:3 | 636 | 407 | 407 | 1145 | 1028 | 534 | 749 | 938 | 731 |
| PC 32:3|PC 14:1_18:2 | 6659 | 4986 | 4979 | 14642 | 13898 | 11107 | 9928 | 11876 | 9759 |
| PC 33:0|PC 16:0_17:0 | 7543 | 6445 | 11871 | 20347 | 16850 | 8714 | 8220 | 13700 | 11711 |
| PC 33:1|PC 15:0_18:1 | 19449 | 13536 | 17045 | 39105 | 37077 | 21278 | 23152 | 25040 | 24460 |
| PC 33:2 | 2044 | 823 | 1324 | 2316 | 2940 | 1343 | 1938 | 1780 | 1813 |
| PC 34:0|PC 16:0_18:0 | 34585 | 26390 | 58992 | 96167 | 85767 | 48553 | 62574 | 140285 | 69164 |
| PC 34:1 | 17846 | 12641 | 16087 | 40574 | 34531 | 18490 | 17246 | 25307 | 22840 |
| PC 34:1|PC 16:0_18:1 | 877993 | 589101 | 757844 | 1769450 | 1543891 | 840556 | 807963 | 1051959 | 1029844 |
| PC 34:2 | 4533 | 3223 | 4083 | 6869 | 6597 | 3090 | 3816 | 4581 | 4599 |
| PC 34:2|PC 15:1_19:1 | 1486 | 903 | 1087 | 2085 | 2023 | 1096 | 999 | 1199 | 1360 |
| PC 34:2|PC 16:0_18:2 | 144232 | 110988 | 148789 | 276642 | 258163 | 141043 | 152620 | 159993 | 174059 |
| PC 34:3|PC 16:0_18:3 | 16225 | 10114 | 15655 | 54239 | 51014 | 24190 | 27521 | 37304 | 29533 |
| PC 34:4|PC 17:2_17:2 | 401 | 215 | 255 | 1152 | 950 | 367 | 385 | 594 | 540 |
| PC 35:0|PC 17:0_18:0 | 1585 | 1135 | 2530 | 3151 | 2541 | 1566 | 1889 | 2762 | 2145 |
| PC 35:1|PC 17:0_18:1 | 7978 | 6334 | 9513 | 14291 | 9201 | 6770 | 7122 | 12144 | 9169 |
| PC 35:2|PC 17:1_18:1 | 5219 | 3215 | 4466 | 9851 | 8690 | 3918 | 5163 | 5429 | 5744 |
| PC 35:3 | 957 | 554 | 709 | 2211 | 2005 | 832 | 1168 | 1197 | 1204 |
| PC 36:0|PC 18:0_18:0 | 1295 | 1071 | 2352 | 2307 | 2070 | 1323 | 1570 | 3092 | 1885 |
| PC 36:1 | 643 | 389 | 981 | 1471 | 1147 | 673 | 1190 | 1639 | 1017 |
| PC 36:1|PC 18:0_18:1 | 111526 | 79237 | 151544 | 236509 | 201758 | 107198 | 175515 | 326042 | 173666 |
| PC 36:2 | 1944 | 1564 | 1986 | 2967 | 2458 | 1238 | 1181 | 2077 | 1927 |
| PC 36:2|PC 18:1_18:1 | 131155 | 109335 | 134737 | 204197 | 171011 | 80335 | 74471 | 139929 | 130646 |
| PC 36:3 | 147 | 225 | 179 | 346 | 290 | 251 | 305 | 409 | 269 |
| PC 36:3|PC 18:1_18:2 | 65913 | 42795 | 58881 | 110538 | 104329 | 52123 | 77120 | 78434 | 73767 |
| PC 36:4 | 533 | 348 | 562 | 1154 | 1057 | 521 | 580 | 1061 | 727 |
| PC 36:4|PC 18:2_18:2 | 17347 | 9819 | 14703 | 44684 | 42820 | 16341 | 24932 | 31367 | 25251 |
| PC 36:5|PC 16:0_20:5 | 3649 | 2038 | 3117 | 7737 | 7841 | 5394 | 5039 | 4860 | 4959 |
| PC 36:5|PC 18:2_18:3 | 1334 | 884 | 1141 | 2614 | 2684 | 1022 | 1649 | 2196 | 1691 |
| PC 36:6 | 98 | 71 | 93 | 359 | 271 | 84 | 113 | 205 | 162 |
| PC 37:1|PC 19:0_18:1 | 641 | 497 | 781 | 648 | 843 | 408 | 535 | 1064 | 677 |

| PC 37:2|PC 18:1_19:1 | 1571 | 947 | 1032 | 1714 | 1533 | 775 | 785 | 1354 | 1214 |
| --- | --- | --- | --- | --- | --- | --- | --- | --- | --- |
| PC 38:1|PC 20:0_18:1 | 1227 | 947 | 1226 | 1562 | 1849 | 908 | 627 | 1524 | 1234 |
| PC 38:2 | 1339 | 798 | 1189 | 1708 | 1339 | 875 | 988 | 1166 | 1175 |
| PC 38:2|PC 20:0_18:2 | 2805 | 2052 | 2085 | 3584 | 2638 | 2405 | 1679 | 4386 | 2704 |
| PC 38:3 | 2110 | 1148 | 1965 | 2699 | 2411 | 1560 | 2182 | 3109 | 2148 |
| PC 38:3|PC 20:0_18:3 | 1048 | 584 | 743 | 1462 | 1296 | 849 | 688 | 1983 | 1082 |
| PC 38:4 | 1877 | 1232 | 2391 | 4231 | 3062 | 1196 | 2087 | 3522 | 2450 |
| PC 38:4|PC 18:1_20:3 | 2767 | 1749 | 2047 | 5011 | 4659 | 2825 | 3899 | 4982 | 3492 |
| PC 38:5|PC 16:0_22:5 | 5724 | 3361 | 6596 | 11736 | 9861 | 7831 | 8951 | 10761 | 8103 |
| PC 38:6|PC 19:3_19:3 | 1412 | 760 | 1288 | 3260 | 3003 | 1742 | 2114 | 2880 | 2057 |
| PC 38:7 | 358 | 231 | 321 | 858 | 750 | 407 | 475 | 551 | 494 |
| PC 40:3|PC 25:1_15:2 | 520 | 302 | 347 | 634 | 530 | 469 | 361 | 775 | 492 |
| PC 40:5|PC 18:0_22:5 | 790 | 716 | 1186 | 1243 | 961 | 700 | 1099 | 2327 | 1128 |
| PC 40:6 | 1175 | 855 | 1256 | 2048 | 2011 | 1050 | 1494 | 2166 | 1507 |
| PC O-29:1 | 378 | 309 | 296 | 580 | 635 | 417 | 517 | 497 | 454 |
| PC O-31:0 | 6481 | 3755 | 2997 | 11768 | 10870 | 5845 | 4273 | 8580 | 6821 |
| PC O-31:1 | 1461 | 908 | 1248 | 3334 | 2853 | 2001 | 2142 | 2820 | 2096 |
| PC O-31:6 | 123 | 101 | 78 | 358 | 224 | 130 | 120 | 222 | 169 |
| PC O-33:2 | 2092 | 1250 | 1847 | 3105 | 3058 | 1983 | 3149 | 2535 | 2377 |
| PC O-33:3 | 1224 | 897 | 1060 | 2188 | 1954 | 925 | 1274 | 1606 | 1391 |
| PC O-33:6 | 530 | 334 | 502 | 953 | 789 | 478 | 474 | 644 | 588 |
| PC O-35:6 | 1078 | 683 | 988 | 3204 | 2933 | 1476 | 1133 | 2027 | 1690 |
| PC O-35:9 | 1232 | 923 | 954 | 2628 | 2475 | 1725 | 1741 | 1985 | 1708 |
| PC O-37:6 | 331 | 236 | 323 | 620 | 538 | 277 | 304 | 497 | 391 |
| PC O-38:10 | 1175 | 696 | 850 | 2252 | 1734 | 1057 | 1097 | 1270 | 1266 |
| PC O-39:0 | 1103 | 1071 | 995 | 1631 | 1424 | 640 | 672 | 1373 | 1114 |
| PC O-39:10 | 2746 | 2072 | 2355 | 5487 | 5192 | 3016 | 2741 | 4101 | 3464 |
| PC O-33:4 | 2542 | 1901 | 2125 | 9198 | 8310 | 4778 | 6470 | 9369 | 5587 |
| **Phosphatidylethanolamines** |  |  |  |  |  |  |  |  |  |
| PE 28:0|PE 14:0_14:0 | 2453 | 2092 | 1180 | 2328 | 1933 | 1581 | 1184 | 1226 | 1747 |
| PE 30:0|PE 14:0_16:0 | 6644 | 5035 | 4981 | 5681 | 5107 | 4108 | 3063 | 3248 | 4734 |
| PE 30:1|PE 12:0_18:1 | 2927 | 1934 | 1016 | 3420 | 3071 | 2011 | 1352 | 2114 | 2231 |
| PE 31:0|PE 15:0_16:0 | 919 | 682 | 537 | 1367 | 978 | 571 | 503 | 322 | 735 |
| PE 31:1 | 335 | 257 | 163 | 771 | 762 | 402 | 332 | 453 | 435 |
| PE 31:1|PE 15:0_16:1 | 305 | 244 | 134 | 491 | 471 | 215 | 126 | 260 | 281 |
| PE 32:0|PE 16:0_16:0 | 10573 | 7930 | 7500 | 15767 | 12683 | 7643 | 5078 | 8013 | 9398 |
| PE 32:1|PE 14:0_18:1 | 42089 | 31706 | 21775 | 71040 | 57076 | 33617 | 20199 | 33648 | 38894 |
| PE 32:1|PE 16:0_16:1 | 7045 | 4657 | 3798 | 17991 | 15997 | 7836 | 5787 | 7498 | 8826 |
| PE 32:2 | 709 | 369 | 298 | 1350 | 1084 | 610 | 601 | 625 | 706 |
| PE 32:2|PE 14:0_18:2 | 5622 | 4660 | 2608 | 7210 | 6662 | 4195 | 3574 | 4361 | 4861 |
| PE 32:3|PE 14:0_18:3 | 232 | 255 | 71 | 489 | 624 | 318 | 238 | 281 | 313 |
| PE 33:0|PE 16:0_17:0 | 1005 | 923 | 1092 | 1089 | 1296 | 838 | 656 | 917 | 977 |
| PE 33:1|PE 15:0_18:1 | 8710 | 7229 | 5457 | 13092 | 11517 | 6144 | 5216 | 6866 | 8029 |
| PE 34:0|PE 16:0_18:0 | 3764 | 2813 | 3490 | 4896 | 4248 | 2660 | 2610 | 4127 | 3576 |
| PE 34:1 | 5465 | 3964 | 4046 | 9761 | 8061 | 4476 | 3956 | 5686 | 5677 |

| PE 34:1|PE 16:0_18:1 | 357606 | 269395 | 233524 | 461985 | 394075 | 220651 | 164073 | 243675 | 293123 |
| --- | --- | --- | --- | --- | --- | --- | --- | --- | --- |
| PE 34:2 | 1281 | 911 | 842 | 3065 | 2411 | 1301 | 1294 | 1338 | 1555 |
| PE 34:2|PE 16:0_18:2 | 147876 | 114600 | 83601 | 213218 | 168470 | 99888 | 86783 | 127277 | 130214 |
| PE 34:3|PE 16:0_18:3 | 15149 | 11328 | 9670 | 23814 | 20996 | 10819 | 7794 | 18624 | 14774 |
| PE 34:3|PE 16:1_18:2 | 3531 | 2481 | 1850 | 11189 | 9047 | 3906 | 2465 | 3985 | 4807 |
| PE 34:4|PE 14:0_20:4 | 645 | 553 | 450 | 1409 | 1084 | 823 | 774 | 897 | 829 |
| PE 34:4|PE 16:1_18:3 | 433 | 264 | 166 | 1702 | 1303 | 582 | 281 | 820 | 694 |
| PE 35:0|PE 17:0_18:0 | 243 | 237 | 142 | 92 | 297 | 74 | 159 | 245 | 186 |
| PE 35:1|PE 17:0_18:1 | 16655 | 14560 | 12693 | 17645 | 15670 | 9872 | 9481 | 11469 | 13506 |
| PE 35:2|PE 17:1_18:1 | 13034 | 9692 | 8605 | 18173 | 15901 | 8918 | 7771 | 11219 | 11664 |
| PE 35:3|PE 17:1_18:2 | 944 | 820 | 566 | 1747 | 1740 | 909 | 894 | 1467 | 1136 |
| PE 36:0|PE 18:0_18:0 | 272 | 255 | 308 | 616 | 482 | 270 | 536 | 893 | 454 |
| PE 36:1|PE 18:0_18:1 | 262093 | 189343 | 203023 | 262489 | 220744 | 139139 | 156879 | 227414 | 207641 |
| PE 36:2 | 2045 | 1304 | 1655 | 3961 | 3613 | 1769 | 2466 | 3256 | 2509 |
| PE 36:2|PE 18:1_18:1 | 688265 | 535139 | 556646 | 909616 | 782685 | 520946 | 388037 | 611367 | 624088 |
| PE 36:3 | 1869 | 1410 | 1529 | 3924 | 3388 | 1863 | 1958 | 2208 | 2269 |
| PE 36:3;2O|PE 14:0_22:3;2O | 908 | 270 | 716 | 1588 | 1211 | 669 | 839 | 956 | 895 |
| PE 36:3|PE 18:1_18:2 | 185859 | 140964 | 140457 | 248208 | 217667 | 133268 | 119487 | 184469 | 171298 |
| PE 36:4|PE 16:0_20:4 | 21814 | 15219 | 12161 | 33459 | 28486 | 17124 | 13836 | 23037 | 20642 |
| PE 36:4|PE 18:1_18:3 | 33988 | 25998 | 27038 | 62000 | 57080 | 29947 | 23620 | 53249 | 39115 |
| PE 36:5|PE 16:0_20:5 | 3914 | 3033 | 2939 | 6070 | 5299 | 4364 | 2778 | 4972 | 4171 |
| PE 36:5|PE 18:2_18:3 | 1453 | 577 | 889 | 4102 | 3626 | 1369 | 1385 | 3160 | 2070 |
| PE 37:1|PE 18:0_19:1 | 1518 | 1113 | 1093 | 1318 | 770 | 789 | 1114 | 1163 | 1110 |
| PE 37:2|PE 18:1_19:1 | 5528 | 4027 | 4047 | 6532 | 5039 | 3316 | 3045 | 3425 | 4370 |
| PE 37:3|PE 18:1_19:2 | 1101 | 924 | 794 | 1244 | 985 | 928 | 870 | 1115 | 995 |
| PE 38:1|PE 20:0_18:1 | 3335 | 2371 | 1375 | 2332 | 2013 | 1688 | 1846 | 1783 | 2093 |
| PE 38:2|PE 18:1_20:1 | 5901 | 4080 | 3515 | 5827 | 5067 | 3978 | 3016 | 3423 | 4351 |
| PE 38:3|PE 18:0_20:3 | 12528 | 8254 | 8154 | 12076 | 11488 | 9533 | 9503 | 16640 | 11022 |
| PE 38:3|PE 18:1_20:2 | 2952 | 2060 | 2166 | 2953 | 2829 | 2096 | 1987 | 2770 | 2476 |
| PE 38:4|PE 18:0_20:4 | 15124 | 10733 | 14049 | 19550 | 17964 | 10792 | 11757 | 22929 | 15362 |
| PE 38:4|PE 18:1_20:3 | 10381 | 8304 | 8185 | 14975 | 14567 | 11313 | 8259 | 15131 | 11389 |
| PE 38:5|PE 16:0_22:5 | 24670 | 17449 | 17517 | 37332 | 28936 | 21206 | 19526 | 33550 | 25023 |
| PE 38:5|PE 18:1_20:4 | 3987 | 2465 | 3094 | 9713 | 8609 | 5888 | 6087 | 6029 | 5734 |
| PE 38:6 | 1044 | 604 | 794 | 2478 | 2187 | 1483 | 1097 | 1649 | 1417 |
| PE 39:1|PE 21:0_18:1 | 899 | 441 | 316 | 487 | 389 | 471 | 446 | 308 | 470 |
| PE 39:2|PE 18:1_21:1 | 1264 | 531 | 450 | 887 | 1150 | 790 | 554 | 566 | 774 |
| PE 40:1|PE 22:0_18:1 | 3398 | 1492 | 422 | 2073 | 1730 | 1879 | 1269 | 799 | 1633 |
| PE 40:2|PE 18:1_22:1 | 787 | 428 | 216 | 755 | 413 | 581 | 357 | 320 | 482 |
| PE 40:4|PE 18:0_22:4 | 1164 | 933 | 1054 | 1335 | 1244 | 899 | 1182 | 3054 | 1358 |
| PE 40:5|PE 18:0_22:5 | 14599 | 9931 | 10254 | 12166 | 10669 | 8751 | 10740 | 19070 | 12022 |
| PE 40:6|PE 18:1_22:5 | 2765 | 1457 | 2035 | 7025 | 5253 | 3397 | 4059 | 4300 | 3786 |
| PE 41:1|PE 23:0_18:1 | 844 | 419 | 194 | 216 | 208 | 516 | 378 | 73 | 356 |
| PE 44:10|PE 22:5_22:5 | 3624 | 1891 | 3030 | 4686 | 3964 | 2145 | 2069 | 3572 | 3123 |
| PE O-19:0_18:1 | 379 | 149 | 153 | 702 | 299 | 434 | 563 | 393 | 384 |
| PE O-30:1|PE O-14:1_16:0 | 291 | 282 | 217 | 221 | 174 | 239 | 276 | 175 | 234 |

| PE O-31:1|PE O-15:1_16:0 | 1554 | 1176 | 712 | 1478 | 1172 | 1001 | 1094 | 581 | 1096 |
| --- | --- | --- | --- | --- | --- | --- | --- | --- | --- |
| PE O-31:2|PE O-15:1_16:1 | 608 | 419 | 348 | 1209 | 954 | 702 | 760 | 646 | 706 |
| PE O-32:1|PE O-16:1_16:0 | 518 | 258 | 343 | 360 | 404 | 311 | 213 | 198 | 326 |
| PE O-32:2|PE O-14:1_18:1 | 4236 | 2944 | 2192 | 6326 | 4526 | 3168 | 3433 | 3795 | 3827 |
| PE O-32:3|PE O-14:1_18:2 | 1228 | 982 | 508 | 1582 | 1456 | 931 | 1308 | 1129 | 1141 |
| PE O-33:2|PE O-15:1_18:1 | 7127 | 5172 | 4842 | 9939 | 8478 | 6660 | 7513 | 7029 | 7095 |
| PE O-33:3|PE O-15:1_18:2 | 3658 | 2582 | 2541 | 4199 | 3781 | 3171 | 3763 | 2633 | 3291 |
| PE O-33:4|PE O-15:1_18:3 | 344 | 354 | 308 | 881 | 658 | 629 | 608 | 553 | 542 |
| PE O-34:2|PE O-16:1_18:1 | 24486 | 18711 | 18185 | 31746 | 25603 | 19456 | 16630 | 27755 | 22822 |
| PE O-34:3|PE O-16:1_18:2 | 14534 | 10547 | 10377 | 16590 | 15402 | 9713 | 9152 | 13772 | 12511 |
| PE O-34:4|PE O-16:1_18:3 | 1893 | 1528 | 1415 | 3746 | 3156 | 2103 | 2129 | 4426 | 2550 |
| PE O-35:1 | 6236 | 3733 | 3922 | 10289 | 8337 | 3551 | 3604 | 7600 | 5909 |
| PE O-35:2|PE O-17:1_18:1 | 2708 | 2117 | 2427 | 4101 | 3491 | 2359 | 2260 | 2887 | 2794 |
| PE O-35:3|PE O-17:1_18:2 | 1833 | 1160 | 1510 | 2293 | 2349 | 1366 | 1325 | 1561 | 1674 |
| PE O-35:3|PE O-17:2_18:1 | 546 | 399 | 518 | 757 | 345 | 495 | 580 | 595 | 529 |
| PE O-35:5|PE O-15:1_20:4 | 796 | 753 | 423 | 942 | 778 | 1001 | 1098 | 531 | 790 |
| PE O-35:6|PE O-15:1_20:5 | 302 | 232 | 163 | 348 | 225 | 358 | 397 | 255 | 285 |
| PE O-36:2|PE O-18:0_18:2 | 1565 | 1847 | 1300 | 418 | 861 | 3941 | 281 | 829 | 1380 |
| PE O-36:2|PE O-18:1_18:1 | 6375 | 4877 | 3723 | 8446 | 7556 | 4314 | 3641 | 5467 | 5550 |
| PE O-36:3|PE O-18:1_18:2 | 1117 | 711 | 797 | 707 | 619 | 969 | 595 | 1649 | 896 |
| PE O-36:3|PE O-18:2_18:1 | 8686 | 5459 | 4588 | 10094 | 7541 | 5811 | 4580 | 7057 | 6727 |
| PE O-36:4|PE O-18:2_18:2 | 3577 | 2899 | 2682 | 5038 | 3472 | 3058 | 2417 | 4066 | 3401 |
| PE O-36:5|PE O-16:1_20:4 | 3471 | 2746 | 2912 | 4155 | 3460 | 2690 | 2688 | 2935 | 3132 |
| PE O-36:5|PE O-18:2_18:3 | 556 | 311 | 340 | 796 | 677 | 391 | 401 | 830 | 538 |
| PE O-36:6|PE O-16:1_20:5 | 1933 | 1061 | 704 | 1628 | 1367 | 1230 | 1028 | 1414 | 1296 |
| PE O-38:4|PE O-18:1_20:3 | 761 | 604 | 489 | 985 | 399 | 629 | 579 | 422 | 608 |
| PE O-38:5|PE O-18:1_20:4 | 1704 | 1162 | 1142 | 2051 | 1751 | 1465 | 1067 | 1387 | 1466 |
| PE O-38:6|PE O-16:1_22:5 | 3344 | 2197 | 3333 | 2380 | 2207 | 2286 | 2737 | 2929 | 2677 |
| PE O-40:6|PE O-18:1_22:5 | 1122 | 743 | 958 | 739 | 934 | 938 | 873 | 576 | 860 |
| PE P-30:1|PE P-14:0_16:1 | 490 | 218 | 264 | 1071 | 787 | 306 | 348 | 441 | 491 |
| PE P-31:0|PE P-15:0_16:0 | 785 | 374 | 476 | 1179 | 704 | 570 | 520 | 517 | 641 |
| PE P-32:1|PE P-14:0_18:1 | 2005 | 1116 | 1304 | 4062 | 3262 | 1310 | 1550 | 1927 | 2067 |
| PE P-32:2|PE P-14:0_18:2 | 625 | 389 | 351 | 1062 | 850 | 590 | 662 | 555 | 635 |
| PE P-33:1|PE P-15:0_18:1 | 1176 | 933 | 1125 | 2395 | 2290 | 1133 | 1986 | 1615 | 1582 |
| PE P-33:2|PE P-15:0_18:2 | 758 | 466 | 497 | 1338 | 1228 | 898 | 1225 | 654 | 883 |
| PE P-33:3|PE P-15:0_18:3 | 374 | 203 | 285 | 862 | 792 | 515 | 593 | 657 | 535 |
| PE P-34:1|PE P-16:0_18:1 | 8092 | 4234 | 4967 | 14755 | 11494 | 6025 | 6023 | 8933 | 8065 |
| PE P-34:2|PE P-16:0_18:2 | 3077 | 1779 | 2007 | 4913 | 4748 | 2917 | 3215 | 3202 | 3232 |
| PE P-34:3|PE P-16:0_18:3 | 636 | 413 | 430 | 1379 | 1074 | 600 | 704 | 1103 | 792 |
| PE P-36:1|PE P-18:0_18:1 | 7635 | 3742 | 4828 | 10480 | 8810 | 4014 | 3674 | 6218 | 6175 |
| PE P-36:2|PE P-18:0_18:2 | 2046 | 1023 | 1435 | 4059 | 2843 | 1858 | 1396 | 2216 | 2109 |
| PE P-36:2|PE P-18:1_18:1 | 1947 | 1004 | 1291 | 3284 | 3015 | 1550 | 1544 | 1709 | 1918 |
| PE P-36:3|PE P-16:0_20:3 | 998 | 614 | 699 | 1884 | 1176 | 825 | 868 | 1269 | 1042 |
| PE P-36:3|PE P-18:1_18:2 | 1023 | 620 | 651 | 1979 | 1388 | 908 | 744 | 990 | 1038 |
| PE P-36:4|PE P-16:0_20:4 | 891 | 540 | 639 | 1127 | 961 | 927 | 877 | 849 | 851 |
| **Phosphatidylinositols** |  |  |  |  |  |  |  |  |  |
|  |  |  |  |  |  |  |  |  |  |

| PI 30:0 | 800 | 414 | 884 | 1968 | 1647 | 1018 | 1106 | 1184 | 1128 |
| --- | --- | --- | --- | --- | --- | --- | --- | --- | --- |
| PI 30:0|PI 14:0_16:0 | 853 | 427 | 880 | 2051 | 1696 | 1060 | 1379 | 1181 | 1191 |
| PI 32:0|PI 16:0_16:0 | 8072 | 4924 | 6403 | 16567 | 12628 | 7215 | 6641 | 9787 | 9030 |
| PI 32:1|PI 14:0_18:1 | 2734 | 1439 | 2034 | 10331 | 7593 | 3330 | 4114 | 4415 | 4499 |
| PI 33:1 | 622 | 368 | 561 | 2000 | 1759 | 561 | 970 | 1433 | 1034 |
| PI 34:0|PI 16:0_18:0 | 6824 | 3882 | 7020 | 13629 | 9525 | 5277 | 6500 | 11919 | 8072 |
| PI 34:1 | 312 | 147 | 337 | 1124 | 928 | 371 | 369 | 538 | 516 |
| PI 34:1|PI 16:0_18:1 | 38445 | 23025 | 28600 | 87769 | 64200 | 28658 | 35427 | 46180 | 44038 |
| PI 34:2|PI 16:1_18:1 | 6226 | 3642 | 3678 | 18871 | 14892 | 5241 | 8815 | 8222 | 8698 |
| PI 34:3 | 222 | 82 | 98 | 1001 | 910 | 175 | 476 | 697 | 458 |
| PI 35:2 | 396 | 198 | 580 | 1467 | 752 | 358 | 990 | 826 | 696 |
| PI 36:0 | 739 | 716 | 1385 | 1450 | 1616 | 878 | 900 | 1199 | 1110 |
| PI 36:1 | 386 | 323 | 311 | 1213 | 624 | 396 | 400 | 763 | 552 |
| PI 36:1|PI 18:0_18:1 | 123549 | 65445 | 93148 | 190139 | 156449 | 69865 | 96296 | 127291 | 115273 |
| PI 36:2 | 697 | 234 | 505 | 1791 | 2016 | 918 | 667 | 1113 | 993 |
| PI 36:2|PI 18:1_18:1 | 56341 | 29529 | 45552 | 104505 | 86048 | 34301 | 60940 | 60638 | 59732 |
| PI 36:3|PI 18:1_18:2 | 9506 | 5422 | 6381 | 21238 | 20163 | 7359 | 13798 | 13540 | 12176 |
| PI 36:4|PI 16:0_20:4 | 2298 | 1623 | 1877 | 7003 | 5956 | 1996 | 3954 | 2527 | 3404 |
| PI 36:4|PI 18:2_18:2 | 320 | 165 | 288 | 1851 | 1762 | 508 | 1037 | 1350 | 910 |
| PI 36:5 | 627 | 667 | 675 | 2100 | 1554 | 1036 | 1354 | 1182 | 1149 |
| PI 37:1 | 1487 | 990 | 1180 | 2319 | 2146 | 1041 | 1246 | 1441 | 1481 |
| PI 37:2 | 1707 | 604 | 848 | 2669 | 2157 | 896 | 1331 | 1217 | 1429 |
| PI 38:1 | 2375 | 1267 | 1483 | 3689 | 3835 | 1225 | 1500 | 1843 | 2152 |
| PI 38:2 | 4566 | 2402 | 3835 | 7256 | 6339 | 2995 | 4033 | 3595 | 4378 |
| PI 38:3|PI 18:0_20:3 | 10405 | 6424 | 9885 | 16103 | 14841 | 9679 | 14990 | 11943 | 11784 |
| PI 38:4|PI 18:0_20:4 | 14963 | 7991 | 16418 | 21162 | 21171 | 11054 | 18541 | 11191 | 15312 |
| PI 38:4|PI 18:1_20:3 | 3736 | 1950 | 3015 | 8834 | 7499 | 4150 | 5600 | 5316 | 5013 |
| PI 38:5|PI 18:0_20:5 | 7521 | 4099 | 6587 | 13178 | 11838 | 6168 | 8169 | 7015 | 8072 |
| PI 38:5|PI 18:1_20:4 | 4843 | 2580 | 4786 | 14093 | 10657 | 4973 | 7157 | 6161 | 6906 |
| PI 38:6 | 1268 | 550 | 778 | 3139 | 2672 | 1168 | 1977 | 1526 | 1635 |
| PI 40:3 | 9586 | 8125 | 11454 | 11715 | 12371 | 7264 | 6914 | 7724 | 9394 |
| PI 40:6 | 303 | 79 | 279 | 633 | 322 | 140 | 441 | 412 | 326 |
| PI 42:10 | 2724 | 1748 | 1949 | 5373 | 4367 | 1616 | 3099 | 3484 | 3045 |
| **Phosphatidylserines** |  |  |  |  |  |  |  |  |  |
| PS 32:0 | 526 | 249 | 299 | 1004 | 911 | 588 | 428 | 489 | 562 |
| PS 32:1|PS 16:0_16:1 | 869 | 557 | 212 | 3140 | 1958 | 1001 | 778 | 472 | 1123 |
| PS 34:1|PS 16:0_18:1 | 22615 | 12907 | 12360 | 42565 | 28332 | 15011 | 14679 | 16556 | 20628 |
| PS 34:2|PS 16:0_18:2 | 6459 | 3842 | 4217 | 12354 | 9175 | 5298 | 5854 | 5179 | 6547 |
| PS 34:3|PS 16:0_18:3 | 248 | 342 | 242 | 1401 | 772 | 367 | 445 | 427 | 530 |
| PS 35:1|PS 18:0_17:1 | 4362 | 2474 | 3389 | 9242 | 7315 | 3263 | 4661 | 4535 | 4905 |
| PS 35:2|PS 17:0_18:2 | 482 | 273 | 416 | 1137 | 741 | 432 | 523 | 671 | 584 |
| PS 36:0|PS 18:0_18:0 | 20538 | 11920 | 19404 | 39738 | 30720 | 14804 | 24305 | 33303 | 24341 |
| PS 36:1|PS 18:0_18:1 | 164107 | 83327 | 150533 | 300140 | 234064 | 106454 | 156165 | 217199 | 176498 |
| PS 36:2|PS 18:1_18:1 | 94595 | 50544 | 70799 | 152582 | 124416 | 59817 | 97326 | 119213 | 96162 |
| PS 36:3|PS 18:0_18:3 | 2851 | 1407 | 1841 | 8171 | 7712 | 3207 | 3703 | 5995 | 4361 |

| PS 36:3|PS 18:1_18:2 | 13598 | 7645 | 12303 | 28411 | 23830 | 10827 | 16893 | 26957 | 17558 |
| --- | --- | --- | --- | --- | --- | --- | --- | --- | --- |
| PS 36:4|PS 18:1_18:3 | 769 | 409 | 800 | 2925 | 1909 | 700 | 1528 | 2411 | 1431 |
| PS 36:4|PS 18:2_18:2 | 210 | 173 | 206 | 585 | 448 | 208 | 277 | 328 | 305 |
| PS 37:0 | 82 | 63 | 79 | 277 | 132 | 68 | 94 | 74 | 109 |
| PS 37:2 | 240 | 119 | 202 | 492 | 376 | 179 | 216 | 356 | 272 |
| PS 38:1|PS 20:0_18:1 | 6029 | 2819 | 6393 | 8838 | 7060 | 4442 | 4789 | 6223 | 5824 |
| PS 38:2|PS 18:1_20:1 | 2458 | 1478 | 2905 | 6327 | 4496 | 2547 | 3480 | 3504 | 3399 |
| PS 38:3 | 21411 | 18326 | 13207 | 39800 | 35249 | 8747 | 18899 | 16164 | 21475 |
| PS 38:3|PS 18:0_20:3 | 13543 | 6596 | 9360 | 26418 | 20772 | 10581 | 16911 | 20203 | 15548 |
| PS 38:4|PS 18:1_20:3 | 3337 | 1449 | 1830 | 4739 | 4071 | 2499 | 3006 | 3948 | 3110 |
| PS 38:5|PS 16:0_22:5 | 2012 | 813 | 972 | 3152 | 1822 | 1675 | 2054 | 1539 | 1755 |
| PS 39:1|PS 21:0_18:1 | 3885 | 2550 | 4341 | 6401 | 5638 | 2711 | 3397 | 4543 | 4183 |
| PS 39:2|PS 18:1_21:1 | 2567 | 1468 | 2746 | 6165 | 4664 | 1941 | 2527 | 3557 | 3204 |
| PS 39:5 | 235 | 99 | 218 | 522 | 417 | 223 | 294 | 354 | 295 |
| PS 40:2 | 2002 | 625 | 1786 | 3786 | 2803 | 1740 | 1513 | 2856 | 2139 |
| PS 40:2|PS 22:0_18:2 | 1624 | 785 | 2140 | 3658 | 2639 | 1710 | 1982 | 2957 | 2187 |
| PS 40:3 | 995 | 936 | 1145 | 2094 | 2358 | 852 | 1599 | 1246 | 1403 |
| PS 40:4|PS 18:0_22:4 | 2741 | 1188 | 3063 | 4978 | 4452 | 1555 | 3117 | 6854 | 3494 |
| PS 40:5|PS 18:0_22:5 | 23829 | 10833 | 24452 | 44453 | 35701 | 16957 | 29626 | 45618 | 28934 |
| PS 40:6|PS 18:1_22:5 | 3070 | 1755 | 2767 | 6557 | 4606 | 1985 | 4762 | 6231 | 3967 |
| PS 41:2 | 611 | 298 | 511 | 1430 | 1378 | 346 | 967 | 982 | 815 |
| PS 42:1|PS 24:0_18:1 | 888 | 105 | 865 | 1406 | 1415 | 641 | 570 | 1412 | 913 |
| **Sulfatides** |  |  |  |  |  |  |  |  |  |
| SHexCer 35:0;3O | 2458 | 986 | 2679 | 4817 | 3470 | 1973 | 2355 | 4126 | 2858 |
| SHexCer 36:4;2O | 3649 | 2894 | 5350 | 8087 | 5773 | 2702 | 1990 | 4281 | 4340 |
| SHexCer 38:2;2O | 328 | 409 | 172 | 500 | 315 | 181 | 327 | 179 | 301 |
| SHexCer 38:7;3O | 447 | 158 | 503 | 940 | 600 | 303 | 347 | 746 | 505 |
| SHexCer 39:5;3O | 1526 | 958 | 2532 | 3719 | 2074 | 859 | 1016 | 2522 | 1901 |
| SHexCer 39:6;3O | 514 | 229 | 482 | 1381 | 891 | 399 | 437 | 783 | 640 |
| SHexCer 39:7;3O | 7351 | 6626 | 10299 | 16968 | 11346 | 5965 | 4918 | 8193 | 8958 |
| SHexCer 39:8;3O | 2840 | 2542 | 3116 | 6726 | 4443 | 2368 | 2378 | 3771 | 3523 |
| SHexCer 40:2;2O | 4698 | 4710 | 3176 | 6298 | 4461 | 2857 | 2722 | 3994 | 4114 |
| SHexCer 40:3;3O | 733 | 770 | 2623 | 3600 | 2561 | 1176 | 1404 | 4652 | 2190 |
| SHexCer 40:8;3O | 388 | 210 | 572 | 578 | 617 | 143 | 407 | 617 | 442 |
| SHexCer 40:9;3O | 1209 | 1062 | 1955 | 2931 | 1871 | 846 | 725 | 1665 | 1533 |
| SHexCer 41:8;3O | 10743 | 10836 | 27438 | 27995 | 23820 | 11111 | 8988 | 19138 | 17509 |
| SHexCer 42:2;2O | 166 | 355 | 362 | 313 | 278 | 159 | 103 | 184 | 240 |
| SHexCer 42:4;3O | 3135 | 2320 | 7833 | 8178 | 5266 | 2488 | 3994 | 10388 | 5450 |
| SHexCer 43:0;3O | 530 | 480 | 1246 | 1923 | 1839 | 361 | 1048 | 611 | 1005 |
| SHexCer 44:2;2O | 2102 | 2572 | 3124 | 2194 | 1935 | 1153 | 1328 | 700 | 1889 |
| SHexCer 46:2;2O | 8378 | 9327 | 9152 | 13174 | 9615 | 5023 | 4817 | 8047 | 8442 |
| **Sphingomyelins** |  |  |  |  |  |  |  |  |  |
| SM 30:0;2O|SM 22:0;2O/8:0 | 952 | 776 | 478 | 970 | 909 | 605 | 687 | 626 | 750 |
| SM 30:1;2O|SM 16:1;2O/14:0 | 996 | 829 | 851 | 1223 | 1253 | 610 | 1024 | 871 | 957 |
| SM 31:0;2O|SM 19:0;2O/12:0 | 312 | 192 | 146 | 405 | 317 | 211 | 203 | 257 | 255 |
| SM 31:1;2O|SM 17:1;2O/14:0 | 940 | 584 | 736 | 1038 | 1175 | 632 | 832 | 733 | 834 |

| SM 32:0;2O | 820 | 592 | 486 | 1292 | 1098 | 730 | 698 | 963 | 835 |
| --- | --- | --- | --- | --- | --- | --- | --- | --- | --- |
| SM 32:0;2O|SM 22:0;2O/10:0 | 24112 | 17845 | 11589 | 41309 | 37874 | 20722 | 25986 | 28793 | 26029 |
| SM 32:1;2O | 396 | 194 | 535 | 506 | 528 | 362 | 421 | 644 | 448 |
| SM 32:1;2O|SM 16:1;2O/16:0 | 37998 | 26688 | 29165 | 72416 | 68270 | 37093 | 62107 | 53293 | 48379 |
| SM 33:0;2O | 3271 | 1869 | 1627 | 5795 | 5707 | 2401 | 2349 | 3997 | 3377 |
| SM 33:1;2O | 506 | 415 | 372 | 1116 | 1044 | 480 | 605 | 813 | 669 |
| SM 33:1;2O|SM 17:1;2O/16:0 | 20771 | 13544 | 15084 | 50206 | 42792 | 21236 | 34034 | 33420 | 28886 |
| SM 34:0;2O | 23075 | 20627 | 13879 | 78053 | 58202 | 21025 | 17122 | 55856 | 35980 |
| SM 34:1;2O | 5636 | 4663 | 5203 | 25713 | 22301 | 13763 | 17961 | 27883 | 15391 |
| SM 34:1;2O | 1599 | 1551 | 2681 | 6103 | 4148 | 1770 | 2221 | 5616 | 3211 |
| SM 34:1;2O|SM 18:1;2O/16:0 | 220256 | 172711 | 205719 | 734261 | 641540 | 331549 | 388778 | 603733 | 412318 |
| SM 34:1;3O | 353 | 227 | 310 | 1792 | 1582 | 579 | 474 | 1124 | 805 |
| SM 34:2;2O|SM 25:2;2O/9:0 | 2098 | 1288 | 1912 | 6070 | 5292 | 2459 | 3045 | 5394 | 3445 |
| SM 35:0;2O | 590 | 403 | 439 | 1399 | 1038 | 539 | 533 | 824 | 720 |
| SM 35:1;2O|SM 16:1;2O/19:0 | 5093 | 2656 | 4037 | 10873 | 10217 | 5438 | 5988 | 6823 | 6391 |
| SM 35:1;2O|SM 18:1;2O/17:0 | 2835 | 2110 | 3091 | 5288 | 4631 | 2427 | 2723 | 4234 | 3417 |
| SM 35:2;2O | 598 | 411 | 427 | 894 | 715 | 350 | 408 | 544 | 543 |
| SM 35:2;2O|SM 21:1;2O/14:1 | 330 | 235 | 220 | 592 | 484 | 267 | 312 | 512 | 369 |
| SM 35:2;3O | 1323 | 862 | 867 | 2377 | 2150 | 1089 | 1157 | 1230 | 1382 |
| SM 36:0;2O | 2163 | 1475 | 1594 | 3456 | 2202 | 1131 | 1513 | 2321 | 1982 |
| SM 36:1;2O|SM 15:0;2O/21:1 | 7050 | 5681 | 8910 | 19203 | 17211 | 9336 | 12926 | 15397 | 11964 |
| SM 36:2;2O|SM 19:1;2O/17:1 | 1872 | 823 | 1507 | 4005 | 3526 | 2023 | 2271 | 3248 | 2410 |
| SM 37:0;2O | 1541 | 1132 | 1115 | 2153 | 1884 | 1016 | 1147 | 1209 | 1400 |
| SM 37:1;2O | 815 | 481 | 948 | 1276 | 1091 | 674 | 718 | 998 | 875 |
| SM 38:0;2O|SM 16:0;2O/22:0 | 16863 | 14259 | 11966 | 19582 | 15508 | 6805 | 7394 | 11309 | 12961 |
| SM 38:1;2O|SM 16:1;2O/22:0 | 66488 | 56193 | 77355 | 105935 | 109641 | 59644 | 92133 | 62140 | 78691 |
| SM 38:2;2O|SM 19:2;2O/19:0 | 754 | 562 | 632 | 1187 | 1026 | 555 | 785 | 762 | 783 |
| SM 39:0;2O | 34002 | 36683 | 30166 | 38573 | 34923 | 21825 | 23507 | 25914 | 30699 |
| SM 39:1;2O|SM 17:1;2O/22:0 | 1410 | 1182 | 1206 | 2808 | 2232 | 1356 | 1052 | 1945 | 1649 |
| SM 39:1;3O | 4027 | 4186 | 4634 | 10011 | 7776 | 4749 | 5282 | 5375 | 5755 |
| SM 39:2;2O|SM 19:1;2O/20:1 | 1144 | 919 | 1314 | 1557 | 1838 | 800 | 1211 | 1439 | 1278 |
| SM 40:0;2O | 14217 | 17026 | 13651 | 20143 | 17134 | 6928 | 6549 | 12387 | 13505 |
| SM 40:1;2O | 2550 | 2520 | 2810 | 4489 | 3857 | 2131 | 2758 | 3537 | 3082 |
| SM 40:1;2O|SM 16:1;2O/24:0 | 167209 | 151043 | 181391 | 285602 | 270145 | 148173 | 176497 | 223287 | 200418 |
| SM 40:1;2O|SM 18:1;2O/22:0 | 2573 | 2431 | 1966 | 3131 | 2676 | 2237 | 1411 | 1925 | 2294 |
| SM 40:1;3O | 577 | 463 | 1027 | 1178 | 1745 | 828 | 509 | 1489 | 977 |
| SM 40:2;2O|SM 18:1;2O/22:1 | 8114 | 6865 | 8454 | 13505 | 13852 | 9005 | 12930 | 7792 | 10065 |
| SM 40:8;2O | 1179 | 814 | 1233 | 1943 | 1360 | 715 | 767 | 1159 | 1146 |
| SM 41:0;2O | 4090 | 5080 | 4219 | 8101 | 5847 | 2108 | 3034 | 5750 | 4779 |
| SM 41:1;2O|SM 18:1;2O/23:0 | 31762 | 36608 | 46640 | 57157 | 46276 | 20869 | 26978 | 57356 | 40456 |
| SM 41:2;2O|SM 18:1;2O/23:1 | 3724 | 3390 | 5007 | 8572 | 7273 | 4403 | 4245 | 8620 | 5654 |
| SM 42:1;2O|SM 18:1;2O/24:0 | 45980 | 58585 | 60877 | 71869 | 67974 | 33758 | 39163 | 80874 | 57385 |
| SM 42:2;2O | 226 | 289 | 306 | 429 | 438 | 291 | 253 | 462 | 337 |
| SM 42:2;2O|SM 18:1;2O/24:1 | 9075 | 8208 | 10068 | 20265 | 16848 | 10887 | 10556 | 16088 | 12750 |
| SM 42:2;3O | 244 | 153 | 260 | 431 | 330 | 212 | 295 | 376 | 288 |
| SM 42:3;2O | 493 | 385 | 586 | 1057 | 926 | 606 | 542 | 712 | 663 |

| SM 43:2;2O|SM 18:1;2O/25:1 | 1147 | 1029 | 913 | 2120 | 1562 | 507 | 358 | 1155 | 1099 |
| --- | --- | --- | --- | --- | --- | --- | --- | --- | --- |
| SM 44:8;2O | 248 | 200 | 222 | 733 | 267 | 271 | 96 | 160 | 275 |
| **Triacylglycerols** |  |  |  |  |  |  |  |  |  |
| TG 46:0|TG 14:0_16:0_16:0 | 32408 | 32153 | 34655 | 40377 | 49479 | 24375 | 17251 | 36352 | 33381 |
| TG 48:1|TG 16:0_16:0_16:1 | 81837 | 61580 | 77080 | 151543 | 100176 | 74259 | 60970 | 104389 | 88979 |
| TG 49:3|TG 15:0_16:0_18:3 | 18337 | 7976 | 14944 | 33509 | 58675 | 26187 | 19403 | 34916 | 26743 |
| TG 50:2|TG 16:0_16:0_18:2 | 41487 | 68970 | 109977 | 109865 | 173657 | 70998 | 45505 | 67351 | 85976 |
| TG 51:2|TG 15:0_18:1_18:1 | 15012 | 10591 | 16483 | 24469 | 23810 | 14646 | 6223 | 12466 | 15462 |
| TG 51:4|TG 15:0_16:0_20:4 | 5781 | 6018 | 12045 | 20699 | 32530 | 15817 | 12222 | 16382 | 15187 |
| TG 52:2|TG 16:0_18:1_18:1 | 25723 | 41822 | 80834 | 83836 | 93456 | 44143 | 32984 | 50646 | 56681 |
| TG 52:3|TG 16:0_18:1_18:2 | 1423209 | 1160154 | 1455519 | 2784130 | 3609413 | 1416177 | 725808 | 1461405 | 1754477 |
| TG 53:4|TG 17:1_18:1_18:2 | 6327 | 5820 | 11358 | 21443 | 24298 | 8086 | 9420 | 9971 | 12090 |
| TG 54:6|TG 18:1_18:2_18:3 | 14818 | 8732 | 20428 | 24897 | 52427 | 18871 | 17121 | 33812 | 23888 |
| TG 56:6|TG 16:0_18:1_22:5 | 7637 | 12759 | 29541 | 27539 | 55232 | 30070 | 17536 | 28144 | 26057 |
| TG 57:6|TG 17:0_18:1_22:5 | 706 | 681 | 1117 | 1271 | 1178 | 1097 | 597 | 1038 | 961 |
| TG 10:0_10:0_20:5 | 73961 | 29532 | 38192 | 113386 | 97329 | 60220 | 34671 | 63816 | 63889 |
| TG 8:0_8:0_16:4 | 1567 | 580 | 926 | 2725 | 2735 | 812 | 656 | 1734 | 1467 |
| TG O-42:5|TG O-20:5_11:0_11:0 | 2379 | 3125 | 2764 | 1976 | 2141 | 1597 | 598 | 1906 | 2061 |
| TG O-49:6|TG O-15:4_17:1_17:1 | 5944 | 7051 | 8416 | 7898 | 9062 | 4552 | 3258 | 6718 | 6612 |
| TG O-51:6|TG O-15:4_17:0_19:2 | 3000 | 3865 | 4707 | 3460 | 4250 | 2455 | 1647 | 3283 | 3333 |
| TG O-51:7|TG O-15:4_15:0_21:3 | 9481 | 9448 | 9968 | 17993 | 11409 | 7264 | 6978 | 12708 | 10656 |
| TG O-52:7|TG O-18:4_16:0_18:3 | 2823 | 2796 | 3488 | 4005 | 4241 | 2796 | 1241 | 2537 | 2991 |
| TG O-54:8|TG O-15:4_18:1_21:3 | 2259 | 2395 | 3025 | 3313 | 2910 | 2270 | 996 | 2245 | 2427 |
| TG O-55:9|TG O-13:1_21:4_21:4 | 3679 | 4588 | 5507 | 7018 | 10139 | 3522 | 1973 | 4199 | 5078 |
| TG O-57:10|TG O-15:0_21:5_21:5 | 1844 | 2252 | 3118 | 2441 | 2281 | 1387 | 1042 | 1753 | 2015 |
| TG O-57:9|TG O-15:1_21:4_21:4 | 1907 | 2072 | 2833 | 2532 | 2860 | 1265 | 1178 | 1395 | 2005 |
| **Others** |  |  |  |  |  |  |  |  |  |
| CoQ8 | 734 | 710 | 536 | 2148 | 1869 | 640 | 512 | 1649 | 1100 |
| DGCC 17:2_22:6 | 1419 | 1118 | 1959 | 2735 | 2291 | 1250 | 2082 | 3696 | 2069 |
| DGGA 36:2|DGGA 18:1_18:1 | 1648 | 1423 | 3594 | 6025 | 4740 | 2263 | 1703 | 4945 | 3293 |
| DGTS 21:0 | 749 | 347 | 586 | 1090 | 748 | 369 | 394 | 477 | 595 |
| LPA 16:3 | 2801 | 2069 | 2492 | 3092 | 2708 | 1863 | 1323 | 2374 | 2340 |
| LPE O-19:4 | 1082 | 879 | 1118 | 1550 | 1226 | 565 | 498 | 1008 | 991 |
| LPE-N (FA)33:2|LPE-N (FA 15:0)18:2 | 1137 | 1144 | 731 | 1970 | 1475 | 919 | 971 | 1034 | 1173 |
| LPG O-13:0 | 1302 | 741 | 932 | 1315 | 1094 | 813 | 917 | 1140 | 1032 |
| NAGly 9:0;O | 904 | 1887 | 1402 | 1625 | 1803 | 852 | 747 | 1191 | 1302 |
| Pentaerythritol tetrakis(3,5-di-tert-butyl-4-hydro | 21777 | 15589 | 31447 | 23815 | 21289 | 12390 | 9772 | 15648 | 18966 |
| PI O-16:0 | 1457 | 1137 | 697 | 2568 | 3327 | 1207 | 4801 | 1821 | 2127 |
| PI-Cer 38:3;2O | 437 | 249 | 388 | 1019 | 924 | 477 | 823 | 826 | 643 |
| PMeOH 40:4|PMeOH 20:2_20:2 | 942 | 101 | 146 | 1335 | 996 | 422 | 429 | 527 | 612 |
| SL 12:0;O/26:0 | 6661 | 3747 | 3042 | 6930 | 5808 | 3645 | 4437 | 7007 | 5160 |
| SL 12:1;O/32:0 | 9158 | 4981 | 5220 | 6436 | 7887 | 2954 | 3726 | 8303 | 6083 |
| ST 27:1;O;S - Cholesterol sulfate | 709 | 681 | 1905 | 2781 | 1810 | 985 | 801 | 1531 | 1400 |

| **Control T21**  **Metabolyte name** | **t21_C1** | **t21_C2** | **t21_C3** | **t21_C4** |  | **t21_C5** |  | **t21_C6** | **t21_C7** | **t21_C8** | **average value** |
| --- | --- | --- | --- | --- | --- | --- | --- | --- | --- | --- | --- |
| **Acyl sterol glycosides** |  |  |  |  |  |  |  |  |  |  |  |
| ASG 27:1;O;Hex;FA 2:0 | 974 | 611 | 443 |  | 632 |  | 198 | 298 | 1033 | 956 | 643 |
| ASG 28:1;O;Hex;FA 14:0 | 382 | 16 | 2402 |  | 1781 |  | 558 | 392 | 2306 | 2662 | 1312 |
| ASG 28:1;O;Hex;FA 16:0 | 314 | 14 | 1418 |  | 1339 |  | 548 | 318 | 1498 | 1714 | 895 |
| ASG 28:1;O;Hex;FA 18:0 | 260 | 15 | 1216 |  | 1180 |  | 857 | 205 | 921 | 1508 | 770 |
| ASG 28:1;O;Hex;FA 20:1 | 404 | 29 | 1170 |  | 822 |  | 632 | 246 | 1071 | 1428 | 725 |
| ASG 28:2;O;Hex;FA 14:0 | 1021 | 16 | 4152 |  | 2472 |  | 1448 | 794 | 3535 | 4100 | 2192 |
| ASG 28:2;O;Hex;FA 16:0 | 394 | 14 | 1964 |  | 1360 |  | 509 | 387 | 1989 | 1894 | 1064 |
| ASG 28:2;O;Hex;FA 16:1 | 295 | 16 | 1227 |  | 758 |  | 401 | 206 | 1115 | 987 | 626 |
| ASG 28:2;O;Hex;FA 20:1 | 221 | 34 | 689 |  | 547 |  | 301 | 132 | 733 | 777 | 429 |
| ASG 29:2;O;Hex;FA 20:5 | 660 | 262 | 1135 |  | 863 |  | 1737 | 466 | 1087 | 1482 | 961 |
| **Carnitines** |  |  |  |  |  |  |  |  |  |  |  |
| CAR 13:0 | 1064 | 1175 | 537 |  | 596 |  | 612 | 323 | 421 | 958 | 711 |
| CAR 4:0 | 4134 | 7727 | 17601 |  | 2852 |  | 4588 | 4523 | 5512 | 11402 | 7292 |
| CAR 5:0 | 2865 | 4495 | 21798 |  | 3567 |  | 5171 | 4331 | 3618 | 13863 | 7464 |
| CAR 21:2 | 3110 | 1632 | 6108 |  | 2875 |  | 1491 | 1451 | 1853 | 3852 | 2797 |
| CAR 21:4 | 2802 | 5520 | 10184 |  | 2211 |  | 518 | 5136 | 3014 | 3947 | 4166 |
| **Ceramides** |  |  |  |  |  |  |  |  |  |  |  |
| Cer 20:2;4O|Cer 12:2;3O/8:0;(2OH) | 3418 | 2465 | 1786 |  | 321 |  | 1094 | 1056 | 811 | 727 | 1460 |
| Cer 24:3;3O|Cer 15:3;2O/9:0;O | 2464 | 1945 | 2182 |  | 1577 |  | 491 | 2677 | 1863 | 1617 | 1852 |
| Cer 32:0;2O|Cer 16:0;2O/16:0 | 641 | 555 | 864 |  | 437 |  | 168 | 293 | 519 | 432 | 489 |
| Cer 32:1;2O|Cer 16:1;2O/16:0 | 580 | 287 | 720 |  | 456 |  | 226 | 479 | 720 | 641 | 513 |
| Cer 33:1;2O|Cer 17:1;2O/16:0 | 220 | 142 | 318 |  | 172 |  | 86 | 78 | 216 | 305 | 192 |
| Cer 33:1;4O|Cer 18:0;3O/15:1;(2OH) | 2071 | 1439 | 3267 |  | 1324 |  | 921 | 1163 | 1227 | 2273 | 1711 |
| Cer 34:1;2O|Cer 18:1;2O/16:0 | 2290 | 1331 | 2511 |  | 2160 |  | 1168 | 1674 | 2167 | 4572 | 2234 |
| Cer 34:1;3O|Cer 19:0;2O/15:1;O | 2196 | 1946 | 2274 |  | 1809 |  | 784 | 1632 | 1388 | 1923 | 1744 |
| Cer 34:3;2O|Cer 12:2;2O/22:1 | 1518 | 883 | 1579 |  | 517 |  | 948 | 380 | 535 | 1678 | 1005 |
| Cer 36:0;3O|Cer 18:0;2O/18:0;O | 14623 | 14092 | 14121 |  | 9952 |  | 3672 | 10736 | 8547 | 10704 | 10806 |
| Cer 36:1;3O|Cer 19:0;2O/17:1;O | 623 | 567 | 658 |  | 548 |  | 186 | 468 | 395 | 530 | 497 |
| Cer 36:3;4O|Cer 19:2;3O/17:1;(2OH) | 1440 | 1420 | 2315 |  | 2086 |  | 330 | 1905 | 1234 | 1692 | 1553 |
| Cer 37:0;2O|Cer 19:0;2O/18:0 | 2557 | 2810 | 1038 |  | 436 |  | 549 | 251 | 525 | 787 | 1119 |
| Cer 38:0;4O | 1514 | 6628 | 729 |  | 174 |  | 414 | 229 | 380 | 842 | 1364 |
| Cer 39:0;3O|Cer 18:0;2O/21:0;O | 6246 | 4914 | 2833 |  | 2086 |  | 1793 | 1392 | 1609 | 3869 | 3093 |
| Cer 40:0;2O|Cer 16:0;2O/24:0 | 984 | 828 | 1578 |  | 810 |  | 352 | 320 | 776 | 368 | 752 |
| Cer 40:1;2O|Cer 16:1;2O/24:0 | 2111 | 1591 | 4239 |  | 2245 |  | 806 | 1398 | 2629 | 1833 | 2107 |
| Cer 41:1;2O|Cer 18:1;2O/23:0 | 1102 | 746 | 1538 |  | 2170 |  | 542 | 540 | 1398 | 916 | 1119 |
| Cer 42:1;2O|Cer 18:1;2O/24:0 | 1605 | 1106 | 2805 |  | 4031 |  | 708 | 1654 | 2520 | 2372 | 2100 |
| Cer 44:2;4O|Cer 28:1;3O(FA 16:0) | 760 | 192 | 1919 |  | 1944 |  | 1604 | 663 | 2367 | 2239 | 1461 |
| Cer 12:0;2O/25:0 | 3990 | 1909 | 8294 |  | 3053 |  | 1347 | 2590 | 2063 | 3908 | 3394 |
| Cer 12:2;2O/19:4 | 634 | 419 | 3370 |  | 880 |  | 276 | 501 | 1294 | 1233 | 1076 |
| Cer 13:2;2O/30:3 | 2727 | 1569 | 4784 |  | 1833 |  | 869 | 2815 | 3245 | 2552 | 2549 |
| **Diacylglycerols** |  |  |  |  |  |  |  |  |  |  |  |
| DG 16:0 | 1054 | 769 | 2748 |  | 950 |  | 379 | 597 | 1023 | 1459 | 1122 |

| DG 18:0 | 3491 | 2276 | 8218 | 3572 | 1109 | 2948 | 3923 | 4591 | 3766 |
| --- | --- | --- | --- | --- | --- | --- | --- | --- | --- |
| DG 20:0 | 11105 | 6107 | 47527 | 40306 | 4688 | 26846 | 20974 | 41243 | 24849 |
| DG 21:0 | 398 | 373 | 1578 | 535 | 226 | 351 | 421 | 828 | 589 |
| DG 22:0 | 5242 | 3209 | 28536 | 24038 | 3403 | 11416 | 12148 | 29430 | 14678 |
| DG 22:0|DG 10:0_12:0 | 459 | 315 | 2117 | 1525 | 329 | 666 | 950 | 1652 | 1002 |
| DG 22:0|DG 8:0_14:0 | 494 | 313 | 2244 | 1641 | 309 | 725 | 926 | 1627 | 1035 |
| DG 22:1 | 2342 | 2084 | 14003 | 5729 | 1416 | 4245 | 3257 | 8291 | 5171 |
| DG 22:1|DG 8:0_14:1 | 3213 | 1793 | 15814 | 6461 | 1622 | 4599 | 3475 | 6683 | 5457 |
| DG 24:0 | 3762 | 2230 | 23530 | 10406 | 3312 | 6292 | 8848 | 17492 | 9484 |
| DG 24:0|DG 10:0_14:0 | 1519 | 975 | 10313 | 5497 | 1345 | 2507 | 4856 | 7360 | 4296 |
| DG 24:0|DG 8:0_16:0 | 1604 | 1014 | 8579 | 6172 | 1176 | 2498 | 4105 | 8343 | 4186 |
| DG 24:1 | 3496 | 2162 | 29985 | 14666 | 2418 | 7627 | 8702 | 15049 | 10513 |
| DG 24:1|DG 10:0_14:1 | 2110 | 1175 | 16263 | 7421 | 1245 | 4101 | 4861 | 7739 | 5614 |
| DG 24:2 | 337 | 256 | 1544 | 842 | 254 | 393 | 500 | 1074 | 650 |
| DG 24:3 | 236 | 167 | 749 | 369 | 117 | 195 | 240 | 563 | 329 |
| DG 25:0|DG 10:0_15:0 | 554 | 228 | 2869 | 870 | 255 | 462 | 1214 | 1385 | 979 |
| DG 26:0|DG 12:0_14:0 | 4378 | 3076 | 18508 | 10613 | 4349 | 5953 | 8244 | 18451 | 9197 |
| DG 26:1 | 3512 | 1855 | 20672 | 9075 | 2338 | 4796 | 5611 | 13294 | 7644 |
| DG 26:1|DG 8:0_18:1 | 2556 | 1207 | 21215 | 9707 | 1678 | 4381 | 5721 | 11262 | 7216 |
| DG 26:2 | 331 | 257 | 1249 | 791 | 299 | 378 | 493 | 974 | 596 |
| DG 26:2|DG 8:0_18:2 | 336 | 222 | 1179 | 877 | 239 | 378 | 533 | 999 | 595 |
| DG 26:3 | 141 | 105 | 868 | 416 | 92 | 167 | 309 | 845 | 368 |
| DG 27:0 | 722 | 518 | 1802 | 483 | 406 | 303 | 546 | 1197 | 747 |
| DG 27:5 | 695 | 476 | 2496 | 733 | 346 | 463 | 1061 | 1168 | 930 |
| DG 28:0|DG 12:0_16:0 | 14357 | 8607 | 41304 | 21774 | 12124 | 14928 | 16444 | 37902 | 20930 |
| DG 28:1 | 6163 | 3759 | 14835 | 3877 | 4876 | 3266 | 3712 | 12197 | 6585 |
| DG 28:1|DG 10:0_18:1 | 7388 | 3497 | 44145 | 16849 | 6518 | 9355 | 13362 | 31327 | 16555 |
| DG 28:2 | 3863 | 2074 | 6488 | 2425 | 1756 | 1217 | 1754 | 4488 | 3008 |
| DG 28:2|DG 14:1_14:1 | 1888 | 1453 | 9218 | 6107 | 1854 | 2445 | 3077 | 7931 | 4246 |
| DG 28:3 | 235 | 162 | 940 | 490 | 207 | 297 | 397 | 1030 | 470 |
| DG 28:3|DG 10:0_18:3 | 241 | 139 | 1212 | 646 | 182 | 381 | 523 | 1218 | 568 |
| DG 30:0 | 420 | 262 | 908 | 475 | 250 | 280 | 339 | 1134 | 508 |
| DG 30:0|DG 14:0_16:0 | 47314 | 33359 | 89953 | 61742 | 37423 | 41712 | 34142 | 84498 | 53768 |
| DG 30:1|DG 16:0_14:1 | 19316 | 9472 | 76150 | 42753 | 22218 | 24915 | 20864 | 66103 | 35224 |
| DG 30:2 | 1376 | 771 | 2713 | 849 | 984 | 522 | 621 | 2119 | 1244 |
| DG 30:2|DG 12:0_18:2 | 1633 | 848 | 7164 | 4397 | 1526 | 1576 | 2501 | 5564 | 3151 |
| DG 30:2|DG 14:1_16:1 | 1603 | 903 | 7180 | 5450 | 1418 | 1814 | 2427 | 6479 | 3409 |
| DG 30:3|DG 12:0_18:3 | 426 | 258 | 1801 | 886 | 446 | 489 | 636 | 2138 | 885 |
| DG 30:5 | 912 | 582 | 314 | 75 | 149 | 48 | 71 | 76 | 278 |
| DG 30:6 | 4522 | 2688 | 9060 | 1595 | 1662 | 1192 | 2821 | 4423 | 3496 |
| DG 30:7 | 586 | 380 | 1212 | 335 | 208 | 146 | 363 | 618 | 481 |
| DG 31:7 | 38061 | 23836 | 31572 | 7520 | 13997 | 8759 | 14296 | 28027 | 20759 |
| DG 31:8 | 1605 | 829 | 3338 | 1074 | 613 | 524 | 1130 | 1576 | 1336 |
| DG 32:1|DG 14:0_18:1 | 68966 | 32678 | 167829 | 109494 | 36088 | 70389 | 43045 | 125607 | 81762 |
| DG 32:2 | 5176 | 3550 | 6058 | 2810 | 3814 | 2303 | 2005 | 5189 | 3863 |
| DG 32:2|DG 14:1_18:1 | 10240 | 4258 | 34117 | 24429 | 7648 | 10138 | 12016 | 29285 | 16517 |

| DG 32:3 | 864 | 698 | 750 | 285 | 601 | 318 | 268 | 818 | 575 |
| --- | --- | --- | --- | --- | --- | --- | --- | --- | --- |
| DG 32:3|DG 14:0_18:3 | 1467 | 900 | 4157 | 2128 | 1290 | 1184 | 1570 | 4493 | 2149 |
| DG 32:6 | 6483 | 5162 | 7571 | 1962 | 2664 | 1511 | 3059 | 4339 | 4094 |
| DG 32:7 | 1866 | 1218 | 1964 | 506 | 630 | 385 | 633 | 1270 | 1059 |
| DG 33:2 | 852 | 887 | 2753 | 1205 | 484 | 1951 | 2746 | 2285 | 1645 |
| DG 33:3 | 482 | 562 | 2308 | 744 | 228 | 1306 | 1509 | 1179 | 1040 |
| DG 33:6 | 67345 | 70521 | 41435 | 9826 | 25341 | 13270 | 17415 | 29135 | 34286 |
| DG 33:8 | 4725 | 2709 | 5422 | 2223 | 2101 | 1324 | 2988 | 5800 | 3412 |
| DG 33:9 | 623 | 340 | 1017 | 145 | 313 | 141 | 392 | 912 | 485 |
| DG 34:0 | 506 | 176 | 1592 | 321 | 153 | 309 | 475 | 450 | 498 |
| DG 34:0|DG 16:0_18:0 | 6937 | 6794 | 17353 | 7629 | 4894 | 3807 | 4203 | 8273 | 7486 |
| DG 34:1|DG 16:0_18:1 | 105272 | 66161 | 221934 | 129121 | 69244 | 111383 | 66710 | 178124 | 118494 |
| DG 34:2 | 399 | 412 | 268 | 106 | 152 | 158 | 119 | 174 | 224 |
| DG 34:2|DG 16:0_18:2 | 27142 | 9723 | 84616 | 56607 | 23035 | 30035 | 29011 | 71948 | 41515 |
| DG 34:3|DG 16:0_18:3 | 3743 | 1796 | 8832 | 5743 | 3411 | 4039 | 3776 | 14506 | 5731 |
| DG 34:6 | 7440 | 5222 | 8776 | 2066 | 2477 | 2043 | 3350 | 5310 | 4586 |
| DG 34:7 | 3387 | 2454 | 5189 | 1159 | 1357 | 935 | 1304 | 2777 | 2320 |
| DG 35:2 | 713 | 537 | 1916 | 605 | 287 | 868 | 1849 | 1498 | 1034 |
| DG 35:3 | 383 | 325 | 1271 | 452 | 251 | 541 | 793 | 711 | 591 |
| DG 35:8 | 18571 | 13204 | 14252 | 6466 | 8366 | 4608 | 5891 | 13423 | 10598 |
| DG 35:9 | 3148 | 2174 | 3409 | 748 | 1439 | 477 | 1335 | 3368 | 2012 |
| DG 36:1|DG 18:0_18:1 | 8146 | 6109 | 37210 | 4520 | 3950 | 5732 | 4525 | 10366 | 10070 |
| DG 36:2|DG 18:1_18:1 | 37390 | 19297 | 120136 | 32407 | 18935 | 35597 | 22281 | 46463 | 41563 |
| DG 36:3|DG 18:1_18:2 | 10943 | 4688 | 49845 | 16275 | 6695 | 10918 | 11136 | 22608 | 16638 |
| DG 36:4|DG 18:1_18:3 | 2498 | 1537 | 6964 | 2500 | 1265 | 1403 | 1623 | 5608 | 2925 |
| DG 36:5|DG 16:0_20:5 | 363 | 178 | 1141 | 667 | 395 | 494 | 433 | 1375 | 631 |
| DG 36:5|DG 18:2_18:3 | 552 | 243 | 1779 | 841 | 331 | 471 | 737 | 1639 | 824 |
| DG 36:7 | 6139 | 4144 | 5289 | 1590 | 2451 | 1511 | 2021 | 3960 | 3388 |
| DG 36:8 | 398 | 398 | 794 | 182 | 192 | 332 | 522 | 490 | 414 |
| DG 37:9 | 4800 | 3404 | 6626 | 1842 | 2646 | 1402 | 2295 | 6570 | 3698 |
| DG 38:5|DG 16:0_22:5 | 3346 | 1585 | 13179 | 5247 | 2150 | 4548 | 4214 | 13677 | 5993 |
| DG 38:6 | 347 | 265 | 414 | 90 | 124 | 59 | 130 | 255 | 211 |
| DG 40:6|DG 18:1_22:5 | 1063 | 575 | 3252 | 1094 | 549 | 773 | 854 | 2135 | 1287 |
| DG 40:8 | 688 | 647 | 746 | 204 | 296 | 340 | 410 | 480 | 476 |
| DG 41:10 | 1091 | 813 | 695 | 253 | 630 | 294 | 288 | 756 | 603 |
| DG 41:11 | 552 | 424 | 488 | 182 | 262 | 170 | 178 | 354 | 326 |
| DG 41:5 | 3049 | 1922 | 2285 | 1318 | 1543 | 906 | 1056 | 1768 | 1731 |
| DG 41:6 | 22616 | 34373 | 19865 | 5633 | 6655 | 5726 | 8703 | 16980 | 15069 |
| DG 43:11 | 1165 | 1127 | 804 | 363 | 459 | 408 | 344 | 611 | 660 |
| DG 43:3 | 762 | 724 | 537 | 200 | 336 | 181 | 256 | 462 | 432 |
| DG 43:4 | 2648 | 3084 | 2548 | 690 | 1087 | 865 | 1076 | 1996 | 1749 |
| DG 43:6 | 17187 | 21982 | 15571 | 8867 | 6361 | 6650 | 4932 | 5376 | 10866 |
| DG 43:7 | 12678 | 16091 | 21492 | 6621 | 3637 | 8795 | 9866 | 13432 | 11576 |
| DG 44:7 | 898 | 586 | 2913 | 1116 | 385 | 1021 | 1177 | 1323 | 1177 |
| DG 44:9 | 722 | 461 | 2556 | 1376 | 562 | 2171 | 2013 | 4689 | 1819 |
| DG 46:6 | 4800 | 5039 | 4212 | 3598 | 3948 | 5121 | 4665 | 4633 | 4502 |

| DG 47:7 | 8896 | 11099 | 11856 | 5657 | 2579 | 4730 | 6194 | 7601 | 7326 |
| --- | --- | --- | --- | --- | --- | --- | --- | --- | --- |
| DG 47:8 | 8785 | 8567 | 7586 | 3755 | 3219 | 3561 | 4331 | 3799 | 5450 |
| DG 51:7 | 2931 | 2259 | 4186 | 2141 | 1092 | 1423 | 1703 | 2506 | 2280 |
| DG 51:8 | 1534 | 948 | 2015 | 1158 | 622 | 840 | 1018 | 1516 | 1206 |
| **Free fatty Acids** |  |  |  |  |  |  |  |  |  |
| FA 12:0 | 861 | 735 | 1251 | 1869 | 554 | 1159 | 893 | 1380 | 1088 |
| FA 14:0 | 7742 | 4402 | 10044 | 34590 | 2423 | 21535 | 13727 | 11194 | 13207 |
| FA 14:1 | 790 | 163 | 1415 | 5407 | 506 | 2458 | 2404 | 2796 | 1992 |
| FA 15:0 | 1078 | 453 | 1065 | 1470 | 548 | 1002 | 1041 | 769 | 928 |
| FA 15:4 | 2520 | 2020 | 1355 | 1347 | 3127 | 876 | 1549 | 1945 | 1842 |
| FA 16:0 | 761617 | 488670 | 748165 | 1536539 | 283432 | 855251 | 763680 | 684124 | 765184 |
| FA 16:0;(2OH) | 597 | 301 | 729 | 1446 | 443 | 965 | 738 | 921 | 767 |
| FA 16:0;O | 2738 | 1811 | 3244 | 6872 | 1790 | 3587 | 3940 | 3251 | 3404 |
| FA 16:1 | 5796 | 1437 | 8702 | 39527 | 4960 | 22420 | 19590 | 13889 | 14540 |
| FA 16:1;O | 3322 | 2219 | 3455 | 7499 | 2868 | 3617 | 3911 | 3581 | 3809 |
| FA 17:0 | 1886 | 1095 | 2323 | 4640 | 958 | 2248 | 2308 | 1683 | 2143 |
| FA 17:1 | 214 | 15 | 359 | 370 | 141 | 132 | 314 | 243 | 223 |
| FA 17:4;O | 887 | 835 | 933 | 2795 | 743 | 862 | 1042 | 983 | 1135 |
| FA 18:0 | 848913 | 538053 | 914526 | 1777544 | 332347 | 857072 | 739597 | 646865 | 831865 |
| FA 18:0;(2OH) | 3595 | 1711 | 1946 | 4923 | 2040 | 3364 | 3736 | 2626 | 2993 |
| FA 18:0;O | 2765 | 1585 | 4225 | 4720 | 2354 | 2505 | 3338 | 3089 | 3073 |
| FA 18:1 | 34777 | 13307 | 73321 | 64552 | 31829 | 35751 | 57879 | 54923 | 45792 |
| FA 18:1;2O | 652 | 403 | 482 | 799 | 993 | 354 | 448 | 391 | 565 |
| FA 18:1;O | 1476 | 1028 | 2059 | 2386 | 2126 | 1329 | 1687 | 1826 | 1740 |
| FA 18:2 | 2093 | 715 | 3537 | 4540 | 1250 | 1853 | 4439 | 2791 | 2652 |
| FA 18:2;O | 2561 | 1670 | 2356 | 6611 | 1729 | 2969 | 3223 | 2721 | 2980 |
| FA 18:3 | 702 | 99 | 2361 | 6156 | 791 | 3714 | 3967 | 3727 | 2690 |
| FA 18:3;4O | 1101 | 691 | 530 | 925 | 896 | 525 | 662 | 590 | 740 |
| FA 18:3;O | 486 | 286 | 435 | 1080 | 380 | 479 | 448 | 449 | 505 |
| FA 19:0 | 479 | 318 | 552 | 1484 | 193 | 440 | 546 | 471 | 560 |
| FA 19:0;(2OH) | 816 | 945 | 812 | 1275 | 1373 | 1070 | 1354 | 1150 | 1100 |
| FA 19:1 | 41 | 47 | 339 | 246 | 90 | 123 | 316 | 160 | 170 |
| FA 19:1;2O | 734 | 462 | 395 | 678 | 540 | 326 | 632 | 498 | 533 |
| FA 19:1;O | 151 | 42 | 594 | 369 | 226 | 214 | 166 | 407 | 271 |
| FA 20:0 | 8594 | 4846 | 9582 | 19374 | 3267 | 7351 | 7570 | 7756 | 8542 |
| FA 20:1 | 463 | 225 | 827 | 1401 | 260 | 400 | 769 | 732 | 635 |
| FA 20:3 | 119 | 25 | 460 | 374 | 158 | 216 | 380 | 396 | 266 |
| FA 20:4 | 999 | 188 | 3283 | 5916 | 1093 | 3504 | 3932 | 4217 | 2892 |
| FA 20:5 | 1924 | 272 | 487 | 1643 | 462 | 794 | 790 | 691 | 883 |
| FA 21:0 | 916 | 603 | 950 | 2634 | 280 | 744 | 830 | 796 | 969 |
| FA 21:1;2O | 373 | 212 | 180 | 486 | 267 | 343 | 453 | 330 | 330 |
| FA 22:0 | 2946 | 1440 | 2974 | 5862 | 1455 | 1991 | 2122 | 2674 | 2683 |
| FA 22:1 | 141 | 105 | 150 | 246 | 219 | 88 | 213 | 125 | 161 |
| FA 22:5 | 399 | 182 | 2335 | 1357 | 724 | 735 | 1572 | 3227 | 1316 |
| FA 22:6 | 1145 | 720 | 1606 | 2888 | 542 | 2194 | 2289 | 2507 | 1736 |
| FA 23:0 | 1838 | 1145 | 1341 | 2613 | 997 | 941 | 1173 | 1695 | 1468 |

| FA 23:1 | 110 | 21 | 312 | 594 | 96 | 194 | 364 | 382 | 259 |
| --- | --- | --- | --- | --- | --- | --- | --- | --- | --- |
| FA 24:0 | 11956 | 6180 | 9525 | 18532 | 6449 | 6274 | 7629 | 8852 | 9425 |
| FA 25:0 | 4950 | 2815 | 3352 | 8231 | 3010 | 2242 | 3056 | 3477 | 3892 |
| FA 26:0 | 20690 | 12018 | 18547 | 44059 | 15314 | 13018 | 15013 | 18245 | 19613 |
| FA 26:1;O | 1081 | 860 | 1273 | 4035 | 1265 | 1242 | 2020 | 1522 | 1662 |
| FA 27:0 | 6401 | 3570 | 8924 | 15337 | 4353 | 4492 | 5645 | 7921 | 7080 |
| FA 27:1;O | 327 | 246 | 399 | 1345 | 280 | 321 | 570 | 484 | 496 |
| FA 28:0 | 28592 | 19970 | 29685 | 56393 | 20464 | 15654 | 23815 | 28457 | 27879 |
| FA 29:0 | 8416 | 4984 | 9327 | 16712 | 5605 | 4226 | 6092 | 6913 | 7784 |
| FA 30:0 | 42100 | 20089 | 61224 | 91248 | 26873 | 24235 | 42224 | 52777 | 45096 |
| FA 31:0 | 6273 | 5054 | 7133 | 12244 | 4067 | 3368 | 5545 | 6156 | 6230 |
| FA 32:0 | 18981 | 10904 | 24167 | 38971 | 11782 | 9779 | 14869 | 18429 | 18485 |
| FA 33:0 | 2276 | 1456 | 3040 | 5329 | 1461 | 1383 | 2185 | 2288 | 2427 |
| FA 34:0 | 6629 | 3913 | 7614 | 12353 | 3623 | 3148 | 4349 | 5102 | 5841 |
| FA 34:8 | 711 | 247 | 3551 | 4048 | 558 | 889 | 3215 | 3405 | 2078 |
| FA 42:5 | 2079434 | 1212712 | 1527578 | 4760903 | 2193168 | 741529 | 2225118 | 1635826 | 2047033 |
| FA 42:6 | 2475 | 921 | 1452 | 3993 | 1588 | 954 | 1903 | 1669 | 1869 |
| FA 44:5 | 621633 | 366126 | 528852 | 1996044 | 594242 | 263318 | 808965 | 593115 | 721537 |
| FA 44:6 | 1290 | 615 | 895 | 3419 | 832 | 700 | 1491 | 1447 | 1336 |
| **Dihexosylceramides** |  |  |  |  |  |  |  |  |  |
| Hex2Cer 32:1;2O|Hex2Cer 16:1;2O/16:0 | 166 | 150 | 415 | 125 | 47 | 134 | 202 | 124 | 170 |
| Hex2Cer 33:1;2O|Hex2Cer 17:1;2O/16:0 | 155 | 112 | 286 | 106 | 52 | 114 | 138 | 153 | 140 |
| Hex2Cer 34:0;2O | 604 | 403 | 1089 | 527 | 183 | 608 | 569 | 901 | 610 |
| Hex2Cer 34:1;2O|Hex2Cer 18:1;2O/16:0 | 3775 | 2519 | 9480 | 3901 | 1552 | 4375 | 4792 | 5636 | 4504 |
| Hex2Cer 36:1;2O|Hex2Cer 18:1;2O/18:0 | 126 | 96 | 372 | 101 | 36 | 88 | 128 | 128 | 134 |
| Hex2Cer 38:1;2O|Hex2Cer 16:1;2O/22:0 | 782 | 576 | 2159 | 508 | 230 | 460 | 929 | 475 | 765 |
| Hex2Cer 39:0;2O|Hex2Cer 17:0;2O/22:0 | 928 | 759 | 1653 | 394 | 167 | 380 | 636 | 384 | 663 |
| Hex2Cer 39:1;2O|Hex2Cer 16:1;2O/23:0 | 1101 | 1068 | 2327 | 610 | 244 | 735 | 1178 | 516 | 972 |
| Hex2Cer 40:1;2O|Hex2Cer 18:1;2O/22:0 | 1923 | 1352 | 3898 | 1553 | 682 | 1166 | 2440 | 1527 | 1818 |
| Hex2Cer 40:2;2O|Hex2Cer 16:1;2O/24:1 | 104 | 69 | 264 | 54 | 24 | 69 | 107 | 61 | 94 |
| Hex2Cer 41:1;2O|Hex2Cer 18:1;2O/23:0 | 1713 | 1407 | 1838 | 708 | 292 | 517 | 954 | 567 | 1000 |
| Hex2Cer 42:1;2O|Hex2Cer 18:1;2O/24:0 | 1109 | 1179 | 1600 | 822 | 463 | 796 | 952 | 736 | 957 |
| Hex2Cer 42:2;2O|Hex2Cer 18:1;2O/24:1 | 229 | 157 | 245 | 142 | 64 | 181 | 132 | 141 | 161 |
|  |  |  |  |  |  |  |  |  |  |
| **Hexosylceramidea** |  |  |  |  |  |  |  |  |  |
|  |  |  |  |  |  |  |  |  |  |
| HexCer 33:1;2O|HexCer 17:1;2O/16:0 | 429 | 238 | 777 | 452 | 219 | 171 | 499 | 290 | 384 |
| HexCer 34:0;2O|HexCer 18:0;2O/16:0 | 253 | 137 | 462 | 252 | 94 | 104 | 185 | 235 | 215 |
| HexCer 34:1;2O | 708 | 648 | 840 | 876 | 490 | 542 | 861 | 754 | 715 |
| HexCer 34:1;2O|HexCer 18:1;2O/16:0 | 2170 | 1544 | 3993 | 2743 | 1121 | 1516 | 2398 | 2320 | 2226 |
| HexCer 34:1;3O|HexCer 18:1;2O/16:0;O | 418 | 239 | 568 | 396 | 324 | 247 | 331 | 573 | 387 |
| HexCer 38:1;2O | 179 | 101 | 888 | 306 | 90 | 152 | 552 | 129 | 299 |
| HexCer 39:1;3O | 540 | 400 | 1851 | 1347 | 614 | 689 | 1105 | 936 | 935 |
| HexCer 40:0;3O | 149 | 99 | 699 | 330 | 25 | 237 | 434 | 409 | 298 |
| HexCer 40:1;2O | 1149 | 1160 | 6325 | 2956 | 975 | 1488 | 4461 | 3827 | 2793 |
| HexCer 40:1;2O|HexCer 18:1;2O/22:0 | 345 | 679 | 1086 | 601 | 451 | 256 | 638 | 681 | 592 |
| HexCer 40:1;3O | 1048 | 693 | 2084 | 2223 | 1172 | 1318 | 1561 | 1825 | 1491 |
| HexCer 41:1;2O | 1120 | 1305 | 3423 | 1945 | 792 | 768 | 2371 | 1777 | 1688 |
| HexCer 41:1;2O|HexCer 18:1;2O/23:0 | 3175 | 2074 | 4788 | 2485 | 1441 | 1306 | 2676 | 2911 | 2607 |

| HexCer 41:1;3O | 947 | 608 | 4958 | 4807 | 1326 | 2222 | 3614 | 5000 | 2935 |
| --- | --- | --- | --- | --- | --- | --- | --- | --- | --- |
| HexCer 42:1;2O | 1604 | 699 | 7153 | 4002 | 827 | 1640 | 5174 | 4123 | 3153 |
| HexCer 42:1;3O | 1133 | 673 | 2479 | 3348 | 1311 | 1775 | 2192 | 4246 | 2144 |
| HexCer 43:1;3O | 124 | 59 | 1335 | 741 | 162 | 455 | 758 | 1066 | 587 |
| HexCer 49:5;4O | 2773 | 2526 | 4210 | 1006 | 691 | 908 | 1853 | 950 | 1865 |
| HexCer 51:9;3O | 631 | 859 | 1323 | 267 | 34 | 256 | 698 | 210 | 535 |
| HexCer 53:9;3O | 818 | 628 | 1109 | 439 | 152 | 298 | 364 | 395 | 525 |
| **Lysophophatidylcholines** |  |  |  |  |  |  |  |  |  |
| LPC 14:0 | 1073 | 1053 | 2725 | 693 | 627 | 1627 | 1195 | 5993 | 1873 |
| LPC 15:0 | 1256 | 1042 | 2315 | 1065 | 881 | 1334 | 1170 | 2254 | 1415 |
| LPC 16:0 | 6366 | 4293 | 20903 | 6478 | 3562 | 19928 | 9875 | 52793 | 15524 |
| LPC 17:0 | 280 | 292 | 702 | 181 | 130 | 318 | 278 | 706 | 361 |
| LPC 18:0 | 1456 | 653 | 3999 | 579 | 680 | 1729 | 1731 | 5831 | 2082 |
| LPC 18:1 | 2778 | 1923 | 11916 | 2720 | 1426 | 4741 | 3711 | 9091 | 4788 |
| LPC 18:2 | 1373 | 1229 | 3065 | 824 | 505 | 1356 | 1375 | 2632 | 1545 |
| LPC 26:0 | 371 | 363 | 737 | 437 | 339 | 379 | 524 | 430 | 447 |
| LPC 28:7 | 362 | 489 | 457 | 94 | 170 | 159 | 959 | 478 | 396 |
| **Lysophosphatidylethanolamines** |  |  |  |  |  |  |  |  |  |
| LPE 16:0 | 934 | 893 | 2062 | 1457 | 1157 | 1899 | 1582 | 4398 | 1798 |
| LPE 16:1 | 120 | 214 | 237 | 421 | 230 | 349 | 166 | 538 | 284 |
| LPE 18:0 | 1127 | 792 | 3643 | 1823 | 1039 | 1530 | 1778 | 4656 | 2048 |
| LPE 18:1 | 5497 | 4709 | 14700 | 8038 | 4066 | 8766 | 6923 | 12271 | 8121 |
| LPE 18:2 | 1182 | 1759 | 3102 | 1757 | 974 | 1851 | 1893 | 3357 | 1985 |
| LPE 18:3 | 1448 | 1541 | 1225 | 345 | 540 | 474 | 568 | 1031 | 896 |
| LPE 20:4 | 142 | 191 | 358 | 235 | 143 | 223 | 220 | 366 | 235 |
| LPI 18:0 | 1376 | 789 | 6809 | 612 | 2171 | 1416 | 1275 | 1436 | 1986 |
| LPI 18:1 | 779 | 451 | 4032 | 991 | 2128 | 1778 | 947 | 2147 | 1657 |
| LPI 18:2 | 259 | 304 | 492 | 157 | 383 | 178 | 133 | 331 | 280 |
| **Monoacylglycerols** |  |  |  |  |  |  |  |  |  |
| MG 15:0 | 689 | 1060 | 394 | 364 | 541 | 184 | 360 | 887 | 560 |
| MG 17:3 | 1822 | 2576 | 2288 | 572 | 816 | 807 | 1479 | 1553 | 1489 |
| MG 17:4 | 4941 | 2773 | 1981 | 250 | 1319 | 649 | 649 | 799 | 1670 |
| MG 18:0 | 3511 | 3806 | 3386 | 1069 | 1472 | 1090 | 1551 | 2062 | 2243 |
| MG 19:3 | 694 | 749 | 813 | 301 | 281 | 346 | 447 | 555 | 523 |
| MG 21:4 | 1369 | 866 | 2988 | 1204 | 673 | 772 | 1251 | 1853 | 1372 |
| MG 22:1 | 938 | 794 | 926 | 339 | 342 | 224 | 310 | 528 | 550 |
| MG 24:2 | 459 | 535 | 439 | 252 | 149 | 142 | 143 | 360 | 310 |
| MG 30:7 | 7647 | 3208 | 17791 | 7552 | 3518 | 2778 | 5556 | 7522 | 6946 |
| MG 32:8 | 939 | 643 | 2287 | 834 | 341 | 525 | 950 | 1185 | 963 |
| MG 34:8 | 2634 | 1872 | 2840 | 557 | 1026 | 647 | 959 | 1898 | 1554 |
| MG 36:6 | 508 | 294 | 1584 | 738 | 220 | 493 | 937 | 1080 | 732 |
| **N-acyl ethanolamines** |  |  |  |  |  |  |  |  |  |
| NAE 12:0 | 6165 | 6098 | 5948 | 3183 | 1353 | 3904 | 3831 | 5371 | 4482 |
| NAE 13:1 | 14934 | 9573 | 7387 | 1457 | 2295 | 2152 | 7088 | 5461 | 6293 |

| NAE 14:1 | 20399 | 13663 | 12454 | 3904 | 4625 | 5431 | 8580 | 8104 | 9645 |
| --- | --- | --- | --- | --- | --- | --- | --- | --- | --- |
| NAE 15:1 | 2737 | 2162 | 1885 | 847 | 692 | 1272 | 1205 | 1174 | 1497 |
| NAE 16:1 | 760386 | 571659 | 378867 | 205945 | 171399 | 365392 | 280221 | 242017 | 371986 |
| NAE 16:2 | 1660924 | 1417672 | 820581 | 409584 | 429703 | 766405 | 510814 | 478567 | 811781 |
| NAE 16:3 | 973747 | 753122 | 744734 | 310665 | 210690 | 516905 | 474904 | 627792 | 576570 |
| NAE 16:4 | 76588 | 57270 | 32758 | 10704 | 15212 | 18745 | 19932 | 21895 | 31638 |
| NAE 17:4 | 9895 | 8177 | 6758 | 2759 | 1694 | 3489 | 6007 | 4953 | 5466 |
| NAE 18:1 | 30531 | 21697 | 13140 | 8993 | 6419 | 18730 | 9971 | 7810 | 14661 |
| NAE 18:2 | 44507 | 35405 | 18702 | 11733 | 10393 | 27612 | 14147 | 11186 | 21711 |
| NAE 18:3 | 5441 | 3995 | 4950 | 2504 | 1350 | 3520 | 3244 | 3444 | 3556 |
| NAE 18:4 | 157391 | 141789 | 102428 | 44503 | 40081 | 67344 | 65542 | 63096 | 85272 |
| NAE 18:5 | 228318 | 235631 | 192160 | 72451 | 66885 | 92919 | 99546 | 134895 | 140351 |
| NAE 20:1 | 3488 | 1895 | 1861 | 1163 | 1011 | 1351 | 1316 | 1228 | 1664 |
| NAE 20:2 | 9596 | 7084 | 4342 | 3432 | 2277 | 5172 | 3315 | 3004 | 4778 |
| NAE 20:4 | 7889 | 5968 | 4370 | 2521 | 1797 | 5036 | 3047 | 2475 | 4138 |
| NAE 20:5 | 6208 | 5429 | 3027 | 1855 | 1700 | 3505 | 1696 | 1944 | 3170 |
| NAE 22:3 | 3058 | 2362 | 5234 | 3621 | 540 | 4463 | 3979 | 3597 | 3357 |
| NAE 22:4 | 2309 | 1389 | 911 | 695 | 451 | 914 | 955 | 757 | 1048 |
| NAE 22:5 | 1743 | 1708 | 962 | 935 | 565 | 1246 | 749 | 781 | 1086 |
| NAE 24:4 | 2172 | 1902 | 1357 | 533 | 415 | 706 | 1224 | 1181 | 1186 |
| NAE 5:0 | 2860 | 4258 | 7033 | 1170 | 2301 | 2178 | 2410 | 5005 | 3402 |
| NAE 6:0 | 16233 | 33167 | 43415 | 9136 | 17182 | 27239 | 19241 | 32272 | 24736 |
| NAE 7:0 | 1477 | 1874 | 2265 | 1341 | 223 | 899 | 565 | 881 | 1191 |
| **Phosphatidylcholines** |  |  |  |  |  |  |  |  |  |
| PC 24:0 | 229 | 173 | 937 | 575 | 143 | 439 | 420 | 988 | 488 |
| PC 26:0|PC 12:0_14:0 | 838 | 624 | 4115 | 2005 | 833 | 1453 | 1152 | 4726 | 1968 |
| PC 27:0|PC 12:0_15:0 | 188 | 177 | 777 | 305 | 186 | 232 | 238 | 834 | 367 |
| PC 28:0 | 419 | 393 | 932 | 605 | 275 | 633 | 523 | 1018 | 600 |
| PC 28:0|PC 14:0_14:0 | 14674 | 11941 | 67310 | 40385 | 16795 | 37444 | 29508 | 72125 | 36273 |
| PC 28:1 | 304 | 250 | 815 | 454 | 208 | 327 | 297 | 868 | 440 |
| PC 29:0|PC 14:0_15:0 | 3425 | 2515 | 14706 | 7141 | 2644 | 7577 | 6160 | 12252 | 7052 |
| PC 30:0|PC 14:0_16:0 | 308133 | 196432 | 802818 | 560159 | 230290 | 514428 | 464861 | 704008 | 472641 |
| PC 30:1|PC 14:0_16:1 | 3187 | 2320 | 9337 | 9914 | 3011 | 6372 | 4106 | 12149 | 6299 |
| PC 30:3 | 341 | 278 | 899 | 370 | 229 | 480 | 301 | 555 | 432 |
| PC 31:0|PC 15:0_16:0 | 18146 | 12898 | 43293 | 20658 | 17601 | 24258 | 20015 | 44092 | 25120 |
| PC 31:1 | 1276 | 827 | 3802 | 1323 | 589 | 1396 | 1215 | 1938 | 1546 |
| PC 31:1|PC 15:0_16:1 | 1161 | 719 | 2904 | 2128 | 934 | 1715 | 1523 | 2516 | 1700 |
| PC 32:0 | 5286 | 2869 | 14147 | 8790 | 5646 | 6950 | 6707 | 20960 | 8919 |
| PC 32:0|PC 16:0_16:0 | 272249 | 168902 | 731248 | 507954 | 249350 | 355299 | 430478 | 876724 | 449026 |
| PC 32:1 | 1630 | 1005 | 1989 | 1180 | 1455 | 1258 | 1117 | 2334 | 1496 |
| PC 32:1|PC 14:0_18:1 | 119502 | 65259 | 185410 | 145378 | 96908 | 125086 | 110177 | 229294 | 134627 |
| PC 32:2|PC 16:1_16:1 | 4188 | 3296 | 13299 | 8534 | 2647 | 6685 | 7878 | 8964 | 6936 |
| PC 32:3|PC 14:0_18:3 | 470 | 301 | 1129 | 716 | 318 | 567 | 653 | 1125 | 660 |
| PC 32:3|PC 14:1_18:2 | 4710 | 2837 | 17536 | 14174 | 4377 | 13015 | 10898 | 17371 | 10615 |
| PC 33:0|PC 16:0_17:0 | 9448 | 6780 | 19456 | 6577 | 6155 | 7975 | 7460 | 10683 | 9317 |
| PC 33:1|PC 15:0_18:1 | 18548 | 11954 | 47067 | 16537 | 11117 | 19456 | 26464 | 29983 | 22641 |

| PC 33:2 | 1637 | 823 | 5561 | 1518 | 530 | 1539 | 2528 | 2301 | 2055 |
| --- | --- | --- | --- | --- | --- | --- | --- | --- | --- |
| PC 34:0|PC 16:0_18:0 | 41637 | 27741 | 166389 | 59917 | 29697 | 50356 | 78184 | 153080 | 75875 |
| PC 34:1 | 16636 | 10829 | 26444 | 10404 | 11108 | 13752 | 15077 | 26469 | 16340 |
| PC 34:1|PC 16:0_18:1 | 736123 | 510576 | 1504973 | 648639 | 407106 | 643115 | 743352 | 1119147 | 789129 |
| PC 34:2 | 4336 | 3552 | 6706 | 2973 | 2508 | 3314 | 3712 | 4511 | 3951 |
| PC 34:2|PC 15:1_19:1 | 1146 | 978 | 2064 | 889 | 395 | 798 | 947 | 1148 | 1046 |
| PC 34:2|PC 16:0_18:2 | 116787 | 82791 | 215181 | 98179 | 70939 | 119087 | 124428 | 140972 | 121045 |
| PC 34:3|PC 16:0_18:3 | 11206 | 7120 | 43967 | 32106 | 11280 | 26089 | 27100 | 50771 | 26205 |
| PC 34:4|PC 17:2_17:2 | 244 | 157 | 490 | 482 | 196 | 309 | 308 | 744 | 366 |
| PC 35:0|PC 17:0_18:0 | 1524 | 1192 | 4434 | 858 | 724 | 1108 | 1364 | 2128 | 1666 |
| PC 35:1|PC 17:0_18:1 | 7310 | 6300 | 20662 | 5449 | 3787 | 6623 | 7467 | 10219 | 8477 |
| PC 35:2|PC 17:1_18:1 | 4653 | 3222 | 17565 | 5374 | 2192 | 4767 | 7822 | 7535 | 6641 |
| PC 35:3 | 985 | 581 | 2161 | 839 | 542 | 735 | 1299 | 1473 | 1077 |
| PC 36:0|PC 18:0_18:0 | 1429 | 1292 | 5563 | 1118 | 844 | 1280 | 2124 | 2573 | 2028 |
| PC 36:1 | 718 | 549 | 2106 | 829 | 595 | 796 | 1305 | 2026 | 1115 |
| PC 36:1|PC 18:0_18:1 | 118251 | 79618 | 483928 | 135690 | 66694 | 102697 | 181816 | 343248 | 188993 |
| PC 36:2 | 2200 | 1251 | 2739 | 869 | 871 | 1104 | 1283 | 1824 | 1518 |
| PC 36:2|PC 18:1_18:1 | 145982 | 120126 | 229834 | 63284 | 58305 | 91430 | 78035 | 115851 | 112856 |
| PC 36:3 | 1342 | 991 | 2472 | 929 | 817 | 1134 | 1651 | 1605 | 1367 |
| PC 36:3|PC 18:1_18:2 | 64022 | 42289 | 151394 | 44420 | 27744 | 56024 | 100845 | 86816 | 71694 |
| PC 36:4 | 507 | 378 | 1331 | 589 | 284 | 562 | 736 | 1073 | 683 |
| PC 36:4|PC 18:2_18:2 | 13905 | 8756 | 51633 | 20933 | 8597 | 17243 | 31533 | 46134 | 24842 |
| PC 36:5|PC 16:0_20:5 | 2350 | 1757 | 8041 | 5684 | 2066 | 5763 | 5376 | 7018 | 4757 |
| PC 36:5|PC 18:2_18:3 | 934 | 840 | 2403 | 1173 | 462 | 959 | 1776 | 2344 | 1361 |
| PC 36:6 | 92 | 39 | 180 | 69 | 58 | 79 | 71 | 227 | 102 |
| PC 37:1|PC 19:0_18:1 | 365 | 679 | 1398 | 380 | 203 | 315 | 436 | 712 | 561 |
| PC 37:2|PC 18:1_19:1 | 1228 | 866 | 2368 | 756 | 365 | 711 | 934 | 1065 | 1037 |
| PC 38:1|PC 20:0_18:1 | 1147 | 726 | 1284 | 687 | 519 | 1047 | 671 | 1947 | 1003 |
| PC 38:2 | 888 | 662 | 1587 | 460 | 242 | 541 | 648 | 965 | 749 |
| PC 38:2|PC 20:0_18:2 | 2420 | 1778 | 2607 | 1643 | 1247 | 2655 | 1519 | 3584 | 2182 |
| PC 38:3 | 1559 | 1288 | 4054 | 1175 | 757 | 1458 | 1985 | 2860 | 1892 |
| PC 38:3|PC 20:0_18:3 | 769 | 517 | 1705 | 969 | 470 | 1036 | 839 | 2440 | 1093 |
| PC 38:4 | 1613 | 1141 | 3003 | 1038 | 875 | 1142 | 1499 | 2585 | 1612 |
| PC 38:4|PC 18:1_20:3 | 2116 | 1531 | 7196 | 2613 | 1095 | 2904 | 4219 | 5787 | 3433 |
| PC 38:5|PC 16:0_22:5 | 4792 | 2865 | 10108 | 4873 | 3352 | 8269 | 7091 | 12323 | 6709 |
| PC 38:6|PC 19:3_19:3 | 1168 | 762 | 4886 | 2229 | 865 | 2389 | 2905 | 3759 | 2370 |
| PC 38:7 | 325 | 192 | 543 | 342 | 139 | 344 | 459 | 486 | 354 |
| PC 40:3|PC 25:1_15:2 | 357 | 314 | 414 | 310 | 182 | 509 | 304 | 748 | 392 |
| PC 40:5|PC 18:0_22:5 | 940 | 585 | 1734 | 483 | 415 | 714 | 919 | 1943 | 967 |
| PC 40:6 | 1301 | 804 | 2893 | 989 | 635 | 1208 | 1728 | 2895 | 1557 |
| PC O-29:1 | 371 | 268 | 854 | 388 | 151 | 430 | 503 | 627 | 449 |
| PC O-31:0 | 5643 | 3325 | 8769 | 4965 | 2475 | 4962 | 4766 | 7982 | 5361 |
| PC O-31:1 | 1258 | 927 | 2703 | 1400 | 778 | 1530 | 1954 | 2618 | 1646 |
| PC O-31:6 | 56 | 60 | 184 | 122 | 79 | 108 | 93 | 201 | 113 |
| PC O-33:2 | 1545 | 1129 | 3580 | 1307 | 624 | 1502 | 2980 | 2472 | 1892 |

| PC O-33:3 | 968 | 740 | 1603 | 674 | 535 | 1042 | 1167 | 1148 | 985 |
| --- | --- | --- | --- | --- | --- | --- | --- | --- | --- |
| PC O-33:6 | 265 | 250 | 739 | 485 | 245 | 460 | 430 | 739 | 452 |
| PC O-35:6 | 604 | 370 | 1813 | 1651 | 767 | 1271 | 1154 | 2217 | 1231 |
| PC O-35:9 | 989 | 565 | 2747 | 1918 | 761 | 1709 | 1630 | 2653 | 1622 |
| PC O-37:6 | 204 | 166 | 654 | 263 | 166 | 228 | 239 | 440 | 295 |
| PC O-38:10 | 699 | 563 | 2394 | 1156 | 580 | 725 | 827 | 1286 | 1029 |
| PC O-39:0 | 1034 | 898 | 1261 | 836 | 505 | 556 | 594 | 818 | 813 |
| PC O-39:10 | 2929 | 1562 | 3458 | 1446 | 1610 | 1828 | 1773 | 3230 | 2229 |
| PC O-33:4 | 2728 | 1696 | 12682 | 7630 | 2943 | 9415 | 8128 | 14381 | 7450 |
| **Phosphatidylethanolamines** |  |  |  |  |  |  |  |  |  |
| PE 28:0|PE 14:0_14:0 | 1113 | 849 | 1048 | 1292 | 764 | 978 | 1190 | 1265 | 1062 |
| PE 30:0|PE 14:0_16:0 | 4203 | 4631 | 4184 | 2489 | 1776 | 3262 | 4064 | 2722 | 3416 |
| PE 30:1|PE 12:0_18:1 | 1355 | 1443 | 1817 | 2727 | 1393 | 1303 | 1555 | 3086 | 1835 |
| PE 31:0|PE 15:0_16:0 | **632** | 747 | 784 | 530 | 332 | 591 | 770 | 597 | 623 |
| PE 32:0|PE 16:0_16:0 | 6849 | 7123 | 6754 | 6113 | 4168 | 5658 | 7964 | 7181 | 6476 |
| PE 32:1|PE 14:0_18:1 | 22952 | 26170 | 21419 | 31242 | 22399 | 19587 | 23440 | 34060 | 25159 |
| PE 32:1|PE 16:0_16:1 | 5500 | 3750 | 8240 | 10007 | 4390 | 6420 | 4772 | 9363 | 6555 |
| PE 32:2 | 497 | 430 | 923 | 679 | 276 | 332 | 521 | 642 | 538 |
| PE 32:2|PE 14:0_18:2 | 2930 | 2810 | 3427 | 4701 | 2779 | 2468 | 4793 | 5674 | 3698 |
| PE 33:0|PE 16:0_17:0 | 849 | 1383 | 1440 | 771 | 430 | 759 | 1130 | 951 | 964 |
| PE 33:1|PE 15:0_18:1 | 5260 | 6117 | 6436 | 5623 | 3929 | 4079 | 5487 | 7023 | 5494 |
| PE 34:0|PE 16:0_18:0 | 4099 | 4155 | 5822 | 2952 | 1790 | 3043 | 5435 | 4455 | 3969 |
| PE 34:1 | 3923 | 3233 | 5111 | 3089 | 2170 | 2363 | 3423 | 4091 | 3425 |
| PE 34:1|PE 16:0_18:1 | 189821 | 217568 | 229333 | 226489 | 138389 | 156416 | 235654 | 269540 | 207901 |
| PE 34:2 | 899 | 803 | 1496 | 903 | 594 | 732 | 1050 | 1402 | 985 |
| PE 34:2|PE 16:0_18:2 | 73595 | 85518 | 74696 | 93708 | 62793 | 54166 | 98999 | 127584 | 83882 |
| PE 34:3|PE 16:0_18:3 | 6731 | 7814 | 6857 | 11343 | 7432 | 5739 | 9040 | 18188 | 9143 |
| PE 34:3|PE 16:1_18:2 | 1908 | 1929 | 1924 | 4378 | 2552 | 2087 | 3319 | 5004 | 2888 |
| PE 34:4|PE 14:0_20:4 | 404 | 181 | 636 | 834 | 369 | 570 | 1041 | 1069 | 638 |
| PE 34:4|PE 16:1_18:3 | 206 | 82 | 205 | 920 | 443 | 378 | 434 | 1181 | 481 |
| PE 35:0|PE 17:0_18:0 | 329 | 489 | 1100 | 308 | 137 | 346 | 672 | 266 | 456 |
| PE 35:1|PE 17:0_18:1 | 10494 | 10761 | 16280 | 9745 | 6105 | 6952 | 14457 | 12380 | 10897 |
| PE 35:2|PE 17:1_18:1 | 8552 | 9762 | 10606 | 8910 | 5793 | 6271 | 10886 | 12660 | 9180 |
| PE 35:3|PE 17:1_18:2 | 611 | 816 | 1142 | 967 | 630 | 741 | 1440 | 1709 | 1007 |
| PE 36:0|PE 18:0_18:0 | 360 | 418 | 834 | 541 | 253 | 323 | 638 | 1305 | 584 |
| PE 36:1|PE 18:0_18:1 | 169285 | 186554 | 327689 | 189445 | 101659 | 113531 | 279868 | 305084 | 209139 |
| PE 36:2 | 1560 | 1059 | 4447 | 2341 | 1135 | 1739 | 3157 | 5058 | 2562 |
| PE 36:2|PE 18:1_18:1 | 479901 | 535588 | 751992 | 467271 | 286034 | 368019 | 586632 | 667738 | 517897 |
| PE 36:3 | 1620 | 1316 | 3416 | 1202 | 672 | 1069 | 1688 | 2277 | 1657 |
| PE 36:3;2O|PE 14:0_22:3;2O | 661 | 421 | 2161 | 812 | 799 | 627 | 1229 | 933 | 955 |
| PE 36:3|PE 18:1_18:2 | 124455 | 139231 | 161368 | 116131 | 78564 | 86743 | 163018 | 186965 | 132059 |
| PE 36:4|PE 16:0_20:4 | 9012 | 11092 | 11788 | 13814 | 10251 | 9286 | 14850 | 21463 | 12694 |
| PE 36:4|PE 18:1_18:3 | 21158 | 25616 | 29707 | 27688 | 17805 | 19356 | 34689 | 58007 | 29253 |
| PE 36:5|PE 16:0_20:5 | 1757 | 2074 | 2696 | 3781 | 2146 | 2438 | 3024 | 5332 | 2906 |
| PE 36:5|PE 18:2_18:3 | 852 | 850 | 1403 | 2094 | 1208 | 1335 | 2358 | 3799 | 1738 |
| PE 37:1|PE 18:0_19:1 | 1213 | 960 | 2074 | 1045 | 440 | 475 | 1657 | 1194 | 1132 |

| PE 37:2|PE 18:1_19:1 | 3939 | 3639 | 5512 | 3407 | 1465 | 2556 | 4325 | 4181 | 3628 |
| --- | --- | --- | --- | --- | --- | --- | --- | --- | --- |
| PE 37:3|PE 18:1_19:2 | 808 | 672 | 1372 | 750 | 395 | 534 | 986 | 1217 | 842 |
| PE 38:1|PE 20:0_18:1 | 2017 | 2316 | 4068 | 1779 | 654 | 1640 | 3403 | 2728 | 2326 |
| PE 38:2|PE 18:1_20:1 | 4278 | 4287 | 6861 | 4646 | 1991 | 3497 | 5326 | 4538 | 4428 |
| PE 38:3|PE 18:0_20:3 | 7986 | 8390 | 12360 | 7504 | 4555 | 5590 | 13280 | 17520 | 9648 |
| PE 38:3|PE 18:1_20:2 | 1811 | 1921 | 2872 | 1800 | 1095 | 1529 | 2814 | 3045 | 2111 |
| PE 38:4|PE 18:0_20:4 | 8759 | 10427 | 17181 | 11852 | 7755 | 7603 | 15637 | 25074 | 13036 |
| PE 38:4|PE 18:1_20:3 | 7654 | 8147 | 12327 | 8250 | 5608 | 8358 | 13177 | 15613 | 9892 |
| PE 38:5|PE 16:0_22:5 | 13512 | 14700 | 18704 | 18079 | 11677 | 14283 | 23742 | 35498 | 18774 |
| PE 38:5|PE 18:1_20:4 | 2911 | 2017 | 9520 | 6955 | 2042 | 4966 | 8341 | 13085 | 6230 |
| PE 38:6 | 1040 | 632 | 2256 | 1360 | 621 | 1147 | 1345 | 2266 | 1333 |
| PE 39:2|PE 18:1_21:1 | 753 | 974 | 899 | 833 | 427 | 568 | 1000 | 875 | 791 |
| PE 40:1|PE 22:0_18:1 | 1644 | 1347 | 3312 | 764 | 391 | 1246 | 2612 | 1813 | 1641 |
| PE 40:2|PE 18:1_22:1 | 478 | 270 | 869 | 487 | 216 | 544 | 942 | 980 | 598 |
| PE 40:4|PE 18:0_22:4 | 972 | 586 | 2186 | 1205 | 621 | 699 | 2193 | 4335 | 1600 |
| PE 40:5|PE 18:0_22:5 | 8880 | 9230 | 14605 | 8530 | 4916 | 5473 | 16545 | 25804 | 11748 |
| PE 44:10|PE 22:5_22:5 | 2055 | 1398 | 5465 | 2850 | 2577 | 1471 | 2577 | 3633 | 2753 |
| PE O-30:1|PE O-14:1_16:0 | 229 | 82 | 205 | 152 | 57 | 179 | 281 | 162 | 168 |
| PE O-31:1|PE O-15:1_16:0 | 786 | 877 | 901 | 832 | 418 | 987 | 1348 | 681 | 854 |
| PE O-31:2|PE O-15:1_16:1 | 462 | 346 | 472 | 719 | 279 | 508 | 894 | 776 | 557 |
| PE O-32:1|PE O-16:1_16:0 | 385 | 449 | 413 | 430 | 121 | 369 | 534 | 349 | 381 |
| PE O-32:2|PE O-14:1_18:1 | 2323 | 2486 | 2388 | 3026 | 1610 | 1880 | 3301 | 4556 | 2696 |
| PE O-32:3|PE O-14:1_18:2 | 760 | 1054 | 848 | 955 | 388 | 672 | 1667 | 1389 | 967 |
| PE O-33:2|PE O-15:1_18:1 | 4911 | 5279 | 6807 | 5370 | 2795 | 4357 | 9408 | 8176 | 5888 |
| PE O-33:3|PE O-15:1_18:2 | 2206 | 2543 | 2614 | 2123 | 1368 | 1890 | 4680 | 3068 | 2561 |
| PE O-33:4|PE O-15:1_18:3 | 287 | 422 | 546 | 492 | 248 | 390 | 846 | 833 | 508 |
| PE O-34:2|PE O-16:1_18:1 | 16869 | 16517 | 21139 | 16811 | 9288 | 12437 | 22588 | 30053 | 18213 |
| PE O-34:3|PE O-16:1_18:2 | 8565 | 9193 | 11104 | 9015 | 4874 | 6646 | 14020 | 15304 | 9840 |
| PE O-34:4|PE O-16:1_18:3 | 1525 | 1693 | 2256 | 2241 | 1113 | 1488 | 2899 | 4909 | 2266 |
| PE O-35:1 | 4081 | 2791 | 6561 | 2738 | 1548 | 3614 | 5042 | 5302 | 3960 |
| PE O-35:2|PE O-17:1_18:1 | 1799 | 2031 | 3274 | 2618 | 1209 | 1789 | 3116 | 3743 | 2447 |
| PE O-35:3|PE O-17:1_18:2 | 1306 | 1245 | 1463 | 1234 | 572 | 904 | 1856 | 1800 | 1297 |
| PE O-35:3|PE O-17:2_18:1 | 319 | 329 | 519 | 378 | 157 | 323 | 719 | 462 | 401 |
| PE O-35:5|PE O-15:1_20:4 | 535 | 346 | 627 | 652 | 366 | 619 | 1381 | 655 | 648 |
| PE O-35:6|PE O-15:1_20:5 | 84 | 258 | 333 | 316 | 134 | 252 | 620 | 232 | 279 |
| PE O-36:2|PE O-18:0_18:2 | 950 | 1112 | 546 | 808 | 384 | 3548 | 524 | 1144 | 1127 |
| PE O-36:2|PE O-18:1_18:1 | 4907 | 4544 | 5967 | 5745 | 2698 | 3884 | 4994 | 7586 | 5040 |
| PE O-36:3|PE O-18:1_18:2 | 651 | 859 | 996 | 657 | 500 | 744 | 1103 | 1752 | 908 |
| PE O-36:3|PE O-18:2_18:1 | 4896 | 4612 | 5779 | 5722 | 2601 | 3797 | 6843 | 8324 | 5322 |
| PE O-36:4|PE O-18:2_18:2 | 1698 | 2812 | 2560 | 2294 | 1364 | 1919 | 3344 | 4274 | 2533 |
| PE O-36:5|PE O-16:1_20:4 | 1965 | 2053 | 2304 | 2352 | 1262 | 2004 | 3829 | 3455 | 2403 |
| PE O-36:5|PE O-18:2_18:3 | 418 | 316 | 531 | 469 | 192 | 405 | 562 | 1064 | 495 |
| PE O-36:6|PE O-16:1_20:5 | 898 | 677 | 944 | 1015 | 560 | 1004 | 1864 | 1884 | 1106 |
| PE O-38:4|PE O-18:1_20:3 | 356 | 371 | 539 | 736 | 238 | 589 | 817 | 810 | 557 |
| PE O-38:5|PE O-18:1_20:4 | 837 | 786 | 1265 | 1416 | 617 | 1046 | 1870 | 1741 | 1197 |

| PE O-38:6|PE O-16:1_22:5 | 1692 | 1588 | 2638 | 1881 | 1073 | 1707 | 3995 | 3880 | 2307 |
| --- | --- | --- | --- | --- | --- | --- | --- | --- | --- |
| PE O-40:6|PE O-18:1_22:5 | 793 | 524 | 771 | 699 | 488 | 670 | 1585 | 1339 | 859 |
| PE O-19:0_18:1 | 454 | 211 | 1504 | 1687 | 176 | 596 | 1399 | 1570 | 950 |
| PE P-30:1|PE P-14:0_16:1 | 241 | 184 | 571 | 274 | 118 | 127 | 321 | 558 | 299 |
| PE P-31:0|PE P-15:0_16:0 | 482 | 427 | 643 | 293 | 153 | 304 | 513 | 453 | 408 |
| PE P-32:1|PE P-14:0_18:1 | 1150 | 779 | 1607 | 992 | 525 | 670 | 980 | 1452 | 1019 |
| PE P-32:2|PE P-14:0_18:2 | 476 | 362 | 681 | 254 | 226 | 332 | 639 | 652 | 453 |
| PE P-33:1|PE P-15:0_18:1 | 1215 | 988 | 2062 | 909 | 479 | 660 | 1445 | 1364 | 1140 |
| PE P-33:2|PE P-15:0_18:2 | 588 | 452 | 1041 | 664 | 304 | 588 | 1181 | 816 | 704 |
| PE P-33:3|PE P-15:0_18:3 | 379 | 238 | 723 | 430 | 180 | 327 | 454 | 880 | 451 |
| PE P-34:1|PE P-16:0_18:1 | 4982 | 3224 | 10660 | 5959 | 2107 | 3806 | 5501 | 9730 | 5746 |
| PE P-34:2|PE P-16:0_18:2 | 2387 | 1828 | 3201 | 1663 | 940 | 1473 | 2384 | 2955 | 2104 |
| PE P-34:3|PE P-16:0_18:3 | 501 | 359 | 936 | 598 | 264 | 426 | 781 | 1134 | 625 |
| PE P-36:1|PE P-18:0_18:1 | 3068 | 1943 | 4106 | 1791 | 1119 | 1608 | 2422 | 2862 | 2365 |
| PE P-36:2|PE P-18:0_18:2 | 1521 | 975 | 2054 | 1182 | 549 | 1118 | 1322 | 1966 | 1336 |
| PE P-36:2|PE P-18:1_18:1 | 1191 | 876 | 2272 | 1283 | 482 | 800 | 988 | 1798 | 1211 |
| PE P-36:3|PE P-16:0_20:3 | 541 | 534 | 991 | 513 | 291 | 464 | 723 | 887 | 618 |
| PE P-36:3|PE P-18:1_18:2 | 620 | 661 | 1052 | 524 | 330 | 512 | 672 | 1073 | 680 |
| PE P-36:4|PE P-16:0_20:4 | 687 | 487 | 828 | 513 | 279 | 526 | 779 | 688 | 598 |
| **Phosphatidylinositolss** |  |  |  |  |  |  |  |  |  |
| PI 30:0 | 690 | 210 | 2120 | 758 | 1095 | 683 | 932 | 1036 | 941 |
| PI 30:0|PI 14:0_16:0 | 741 | 299 | 2109 | 777 | 1118 | 764 | 939 | 1040 | 973 |
| PI 32:0|PI 16:0_16:0 | 4058 | 1997 | 7423 | 5524 | 5820 | 4760 | 4916 | 7127 | 5203 |
| PI 32:1|PI 14:0_18:1 | 2376 | 693 | 5252 | 3260 | 4466 | 2933 | 2719 | 3913 | 3201 |
| PI 33:1 | 709 | 223 | 2117 | 815 | 1001 | 677 | 889 | 1197 | 953 |
| PI 34:0|PI 16:0_18:0 | 5103 | 2866 | 13576 | 5005 | 5599 | 4044 | 5760 | 8697 | 6331 |
| PI 34:1|PI 16:0_18:1 | 25481 | 12616 | 51694 | 26436 | 30961 | 22403 | 26546 | 38759 | 29362 |
| PI 34:2|PI 16:1_18:1 | 4841 | 2356 | 9382 | 5655 | 7303 | 4529 | 6630 | 8143 | 6105 |
| PI 34:3 | 242 | 25 | 480 | 453 | 458 | 348 | 392 | 714 | 389 |
| PI 35:2 | 423 | 256 | 1911 | 592 | 529 | 429 | 823 | 889 | 731 |
| PI 36:0 | 1375 | 588 | 6800 | 1196 | 1122 | 1129 | 1439 | 1413 | 1883 |
| PI 36:1|PI 18:0_18:1 | 108367 | 55727 | 357422 | 73847 | 84397 | 71402 | 109270 | 108880 | 121164 |
| PI 36:2 | 552 | 383 | 1583 | 399 | 441 | 412 | 497 | 1068 | 667 |
| PI 36:2|PI 18:1_18:1 | 56189 | 24990 | 136177 | 37156 | 43220 | 33596 | 63046 | 55735 | 56264 |
| PI 36:3|PI 18:1_18:2 | 8788 | 3500 | 21276 | 8360 | 9405 | 6643 | 12840 | 13133 | 10493 |
| PI 36:4|PI 16:0_20:4 | 2310 | 1038 | 3691 | 2021 | 2245 | 2525 | 3484 | 2317 | 2454 |
| PI 36:4|PI 18:2_18:2 | 641 | 101 | 1532 | 902 | 905 | 621 | 1092 | 1682 | 935 |
| PI 36:5 | 781 | 599 | 1369 | 889 | 790 | 1001 | 1427 | 1202 | 1007 |
| PI 37:1 | 1554 | 685 | 5885 | 1112 | 1121 | 915 | 1588 | 1582 | 1805 |
| PI 37:2 | 1161 | 509 | 3565 | 905 | 878 | 955 | 1294 | 1232 | 1312 |
| PI 38:1 | 2113 | 900 | 7252 | 1672 | 1554 | 1278 | 2011 | 1899 | 2335 |
| PI 38:2 | 3521 | 1724 | 11978 | 2518 | 2881 | 2584 | 3904 | 3465 | 4072 |
| PI 38:3|PI 18:0_20:3 | 12325 | 5340 | 34786 | 6119 | 7461 | 10247 | 20118 | 8771 | 13146 |
| PI 38:4|PI 18:0_20:4 | 14903 | 6629 | 42910 | 7968 | 9191 | 11776 | 22224 | 10086 | 15711 |
| PI 38:4|PI 18:1_20:3 | 3968 | 1824 | 7932 | 3074 | 3281 | 4231 | 6157 | 4886 | 4419 |

| PI 38:5|PI 18:0_20:5 | 7383 | 3346 | 17914 | 4797 | 5454 | 6659 | 10028 | 7160 | 7843 |
| --- | --- | --- | --- | --- | --- | --- | --- | --- | --- |
| PI 38:5|PI 18:1_20:4 | 4977 | 1927 | 10868 | 4420 | 5242 | 6296 | 8629 | 6218 | 6072 |
| PI 38:6 | 910 | 344 | 2568 | 1404 | 1692 | 1442 | 1818 | 2040 | 1527 |
| PI 40:3 | 13496 | 14423 | 12406 | 16791 | 2654 | 13908 | 10512 | 12648 | 12105 |
| PI 40:6 | 119 | 85 | 715 | 265 | 267 | 297 | 389 | 631 | 346 |
| PI 42:10 | 2980 | 932 | 5224 | 1535 | 1813 | 1264 | 2605 | 2600 | 2369 |
| **Phosphatidylserines** |  |  |  |  |  |  |  |  |  |
| PS 34:1|PS 16:0_18:1 | 15529 | 9719 | 16897 | 9282 | 13928 | 10800 | 10079 | 11502 | 12217 |
| PS 34:2|PS 16:0_18:2 | 4841 | 2846 | 6854 | 3928 | 4578 | 4368 | 4694 | 4798 | 4613 |
| PS 34:3|PS 16:0_18:3 | 211 | 177 | 480 | 408 | 457 | 416 | 329 | 385 | 358 |
| PS 35:1|PS 18:0_17:1 | 3922 | 2337 | 8494 | 2264 | 3224 | 3077 | 4462 | 3633 | 3927 |
| PS 36:0|PS 18:0_18:0 | 20212 | 13762 | 47424 | 15332 | 15551 | 15649 | 21857 | 29636 | 22428 |
| PS 36:1|PS 18:0_18:1 | 135791 | 82795 | 363093 | 106709 | 129125 | 107294 | 156939 | 185169 | 158364 |
| PS 36:2|PS 18:1_18:1 | 82823 | 48735 | 195476 | 56485 | 69821 | 58030 | 103145 | 98170 | 89086 |
| PS 36:3|PS 18:0_18:3 | 3023 | 1615 | 8701 | 2302 | 1924 | 2287 | 4292 | 6318 | 3808 |
| PS 36:3|PS 18:1_18:2 | 14553 | 7609 | 33780 | 12075 | 14500 | 10747 | 19153 | 24122 | 17067 |
| PS 36:4|PS 18:1_18:3 | 851 | 405 | 2845 | 1254 | 1512 | 1264 | 1508 | 2203 | 1480 |
| PS 38:1|PS 20:0_18:1 | 4559 | 2688 | 15932 | 3036 | 3316 | 4153 | 4727 | 4758 | 5396 |
| PS 38:2|PS 18:1_20:1 | 2667 | 1696 | 7972 | 2186 | 1973 | 2353 | 3269 | 3174 | 3161 |
| PS 38:3 | 16386 | 10804 | 38384 | 30415 | 14984 | 14763 | 27296 | 21002 | 21754 |
| PS 38:3|PS 18:0_20:3 | 10622 | 6821 | 26205 | 9916 | 11272 | 10429 | 16396 | 17746 | 13676 |
| PS 38:4|PS 18:1_20:3 | 2669 | 1081 | 4938 | 1405 | 2371 | 1893 | 2692 | 3691 | 2593 |
| PS 38:5|PS 16:0_22:5 | 1131 | 564 | 2337 | 1252 | 1071 | 1590 | 1994 | 1838 | 1472 |
| PS 39:1|PS 21:0_18:1 | 3756 | 2041 | 11648 | 2907 | 2582 | 3153 | 4456 | 4257 | 4350 |
| PS 39:2|PS 18:1_21:1 | 2353 | 1865 | 5654 | 2227 | 1704 | 1759 | 2359 | 2627 | 2569 |
| PS 40:1|PS 22:0_18:1 | 3656 | 2643 | 15121 | 3502 | 4237 | 4543 | 4976 | 5419 | 5512 |
| PS 40:2 | 1695 | 554 | 6087 | 1518 | 1595 | 1694 | 2198 | 2465 | 2226 |
| PS 40:2|PS 22:0_18:2 | 1527 | 818 | 5843 | 1385 | 1353 | 1351 | 1847 | 2257 | 2048 |
| PS 40:3 | 1015 | 788 | 5780 | 2916 | 916 | 1361 | 3371 | 2047 | 2274 |
| PS 40:4|PS 18:0_22:4 | 2107 | 1009 | 6913 | 2494 | 2370 | 1672 | 3380 | 5822 | 3221 |
| PS 40:5|PS 18:0_22:5 | 18895 | 11086 | 49217 | 17080 | 20570 | 16809 | 30645 | 42769 | 25884 |
| PS 40:6|PS 18:1_22:5 | 3104 | 1474 | 6960 | 2848 | 3251 | 2350 | 5156 | 6683 | 3979 |
| PS 41:2 | 580 | 170 | 3338 | 901 | 817 | 902 | 1096 | 1512 | 1164 |
| PS 42:1|PS 24:0_18:1 | 660 | 352 | 3355 | 667 | 768 | 991 | 966 | 1451 | 1151 |
| **Sulfatides** |  |  |  |  |  |  |  |  |  |
| SHexCer 35:0;3O | 2332 | 623 | 4679 | 2578 | 5288 | 1229 | 3319 | 3506 | 2944 |
| SHexCer 36:4;2O | 2862 | 1351 | 2449 | 1720 | 5073 | 987 | 2213 | 2641 | 2412 |
| SHexCer 38:2;2O | 421 | 89 | 500 | 291 | 475 | 181 | 453 | 142 | 319 |
| SHexCer 38:7;3O | 287 | 66 | 521 | 628 | 1145 | 278 | 428 | 506 | 483 |
| SHexCer 39:5;3O | 1087 | 350 | 1694 | 1199 | 3381 | 732 | 1161 | 1360 | 1370 |
| SHexCer 39:6;3O | 461 | 65 | 955 | 633 | 1184 | 275 | 730 | 608 | 614 |
| SHexCer 39:7;3O | 6684 | 2645 | 5694 | 6476 | 9551 | 2698 | 7028 | 6907 | 5960 |
| SHexCer 39:8;3O | 2082 | 864 | 1738 | 2448 | 3613 | 925 | 2612 | 2930 | 2151 |
| SHexCer 40:2;2O | 3984 | 1478 | 3929 | 2680 | 5214 | 2140 | 3511 | 3608 | 3318 |
| SHexCer 40:3;3O | 1360 | 487 | 2946 | 1780 | 2739 | 836 | 2054 | 4012 | 2027 |
| SHexCer 40:8;3O | 347 | 138 | 601 | 370 | 1128 | 222 | 335 | 393 | 442 |

| SHexCer 40:9;3O | 919 | 333 | 1121 | 842 | 2943 | 507 | 774 | 849 | 1036 |
| --- | --- | --- | --- | --- | --- | --- | --- | --- | --- |
| SHexCer 41:8;3O | 13889 | 6322 | 12911 | 7651 | 19548 | 4324 | 10125 | 11684 | 10807 |
| SHexCer 42:2;2O | 212 | 64 | 373 | 150 | 239 | 74 | 316 | 145 | 197 |
| SHexCer 42:4;3O | 3968 | 1294 | 7847 | 4825 | 6955 | 1823 | 5730 | 9458 | 5238 |
| SHexCer 43:0;3O | 363 | 48 | 2157 | 559 | 870 | 362 | 1503 | 1121 | 873 |
| SHexCer 44:2;2O | 2817 | 922 | 3886 | 1836 | 3429 | 813 | 3178 | 1068 | 2244 |
| SHexCer 46:2;2O | 7992 | 3815 | 11981 | 7838 | 16372 | 3921 | 8108 | 8314 | 8543 |
| **Sphingomyelins** |  |  |  |  |  |  |  |  |  |
| SM 30:0;2O|SM 22:0;2O/8:0 | 1030 | 956 | 1705 | 715 | 211 | 977 | 1037 | 727 | 920 |
| SM 30:1;2O|SM 16:1;2O/14:0 | 937 | 744 | 2084 | 538 | 339 | 856 | 1086 | 917 | 938 |
| SM 31:0;2O|SM 19:0;2O/12:0 | 313 | 258 | 542 | 195 | 94 | 222 | 283 | 222 | 266 |
| SM 31:1;2O|SM 17:1;2O/14:0 | 741 | 487 | 2082 | 621 | 278 | 694 | 1028 | 760 | 836 |
| SM 32:0;2O|SM 22:0;2O/10:0 | 23390 | 15467 | 53597 | 26407 | 10061 | 36875 | 31930 | 34076 | 28975 |
| SM 32:1;2O|SM 16:1;2O/16:0 | 38652 | 23794 | 131081 | 48252 | 22770 | 67485 | 82394 | 75693 | 61265 |
| SM 33:0;2O | 2885 | 1924 | 8684 | 3629 | 1356 | 3585 | 4041 | 5061 | 3896 |
| SM 33:1;2O|SM 17:1;2O/16:0 | 19384 | 11204 | 82024 | 34146 | 13013 | 38143 | 42999 | 51797 | 36589 |
| SM 34:0;2O | 22078 | 13473 | 35261 | 25156 | 15515 | 23821 | 17142 | 49829 | 25284 |
| SM 34:1;2O | 2030 | 542 | 2665 | 3171 | 4291 | 1741 | 3342 | 4263 | 2755 |
| SM 34:1;2O|SM 18:1;2O/16:0 | 216869 | 141576 | 676044 | 454866 | 189324 | 425978 | 433766 | 708300 | 405840 |
| SM 34:1;3O | 391 | 262 | 997 | 896 | 387 | 710 | 504 | 1473 | 703 |
| SM 34:2;2O|SM 25:2;2O/9:0 | 1849 | 1426 | 6243 | 3928 | 1631 | 3799 | 3524 | 7146 | 3693 |
| SM 35:0;2O | 463 | 349 | 1370 | 462 | 314 | 561 | 697 | 899 | 639 |
| SM 35:1;2O|SM 16:1;2O/19:0 | 4529 | 2700 | 12304 | 4211 | 2616 | 5649 | 5730 | 8209 | 5744 |
| SM 35:1;2O|SM 18:1;2O/17:0 | 2913 | 2179 | 7208 | 1987 | 1224 | 2749 | 3082 | 4339 | 3210 |
| SM 35:2;2O | 370 | 268 | 1030 | 349 | 183 | 447 | 394 | 509 | 444 |
| SM 35:2;2O|SM 21:1;2O/14:1 | 287 | 185 | 738 | 319 | 137 | 279 | 322 | 464 | 341 |
| SM 35:2;3O | 1029 | 648 | 1689 | 863 | 459 | 751 | 1159 | 1423 | 1003 |
| SM 36:0;2O | 1914 | 1401 | 3247 | 1133 | 595 | 1012 | 1296 | 1514 | 1514 |
| SM 36:1;2O|SM 15:0;2O/21:1 | 7534 | 4740 | 32116 | 13523 | 4545 | 10498 | 17782 | 21622 | 14045 |
| SM 36:2;2O|SM 19:1;2O/17:1 | 1871 | 1113 | 4906 | 1710 | 1023 | 1406 | 2235 | 4453 | 2340 |
| SM 37:0;2O | 1594 | 1193 | 2069 | 1249 | 573 | 784 | 1502 | 1123 | 1261 |
| SM 37:1;2O | 729 | 460 | 1657 | 394 | 272 | 478 | 713 | 741 | 680 |
| SM 38:0;2O|SM 16:0;2O/22:0 | 18630 | 15040 | 17667 | 6772 | 3973 | 6138 | 10527 | 9123 | 10984 |
| SM 38:1;2O|SM 16:1;2O/22:0 | 75617 | 53654 | 231202 | 67995 | 33891 | 62672 | 119213 | 68151 | 89049 |
| SM 38:2;2O|SM 19:2;2O/19:0 | 887 | 528 | 1503 | 439 | 405 | 434 | 802 | 785 | 723 |
| SM 39:0;2O | 38647 | 26167 | 50589 | 15636 | 9460 | 18276 | 29782 | 20293 | 26106 |
| SM 39:1;2O|SM 17:1;2O/22:0 | 1833 | 1492 | 3719 | 1546 | 587 | 1793 | 1503 | 2297 | 1846 |
| SM 39:1;3O | 4256 | 3498 | 9744 | 5288 | 3444 | 3241 | 3979 | 4783 | 4779 |
| SM 39:2;2O|SM 19:1;2O/20:1 | 1428 | 1099 | 2190 | 998 | 614 | 852 | 1598 | 1483 | 1283 |
| SM 40:0;2O | 15038 | 13739 | 18316 | 6990 | 5129 | 6877 | 5908 | 8870 | 10108 |
| SM 40:1;2O|SM 16:1;2O/24:0 | 164053 | 114197 | 305687 | 149720 | 85434 | 138854 | 198528 | 176549 | 166628 |
| SM 40:1;2O|SM 18:1;2O/22:0 | 2805 | 2290 | 3025 | 1068 | 923 | 1377 | 1714 | 2192 | 1924 |
| SM 40:1;3O | 720 | 631 | 1262 | 842 | 408 | 808 | 470 | 1033 | 772 |
| SM 40:2;2O|SM 18:1;2O/22:1 | 9526 | 6150 | 22125 | 8701 | 4154 | 10181 | 15679 | 9826 | 10793 |
| SM 40:8;2O | 687 | 539 | 657 | 309 | 231 | 269 | 362 | 405 | 432 |
| SM 41:0;2O | 4271 | 3972 | 3820 | 2826 | 1815 | 2533 | 2445 | 3583 | 3158 |

| SM 41:1;2O|SM 18:1;2O/23:0 | 33637 | 34559 | 49339 | 27931 | 21582 | 21075 | 20406 | 31735 | 30033 |
| --- | --- | --- | --- | --- | --- | --- | --- | --- | --- |
| SM 41:2;2O|SM 18:1;2O/23:1 | 4237 | 4225 | 7564 | 6028 | 3303 | 6184 | 4067 | 9496 | 5638 |
| SM 42:1;2O|SM 18:1;2O/24:0 | 40850 | 37839 | 44176 | 44661 | 27758 | 45832 | 32710 | 54355 | 41023 |
| SM 42:2;2O|SM 18:1;2O/24:1 | 8246 | 5982 | 12017 | 8728 | 6119 | 11060 | 11009 | 14782 | 9743 |
| SM 32:0;2O | 664 | 605 | 1169 | 546 | 279 | 876 | 693 | 831 | 708 |
| SM 33:1;2O | 462 | 436 | 1687 | 687 | 350 | 851 | 817 | 1009 | 787 |
| SM 34:1;2O | 6497 | 3980 | 34823 | 24259 | 8767 | 26576 | 25551 | 42489 | 21618 |
| SM 40:1;2O | 2515 | 2172 | 5992 | 2887 | 1317 | 3217 | 4319 | 3184 | 3201 |
| SM 42:2;2O | 323 | 180 | 387 | 313 | 182 | 362 | 331 | 447 | 316 |
| SM 42:2;3O | 112 | 102 | 334 | 149 | 71 | 133 | 268 | 197 | 171 |
| SM 42:3;2O | 480 | 284 | 653 | 392 | 278 | 469 | 441 | 722 | 465 |
| SM 43:2;2O|SM 18:1;2O/25:1 | 1204 | 1053 | 1326 | 901 | 816 | 799 | 532 | 1106 | 967 |
| SM 44:8;2O | 251 | 317 | 108 | 38 | 92 | 37 | 44 | 21 | 113 |
| **Triacylglycrols** |  |  |  |  |  |  |  |  |  |
| TG 46:0|TG 14:0_16:0_16:0 | 21466 | 28093 | 25383 | 21476 | 37964 | 12370 | 29045 | 26064 | 25233 |
| TG 48:1|TG 16:0_16:0_16:1 | 95690 | 74409 | 63431 | 86071 | 112465 | 56447 | 78211 | 72396 | 79890 |
| TG 49:3|TG 15:0_16:0_18:3 | 22996 | 13153 | 94045 | 41794 | 17248 | 17712 | 98163 | 40895 | 43251 |
| TG 50:2|TG 16:0_16:0_18:2 | 118620 | 70042 | 87360 | 50666 | 123771 | 46910 | 86328 | 49637 | 79167 |
| TG 51:2|TG 15:0_18:1_18:1 | 15453 | 19091 | 32368 | 11098 | 14953 | 11673 | 26273 | 10862 | 17721 |
| TG 51:4|TG 15:0_16:0_20:4 | 13503 | 7787 | 57551 | 25479 | 9552 | 12748 | 43415 | 23674 | 24214 |
| TG 52:2|TG 16:0_18:1_18:1 | 53175 | 72075 | 46353 | 37293 | 51670 | 39682 | 68524 | 39113 | 50986 |
| TG 52:3|TG 16:0_18:1_18:2 | 1833474 | 1646067 | 2231878 | 1294814 | 1817955 | 1026953 | 2597689 | 1624470 | 1759162 |
| TG 53:4|TG 17:1_18:1_18:2 | 17290 | 7154 | 50550 | 17069 | 16994 | 11804 | 23986 | 13188 | 19754 |
| TG 54:6|TG 18:1_18:2_18:3 | 31079 | 14114 | 68621 | 28033 | 21908 | 14700 | 74761 | 42981 | 37025 |
| TG 56:6|TG 16:0_18:1_22:5 | 35451 | 18247 | 84071 | 28792 | 34148 | 28412 | 62594 | 41176 | 41611 |
| TG 57:6|TG 17:0_18:1_22:5 | 1462 | 1170 | 5463 | 784 | 910 | 807 | 2724 | 1440 | 1845 |
| TG 10:0_10:0_20:5 | 45564 | 19887 | 56799 | 31251 | 17413 | 23034 | 25744 | 47145 | 33355 |
| TG 8:0_8:0_16:4 | 699 | 536 | 817 | 216 | 376 | 192 | 277 | 1070 | 523 |
| TG O-42:5|TG O-20:5_11:0_11:0 | 2542 | 6823 | 1464 | 747 | 2040 | 702 | 1673 | 1032 | 2128 |
| TG O-49:6|TG O-15:4_17:1_17:1 | 5312 | 7418 | 4895 | 4109 | 7743 | 2429 | 5667 | 4902 | 5309 |
| TG O-51:6|TG O-15:4_17:0_19:2 | 2928 | 5466 | 2169 | 2528 | 4160 | 1936 | 2499 | 2487 | 3022 |
| TG O-51:7|TG O-15:4_15:0_21:3 | 9334 | 12210 | 8529 | 10826 | 13406 | 8057 | 9084 | 9457 | 10113 |
| TG O-52:7|TG O-18:4_16:0_18:3 | 3160 | 5396 | 2766 | 1488 | 3014 | 1635 | 3152 | 1761 | 2796 |
| TG O-54:8|TG O-15:4_18:1_21:3 | 2757 | 3976 | 3653 | 1293 | 2210 | 1486 | 2967 | 1553 | 2487 |
| TG O-55:9|TG O-13:1_21:4_21:4 | 4556 | 8008 | 4560 | 2659 | 5378 | 2258 | 6346 | 3107 | 4609 |
| TG O-57:10|TG O-15:0_21:5_21:5 | 2794 | 3998 | 3732 | 1142 | 2568 | 1409 | 3392 | 1393 | 2553 |
| TG O-57:9|TG O-15:1_21:4_21:4 | 2638 | 5017 | 3382 | 969 | 1958 | 1369 | 5554 | 1993 | 2860 |
| **Others** |  |  |  |  |  |  |  |  |  |
| CoQ8 | 859 | 524 | 635 | 493 | 629 | 567 | 451 | 1362 | 690 |
| DGCC 17:2_22:6 | 1544 | 1076 | 6271 | 1498 | 921 | 1526 | 2235 | 3991 | 2383 |
| DGGA 36:2|DGGA 18:1_18:1 | 1482 | 575 | 1479 | 1756 | 3712 | 883 | 1768 | 2996 | 1831 |
| DGTS 21:0 | 290 | 307 | 885 | 350 | 136 | 386 | 606 | 614 | 447 |
| LPA 16:3 | 3041 | 2994 | 1228 | 1150 | 778 | 1647 | 1259 | 1484 | 1698 |
| LPE O-19:4 | 754 | 790 | 717 | 216 | 288 | 204 | 312 | 458 | 467 |

| LPE-N (FA)33:2|LPE-N (FA 15:0)18:2 | 835 | 712 | 1016 |  | 1125 |  | 765 | 587 | 1314 | 1596 | 994 |
| --- | --- | --- | --- | --- | --- | --- | --- | --- | --- | --- | --- |
| LPG O-13:0 | 1580 | 1960 | 1161 |  | 863 |  | 323 | 858 | 756 | 1057 | 1070 |
| NAGly 9:0;O | 2906 | 3349 | 573 |  | 763 |  | 441 | 163 | 528 | 464 | 1148 |
| Pentaerythritol tetrakis(3,5-di-tert-butyl-4-hydro | 27188 | 38761 | 24212 |  | 36636 |  | 5550 | 14874 | 17494 | 23410 | 23516 |
| PI O-16:0 | 920 | 932 | 1674 |  | 577 |  | 1488 | 1139 | 674 | 1295 | 1087 |
| PI-Cer 38:3;2O | 417 | 268 | 1071 |  | 504 |  | 224 | 466 | 941 | 1058 | 619 |
| SL 12:0;O/26:0 | 4130 | 3406 | 7327 |  | 2841 |  | 1237 | 4914 | 5844 | 4715 | 4302 |
| SL 12:1;O/32:0 | 4786 | 3924 | 6041 |  | 1661 |  | 1520 | 2946 | 9619 | 3578 | 4259 |
| ST 27:1;O;S - Cholesterol sulfate | 2608 | 854 | 2097 |  | 1838 |  | 1840 | 918 | 1714 | 1250 | 1640 |
| **Cotrol T28** |  |  |  |  |  |  |  |  |  |  |  |
| **Metabolite name** | **t28_C1** | **t28_C2** | **t28_C3** | **t28_C4** |  | **t28_C5** |  | **t28_C6** | **t28_C7** | **t28_C8** | **average value** |
| **Acyl sterol glycosides** |  |  |  |  |  |  |  |  |  |  |  |
| ASG 27:1;O;Hex;FA 2:0 | 631 | 1191 | 1077 |  | 868 |  | 1055 | 760 | 888 | 593 | 883 |
| ASG 28:1;O;Hex;FA 14:0 | 978 | 375 | 172 |  | 4660 |  | 3156 | 671 | 751 | 3554 | 1790 |
| ASG 28:1;O;Hex;FA 16:0 | 611 | 321 | 102 |  | 4132 |  | 2758 | 593 | 619 | 3008 | 1518 |
| ASG 28:1;O;Hex;FA 18:0 | 429 | 342 | 128 |  | 3376 |  | 2282 | 368 | 503 | 2068 | 1187 |
| ASG 28:1;O;Hex;FA 20:1 | 467 | 393 | 122 |  | 2657 |  | 1960 | 365 | 550 | 1294 | 976 |
| ASG 28:2;O;Hex;FA 14:0 | 1307 | 768 | 348 |  | 6091 |  | 4850 | 1108 | 1187 | 3840 | 2437 |
| ASG 28:2;O;Hex;FA 16:0 | 878 | 388 | 205 |  | 3362 |  | 2466 | 603 | 626 | 2380 | 1363 |
| ASG 28:2;O;Hex;FA 16:1 | 454 | 190 | 85 |  | 1611 |  | 1151 | 312 | 245 | 761 | 601 |
| ASG 28:2;O;Hex;FA 20:1 | 366 | 235 | 79 |  | 1411 |  | 957 | 273 | 295 | 712 | 541 |
| ASG 29:2;O;Hex;FA 20:5 | 382 | 699 | 592 |  | 1612 |  | 1088 | 401 | 615 | 704 | 762 |
| **Carnitines** |  |  |  |  |  |  |  |  |  |  |  |
| CAR 13:0 | 682 | 569 | 291 |  | 848 |  | 648 | 708 | 780 | 512 | 630 |
| CAR 4:0 | 3581 | 6905 | 5791 |  | 2571 |  | 11420 | 4906 | 11485 | 10000 | 7082 |
| CAR 5:0 | 2364 | 4257 | 9683 |  | 4104 |  | 19755 | 5709 | 10875 | 18295 | 9380 |
| CAR 21:2 | 2019 | 1968 | 1117 |  | 6626 |  | 5566 | 1765 | 3173 | 3628 | 3233 |
| CAR 21:4 | 1893 | 3244 | 1859 |  | 614 |  | 4468 | 4142 | 1721 | 8422 | 3295 |
| **Ceramides** |  |  |  |  |  |  |  |  |  |  |  |
| Cer 20:2;4O|Cer 12:2;3O/8:0;(2OH) | 1906 | 2626 | 808 |  | 732 |  | 1829 | 1021 | 5107 | 1065 | 1887 |
| Cer 24:3;3O|Cer 15:3;2O/9:0;O | 1918 | 2562 | 1912 |  | 1373 |  | 2271 | 2117 | 3062 | 2097 | 2164 |
| Cer 32:0;2O|Cer 16:0;2O/16:0 | 619 | 626 | 360 |  | 1043 |  | 939 | 424 | 718 | 740 | 684 |
| Cer 32:1;2O|Cer 16:1;2O/16:0 | 473 | 417 | 214 |  | 904 |  | 649 | 383 | 455 | 846 | 543 |
| Cer 33:1;2O|Cer 17:1;2O/16:0 | 155 | 194 | 98 |  | 369 |  | 377 | 109 | 204 | 501 | 251 |
| Cer 33:1;4O|Cer 18:0;3O/15:1;(2OH) | 1439 | 1007 | 579 |  | 1368 |  | 2063 | 1103 | 1909 | 3475 | 1618 |
| Cer 34:1;2O|Cer 18:1;2O/16:0 | 1486 | 2144 | 887 |  | 3454 |  | 2609 | 2202 | 1501 | 4156 | 2305 |
| Cer 34:1;2O|Cer 18:1;2O/16:0 | 391 | 410 | 236 |  | 893 |  | 779 | 561 | 345 | 763 | 547 |
| Cer 34:1;3O|Cer 19:0;2O/15:1;O | 1755 | 1423 | 1700 |  | 2915 |  | 3261 | 2638 | 1582 | 2649 | 2240 |
| Cer 34:3;2O|Cer 12:2;2O/22:1 | 857 | 757 | 570 |  | 1710 |  | 1661 | 742 | 1477 | 1010 | 1098 |
| Cer 36:0;3O|Cer 18:0;2O/18:0;O | 12261 | 14563 | 11420 |  | 10452 |  | 14042 | 13197 | 16058 | 14604 | 13325 |
| Cer 36:1;3O|Cer 19:0;2O/17:1;O | 454 | 572 | 297 |  | 855 |  | 706 | 636 | 565 | 709 | 599 |
| Cer 36:3;4O|Cer 19:2;3O/17:1;(2OH) | 1353 | 1703 | 1480 |  | 2300 |  | 2176 | 1585 | 1803 | 2077 | 1810 |
| Cer 37:0;2O|Cer 19:0;2O/18:0 | 1154 | 1947 | 664 |  | 2103 |  | 2203 | 529 | 3533 | 824 | 1620 |
| Cer 38:0;4O | 826 | 603 | 675 |  | 662 |  | 459 | 209 | 961 | 450 | 606 |

| Cer 39:0;3O|Cer 18:0;2O/21:0;O | 4237 | 3685 | 1135 | 4554 | 3590 | 1058 | 3300 | 1865 | 2928 |
| --- | --- | --- | --- | --- | --- | --- | --- | --- | --- |
| Cer 40:0;2O|Cer 16:0;2O/24:0 | 763 | 964 | 514 | 1371 | 1209 | 541 | 701 | 793 | 857 |
| Cer 40:1;2O|Cer 16:1;2O/24:0 | 1485 | 1524 | 889 | 2860 | 1930 | 1498 | 2100 | 2307 | 1824 |
| Cer 40:1;2O|Cer 16:1;2O/24:0 | 891 | 770 | 453 | 1182 | 951 | 707 | 984 | 1215 | 894 |
| Cer 41:1;2O|Cer 18:1;2O/23:0 | 1200 | 1115 | 541 | 1521 | 1741 | 695 | 868 | 4189 | 1484 |
| Cer 42:1;2O|Cer 18:1;2O/24:0 | 1539 | 1215 | 711 | 3310 | 4260 | 1929 | 1558 | 3883 | 2301 |
| Cer 42:1;2O|Cer 18:1;2O/24:0 | 520 | 457 | 323 | 1401 | 1544 | 781 | 775 | 1369 | 896 |
| Cer 42:1;2O|Cer 18:1;2O/24:0 | 597 | 448 | 244 | 1785 | 1158 | 430 | 396 | 992 | 756 |
| Cer 44:2;4O|Cer 28:1;3O(FA 16:0) | 917 | 799 | 461 | 3713 | 2747 | 1094 | 1074 | 2858 | 1708 |
| Cer 12:0;2O/25:0 | 2287 | 2493 | 1973 | 6282 | 4481 | 2220 | 3715 | 2874 | 3291 |
| Cer 12:2;2O/19:4 | 604 | 469 | 438 | 2112 | 1955 | 537 | 1624 | 2077 | 1227 |
| Cer 13:2;2O/30:3 | 2914 | 2386 | 1385 | 3684 | 4546 | 2965 | 2274 | 3167 | 2915 |
| **Diacylglycerols** |  |  |  |  |  |  |  |  |  |
| DG 16:0 | 880 | 593 | 499 | 1217 | 1436 | 667 | 713 | 1791 | 975 |
| DG 18:0 | 3431 | 1732 | 1200 | 5043 | 6614 | 2964 | 1241 | 6394 | 3577 |
| DG 20:0 | 20822 | 7928 | 6889 | 39679 | 46896 | 23509 | 6083 | 72047 | 27982 |
| DG 21:0 | 338 | 220 | 124 | 705 | 751 | 280 | 297 | 663 | 422 |
| DG 22:0 | 6277 | 3920 | 3650 | 25521 | 29682 | 10083 | 4436 | 37088 | 15082 |
| DG 22:0|DG 10:0_12:0 | 376 | 289 | 422 | 1453 | 1733 | 621 | 796 | 2564 | 1032 |
| DG 22:0|DG 8:0_14:0 | 406 | 324 | 437 | 1596 | 1690 | 662 | 707 | 2400 | 1028 |
| DG 22:1 | 3313 | 1163 | 872 | 4763 | 5566 | 2029 | 1192 | 4903 | 2975 |
| DG 22:1|DG 8:0_14:1 | 2958 | 1311 | 1242 | 5997 | 6202 | 3504 | 1736 | 6434 | 3673 |
| DG 24:0 | 3720 | 3019 | 4566 | 18276 | 19528 | 6773 | 7443 | 21179 | 10563 |
| DG 24:0|DG 10:0_14:0 | 1704 | 1409 | 2206 | 8617 | 8750 | 2983 | 2701 | 9422 | 4724 |
| DG 24:0|DG 8:0_16:0 | 1692 | 1255 | 1941 | 8432 | 10426 | 3453 | 2579 | 10329 | 5013 |
| DG 24:1 | 4188 | 2572 | 2226 | 14039 | 16132 | 5460 | 2891 | 14637 | 7768 |
| DG 24:1|DG 10:0_14:1 | 2469 | 1473 | 1565 | 7619 | 8981 | 3000 | 1989 | 8451 | 4443 |
| DG 24:2 | 338 | 286 | 242 | 1266 | 1405 | 384 | 350 | 1120 | 674 |
| DG 24:3 | 240 | 152 | 143 | 643 | 603 | 197 | 217 | 830 | 378 |
| DG 25:0|DG 10:0_15:0 | 579 | 377 | 479 | 1778 | 2076 | 652 | 1184 | 1860 | 1123 |
| DG 26:0|DG 12:0_14:0 | 4404 | 3976 | 4555 | 19976 | 20915 | 7863 | 7063 | 21693 | 11306 |
| DG 26:1 | 2946 | 2457 | 2521 | 12251 | 13384 | 4931 | 3496 | 13097 | 6885 |
| DG 26:1|DG 8:0_18:1 | 2462 | 2003 | 2276 | 11066 | 11946 | 5143 | 2667 | 13047 | 6326 |
| DG 26:2 | 275 | 261 | 238 | 1330 | 1309 | 392 | 512 | 1195 | 689 |
| DG 26:2|DG 8:0_18:2 | 303 | 205 | 213 | 1122 | 1269 | 384 | 420 | 1109 | 628 |
| DG 26:3 | 185 | 128 | 136 | 766 | 850 | 233 | 212 | 810 | 415 |
| DG 27:0 | 485 | 402 | 358 | 1378 | 1207 | 391 | 1045 | 1090 | 794 |
| DG 27:5 | 568 | 450 | 387 | 1474 | 1508 | 529 | 1678 | 1338 | 992 |
| DG 28:0|DG 12:0_16:0 | 11362 | 9471 | 9436 | 46883 | 43871 | 18470 | 20441 | 42017 | 25244 |
| DG 28:1 | 5074 | 4398 | 2688 | 13101 | 13515 | 4647 | 6089 | 9675 | 7398 |
| DG 28:1|DG 10:0_18:1 | 8261 | 5600 | 6773 | 33404 | 34823 | 13297 | 8753 | 34741 | 18207 |
| DG 28:2 | 1720 | 1666 | 866 | 5339 | 4787 | 1310 | 2048 | 4125 | 2733 |
| DG 28:2|DG 14:1_14:1 | 2029 | 1446 | 1701 | 9669 | 10085 | 3325 | 2534 | 10901 | 5211 |
| DG 28:3 | 273 | 233 | 269 | 1338 | 1043 | 319 | 407 | 1192 | 634 |
| DG 28:3|DG 10:0_18:3 | 336 | 248 | 277 | 1528 | 1510 | 491 | 460 | 1926 | 847 |
| DG 30:0 | 414 | 349 | 333 | 1447 | 1362 | 480 | 398 | 1064 | 731 |

| DG 30:0|DG 14:0_16:0 | 39890 | 49325 | 37011 | 157835 | 142374 | 67652 | 64469 | 99109 | 82208 |
| --- | --- | --- | --- | --- | --- | --- | --- | --- | --- |
| DG 30:1|DG 16:0_14:1 | 17361 | 17857 | 15205 | 105969 | 75784 | 49869 | 21401 | 70768 | 46777 |
| DG 30:2 | 945 | 848 | 451 | 3000 | 3081 | 647 | 1812 | 1678 | 1558 |
| DG 30:2|DG 12:0_18:2 | 1474 | 1247 | 1182 | 9560 | 7968 | 2364 | 2210 | 6869 | 4109 |
| DG 30:2|DG 14:1_16:1 | 1634 | 1228 | 916 | 7372 | 6211 | 2055 | 1574 | 6480 | 3434 |
| DG 30:3|DG 12:0_18:3 | 465 | 340 | 524 | 2482 | 2696 | 810 | 740 | 2566 | 1328 |
| DG 30:5 | 209 | 206 | 54 | 189 | 167 | 56 | 292 | 101 | 159 |
| DG 30:6 | 2635 | 2046 | 1006 | 5795 | 5566 | 1546 | 7715 | 3884 | 3774 |
| DG 30:7 | 311 | 213 | 129 | 702 | 632 | 160 | 694 | 583 | 428 |
| DG 31:7 | 27176 | 16958 | 7104 | 35605 | 27877 | 8623 | 37301 | 16029 | 22084 |
| DG 31:8 | 1398 | 1018 | 577 | 3422 | 2758 | 775 | 2740 | 1603 | 1786 |
| DG 32:1|DG 14:0_18:1 | 41506 | 53385 | 35475 | 171365 | 188055 | 97878 | 60073 | 133347 | 97635 |
| DG 32:2 | 4383 | 3819 | 2054 | 9821 | 8076 | 3213 | 4847 | 4431 | 5081 |
| DG 32:2|DG 14:1_18:1 | 9238 | 7647 | 6587 | 45380 | 41474 | 14139 | 11007 | 29243 | 20589 |
| DG 32:3 | 843 | 690 | 358 | 1465 | 1408 | 503 | 996 | 810 | 884 |
| DG 32:3|DG 14:0_18:3 | 1664 | 1592 | 1590 | 7611 | 6652 | 2203 | 2315 | 5746 | 3672 |
| DG 32:6 | 3851 | 3301 | 2047 | 7487 | 5047 | 1953 | 12820 | 5587 | 5262 |
| DG 32:7 | 928 | 713 | 329 | 1866 | 1440 | 407 | 1818 | 1327 | 1104 |
| DG 33:2 | 4141 | 2258 | 7657 | 2950 | 2685 | 3957 | 4473 | 4477 | 4075 |
| DG 33:3 | 2953 | 1617 | 3958 | 1392 | 1421 | 2592 | 3011 | 2623 | 2446 |
| DG 33:6 | 43349 | 32816 | 9066 | 35543 | 38995 | 13858 | 89191 | 20766 | 35448 |
| DG 33:8 | 3779 | 2374 | 1177 | 8355 | 7844 | 1537 | 6822 | 4270 | 4520 |
| DG 33:9 | 679 | 201 | 305 | 1225 | 1012 | 272 | 762 | 826 | 660 |
| DG 34:0 | 242 | 251 | 179 | 425 | 497 | 276 | 498 | 494 | 358 |
| DG 34:0|DG 16:0_18:0 | 4826 | 5958 | 4711 | 15238 | 9918 | 6325 | 9173 | 13407 | 8694 |
| DG 34:1|DG 16:0_18:1 | 79170 | 85265 | 70756 | 223698 | 207702 | 118098 | 113340 | 157252 | 131910 |
| DG 34:2 | 195 | 409 | 87 | 257 | 257 | 147 | 637 | 158 | 268 |
| DG 34:2|DG 16:0_18:2 | 25232 | 22524 | 18087 | 137821 | 116477 | 46560 | 31484 | 80949 | 59892 |
| DG 34:3|DG 16:0_18:3 | 4022 | 3228 | 2864 | 22775 | 22167 | 7300 | 6921 | 21821 | 11387 |
| DG 34:6 | 4570 | 3339 | 1790 | 7038 | 5060 | 2465 | 9087 | 5595 | 4868 |
| DG 34:7 | 1930 | 1593 | 738 | 3944 | 3265 | 1145 | 4289 | 2541 | 2431 |
| DG 35:2 | 2174 | 1223 | 3592 | 1720 | 1677 | 2060 | 2706 | 2653 | 2226 |
| DG 35:3 | 1798 | 1000 | 2959 | 1167 | 997 | 1323 | 1813 | 1503 | 1570 |
| DG 35:8 | 14823 | 11049 | 4092 | 21342 | 16991 | 5245 | 20725 | 11235 | 13188 |
| DG 35:9 | 2813 | 1670 | 1145 | 7171 | 6843 | 1018 | 8189 | 4287 | 4142 |
| DG 36:1|DG 18:0_18:1 | 6791 | 6251 | 5168 | 10067 | 5419 | 4704 | 9906 | 6773 | 6885 |
| DG 36:2|DG 18:1_18:1 | 27679 | 32307 | 27988 | 76226 | 60872 | 30966 | 46081 | 38503 | 42578 |
| DG 36:3|DG 18:1_18:2 | 9674 | 7661 | 6596 | 38571 | 29493 | 10795 | 13481 | 21440 | 17214 |
| DG 36:4|DG 18:1_18:3 | 2330 | 1760 | 1820 | 8673 | 7512 | 2557 | 3895 | 7078 | 4453 |
| DG 36:5|DG 16:0_20:5 | 342 | 274 | 201 | 1807 | 1467 | 520 | 542 | 1518 | 834 |
| DG 36:5|DG 18:2_18:3 | 475 | 469 | 499 | 2507 | 2051 | 682 | 992 | 2433 | 1263 |
| DG 36:7 | 3698 | 2478 | 1042 | 4766 | 3670 | 1627 | 4863 | 3174 | 3165 |
| DG 36:8 | 1337 | 782 | 1326 | 746 | 636 | 625 | 1759 | 858 | 1009 |
| DG 37:9 | 4756 | 3086 | 1838 | 11164 | 9472 | 2019 | 11026 | 7465 | 6353 |
| DG 38:5|DG 16:0_22:5 | 3050 | 2844 | 2295 | 11425 | 9740 | 5173 | 4307 | 13422 | 6532 |
| DG 38:6 | 190 | 212 | 89 | 416 | 317 | 142 | 472 | 182 | 252 |

| DG 40:6|DG 18:1_22:5 | 727 | 656 | 482 | 1880 | 1648 | 767 | 1199 | 1831 | 1149 |
| --- | --- | --- | --- | --- | --- | --- | --- | --- | --- |
| DG 40:8 | 1059 | 705 | 1256 | 780 | 631 | 569 | 1676 | 678 | 919 |
| DG 41:10 | 866 | 307 | 170 | 1066 | 681 | 271 | 714 | 709 | 598 |
| DG 41:11 | 351 | 284 | 119 | 497 | 392 | 155 | 378 | 335 | 314 |
| DG 41:5 | 1819 | 1943 | 962 | 3203 | 2352 | 1268 | 2984 | 1959 | 2061 |
| DG 41:6 | 13894 | 14653 | 4804 | 35357 | 13339 | 10969 | 10646 | 13660 | 14665 |
| DG 43:11 | 747 | 560 | 273 | 1088 | 985 | 322 | 1039 | 502 | 690 |
| DG 43:3 | 513 | 414 | 184 | 676 | 438 | 208 | 677 | 351 | 433 |
| DG 43:4 | 2163 | 1299 | 687 | 2075 | 2000 | 916 | 2859 | 1655 | 1707 |
| DG 43:6 | 10734 | 10056 | 5417 | 14563 | 11776 | 4119 | 13829 | 12462 | 10369 |
| DG 43:7 | 8804 | 4827 | 5324 | 9136 | 11178 | 9281 | 8274 | 10569 | 8424 |
| DG 44:7 | 708 | 653 | 693 | 1384 | 1655 | 1033 | 931 | 1421 | 1060 |
| DG 44:9 | 992 | 866 | 701 | 3027 | 2439 | 1792 | 723 | 3151 | 1711 |
| DG 46:6 | 3957 | 4557 | 4143 | 3484 | 3924 | 4311 | 3342 | 4237 | 3994 |
| DG 47:7 | 6812 | 8153 | 5153 | 17827 | 14088 | 6898 | 12457 | 5734 | 9640 |
| DG 47:8 | 7947 | 5438 | 3331 | 9867 | 6155 | 4297 | 10673 | 5619 | 6666 |
| DG 51:7 | 1966 | 1976 | 856 | 4809 | 3206 | 2233 | 2466 | 3187 | 2587 |
| DG 51:8 | 1119 | 1015 | 639 | 2285 | 2399 | 1107 | 1659 | 2242 | 1558 |
| **Free Fatty Acids** |  |  |  |  |  |  |  |  |  |
| FA 12:0 | 843 | 766 | 559 | 963 | 1092 | 781 | 778 | 1452 | 904 |
| FA 14:0 | 21781 | 10367 | 13073 | 10434 | 12048 | 16158 | 8496 | 16763 | 13640 |
| FA 14:1 | 2157 | 627 | 723 | 2030 | 1968 | 1535 | 265 | 3889 | 1649 |
| FA 15:0 | 836 | 765 | 518 | 936 | 974 | 788 | 618 | 879 | 789 |
| FA 15:4 | 871 | 1826 | 1120 | 2446 | 1711 | 899 | 2544 | 1239 | 1582 |
| FA 16:0 | 634107 | 711023 | 596244 | 812186 | 918401 | 650021 | 582376 | 673299 | 697207 |
| FA 16:0;(2OH) | 830 | 591 | 885 | 1112 | 876 | 498 | 464 | 1246 | 813 |
| FA 16:0;O | 2324 | 3279 | 2664 | 3953 | 3199 | 2512 | 2394 | 2817 | 2893 |
| FA 16:1 | 17207 | 6654 | 6953 | 13613 | 9963 | 14970 | 3394 | 22693 | 11931 |
| FA 16:1;O | 2628 | 3886 | 3179 | 4143 | 3674 | 2712 | 3016 | 3208 | 3306 |
| FA 17:0 | 1695 | 1673 | 1347 | 1969 | 2268 | 1500 | 1318 | 1726 | 1687 |
| FA 17:1 | 164 | 113 | 75 | 196 | 174 | 130 | 123 | 174 | 144 |
| FA 17:4;O | 1557 | 2184 | 1229 | 1165 | 1560 | 667 | 1435 | 1672 | 1434 |
| FA 18:0 | 654267 | 823008 | 628445 | 890095 | 1044621 | 639490 | 669051 | 705022 | 756750 |
| FA 18:0;(2OH) | 2982 | 4679 | 3455 | 3747 | 2569 | 2513 | 4153 | 3120 | 3402 |
| FA 18:0;O | 1612 | 2023 | 1667 | 3387 | 2326 | 1614 | 1560 | 2088 | 2035 |
| FA 18:1 | 33786 | 21380 | 17479 | 52202 | 38406 | 24929 | 18673 | 42227 | 31135 |
| FA 18:1;2O | 417 | 763 | 299 | 647 | 593 | 317 | 556 | 325 | 490 |
| FA 18:1;O | 963 | 1438 | 912 | 2257 | 1702 | 811 | 1249 | 1556 | 1361 |
| FA 18:2 | 2310 | 1204 | 1173 | 3162 | 2445 | 1332 | 1313 | 2856 | 1974 |
| FA 18:2;O | 1928 | 3823 | 2082 | 3218 | 3034 | 1937 | 2185 | 2021 | 2529 |
| FA 18:3 | 5348 | 1043 | 2691 | 4775 | 3237 | 3374 | 1054 | 7866 | 3674 |
| FA 18:3;4O | 533 | 1145 | 607 | 892 | 695 | 444 | 1110 | 533 | 745 |
| FA 18:3;O | 392 | 819 | 427 | 591 | 478 | 315 | 314 | 320 | 457 |
| FA 19:0 | 438 | 412 | 336 | 648 | 622 | 438 | 429 | 391 | 464 |
| FA 19:0;(2OH) | 950 | 1082 | 1104 | 1083 | 929 | 1033 | 792 | 976 | 994 |
| FA 19:1 | 261 | 105 | 92 | 181 | 146 | 139 | 27 | 196 | 143 |

| FA 19:1;2O | 407 | 682 | 548 | 932 | 665 | 358 | 708 | 459 | 595 |
| --- | --- | --- | --- | --- | --- | --- | --- | --- | --- |
| FA 19:1;O | 208 | 92 | 65 | 287 | 172 | 127 | 47 | 306 | 163 |
| FA 20:0 | 7198 | 8863 | 6365 | 11177 | 11870 | 6482 | 7512 | 7037 | 8313 |
| FA 20:1 | 418 | 326 | 329 | 596 | 388 | 306 | 227 | 436 | 378 |
| FA 20:3 | 183 | 144 | 127 | 353 | 232 | 166 | 191 | 369 | 221 |
| FA 20:4 | 3530 | 1053 | 2318 | 3114 | 2400 | 1910 | 1394 | 5457 | 2647 |
| FA 20:5 | 745 | 1723 | 10653 | 1182 | 573 | 370 | 1644 | 933 | 2228 |
| FA 21:0 | 796 | 856 | 791 | 1377 | 1272 | 663 | 534 | 828 | 890 |
| FA 21:1;2O | 321 | 666 | 355 | 587 | 324 | 229 | 587 | 254 | 415 |
| FA 22:0 | 2189 | 3095 | 1970 | 4143 | 4161 | 1834 | 2735 | 1916 | 2755 |
| FA 22:1 | 145 | 202 | 139 | 356 | 266 | 124 | 84 | 148 | 183 |
| FA 22:5 | 547 | 374 | 445 | 1213 | 854 | 450 | 756 | 2524 | 895 |
| FA 22:6 | 1593 | 1182 | 1838 | 1895 | 1360 | 1152 | 1171 | 2960 | 1644 |
| FA 23:0 | 1155 | 1827 | 1011 | 2261 | 1995 | 1064 | 1363 | 936 | 1452 |
| FA 23:1 | 184 | 128 | 147 | 392 | 240 | 142 | 147 | 452 | 229 |
| FA 24:0 | 6804 | 10387 | 6577 | 14562 | 12743 | 6282 | 9271 | 6182 | 9101 |
| FA 25:0 | 2486 | 4380 | 2441 | 6392 | 5120 | 2593 | 3427 | 2749 | 3699 |
| FA 26:0 | 14506 | 25656 | 14547 | 34063 | 29207 | 13979 | 22440 | 15265 | 21208 |
| FA 26:1;O | 1309 | 1762 | 1419 | 2992 | 1685 | 1161 | 1149 | 1155 | 1579 |
| FA 27:0 | 5353 | 6942 | 4688 | 14286 | 11507 | 5958 | 6912 | 7822 | 7933 |
| FA 27:1;O | 407 | 593 | 429 | 1038 | 652 | 449 | 336 | 382 | 536 |
| FA 28:0 | 22603 | 34448 | 19604 | 57926 | 45055 | 20902 | 35105 | 25065 | 32588 |
| FA 29:0 | 6638 | 10243 | 5909 | 16464 | 12858 | 5706 | 9434 | 7131 | 9298 |
| FA 30:0 | 42230 | 51352 | 27937 | 107581 | 93649 | 36953 | 51991 | 55928 | 58453 |
| FA 31:0 | 4745 | 8542 | 4348 | 14516 | 10677 | 4576 | 8797 | 5183 | 7673 |
| FA 32:0 | 17446 | 25918 | 12776 | 48482 | 34943 | 14909 | 28262 | 20020 | 25344 |
| FA 33:0 | 2650 | 3427 | 2079 | 7191 | 5598 | 2015 | 3848 | 3142 | 3744 |
| FA 34:0 | 4941 | 9204 | 4039 | 14799 | 12183 | 4317 | 9203 | 5312 | 8000 |
| FA 34:8 | 2098 | 1215 | 1010 | 7937 | 4404 | 1571 | 1722 | 4530 | 3061 |
| FA 42:5 | 1205873 | 2929130 | 1772893 | 4538064 | 3399403 | 1143713 | 3779994 | 1296055 | 2508140 |
| FA 42:6 | 1298 | 2768 | 1654 | 4002 | 2510 | 1596 | 2805 | 1197 | 2229 |
| FA 44:5 | 471632 | 789814 | 557871 | 1682089 | 1420716 | 584943 | 1138271 | 525657 | 896374 |
| FA 44:6 | 873 | 1715 | 1043 | 3391 | 2519 | 1434 | 1756 | 1335 | 1758 |
| **Dihexosylceramides** |  |  |  |  |  |  |  |  |  |
| Hex2Cer 32:1;2O|Hex2Cer 16:1;2O/16:0 | 198 | 172 | 83 | 156 | 220 | 147 | 155 | 167 | 162 |
| Hex2Cer 33:1;2O|Hex2Cer 17:1;2O/16:0 | 167 | 148 | 99 | 204 | 190 | 164 | 111 | 144 | 153 |
| Hex2Cer 34:0;2O | 597 | 670 | 334 | 1185 | 865 | 742 | 434 | 1061 | 736 |
| Hex2Cer 34:1;2O|Hex2Cer 18:1;2O/16:0 | 4059 | 5141 | 2086 | 8258 | 6947 | 6879 | 2885 | 9171 | 5678 |
| Hex2Cer 36:1;2O|Hex2Cer 18:1;2O/18:0 | 104 | 179 | 77 | 223 | 118 | 117 | 87 | 121 | 128 |
| Hex2Cer 38:1;2O|Hex2Cer 16:1;2O/22:0 | 790 | 821 | 519 | 1114 | 949 | 575 | 604 | 483 | 732 |
| Hex2Cer 39:0;2O|Hex2Cer 17:0;2O/22:0 | 734 | 848 | 482 | 628 | 573 | 432 | 334 | 446 | 560 |
| Hex2Cer 39:1;2O|Hex2Cer 16:1;2O/23:0 | 1172 | 1330 | 770 | 1051 | 1077 | 762 | 848 | 380 | 924 |
| Hex2Cer 40:1;2O|Hex2Cer 18:1;2O/22:0 | 1757 | 2475 | 1375 | 2640 | 2321 | 1769 | 1495 | 1631 | 1933 |
| Hex2Cer 40:2;2O|Hex2Cer 16:1;2O/24:1 | 132 | 135 | 92 | 110 | 118 | 148 | 60 | 91 | 110 |
| Hex2Cer 41:1;2O|Hex2Cer 18:1;2O/23:0 | 1123 | 1437 | 467 | 1631 | 1088 | 1085 | 1390 | 501 | 1090 |
| Hex2Cer 42:1;2O|Hex2Cer 18:1;2O/24:0 | 1385 | 1215 | 765 | 1271 | 1186 | 892 | 897 | 660 | 1034 |

| Hex2Cer 42:2;2O|Hex2Cer 18:1;2O/24:1 | 170 | 205 | 130 | 321 | 179 | 385 | 77 | 177 | 206 |
| --- | --- | --- | --- | --- | --- | --- | --- | --- | --- |
| HexCer 33:1;2O|HexCer 17:1;2O/16:0 | 322 | 360 | 379 | 717 | 750 | 299 | 454 | 468 | 469 |
| HexCer 34:0;2O|HexCer 18:0;2O/16:0 | 222 | 190 | 133 | 475 | 404 | 145 | 241 | 402 | 276 |
| HexCer 34:1;2O | 729 | 837 | 720 | 1903 | 2073 | 663 | 557 | 840 | 1040 |
| HexCer 34:1;2O|HexCer 18:1;2O/16:0 | 726 | 876 | 622 | 2252 | 1407 | 699 | 977 | 1258 | 1102 |
| HexCer 34:1;2O|HexCer 18:1;2O/16:0 | 1071 | 1012 | 901 | 3007 | 2738 | 1138 | 1180 | 1935 | 1623 |
| HexCer 34:1;3O|HexCer 18:1;2O/16:0;O | 349 | 168 | 302 | 853 | 770 | 371 | 125 | 680 | 452 |
| HexCer 38:1;2O | 308 | 148 | 328 | 448 | 447 | 148 | 109 | 194 | 266 |
| HexCer 39:1;3O | 803 | 590 | 577 | 1756 | 1532 | 875 | 278 | 894 | 913 |
| HexCer 40:0;3O | 469 | 105 | 131 | 799 | 718 | 406 | 22 | 560 | 401 |
| HexCer 40:1;2O | 3294 | 1211 | 1794 | 6666 | 6460 | 2009 | 1458 | 5320 | 3526 |
| HexCer 40:1;2O|HexCer 18:1;2O/22:0 | 752 | 901 | 592 | 1484 | 871 | 432 | 714 | 412 | 770 |
| HexCer 40:1;3O | 1221 | 909 | 788 | 3428 | 3262 | 1249 | 385 | 1844 | 1636 |
| HexCer 41:1;2O | 1833 | 1129 | 1205 | 3019 | 3529 | 1015 | 1405 | 2559 | 1962 |
| HexCer 41:1;2O|HexCer 18:1;2O/23:0 | 1603 | 2008 | 1462 | 4091 | 3699 | 1936 | 3289 | 2700 | 2598 |
| HexCer 41:1;2O|HexCer 18:1;2O/23:0 | 722 | 1058 | 924 | 2002 | 1911 | 728 | 1652 | 915 | 1239 |
| HexCer 41:1;3O | 2398 | 945 | 1143 | 8577 | 7712 | 3433 | 728 | 6480 | 3927 |
| HexCer 42:1;2O | 4469 | 1973 | 2380 | 9171 | 9661 | 2958 | 1954 | 4946 | 4689 |
| HexCer 42:1;3O | 1673 | 986 | 907 | 5125 | 5434 | 2571 | 700 | 4554 | 2744 |
| HexCer 43:1;3O | 781 | 260 | 285 | 1575 | 1582 | 692 | 137 | 985 | 787 |
| HexCer 49:5;4O | 2870 | 3271 | 1456 | 2334 | 2037 | 1318 | 1215 | 1188 | 1961 |
| HexCer 51:9;3O | 730 | 1016 | 266 | 601 | 599 | 570 | 752 | 155 | 586 |
| HexCer 53:9;3O | 699 | 872 | 297 | 718 | 590 | 411 | 422 | 390 | 550 |
| **Lysophophatidylcholines** |  |  |  |  |  |  |  |  |  |
| LPC 14:0 | 1449 | 1015 | 1698 | 1377 | 2969 | 1513 | 1990 | 6365 | 2297 |
| LPC 15:0 | 1245 | 1002 | 1125 | 957 | 1654 | 1382 | 1333 | 3056 | 1469 |
| LPC 16:0 | 9302 | 6285 | 11189 | 10216 | 23614 | 19679 | 10710 | 76837 | 20979 |
| LPC 17:0 | 243 | 209 | 272 | 292 | 468 | 369 | 412 | 771 | 379 |
| LPC 18:0 | 663 | 659 | 1357 | 1884 | 2899 | 1446 | 1944 | 6154 | 2126 |
| LPC 18:1 | 3001 | 2522 | 3028 | 2752 | 6192 | 3868 | 3009 | 10060 | 4304 |
| LPC 18:2 | 1296 | 985 | 1388 | 1271 | 2192 | 1256 | 1666 | 2918 | 1622 |
| LPC 26:0 | 424 | 377 | 378 | 594 | 619 | 455 | 494 | 623 | 495 |
| LPC 28:7 | 524 | 174 | 411 | 512 | 502 | 506 | 366 | 1172 | 521 |
| **Lysophosphatidylethanolamines** |  |  |  |  |  |  |  |  |  |
| LPE 16:0 | 1386 | 1416 | 1064 | 4337 | 3720 | 2113 | 1605 | 5718 | 2670 |
| LPE 16:1 | 275 | 233 | 52 | 536 | 572 | 390 | 126 | 613 | 350 |
| LPE 18:0 | 1679 | 1153 | 1187 | 3331 | 2757 | 1649 | 1536 | 4955 | 2281 |
| LPE 18:1 | 6605 | 6688 | 4872 | 12745 | 11868 | 7217 | 5477 | 10977 | 8306 |
| LPE 18:2 | 1730 | 1529 | 1262 | 3088 | 3262 | 1435 | 1644 | 3005 | 2119 |
| LPE 18:3 | 1146 | 794 | 512 | 442 | 1205 | 503 | 1282 | 1227 | 889 |
| LPE 20:4 | 227 | 222 | 163 | 451 | 389 | 250 | 228 | 435 | 296 |
| **Lysophosphatidylinositols** |  |  |  |  |  |  |  |  |  |
| LPI 18:0 | 1911 | 1078 | 1318 | 5009 | 2246 | 834 | 2293 | 1996 | 2086 |
| LPI 18:1 | 1907 | 795 | 676 | 5168 | 1892 | 886 | 1926 | 2405 | 1957 |
| LPI 18:2 | 239 | 229 | 132 | 814 | 320 | 159 | 368 | 317 | 322 |
| **Monoacyl glycerols** |  |  |  |  |  |  |  |  |  |

| MG 15:0 | 579 | 478 | 215 | 716 | 556 | 605 | 658 | 446 | 531 |
| --- | --- | --- | --- | --- | --- | --- | --- | --- | --- |
| MG 17:3 | 634 | 1008 | 992 | 941 | 1513 | 857 | 2726 | 1398 | 1259 |
| MG 17:4 | 1841 | 2542 | 641 | 571 | 1501 | 977 | 4610 | 1114 | 1725 |
| MG 18:0 | 1222 | 2002 | 1062 | 2368 | 2851 | 1357 | 3696 | 2178 | 2092 |
| MG 19:3 | 365 | 529 | 372 | 272 | 536 | 335 | 967 | 498 | 484 |
| MG 21:4 | 1099 | 800 | 684 | 2470 | 2195 | 862 | 2165 | 1589 | 1483 |
| MG 22:1 | 521 | 492 | 195 | 1030 | 799 | 355 | 945 | 679 | 627 |
| MG 24:2 | 344 | 316 | 193 | 351 | 427 | 242 | 206 | 353 | 304 |
| MG 30:7 | 4621 | 4184 | 3937 | 13074 | 9606 | 3860 | 8657 | 4891 | 6604 |
| MG 32:8 | 808 | 563 | 352 | 1436 | 1322 | 548 | 1045 | 1076 | 894 |
| MG 34:8 | 1757 | 1363 | 629 | 2337 | 2052 | 706 | 2377 | 1324 | 1568 |
| MG 36:6 | 327 | 366 | 258 | 1294 | 1133 | 502 | 651 | 899 | 679 |
| **N-acyl ethanolamines** |  |  |  |  |  |  |  |  |  |
| NAE 12:0 | 3976 | 4943 | 3614 | 2520 | 5762 | 5083 | 4729 | 5150 | 4472 |
| NAE 13:1 | 7140 | 7048 | 5767 | 2328 | 6443 | 4841 | 12411 | 7736 | 6714 |
| NAE 14:1 | 10447 | 12743 | 8529 | 4401 | 11157 | 7771 | 21087 | 10283 | 10802 |
| NAE 15:1 | 1867 | 2360 | 1262 | 684 | 1887 | 1671 | 3155 | 1614 | 1812 |
| NAE 16:1 | 451879 | 723148 | 330547 | 202330 | 460883 | 398339 | 943295 | 348543 | 482370 |
| NAE 16:2 | 916290 | 1632732 | 584490 | 335141 | 980067 | 751997 | 2065430 | 635719 | 987733 |
| NAE 16:3 | 622511 | 945508 | 476825 | 228765 | 642802 | 615481 | 1074247 | 781322 | 673433 |
| NAE 16:4 | 31758 | 49831 | 19433 | 11483 | 27834 | 30413 | 70580 | 29908 | 33905 |
| NAE 17:4 | 5830 | 7268 | 5977 | 3554 | 8449 | 4784 | 11111 | 7451 | 6803 |
| NAE 18:1 | 19229 | 34055 | 13230 | 10295 | 18624 | 16793 | 42680 | 12400 | 20913 |
| NAE 18:2 | 28142 | 53405 | 19074 | 9957 | 25164 | 24384 | 60763 | 17390 | 29785 |
| NAE 18:3 | 3520 | 5117 | 3246 | 1732 | 3836 | 4149 | 6138 | 5900 | 4205 |
| NAE 18:4 | 94721 | 133764 | 63438 | 37533 | 107705 | 79417 | 173541 | 89684 | 97475 |
| NAE 18:5 | 158691 | 199847 | 85073 | 59070 | 165888 | 123311 | 246236 | 145751 | 147983 |
| NAE 20:1 | 1849 | 2710 | 1880 | 2390 | 2079 | 2017 | 4193 | 1788 | 2363 |
| NAE 20:2 | 4711 | 8831 | 3219 | 4255 | 5501 | 4871 | 10607 | 4270 | 5783 |
| NAE 20:4 | 5185 | 9269 | 3700 | 3051 | 4953 | 4568 | 10158 | 4394 | 5660 |
| NAE 20:5 | 4741 | 6986 | 2469 | 2207 | 4074 | 2752 | 7477 | 2761 | 4183 |
| NAE 22:3 | 2848 | 3290 | 3343 | 1395 | 3795 | 3274 | 2720 | 3842 | 3063 |
| NAE 22:4 | 1240 | 1547 | 930 | 1061 | 1171 | 969 | 2391 | 863 | 1271 |
| NAE 22:5 | 1275 | 1821 | 865 | 840 | 1294 | 1452 | 2417 | 1177 | 1393 |
| NAE 24:4 | 1233 | 1551 | 1053 | 984 | 1678 | 1022 | 2379 | 1220 | 1390 |
| NAE 5:0 | 2180 | 4096 | 2141 | 900 | 3656 | 1922 | 5691 | 3677 | 3033 |
| NAE 6:0 | 19689 | 42078 | 15172 | 7914 | 42261 | 24342 | 38518 | 23209 | 26648 |
| NAE 7:0 | 586 | 1013 | 581 | 133 | 1273 | 897 | 1053 | 1108 | 830 |
| **Phosphatidylcholines** |  |  |  |  |  |  |  |  |  |
| PC 24:0 | 280 | 155 | 514 | 1033 | 1219 | 645 | 479 | 1101 | 678 |
| PC 26:0|PC 12:0_14:0 | 945 | 833 | 1577 | 3535 | 3976 | 1861 | 1632 | 6461 | 2602 |
| PC 27:0|PC 12:0_15:0 | 198 | 201 | 182 | 592 | 607 | 287 | 362 | 804 | 404 |
| PC 28:0 | 472 | 341 | 413 | 960 | 1023 | 622 | 492 | 1099 | 678 |
| PC 28:0|PC 14:0_14:0 | 24602 | 21870 | 26206 | 64658 | 66491 | 47028 | 22270 | 83101 | 44528 |
| PC 28:1 | 350 | 237 | 252 | 802 | 946 | 326 | 307 | 864 | 511 |
| PC 29:0|PC 14:0_15:0 | 4967 | 3681 | 4060 | 10380 | 10947 | 8142 | 4173 | 13918 | 7533 |

| PC 30:0|PC 14:0_16:0 | 375301 | 354925 | 340713 | 934909 | 865405 | 616780 | 333195 | 905261 | 590811 |
| --- | --- | --- | --- | --- | --- | --- | --- | --- | --- |
| PC 30:1|PC 14:0_16:1 | 4774 | 3602 | 2712 | 12613 | 12770 | 7469 | 2902 | 12500 | 7418 |
| PC 30:3 | 285 | 225 | 261 | 549 | 607 | 409 | 251 | 561 | 393 |
| PC 31:0|PC 15:0_16:0 | 15862 | 16815 | 18035 | 36798 | 30024 | 26554 | 17696 | 35457 | 24655 |
| PC 31:1 | 1316 | 994 | 1102 | 3059 | 2649 | 1826 | 882 | 2574 | 1800 |
| PC 31:1|PC 15:0_16:1 | 1239 | 1020 | 842 | 2950 | 3146 | 1710 | 1028 | 2691 | 1828 |
| PC 32:0 | 5806 | 7182 | 6714 | 21744 | 19162 | 10836 | 5828 | 21975 | 12406 |
| PC 32:0|PC 16:0_16:0 | 306324 | 302169 | 285105 | 977103 | 831298 | 485502 | 324669 | 988643 | 562602 |
| PC 32:1 | 1516 | 969 | 761 | 2414 | 2071 | 1721 | 653 | 1452 | 1445 |
| PC 32:1|PC 14:0_18:1 | 129627 | 85636 | 67507 | 246996 | 226359 | 176220 | 42012 | 169064 | 142928 |
| PC 32:2|PC 16:1_16:1 | 5868 | 4711 | 4068 | 11920 | 11807 | 6650 | 4544 | 8307 | 7234 |
| PC 32:3|PC 14:0_18:3 | 597 | 510 | 501 | 1843 | 1747 | 682 | 650 | 1509 | 1005 |
| PC 32:3|PC 14:1_18:2 | 7023 | 6119 | 6568 | 19439 | 19614 | 14320 | 4340 | 21048 | 12309 |
| PC 33:0|PC 16:0_17:0 | 7733 | 6812 | 7125 | 12021 | 11859 | 8310 | 7287 | 6772 | 8490 |
| PC 33:1|PC 15:0_18:1 | 22054 | 16865 | 12338 | 29966 | 30009 | 21503 | 13631 | 26116 | 21560 |
| PC 33:2 | 1257 | 857 | 983 | 3228 | 2992 | 1382 | 1181 | 2699 | 1822 |
| PC 34:0|PC 16:0_18:0 | 38254 | 41077 | 60221 | 107940 | 89377 | 59005 | 51558 | 133504 | 72617 |
| PC 34:1 | 14281 | 14800 | 9637 | 29832 | 29928 | 17834 | 10084 | 24785 | 18898 |
| PC 34:1|PC 16:0_18:1 | 641495 | 594785 | 484369 | 1370082 | 1258626 | 753475 | 571951 | 1194683 | 858683 |
| PC 34:2 | 4249 | 3632 | 2817 | 6055 | 5413 | 2986 | 3081 | 3347 | 3947 |
| PC 34:2|PC 15:1_19:1 | 1008 | 861 | 725 | 1545 | 1387 | 771 | 971 | 1222 | 1061 |
| PC 34:2|PC 16:0_18:2 | 105856 | 105878 | 95503 | 200081 | 172546 | 129689 | 83705 | 123809 | 127133 |
| PC 34:3|PC 16:0_18:3 | 18835 | 15549 | 24208 | 79945 | 76552 | 39188 | 15092 | 80272 | 43705 |
| PC 34:4|PC 17:2_17:2 | 303 | 246 | 194 | 874 | 847 | 394 | 238 | 852 | 493 |
| PC 35:0|PC 17:0_18:0 | 1308 | 1297 | 1374 | 1857 | 1755 | 974 | 1645 | 1714 | 1490 |
| PC 35:1|PC 17:0_18:1 | 6279 | 7302 | 7804 | 7266 | 7864 | 6320 | 6770 | 6578 | 7023 |
| PC 35:2|PC 17:1_18:1 | 5820 | 4508 | 3799 | 8482 | 8106 | 5109 | 3790 | 6291 | 5738 |
| PC 35:3 | 991 | 599 | 729 | 2037 | 1651 | 841 | 706 | 1482 | 1130 |
| PC 36:0|PC 18:0_18:0 | 1071 | 876 | 1436 | 2456 | 1785 | 1206 | 2072 | 1842 | 1593 |
| PC 36:1 | 759 | 782 | 699 | 1509 | 1256 | 711 | 835 | 1394 | 993 |
| PC 36:1|PC 18:0_18:1 | 105378 | 98062 | 116584 | 218908 | 190639 | 93803 | 128554 | 289052 | 155122 |
| PC 36:2 | 1634 | 1095 | 879 | 1708 | 891 | 1024 | 779 | 948 | 1120 |
| PC 36:2|PC 18:1_18:1 | 123777 | 114294 | 75003 | 93888 | 86518 | 69645 | 47933 | 64005 | 84383 |
| PC 36:3 | 1436 | 1303 | 1199 | 1907 | 1907 | 1241 | 1149 | 1568 | 1464 |
| PC 36:3|PC 18:1_18:2 | 70744 | 51049 | 42651 | 89337 | 92205 | 56751 | 48070 | 81036 | 66480 |
| PC 36:4 | 587 | 396 | 455 | 1291 | 1185 | 634 | 405 | 1275 | 779 |
| PC 36:4|PC 18:2_18:2 | 16812 | 13221 | 16673 | 55038 | 48263 | 23648 | 15738 | 63262 | 31582 |
| PC 36:5|PC 16:0_20:5 | 2422 | 1846 | 2690 | 8670 | 7905 | 4823 | 2564 | 8191 | 4889 |
| PC 36:5|PC 18:2_18:3 | 1400 | 1174 | 967 | 3443 | 3022 | 1471 | 1504 | 2993 | 1997 |
| PC 36:6 | 101 | 61 | 93 | 331 | 352 | 148 | 135 | 369 | 199 |
| PC 37:1|PC 19:0_18:1 | 544 | 589 | 509 | 639 | 544 | 331 | 623 | 603 | 548 |
| PC 37:2|PC 18:1_19:1 | 1042 | 718 | 664 | 1136 | 1075 | 625 | 758 | 983 | 875 |
| PC 38:1|PC 20:0_18:1 | 923 | 909 | 574 | 1317 | 1273 | 919 | 546 | 1408 | 984 |
| PC 38:2 | 835 | 676 | 489 | 1152 | 767 | 540 | 666 | 939 | 758 |
| PC 38:2|PC 20:0_18:2 | 2088 | 1815 | 1770 | 3154 | 2722 | 2048 | 1667 | 3329 | 2324 |
| PC 38:3 | 1425 | 956 | 1223 | 2497 | 1858 | 1516 | 1986 | 2321 | 1723 |

| PC 38:3|PC 20:0_18:3 | 840 | 540 | 619 | 1783 | 1610 | 822 | 682 | 2917 | 1227 |
| --- | --- | --- | --- | --- | --- | --- | --- | --- | --- |
| PC 38:4 | 1101 | 1198 | 1029 | 2132 | 1951 | 904 | 1108 | 1756 | 1397 |
| PC 38:4|PC 18:1_20:3 | 2426 | 2188 | 1991 | 4526 | 4022 | 2853 | 2723 | 6598 | 3416 |
| PC 38:5|PC 16:0_22:5 | 4192 | 4148 | 4645 | 8436 | 7117 | 6126 | 5560 | 10592 | 6352 |
| PC 38:6|PC 19:3_19:3 | 1274 | 1102 | 1075 | 2923 | 2871 | 2152 | 1381 | 4811 | 2199 |
| PC 38:7 | 305 | 262 | 194 | 708 | 732 | 364 | 419 | 756 | 468 |
| PC 40:3|PC 25:1_15:2 | 298 | 305 | 223 | 633 | 549 | 425 | 291 | 855 | 447 |
| PC 40:5|PC 18:0_22:5 | 650 | 564 | 619 | 1034 | 841 | 572 | 978 | 1226 | 811 |
| PC 40:6 | 1124 | 963 | 945 | 2090 | 1650 | 1062 | 1183 | 2558 | 1447 |
| PC O-29:1 | 373 | 365 | 310 | 584 | 666 | 483 | 484 | 626 | 486 |
| PC O-31:0 | 4567 | 4678 | 2531 | 8810 | 9151 | 5109 | 3224 | 9583 | 5957 |
| PC O-31:1 | 1172 | 1076 | 942 | 2545 | 2180 | 1618 | 1100 | 3062 | 1712 |
| PC O-31:6 | 79 | 89 | 73 | 172 | 209 | 131 | 125 | 227 | 138 |
| PC O-33:2 | 1575 | 1210 | 1457 | 2560 | 2569 | 1278 | 2526 | 2044 | 1902 |
| PC O-33:3 | 943 | 789 | 654 | 1577 | 1209 | 976 | 1012 | 1287 | 1056 |
| PC O-33:6 | 348 | 325 | 357 | 1018 | 894 | 620 | 528 | 634 | 591 |
| PC O-35:6 | 731 | 605 | 767 | 2571 | 2938 | 1564 | 905 | 2364 | 1556 |
| PC O-35:9 | 1223 | 1009 | 1021 | 2935 | 2956 | 2094 | 728 | 2812 | 1847 |
| PC O-37:6 | 204 | 202 | 227 | 516 | 430 | 268 | 241 | 424 | 314 |
| PC O-38:10 | 825 | 713 | 831 | 2580 | 1718 | 840 | 755 | 1930 | 1274 |
| PC O-39:0 | 669 | 801 | 552 | 993 | 979 | 591 | 598 | 620 | 725 |
| PC O-39:10 | 1943 | 2283 | 1457 | 4172 | 3148 | 2171 | 1606 | 2497 | 2410 |
| PC O-33:4 | 4088 | 3261 | 3671 | 13066 | 11181 | 8058 | 2809 | 14758 | 7611 |
| **Phosphatidylethanolamines** |  |  |  |  |  |  |  |  |  |
| PE 28:0|PE 14:0_14:0 | 1566 | 1608 | 989 | 2571 | 2213 | 1746 | 978 | 1228 | 1613 |
| PE 30:0|PE 14:0_16:0 | 3587 | 5454 | 3619 | 5481 | 5510 | 4949 | 2762 | 2486 | 4231 |
| PE 30:1|PE 12:0_18:1 | 1890 | 2020 | 866 | 3734 | 3532 | 2066 | 866 | 3095 | 2259 |
| PE 31:0|PE 15:0_16:0 | 591 | 773 | 490 | 1061 | 916 | 899 | 390 | 428 | 693 |
| PE 32:0|PE 16:0_16:0 | 6226 | 8798 | 4900 | 11724 | 10716 | 8758 | 5619 | 6354 | 7887 |
| PE 32:1|PE 14:0_18:1 | 18583 | 26505 | 11433 | 49491 | 42696 | 31255 | 15370 | 31940 | 28409 |
| PE 32:1|PE 16:0_16:1 | 4636 | 4339 | 2845 | 12203 | 9722 | 8444 | 3173 | 8433 | 6724 |
| PE 32:2 | 301 | 330 | 293 | 1064 | 1082 | 514 | 419 | 884 | 611 |
| PE 32:2|PE 14:0_18:2 | 2786 | 3649 | 1658 | 6606 | 6214 | 3985 | 2880 | 5294 | 4134 |
| PE 32:3|PE 14:0_18:3 | 260 | 139 | 190 | 566 | 696 | 280 | 95 | 614 | 355 |
| PE 33:0|PE 16:0_17:0 | 760 | 1151 | 833 | 1080 | 1047 | 1070 | 633 | 587 | 895 |
| PE 33:1|PE 15:0_18:1 | 4829 | 6624 | 3613 | 9596 | 8471 | 6394 | 4668 | 5828 | 6253 |
| PE 34:0|PE 16:0_18:0 | 3181 | 4525 | 3672 | 5480 | 4382 | 3853 | 3172 | 3523 | 3973 |
| PE 34:1 | 3196 | 2770 | 1629 | 5799 | 5567 | 3138 | 2654 | 4839 | 3699 |
| PE 34:1|PE 16:0_18:1 | 166512 | 227283 | 122355 | 373423 | 307585 | 246878 | 153394 | 247742 | 230646 |
| PE 34:2 | 900 | 849 | 391 | 2005 | 1584 | 1013 | 959 | 1409 | 1139 |
| PE 34:2|PE 16:0_18:2 | 66097 | 88679 | 39919 | 161682 | 136288 | 90247 | 64810 | 117682 | 95676 |
| PE 34:3|PE 16:0_18:3 | 6466 | 8364 | 4565 | 24454 | 20819 | 11818 | 6842 | 21660 | 13123 |
| PE 34:3|PE 16:1_18:2 | 2239 | 2873 | 853 | 6245 | 5653 | 3786 | 1317 | 4559 | 3441 |
| PE 34:4|PE 14:0_20:4 | 556 | 616 | 439 | 1466 | 1719 | 981 | 607 | 1451 | 979 |
| PE 34:4|PE 16:1_18:3 | 257 | 257 | 90 | 1391 | 1171 | 740 | 115 | 1478 | 687 |

| PE 35:0|PE 17:0_18:0 | 536 | 399 | 457 | 465 | 326 | 417 | 204 | 230 | 379 |
| --- | --- | --- | --- | --- | --- | --- | --- | --- | --- |
| PE 35:1|PE 17:0_18:1 | 10140 | 13578 | 8464 | 15023 | 12775 | 10001 | 9822 | 9725 | 11191 |
| PE 35:2|PE 17:1_18:1 | 7918 | 10189 | 5583 | 14513 | 13349 | 8884 | 7312 | 10759 | 9813 |
| PE 35:3|PE 17:1_18:2 | 809 | 1087 | 499 | 2685 | 1903 | 1066 | 825 | 1916 | 1349 |
| PE 36:0|PE 18:0_18:0 | 426 | 478 | 482 | 843 | 873 | 566 | 363 | 993 | 628 |
| PE 36:1|PE 18:0_18:1 | 183147 | 212374 | 141235 | 320789 | 259911 | 159081 | 178888 | 260394 | 214477 |
| PE 36:2 | 1457 | 1103 | 1146 | 3991 | 4344 | 2060 | 1657 | 6961 | 2840 |
| PE 36:2|PE 18:1_18:1 | 415917 | 570586 | 352323 | 771941 | 664472 | 529177 | 389431 | 570076 | 532990 |
| PE 36:3 | 1545 | 1437 | 926 | 2726 | 2493 | 1210 | 1561 | 1858 | 1720 |
| PE 36:3;2O|PE 14:0_22:3;2O | 1015 | 969 | 375 | 2080 | 957 | 873 | 774 | 1010 | 1007 |
| PE 36:3|PE 18:1_18:2 | 102672 | 140248 | 78902 | 212058 | 205615 | 126536 | 113630 | 165771 | 143179 |
| PE 36:4|PE 16:0_20:4 | 9081 | 11324 | 7177 | 27217 | 23876 | 14519 | 10499 | 21145 | 15605 |
| PE 36:4|PE 18:1_18:3 | 19129 | 27149 | 16019 | 61562 | 57729 | 30048 | 21471 | 61749 | 36857 |
| PE 36:5|PE 16:0_20:5 | 1899 | 2056 | 1311 | 6505 | 5239 | 3435 | 1948 | 6317 | 3589 |
| PE 36:5|PE 18:2_18:3 | 1041 | 1119 | 778 | 4604 | 4052 | 2245 | 1424 | 4388 | 2456 |
| PE 37:1|PE 18:0_19:1 | 1319 | 1465 | 880 | 1940 | 1446 | 965 | 1041 | 1182 | 1280 |
| PE 37:2|PE 18:1_19:1 | 3538 | 4717 | 2705 | 5164 | 4017 | 3642 | 2537 | 3663 | 3748 |
| PE 37:3|PE 18:1_19:2 | 730 | 984 | 507 | 1385 | 1256 | 891 | 596 | 1190 | 942 |
| PE 38:1|PE 20:0_18:1 | 2776 | 2563 | 1549 | 3294 | 2298 | 1961 | 1514 | 2447 | 2300 |
| PE 38:2|PE 18:1_20:1 | 4586 | 5082 | 3073 | 5955 | 4941 | 4441 | 2999 | 3762 | 4355 |
| PE 38:3|PE 18:0_20:3 | 7330 | 9872 | 5680 | 15520 | 11927 | 8901 | 10488 | 15870 | 10699 |
| PE 38:3|PE 18:1_20:2 | 1772 | 2092 | 1640 | 2907 | 2600 | 2077 | 1744 | 3311 | 2268 |
| PE 38:4|PE 18:0_20:4 | 7438 | 10076 | 7464 | 19037 | 16608 | 10121 | 11852 | 23306 | 13238 |
| PE 38:4|PE 18:1_20:3 | 7316 | 9484 | 6053 | 16095 | 14224 | 11636 | 9391 | 16415 | 11327 |
| PE 38:5|PE 16:0_22:5 | 12938 | 17912 | 10206 | 29365 | 27505 | 18674 | 16942 | 32231 | 20722 |
| PE 38:5|PE 18:1_20:4 | 3900 | 2850 | 2602 | 9883 | 8367 | 6159 | 3723 | 15818 | 6663 |
| PE 38:6 | 726 | 803 | 489 | 2600 | 2040 | 1128 | 832 | 1949 | 1321 |
| PE 39:2|PE 18:1_21:1 | 926 | 842 | 439 | 992 | 909 | 773 | 360 | 719 | 745 |
| PE 40:1|PE 22:0_18:1 | 3150 | 2888 | 671 | 2378 | 1584 | 2226 | 1176 | 2053 | 2016 |
| PE 40:2|PE 18:1_22:1 | 895 | 713 | 225 | 1141 | 890 | 845 | 476 | 977 | 770 |
| PE 40:4|PE 18:0_22:4 | 1327 | 1388 | 970 | 2331 | 1879 | 1010 | 1391 | 5091 | 1923 |
| PE 40:5|PE 18:0_22:5 | 8422 | 11258 | 6350 | 14806 | 12824 | 7665 | 11404 | 22751 | 11935 |
| PE 40:6|PE 18:1_22:5 | 2985 | 2899 | 2451 | 9991 | 5381 | 3610 | 3113 | 7481 | 4739 |
| PE 44:10|PE 22:5_22:5 | 1992 | 2276 | 1854 | 7371 | 4048 | 2162 | 2074 | 3694 | 3184 |
| PE O-30:1|PE O-14:1_16:0 | 305 | 315 | 157 | 122 | 198 | 255 | 191 | 104 | 206 |
| PE O-31:1|PE O-15:1_16:0 | 971 | 1245 | 733 | 1519 | 943 | 1142 | 893 | 609 | 1007 |
| PE O-31:2|PE O-15:1_16:1 | 414 | 472 | 258 | 1090 | 948 | 630 | 509 | 900 | 653 |
| PE O-32:1|PE O-16:1_16:0 | 390 | 435 | 430 | 635 | 477 | 644 | 339 | 388 | 467 |
| PE O-32:2|PE O-14:1_18:1 | 2147 | 2636 | 1369 | 4619 | 3321 | 2654 | 2285 | 3975 | 2876 |
| PE O-32:3|PE O-14:1_18:2 | 806 | 1024 | 558 | 1594 | 1552 | 906 | 948 | 1122 | 1064 |
| PE O-33:2|PE O-15:1_18:1 | 4521 | 5692 | 3309 | 9653 | 7696 | 6793 | 6326 | 8336 | 6541 |
| PE O-33:3|PE O-15:1_18:2 | 2129 | 2635 | 1559 | 3815 | 3405 | 2516 | 2701 | 2639 | 2675 |
| PE O-33:4|PE O-15:1_18:3 | 345 | 464 | 240 | 949 | 714 | 509 | 389 | 860 | 559 |
| PE O-34:2|PE O-16:1_18:1 | 14104 | 17929 | 11105 | 28753 | 21544 | 18298 | 14555 | 28681 | 19371 |
| PE O-34:3|PE O-16:1_18:2 | 7569 | 9302 | 5569 | 14703 | 11222 | 10499 | 9009 | 15030 | 10363 |

| PE O-34:4|PE O-16:1_18:3 | 1582 | 1963 | 1252 | 4430 | 3483 | 2550 | 1671 | 5376 | 2788 |
| --- | --- | --- | --- | --- | --- | --- | --- | --- | --- |
| PE O-35:1 | 2642 | 3046 | 1604 | 5552 | 5842 | 3275 | 3090 | 4902 | 3744 |
| PE O-35:2|PE O-17:1_18:1 | 1956 | 2237 | 1577 | 4606 | 3373 | 2939 | 1748 | 4212 | 2831 |
| PE O-35:3|PE O-17:1_18:2 | 989 | 1380 | 858 | 1937 | 1770 | 1659 | 1386 | 1834 | 1477 |
| PE O-35:3|PE O-17:2_18:1 | 371 | 383 | 182 | 543 | 545 | 597 | 499 | 604 | 466 |
| PE O-35:5|PE O-15:1_20:4 | 638 | 561 | 326 | 978 | 770 | 749 | 505 | 691 | 652 |
| PE O-35:6|PE O-15:1_20:5 | 333 | 230 | 225 | 422 | 354 | 379 | 345 | 282 | 321 |
| PE O-36:2|PE O-18:0_18:2 | 857 | 1402 | 273 | 746 | 767 | 10146 | 284 | 1270 | 1968 |
| PE O-36:2|PE O-18:1_18:1 | 4564 | 5209 | 2934 | 8887 | 6566 | 5543 | 3489 | 7110 | 5538 |
| PE O-36:3|PE O-18:1_18:2 | 721 | 946 | 543 | 1310 | 1104 | 1526 | 513 | 1957 | 1077 |
| PE O-36:3|PE O-18:2_18:1 | 4590 | 5377 | 3150 | 9566 | 6939 | 6318 | 4153 | 8756 | 6106 |
| PE O-36:4|PE O-18:2_18:2 | 2121 | 2512 | 1331 | 3651 | 3003 | 2672 | 2094 | 4188 | 2697 |
| PE O-36:5|PE O-16:1_20:4 | 2123 | 2354 | 1122 | 3057 | 2263 | 2559 | 2108 | 3505 | 2386 |
| PE O-36:5|PE O-18:2_18:3 | 424 | 494 | 328 | 790 | 680 | 583 | 307 | 1115 | 590 |
| PE O-36:6|PE O-16:1_20:5 | 899 | 908 | 473 | 1403 | 1231 | 1100 | 667 | 1795 | 1059 |
| PE O-38:4|PE O-18:1_20:3 | 557 | 558 | 321 | 950 | 888 | 958 | 450 | 1199 | 735 |
| PE O-38:5|PE O-18:1_20:4 | 1004 | 1202 | 609 | 1415 | 1388 | 1466 | 1114 | 1676 | 1234 |
| PE O-38:6|PE O-16:1_22:5 | 2231 | 2207 | 1148 | 2442 | 1884 | 2402 | 2333 | 4085 | 2341 |
| PE O-40:6|PE O-18:1_22:5 | 664 | 689 | 460 | 968 | 587 | 827 | 568 | 1285 | 756 |
| PE O-19:0_18:1 | 1025 | 637 | 570 | 2404 | 2133 | 1061 | 1035 | 1736 | 1325 |
| PE P-30:1|PE P-14:0_16:1 | 349 | 267 | 244 | 429 | 434 | 272 | 241 | 585 | 352 |
| PE P-31:0|PE P-15:0_16:0 | 372 | 325 | 224 | 523 | 568 | 366 | 452 | 368 | 400 |
| PE P-32:1|PE P-14:0_18:1 | 999 | 797 | 683 | 1937 | 1626 | 784 | 1245 | 1816 | 1236 |
| PE P-32:2|PE P-14:0_18:2 | 310 | 281 | 264 | 627 | 753 | 367 | 307 | 595 | 438 |
| PE P-33:1|PE P-15:0_18:1 | 1004 | 653 | 551 | 1638 | 1258 | 849 | 1054 | 1253 | 1032 |
| PE P-33:2|PE P-15:0_18:2 | 605 | 467 | 409 | 1110 | 872 | 706 | 804 | 827 | 725 |
| PE P-33:3|PE P-15:0_18:3 | 261 | 211 | 237 | 859 | 681 | 427 | 261 | 1123 | 508 |
| PE P-34:1|PE P-16:0_18:1 | 4362 | 3790 | 3291 | 8903 | 6666 | 5325 | 4725 | 9879 | 5868 |
| PE P-34:2|PE P-16:0_18:2 | 1606 | 1528 | 1216 | 3633 | 3006 | 1948 | 1824 | 3006 | 2221 |
| PE P-34:3|PE P-16:0_18:3 | 499 | 403 | 394 | 1196 | 981 | 539 | 549 | 1634 | 774 |
| PE P-36:1|PE P-18:0_18:1 | 2258 | 1758 | 1455 | 3311 | 3129 | 1917 | 2610 | 3089 | 2441 |
| PE P-36:2|PE P-18:0_18:2 | 1023 | 1108 | 631 | 2477 | 1814 | 1219 | 1328 | 1860 | 1433 |
| PE P-36:2|PE P-18:1_18:1 | 1073 | 784 | 545 | 1769 | 1373 | 894 | 1038 | 2095 | 1196 |
| PE P-36:3|PE P-16:0_20:3 | 552 | 507 | 409 | 949 | 717 | 638 | 702 | 1027 | 688 |
| PE P-36:3|PE P-18:1_18:2 | 563 | 559 | 469 | 1009 | 950 | 712 | 697 | 1371 | 791 |
| PE P-36:4|PE P-16:0_20:4 | 406 | 392 | 244 | 816 | 474 | 370 | 531 | 668 | 488 |
| **Phosphatidylinositol** |  |  |  |  |  |  |  |  |  |
| PI 30:0 | 856 | 558 | 627 | 3113 | 1442 | 842 | 689 | 1069 | 1149 |
| PI 30:0|PI 14:0_16:0 | 998 | 666 | 652 | 3321 | 1524 | 863 | 687 | 1169 | 1235 |
| PI 32:0|PI 16:0_16:0 | 5599 | 4333 | 2947 | 18827 | 9724 | 5965 | 4635 | 9125 | 7644 |
| PI 32:1|PI 14:0_18:1 | 2876 | 1986 | 1508 | 11028 | 4553 | 2994 | 1777 | 3684 | 3801 |
| PI 33:1 | 775 | 501 | 361 | 2695 | 1185 | 691 | 568 | 1116 | 986 |
| PI 34:0|PI 16:0_18:0 | 5617 | 4471 | 3817 | 16019 | 9888 | 4619 | 5995 | 7744 | 7271 |
| PI 34:1|PI 16:0_18:1 | 26831 | 20900 | 11440 | 83701 | 38458 | 24187 | 20269 | 38459 | 33031 |
| PI 34:2|PI 16:1_18:1 | 5927 | 3937 | 2110 | 18138 | 8926 | 5116 | 4485 | 7594 | 7029 |

| PI 34:3 | 419 | 243 | 140 | 1812 | 783 | 385 | 266 | 931 | 622 |
| --- | --- | --- | --- | --- | --- | --- | --- | --- | --- |
| PI 35:2 | 668 | 502 | 312 | 1557 | 860 | 547 | 294 | 703 | 680 |
| PI 36:0 | 1682 | 1265 | 1683 | 3131 | 2706 | 1253 | 1194 | 1248 | 1770 |
| PI 36:1|PI 18:0_18:1 | 121462 | 85403 | 67738 | 265229 | 128851 | 79455 | 82378 | 100086 | 116325 |
| PI 36:2|PI 18:1_18:1 | 56664 | 42480 | 26762 | 129289 | 60891 | 35371 | 40278 | 50172 | 55238 |
| PI 36:3|PI 18:1_18:2 | 10303 | 7413 | 4992 | 32346 | 14537 | 7624 | 8747 | 13051 | 12377 |
| PI 36:4|PI 16:0_20:4 | 2932 | 2092 | 1164 | 6866 | 3804 | 2531 | 2269 | 2358 | 3002 |
| PI 36:4|PI 18:2_18:2 | 837 | 494 | 372 | 2990 | 1328 | 707 | 608 | 1587 | 1115 |
| PI 36:5 | 952 | 738 | 582 | 2114 | 1472 | 964 | 867 | 976 | 1083 |
| PI 37:1 | 1897 | 1215 | 907 | 3275 | 1538 | 1081 | 765 | 1574 | 1532 |
| PI 37:2 | 1682 | 1242 | 737 | 2982 | 1521 | 976 | 621 | 1232 | 1374 |
| PI 38:1 | 2645 | 1437 | 1030 | 4542 | 1952 | 1389 | 1027 | 1680 | 1963 |
| PI 38:2 | 4796 | 2936 | 2174 | 9303 | 3553 | 2711 | 2435 | 3336 | 3905 |
| PI 38:3|PI 18:0_20:3 | 14662 | 9998 | 8721 | 25596 | 13281 | 9426 | 11774 | 7828 | 12661 |
| PI 38:4|PI 18:0_20:4 | 14340 | 11721 | 11067 | 30930 | 16845 | 11328 | 15167 | 8582 | 14998 |
| PI 38:4|PI 18:1_20:3 | 5021 | 2869 | 2490 | 10039 | 4855 | 4051 | 3876 | 4466 | 4708 |
| PI 38:5|PI 18:0_20:5 | 7758 | 5904 | 4661 | 16074 | 8523 | 6273 | 5948 | 5556 | 7587 |
| PI 38:5|PI 18:1_20:4 | 6183 | 4243 | 3330 | 14737 | 7495 | 5452 | 4713 | 5193 | 6418 |
| PI 38:6 | 1418 | 838 | 649 | 4170 | 1867 | 1178 | 787 | 1401 | 1538 |
| PI 40:3 | 13726 | 18926 | 9591 | 16937 | 19766 | 11284 | 20517 | 16065 | 15851 |
| PI 40:6 | 298 | 188 | 155 | 541 | 337 | 243 | 199 | 643 | 325 |
| PI 42:10 | 1933 | 1913 | 1296 | 5279 | 2782 | 1417 | 1852 | 2183 | 2332 |
| **Phosphatidylserines** |  |  |  |  |  |  |  |  |  |
| PS 34:1|PS 16:0_18:1 | 11562 | 12303 | 5153 | 31967 | 13724 | 14001 | 9757 | 11248 | 13714 |
| PS 34:2|PS 16:0_18:2 | 4586 | 3525 | 1466 | 11988 | 5003 | 5010 | 3277 | 4464 | 4915 |
| PS 34:3|PS 16:0_18:3 | 425 | 249 | 163 | 1265 | 589 | 551 | 204 | 536 | 498 |
| PS 35:1|PS 18:0_17:1 | 4347 | 3555 | 2075 | 8188 | 4098 | 3114 | 3412 | 3528 | 4039 |
| PS 36:0|PS 18:0_18:0 | 22205 | 18238 | 12548 | 43311 | 24545 | 17035 | 21881 | 23765 | 22941 |
| PS 36:1|PS 18:0_18:1 | 157106 | 120263 | 86231 | 331960 | 159612 | 111661 | 144137 | 157217 | 158524 |
| PS 36:2|PS 18:1_18:1 | 88202 | 74458 | 43077 | 189278 | 91154 | 59701 | 76610 | 87860 | 88792 |
| PS 36:3|PS 18:0_18:3 | 2208 | 2253 | 1920 | 12432 | 6219 | 2586 | 3024 | 7601 | 4780 |
| PS 36:3|PS 18:1_18:2 | 15495 | 11481 | 7295 | 42935 | 20125 | 12122 | 13420 | 23055 | 18241 |
| PS 36:4|PS 18:1_18:3 | 1507 | 1033 | 692 | 4969 | 2679 | 1475 | 1075 | 2706 | 2017 |
| PS 38:1|PS 20:0_18:1 | 4995 | 4071 | 4056 | 10512 | 4658 | 4750 | 4478 | 3952 | 5184 |
| PS 38:2|PS 18:1_20:1 | 2985 | 2353 | 2064 | 5590 | 2955 | 2713 | 2527 | 2946 | 3017 |
| PS 38:3 | 27287 | 13828 | 12880 | 57426 | 39088 | 18910 | 11081 | 29699 | 26275 |
| PS 38:3|PS 18:0_20:3 | 12665 | 10509 | 7065 | 30344 | 15341 | 9501 | 12980 | 14886 | 14161 |
| PS 38:4|PS 18:1_20:3 | 2206 | 1943 | 1367 | 5743 | 2916 | 2018 | 2168 | 3002 | 2670 |
| PS 38:5|PS 16:0_22:5 | 1537 | 1248 | 643 | 3826 | 1357 | 1719 | 1077 | 1891 | 1662 |
| PS 39:1|PS 21:0_18:1 | 3869 | 2584 | 2743 | 7760 | 3899 | 3007 | 2895 | 3680 | 3805 |
| PS 39:2|PS 18:1_21:1 | 2096 | 1575 | 1632 | 4667 | 2851 | 2105 | 1503 | 2531 | 2370 |
| PS 40:1|PS 22:0_18:1 | 4816 | 4187 | 4218 | 9836 | 5010 | 4059 | 4288 | 4557 | 5121 |
| PS 40:2 | 1959 | 1471 | 1419 | 3646 | 1959 | 1742 | 1364 | 2118 | 1960 |
| PS 40:2|PS 22:0_18:2 | 1862 | 1352 | 1313 | 3715 | 1830 | 1628 | 1313 | 1906 | 1865 |
| PS 40:3 | 2447 | 1181 | 1742 | 5610 | 3993 | 1841 | 967 | 2756 | 2567 |
| PS 40:4|PS 18:0_22:4 | 2567 | 1720 | 1802 | 7254 | 3105 | 1726 | 2605 | 5508 | 3286 |

| PS 40:5|PS 18:0_22:5 | 21273 | 16103 | 13542 | 54444 | 27062 | 15254 | 23484 | 39306 | 26308 |
| --- | --- | --- | --- | --- | --- | --- | --- | --- | --- |
| PS 40:6|PS 18:1_22:5 | 2771 | 2122 | 1622 | 8198 | 3694 | 2225 | 3186 | 6346 | 3771 |
| PS 41:2 | 873 | 582 | 837 | 2609 | 1164 | 823 | 657 | 1568 | 1139 |
| PS 42:1|PS 24:0_18:1 | 922 | 664 | 725 | 2031 | 1078 | 1034 | 728 | 1256 | 1055 |
| **Sulfatides** |  |  |  |  |  |  |  |  |  |
| SHexCer 35:0;3O | 1359 | 1955 | 1323 | 6016 | 2304 | 1203 | 1919 | 1946 | 2253 |
| SHexCer 36:4;2O | 793 | 3007 | 1430 | 3601 | 2754 | 1018 | 2447 | 1292 | 2043 |
| SHexCer 38:2;2O | 304 | 406 | 197 | 368 | 326 | 189 | 170 | 117 | 260 |
| SHexCer 38:7;3O | 234 | 275 | 243 | 920 | 441 | 186 | 249 | 225 | 347 |
| SHexCer 39:5;3O | 626 | 1116 | 652 | 2022 | 1019 | 458 | 903 | 670 | 933 |
| SHexCer 39:6;3O | 345 | 440 | 324 | 903 | 514 | 251 | 482 | 338 | 450 |
| SHexCer 39:7;3O | 2408 | 5362 | 2911 | 10567 | 6336 | 3185 | 4090 | 4091 | 4869 |
| SHexCer 39:8;3O | 911 | 1996 | 1015 | 4369 | 2718 | 1298 | 1394 | 1565 | 1908 |
| SHexCer 40:2;2O | 2252 | 3474 | 1476 | 5328 | 3158 | 2171 | 1409 | 2372 | 2705 |
| SHexCer 40:3;3O | 610 | 1042 | 1481 | 3135 | 1714 | 826 | 1253 | 1801 | 1483 |
| SHexCer 40:8;3O | 195 | 357 | 171 | 990 | 327 | 183 | 395 | 249 | 358 |
| SHexCer 40:9;3O | 478 | 965 | 513 | 1662 | 818 | 338 | 767 | 436 | 747 |
| SHexCer 41:8;3O | 3706 | 10811 | 6248 | 17414 | 11558 | 4666 | 9604 | 5711 | 8715 |
| SHexCer 42:2;2O | 196 | 295 | 136 | 309 | 202 | 122 | 80 | 102 | 180 |
| SHexCer 42:4;3O | 1929 | 3365 | 3265 | 7900 | 4347 | 1789 | 3718 | 4414 | 3841 |
| SHexCer 43:0;3O | 615 | 655 | 870 | 2209 | 1513 | 513 | 612 | 862 | 981 |
| SHexCer 44:2;2O | 1799 | 3144 | 1513 | 3057 | 2013 | 1142 | 1746 | 786 | 1900 |
| SHexCer 46:2;2O | 6214 | 9522 | 4992 | 16028 | 8504 | 4988 | 5878 | 5706 | 7729 |
| **Sphingomyelins** |  |  |  |  |  |  |  |  |  |
| SM 30:0;2O|SM 22:0;2O/8:0 | 1146 | 860 | 576 | 912 | 1327 | 755 | 657 | 948 | 898 |
| SM 30:1;2O|SM 16:1;2O/14:0 | 1005 | 841 | 585 | 1131 | 1115 | 714 | 893 | 604 | 861 |
| SM 31:0;2O|SM 19:0;2O/12:0 | 286 | 207 | 146 | 287 | 237 | 155 | 211 | 255 | 223 |
| SM 31:1;2O|SM 17:1;2O/14:0 | 644 | 544 | 513 | 986 | 988 | 577 | 739 | 775 | 721 |
| SM 32:0;2O|SM 22:0;2O/10:0 | 30530 | 24305 | 15770 | 44583 | 44093 | 33001 | 15385 | 44368 | 31504 |
| SM 32:1;2O|SM 16:1;2O/16:0 | 48211 | 39245 | 33872 | 77449 | 87004 | 59485 | 42921 | 71000 | 57398 |
| SM 33:0;2O | 3784 | 2863 | 2063 | 5883 | 4734 | 3478 | 2223 | 6432 | 3933 |
| SM 33:1;2O|SM 17:1;2O/16:0 | 25645 | 21133 | 21660 | 51254 | 52988 | 33123 | 23676 | 50491 | 34996 |
| SM 34:0;2O | 20547 | 22099 | 10228 | 48298 | 36185 | 27948 | 10435 | 62617 | 29795 |
| SM 34:1;2O | 1305 | 1926 | 1397 | 4569 | 2950 | 1742 | 1576 | 2390 | 2232 |
| SM 34:1;2O|SM 18:1;2O/16:0 | 265994 | 231954 | 220792 | 739394 | 640872 | 444766 | 248216 | 797079 | 448633 |
| SM 34:1;3O | 344 | 259 | 268 | 1268 | 1134 | 783 | 280 | 1341 | 710 |
| SM 34:2;2O|SM 25:2;2O/9:0 | 2270 | 1925 | 1750 | 5773 | 5145 | 3267 | 2453 | 7250 | 3729 |
| SM 35:0;2O | 465 | 402 | 307 | 910 | 847 | 546 | 330 | 790 | 575 |
| SM 35:1;2O|SM 16:1;2O/19:0 | 5452 | 4057 | 4202 | 10037 | 8692 | 6565 | 3266 | 6599 | 6109 |
| SM 35:1;2O|SM 18:1;2O/17:0 | 2395 | 2411 | 2034 | 4550 | 3884 | 2806 | 2891 | 3663 | 3079 |
| SM 35:2;2O|SM 21:1;2O/14:1 | 268 | 284 | 209 | 551 | 458 | 236 | 320 | 476 | 350 |
| SM 35:2;3O | 924 | 690 | 558 | 2106 | 1843 | 935 | 1013 | 1691 | 1220 |
| SM 36:0;2O | 1569 | 1558 | 1045 | 2680 | 1931 | 1288 | 1111 | 1898 | 1635 |
| SM 36:1;2O|SM 15:0;2O/21:1 | 9090 | 7844 | 9339 | 24026 | 21075 | 14151 | 7343 | 24824 | 14711 |
| SM 36:2;2O|SM 19:1;2O/17:1 | 1847 | 1198 | 1618 | 3841 | 3680 | 2033 | 1614 | 4246 | 2510 |
| SM 37:0;2O | 1051 | 1182 | 891 | 1958 | 1585 | 854 | 1380 | 1092 | 1249 |

| SM 37:1;2O | 494 | 506 | 342 | 796 | 825 | 465 | 538 | 657 | 578 |
| --- | --- | --- | --- | --- | --- | --- | --- | --- | --- |
| SM 38:0;2O|SM 16:0;2O/22:0 | 11617 | 10352 | 5384 | 12917 | 11675 | 6774 | 8154 | 5381 | 9032 |
| SM 38:1;2O|SM 16:1;2O/22:0 | 72317 | 76833 | 83486 | 122143 | 111089 | 69962 | 77884 | 55116 | 83604 |
| SM 38:2;2O|SM 19:2;2O/19:0 | 696 | 610 | 456 | 1210 | 1113 | 551 | 655 | 748 | 755 |
| SM 39:0;2O | 27505 | 25364 | 15786 | 34326 | 22202 | 19285 | 14744 | 18106 | 22165 |
| SM 39:1;2O|SM 17:1;2O/22:0 | 1734 | 1424 | 1158 | 2930 | 2494 | 2186 | 1340 | 2296 | 1945 |
| SM 39:1;3O | 4300 | 4964 | 5314 | 10524 | 5644 | 3738 | 3905 | 4812 | 5400 |
| SM 39:2;2O|SM 19:1;2O/20:1 | 1344 | 1166 | 914 | 2127 | 1919 | 986 | 1385 | 1310 | 1394 |
| SM 40:0;2O | 12245 | 10831 | 5135 | 14715 | 11070 | 7474 | 6997 | 9394 | 9733 |
| SM 40:1;2O|SM 16:1;2O/24:0 | 134701 | 136234 | 137940 | 243116 | 211372 | 134369 | 130618 | 149374 | 159715 |
| SM 40:1;2O|SM 18:1;2O/22:0 | 2140 | 2275 | 1440 | 3012 | 2769 | 1595 | 2136 | 1690 | 2132 |
| SM 40:1;3O | 611 | 684 | 542 | 1459 | 1761 | 931 | 451 | 935 | 922 |
| SM 40:2;2O|SM 18:1;2O/22:1 | 10234 | 10530 | 10727 | 16772 | 13963 | 12435 | 11388 | 8481 | 11816 |
| SM 40:8;2O | 324 | 328 | 170 | 573 | 508 | 190 | 697 | 316 | 388 |
| SM 41:0;2O | 3374 | 3131 | 2643 | 5422 | 3832 | 2302 | 3464 | 3453 | 3453 |
| SM 41:1;2O|SM 18:1;2O/23:0 | 33756 | 28815 | 16411 | 55502 | 33291 | 34183 | 39657 | 22116 | 32966 |
| SM 41:2;2O|SM 18:1;2O/23:1 | 3459 | 3980 | 3513 | 9157 | 7126 | 4669 | 5438 | 9474 | 5852 |
| SM 42:1;2O|SM 18:1;2O/24:0 | 28394 | 45279 | 39298 | 75863 | 62282 | 35186 | 45860 | 38130 | 46286 |
| SM 42:2;2O|SM 18:1;2O/24:1 | 7206 | 7278 | 6864 | 16559 | 10875 | 11914 | 7188 | 14395 | 10285 |
| SM 32:0;2O | 916 | 654 | 397 | 1234 | 1174 | 802 | 647 | 1048 | 859 |
| SM 33:1;2O | 461 | 478 | 432 | 1087 | 826 | 645 | 541 | 973 | 681 |
| SM 34:1;2O | 11758 | 8579 | 10478 | 41522 | 34710 | 25881 | 6704 | 45688 | 23165 |
| SM 40:1;2O | 2461 | 2518 | 3046 | 4111 | 3752 | 2677 | 2277 | 3037 | 2985 |
| SM 42:2;2O | 291 | 251 | 220 | 489 | 293 | 329 | 188 | 420 | 310 |
| Triacylglycerols |  |  |  |  |  |  |  |  |  |
| TG 46:0|TG 14:0_16:0_16:0 | 14418 | 14884 | 6051 | 52166 | 18154 | 7622 | 11025 | 7204 | 16441 |
| TG 48:1|TG 16:0_16:0_16:1 | 54097 | 52143 | 25491 | 67085 | 55601 | 45951 | 39476 | 38902 | 47343 |
| TG 49:3|TG 15:0_16:0_18:3 | 18333 | 12051 | 12710 | 98402 | 56566 | 18971 | 11712 | 33021 | 32721 |
| TG 50:2|TG 16:0_16:0_18:2 | 58517 | 26896 | 14483 | 68406 | 50149 | 34910 | 24127 | 13655 | 36393 |
| TG 51:2|TG 15:0_18:1_18:1 | 11365 | 4914 | 3623 | 7764 | 5541 | 7088 | 3562 | 3876 | 5967 |
| TG 51:4|TG 15:0_16:0_20:4 | 10052 | 8592 | 7686 | 53056 | 26028 | 11668 | 6632 | 16805 | 17565 |
| TG 52:2|TG 16:0_18:1_18:1 | 37904 | 21087 | 17107 | 25613 | 18939 | 10221 | 16564 | 9349 | 19598 |
| TG 52:3|TG 16:0_18:1_18:2 | 1088526 | 611449 | 408643 | 2216304 | 1374044 | 893480 | 470358 | 746114 | 976115 |
| TG 53:4|TG 17:1_18:1_18:2 | 13963 | 7630 | 3479 | 20857 | 19358 | 8810 | 6264 | 10723 | 11385 |
| TG 54:6|TG 18:1_18:2_18:3 | 15365 | 11755 | 10827 | 65915 | 35704 | 15896 | 10662 | 23578 | 23713 |
| TG 56:6|TG 16:0_18:1_22:5 | 21160 | 7686 | 5388 | 34958 | 27544 | 18535 | 13551 | 20227 | 18631 |
| TG 57:6|TG 17:0_18:1_22:5 | 935 | 332 | 312 | 908 | 472 | 573 | 364 | 663 | 570 |
| TG 10:0_10:0_20:5 | 21805 | 27475 | 12011 | 80857 | 63288 | 24090 | 47866 | 54440 | 41479 |
| TG 8:0_8:0_16:4 | 431 | 514 | 272 | 1490 | 1451 | 306 | 1037 | 1018 | 815 |
| TG O-42:5|TG O-20:5_11:0_11:0 | 1376 | 691 | 328 | 1062 | 582 | 440 | 369 | 284 | 641 |
| TG O-49:6|TG O-15:4_17:1_17:1 | 3721 | 3064 | 1337 | 9826 | 3180 | 1578 | 2160 | 1658 | 3316 |
| TG O-51:6|TG O-15:4_17:0_19:2 | 1782 | 1471 | 849 | 6228 | 1225 | 852 | 1117 | 558 | 1760 |
| TG O-51:7|TG O-15:4_15:0_21:3 | 7095 | 5460 | 3283 | 8464 | 7586 | 5864 | 4990 | 5308 | 6006 |
| TG O-52:7|TG O-18:4_16:0_18:3 | 1909 | 944 | 629 | 1587 | 1107 | 912 | 603 | 642 | 1042 |
| TG O-54:8|TG O-15:4_18:1_21:3 | 2009 | 777 | 529 | 1006 | 730 | 809 | 550 | 451 | 857 |

| TG O-55:9|TG O-13:1_21:4_21:4 | 3592 | 1609 | 946 | 3243 | 3079 | 1593 | 1209 | 1024 | 2037 |
| --- | --- | --- | --- | --- | --- | --- | --- | --- | --- |
| TG O-57:10|TG O-15:0_21:5_21:5 | 2357 | 827 | 602 | 1201 | 1043 | 829 | 689 | 545 | 1012 |
| TG O-57:9|TG O-15:1_21:4_21:4 | 2391 | 717 | 735 | 981 | 974 | 714 | 810 | 473 | 974 |
| **Others** |  |  |  |  |  |  |  |  |  |
| CoQ8 | 766 | 695 | 394 | 1267 | 976 | 629 | 292 | 1216 | 779 |
| DGCC 17:2_22:6 | 1470 | 1360 | 1796 | 2691 | 1968 | 1331 | 1560 | 2770 | 1868 |
| DGGA 36:2|DGGA 18:1_18:1 | 660 | 1552 | 1268 | 3561 | 2411 | 1079 | 1938 | 1546 | 1752 |
| DGTS 21:0 | 799 | 468 | 1174 | 622 | 611 | 728 | 949 | 1143 | 812 |
| LPA 16:3 | 1606 | 2284 | 1419 | 1879 | 1902 | 1518 | 2475 | 1610 | 1837 |
| LPE O-19:4 | 476 | 408 | 200 | 670 | 498 | 181 | 802 | 443 | 460 |
| LPE-N (FA)33:2|LPE-N (FA 15:0)18:2 | 785 | 1010 | 467 | 1730 | 1631 | 956 | 879 | 1503 | 1120 |
| LPG O-13:0 | 875 | 1151 | 915 | 1297 | 1342 | 866 | 1201 | 1022 | 1084 |
| NAGly 9:0;O | 863 | 2085 | 811 | 576 | 1027 | 493 | 3063 | 351 | 1159 |
| Pentaerythritol tetrakis(3,5-di-tert-butyl-4-hydro | 20972 | 52811 | 20807 | 37635 | 43686 | 23059 | 59594 | 36125 | 36836 |
| PI O-16:0 | 1437 | 920 | 665 | 3762 | 1787 | 846 | 1742 | 2082 | 1655 |
| PI-Cer 38:3;2O | 487 | 406 | 428 | 966 | 842 | 506 | 579 | 1274 | 686 |
| SL 12:0;O/26:0 | 3805 | 2963 | 2111 | 5517 | 7038 | 4146 | 3729 | 5736 | 4381 |
| SL 12:1;O/32:0 | 3722 | 2721 | 1604 | 6201 | 6633 | 3072 | 5022 | 3616 | 4074 |
| ST 27:1;O;S - Cholesterol sulfate | 1043 | 1468 | 1192 | 2393 | 1790 | 944 | 1186 | 1032 | 1381 |
| **EO Treated T0** |  |  |  |  |  |  |  |  |  |
| **Metabolyte Name** | **t0_T1** | **t0_T2** | **t0_T3** | **t0_T4** | **t0_T5** | **t0_T6** | **t0_T7** | **t0_T8** | **average value** |
| ASG 27:1;O;Hex;FA 2:0 | 184,56 | 187,88 | 232,66 | 428,71 | 83,6 | 402,15 | 198,73 | 177,31 | 236,95 |
| ASG 28:1;O;Hex;FA 14:0 | 67,17 | 2,98 | 823,05 | 2543,7 | 3602,49 | 1163,74 | 1192,62 | 1289,82 | 1335,69625 |
| ASG 28:1;O;Hex;FA 16:0 | 2,5 | 30,18 | 679,24 | 1978,68 | 3434,98 | 885,3 | 1001,01 | 1198,48 | 1151,29625 |
| ASG 28:1;O;Hex;FA 18:0 | 10,93 | 29,2 | 480,34 | 1203,27 | 2952,96 | 532,49 | 600,33 | 786,44 | 824,495 |
| ASG 28:1;O;Hex;FA 20:1 | 28,94 | 26,93 | 601,1 | 1161,37 | 3296,06 | 1175,76 | 493,91 | 1026,15 | 976,2775 |
| ASG 28:2;O;Hex;FA 14:0 | 118,26 | 16,68 | 1334,83 | 3800,01 | 8320,71 | 3258,95 | 2277,77 | 3487,51 | 2826,84 |
| ASG 28:2;O;Hex;FA 16:0 | 37,21 | 29,32 | 770,95 | 2011,34 | 4558,59 | 1130,81 | 1186,39 | 1494,56 | 1402,39625 |
| ASG 28:2;O;Hex;FA 16:1 | 81,72 | 2,04 | 456,35 | 1174,44 | 2294,09 | 1069,33 | 715,55 | 1181,17 | 871,83625 |
| ASG 28:2;O;Hex;FA 20:1 | 2,63 | 2,63 | 362,19 | 654,66 | 1956,43 | 625,81 | 409,93 | 859,2 | 609,185 |
| ASG 29:2;O;Hex;FA 20:5 | 669,57 | 663,45 | 853,42 | 950,35 | 1835,93 | 1363,25 | 835,52 | 1407,65 | 1072,3925 |
| **Carnitines** |  |  |  |  |  |  |  |  |  |
| CAR 13:0 | 281,73 | 340,3 | 342,87 | 4577,22 | 850,04 | 2199,2 | 712,65 | 768,58 | 1259,07375 |
| CAR 21:2 | 1499 | 811 | 1564 | 7044 | 13134,1 | 5768,03 | 3076,47 | 3596,22 | 4561,565 |
| CAR 21:4 | 2930 | 744 | 656 | 616 | 2340,89 | 1259,84 | 652,27 | 615,81 | 1226,74625 |
| CAR 4:0 | 11856,43 | 2882,97 | 3829,75 | 30293,27 | 21173,66 | 4982,33 | 3325,27 | 6731,69 | 10634,4213 |
| CAR 5:0 | 6267,28 | 1574,22 | 3956,79 | 42943,43 | 30904,3 | 5415,9 | 3659,67 | 7013,85 | 12716,93 |
| **Ceramides** |  |  |  |  |  |  |  |  |  |
| Cer 12:0;2O/25:0 | 3010 | 1955 | 2789 | 18053 | 16944,31 | 8968,96 | 6257,09 | 6206,69 | 8023 |
| Cer 12:2;2O/19:4 | 329 | 213 | 689 | 1407 | 2592,32 | 1450,95 | 1400,62 | 928,82 | 1126 |
| Cer 13:2;2O/30:3 | 1079 | 2265 | 3248 | 4938 | 5328,4 | 4421,51 | 2766,87 | 4652,9 | 3588 |
| Cer 20:2;4O|Cer 12:2;3O/8:0;(2OH) | 4717,82 | 2157,67 | 1741,6 | 8853,83 | 8714,07 | 2929,88 | 1644,05 | 4328,69 | 4386 |
| Cer 24:3;3O|Cer 15:3;2O/9:0;O | 1145,51 | 907,41 | 1456,19 | 1272,17 | 3516,6 | 1610,69 | 808,93 | 1818,8 | 1567 |
| Cer 32:0;2O|Cer 16:0;2O/16:0 | 257,42 | 234,06 | 353,38 | 722,14 | 1097,02 | 602,09 | 564,64 | 618,31 | 556 |

| Cer 32:1;2O|Cer 16:1;2O/16:0 | 235,89 | 180,18 | 548,78 | 412,79 | 1090,86 | 756,03 | 510,16 | 660,76 | 549 |
| --- | --- | --- | --- | --- | --- | --- | --- | --- | --- |
| Cer 33:1;2O|Cer 17:1;2O/16:0 | 89,81 | 106,33 | 240,32 | 357,8 | 761,24 | 349,24 | 297,36 | 289,96 | 312 |
| Cer 33:1;4O|Cer 18:0;3O/15:1;(2OH) | 1415,25 | 855,3 | 1232,3 | 8743,58 | 4321,85 | 2849,28 | 1312,75 | 1510,3 | 2780 |
| Cer 34:1;2O|Cer 18:1;2O/16:0 | 1021,43 | 980,29 | 2342,8 | 6415,88 | 8028,89 | 3183,7 | 1649,46 | 2505,53 | 3266 |
| Cer 34:1;3O|Cer 19:0;2O/15:1;O | 807,6 | 1307,72 | 2562,46 | 1519,72 | 4178,39 | 1740,84 | 1290,73 | 2103,16 | 1939 |
| Cer 34:3;2O|Cer 12:2;2O/22:1 | 966,72 | 414,47 | 709,88 | 8722,87 | 4830,46 | 2215,51 | 2219,59 | 1645,16 | 2716 |
| Cer 36:0;3O|Cer 18:0;2O/18:0;O | 9398,31 | 7146,36 | 9915,88 | 14799,12 | 19391,41 | 12987,8 | 8130,71 | 14092,98 | 11983 |
| Cer 36:1;3O|Cer 19:0;2O/17:1;O | 244,22 | 283,7 | 498,11 | 750,42 | 856,64 | 448,25 | 310,21 | 432,26 | 478 |
| Cer 36:3;4O|Cer 19:2;3O/17:1;(2OH) | 736,84 | 832,89 | 1196,31 | 2449,37 | 1869,12 | 1225,81 | 864,34 | 1060,64 | 1279 |
| Cer 37:0;2O|Cer 19:0;2O/18:0 | 764,53 | 582,04 | 307,94 | 871,22 | 1337,17 | 1014,31 | 432,8 | 642,77 | 744 |
| Cer 38:0;4O | 1069,03 | 433,4 | 289,04 | 30208,86 | 2947,53 | 2107,11 | 484,04 | 982,86 | 4815 |
| Cer 39:0;3O|Cer 18:0;2O/21:0;O | 5732,31 | 2849,88 | 3371,36 | 39827,45 | 15524,52 | 10577,8 | 3327,49 | 6270,52 | 10935 |
| Cer 40:0;2O|Cer 16:0;2O/24:0 | 808,56 | 543,43 | 631,22 | 5787,27 | 1943,91 | 1180,66 | 689,1 | 1129,42 | 1589 |
| Cer 40:1;2O|Cer 16:1;2O/24:0 | 2184,67 | 2150,83 | 2427,31 | 11386,48 | 7521,32 | 3257,1 | 2717,8 | 3184,4 | 4354 |
| Cer 41:1;2O|Cer 18:1;2O/23:0 | 856,38 | 533,51 | 1009,4 | 4935,23 | 3609,06 | 1021,18 | 750,97 | 1623,34 | 1792 |
| Cer 42:1;2O|Cer 18:1;2O/24:0 | 2058,27 | 1519,84 | 2807,78 | 14689,29 | 6226,22 | 3639,71 | 2505,74 | 3643,58 | 4636 |
| Cer 44:2;4O|Cer 28:1;3O(FA 16:0) | 445,42 | 395,82 | 1301,41 | 3376,42 | 4623,45 | 1701,49 | 957,48 | 1904,54 | 1838 |
| **Diacylglycerols** |  |  |  |  |  |  |  |  |  |
| DG 16:0 | 805,31 | 266,14 | 790,33 | 9053,42 | 6323,97 | 2692,1 | 1573,62 | 2396,37 | 2988 |
| DG 18:0 | 1951,27 | 1120,74 | 2752,26 | 8116,76 | 16933,51 | 6731,69 | 4139,45 | 5973,78 | 5965 |
| DG 20:0 | 4709,44 | 3561,91 | 17070,04 | 64171,47 | 91300,71 | 26716,4 | 16495,7 | 28457,08 | 31560 |
| DG 21:0 | 329,1 | 166,46 | 355,82 | 5478,35 | 1944,22 | 848,1 | 575,68 | 604,17 | 1288 |
| DG 22:0 | 2313,87 | 1916,65 | 7118,42 | 27296,18 | 48675,02 | 13897,6 | 13379,9 | 11186,6 | 15723 |
| DG 22:0|DG 10:0_12:0 | 198,45 | 183,69 | 548,69 | 2225,9 | 2240,9 | 951,27 | 2168,93 | 909,85 | 1178 |
| DG 22:0|DG 8:0_14:0 | 311,65 | 174,04 | 519,96 | 2776,75 | 2116,9 | 1077,26 | 2009,09 | 916 | 1238 |
| DG 22:1 | 2044,97 | 1353,45 | 4060,25 | 11936,97 | 28549,14 | 7453,04 | 5779,55 | 8479,69 | 8707 |
| DG 22:1|DG 8:0_14:1 | 1750,11 | 1363,19 | 4083,99 | 16441,61 | 25956,55 | 5323,77 | 6107,74 | 8811,98 | 8730 |
| DG 24:0 | 1951,07 | 1633,52 | 4799,23 | 15559,56 | 21352,46 | 10557,2 | 33518,4 | 6303,46 | 11959 |
| DG 24:0|DG 10:0_14:0 | 814,26 | 620,53 | 2079,56 | 6686,86 | 6726,24 | 3591,63 | 15333,4 | 2836,61 | 4836 |
| DG 24:0|DG 8:0_16:0 | 828,74 | 528,89 | 2038,81 | 6046,61 | 11375,99 | 3806,78 | 10706 | 3071,2 | 4800 |
| DG 24:1 | 1694,03 | 1189,47 | 5900,6 | 20720,63 | 34951,79 | 7709,31 | 11655,6 | 9511,45 | 11667 |
| DG 24:1|DG 10:0_14:1 | 1175,43 | 989,43 | 3416,42 | 14229,57 | 19969,87 | 4486,88 | 6639,92 | 5666,76 | 7072 |
| DG 24:2 | 372,28 | 211,46 | 383,96 | 3900,16 | 2495,03 | 886,52 | 742,88 | 543,51 | 1192 |
| DG 24:3 | 185,18 | 114,75 | 207,58 | 3174 | 1794,62 | 464,44 | 461,94 | 374,23 | 847 |
| DG 25:0|DG 10:0_15:0 | 187,54 | 121,21 | 502,24 | 1539,33 | 2436,46 | 1147,38 | 1630,81 | 1077 | 1080 |
| DG 26:0|DG 12:0_14:0 | 2242,8 | 1720,33 | 5228,36 | 17529,07 | 31873,58 | 11921,7 | 24110 | 6242,82 | 12609 |
| DG 26:1 | 1542,48 | 1155,22 | 4183,29 | 14649,4 | 24483,33 | 8455,88 | 15262,1 | 6718,5 | 9556 |
| DG 26:1|DG 8:0_18:1 | 1164,68 | 821,92 | 3481,21 | 11404,42 | 18989,27 | 5854,38 | 12566,9 | 5960,7 | 7530 |
| DG 26:2 | 311,13 | 199,65 | 365,14 | 4743,58 | 2624,76 | 983,45 | 1147,33 | 577,32 | 1369 |
| DG 26:2|DG 8:0_18:2 | 227,29 | 173,56 | 274,18 | 4033,08 | 2174,61 | 779,68 | 1086,88 | 569,47 | 1165 |
| DG 26:3 | 128,47 | 127,28 | 180,33 | 2906,84 | 1276,38 | 398,15 | 763,67 | 281,91 | 758 |
| DG 27:0 | 482,44 | 311,99 | 615,24 | 3761,98 | 3480,66 | 1896,59 | 1916,22 | 954,9 | 1678 |
| DG 27:5 | 401,6 | 263,56 | 574,54 | 4290,34 | 2013,87 | 1702,15 | 1464,72 | 1406,51 | 1515 |
| DG 28:0|DG 12:0_16:0 | 8215,61 | 6173,9 | 14258,26 | 43853,51 | 71707,34 | 37148,8 | 62839,6 | 16990,97 | 32648 |
| DG 28:1 | 3901 | 2500,18 | 5169,57 | 41009,5 | 41709,3 | 16432 | 20092,6 | 7918,45 | 17342 |
| DG 28:1|DG 10:0_18:1 | 3437,4 | 2480,42 | 9140,53 | 24538,8 | 54758,83 | 18017,2 | 43293,7 | 12728,46 | 21049 |

| DG 28:2 | 1542,72 | 1025,81 | 2318,69 | 18095,75 | 15648,65 | 5613,1 | 4561,58 | 3445,21 | 6531 |
| --- | --- | --- | --- | --- | --- | --- | --- | --- | --- |
| DG 28:2|DG 14:1_14:1 | 1277,67 | 999,9 | 2587,45 | 16869,13 | 17060,37 | 5205,22 | 9141,45 | 4270,1 | 7176 |
| DG 28:3 | 246,68 | 207,15 | 345,08 | 3929,35 | 2385,72 | 910,11 | 1755,46 | 590,21 | 1296 |
| DG 28:3|DG 10:0_18:3 | 228,44 | 136,69 | 362,57 | 2766,7 | 2230,64 | 740,58 | 1661,26 | 465,76 | 1074 |
| DG 30:0 | 402,63 | 270,74 | 378,5 | 2849,23 | 3209,45 | 1439,42 | 1704,13 | 660,79 | 1364 |
| DG 30:0|DG 14:0_16:0 | 37664,4 | 25068,7 | 47372,12 | 188144,05 | 249208,7 | 118573 | 133199 | 52260,53 | 106436 |
| DG 30:1|DG 16:0_14:1 | 10421,83 | 7564,64 | 19025,31 | 70236,59 | 219789,95 | 63935,4 | 75423,5 | 22407,07 | 61101 |
| DG 30:2 | 764,19 | 601,43 | 891,19 | 7506,91 | 10095,19 | 3871,07 | 3357,3 | 1437,7 | 3566 |
| DG 30:2|DG 12:0_18:2 | 975,79 | 561,72 | 1829,97 | 6356,03 | 14410,76 | 4653,74 | 8398,52 | 2465,76 | 4957 |
| DG 30:2|DG 14:1_16:1 | 822,31 | 596,42 | 1709,52 | 8789,48 | 14237,44 | 4327,72 | 6973,4 | 2242,53 | 4962 |
| DG 30:3|DG 12:0_18:3 | 330,07 | 257,64 | 600,1 | 2989,39 | 3676,48 | 1370,54 | 2905,09 | 872,42 | 1625 |
| DG 30:5 | 309,69 | 191,2 | 134,02 | 6274,63 | 890,3 | 584,35 | 138,72 | 262,22 | 1098 |
| DG 30:6 | 2283,94 | 1269,47 | 2398,78 | 16273,4 | 15766,3 | 8704,01 | 5687,26 | 5491,35 | 7234 |
| DG 30:7 | 362,13 | 201,56 | 384,01 | 5119,03 | 2123,83 | 1153,63 | 513,96 | 676,23 | 1317 |
| DG 31:7 | 21278,57 | 11810,6 | 15270,98 | 104278,59 | 104046,59 | 63859,1 | 20906,3 | 32749,45 | 46775 |
| DG 31:8 | 1110,03 | 742,39 | 734,98 | 7568,39 | 8847,08 | 3289,08 | 1954,1 | 1937,91 | 3273 |
| DG 32:1|DG 14:0_18:1 | 36993,66 | 24807,9 | 43834,88 | 214097,85 | 369845,55 | 142992 | 177311 | 72024,92 | 135238 |
| DG 32:2 | 3827,61 | 2583,46 | 3336,94 | 29289,17 | 30445,67 | 11310,1 | 7678,88 | 4665,19 | 11642 |
| DG 32:2|DG 14:1_18:1 | 4952,46 | 3663,29 | 10136,68 | 25366,83 | 87325,75 | 24975,6 | 37292,9 | 13371,67 | 25886 |
| DG 32:3 | 1046,63 | 826,5 | 452,81 | 11044,2 | 6357,98 | 2428,26 | 1223,99 | 889,42 | 3034 |
| DG 32:3|DG 14:0_18:3 | 1379,71 | 1031,66 | 1517,97 | 9204,45 | 15344,94 | 5219,54 | 7068,04 | 2230,49 | 5375 |
| DG 32:6 | 4124,47 | 2265,17 | 2966,35 | 22040,48 | 17938,49 | 10594,7 | 5961,75 | 6509,54 | 9050 |
| DG 32:7 | 748,68 | 527,31 | 590,17 | 6893,94 | 6087,37 | 3325,01 | 1309,62 | 1647,7 | 2641 |
| DG 33:2 | 715,59 | 347,58 | 451,18 | 3357,43 | 3301,88 | 2690,9 | 786,03 | 824,85 | 1559 |
| DG 33:3 | 416,07 | 216,89 | 292,09 | 881,89 | 1955,28 | 1425,3 | 406,31 | 654,33 | 781 |
| DG 33:6 | 70275,86 | 34570,4 | 21181,69 | 649239,95 | 149061,75 | 107305 | 32128,6 | 56259,96 | 140003 |
| DG 33:8 | 2910,9 | 1775,42 | 2398,97 | 18656,94 | 24249,37 | 12526,5 | 6111,56 | 7030,09 | 9457 |
| DG 33:9 | 863,15 | 458,33 | 507,85 | 7993,46 | 5046,82 | 1902,4 | 1902,15 | 1277,65 | 2494 |
| DG 34:0 | 410,74 | 304,06 | 570,79 | 4953,34 | 3161,61 | 1175,92 | 895,9 | 1205,19 | 1585 |
| DG 34:0|DG 16:0_18:0 | 9430,68 | 6915,27 | 5075,87 | 117344,54 | 23814,42 | 21161,4 | 12262 | 9749,32 | 25719 |
| DG 34:1|DG 16:0_18:1 | 98706,8 | 74085,7 | 62222,15 | 567117,4 | 540515,9 | 316489 | 204908 | 153420,55 | 252183 |
| DG 34:2 | 462,65 | 284,89 | 228,52 | 4031,18 | 813,44 | 482,24 | 283,78 | 383,36 | 871 |
| DG 34:2|DG 16:0_18:2 | 19672,52 | 14080,1 | 32121,17 | 116276,55 | 330570,75 | 90893,5 | 105129 | 54988,61 | 95466 |
| DG 34:3|DG 16:0_18:3 | 4073,38 | 2745,16 | 4472,57 | 29741,24 | 50083,87 | 12475,9 | 15703 | 7761,84 | 15882 |
| DG 34:6 | 6045,88 | 3313,18 | 3292,65 | 56935,79 | 18017,46 | 12746,2 | 4269,93 | 8194,11 | 14102 |
| DG 34:7 | 2033,53 | 1034,05 | 1539,08 | 15823,07 | 10069,76 | 6436,41 | 2522,02 | 3744,6 | 5400 |
| DG 35:2 | 539,36 | 281,62 | 439,16 | 2564,01 | 2317,25 | 2127,49 | 792,73 | 889,41 | 1244 |
| DG 35:3 | 445,72 | 147,74 | 234,16 | 1163,29 | 1822,46 | 937,3 | 384,68 | 391,53 | 691 |
| DG 35:8 | 14201,28 | 7755,1 | 6659,39 | 63392,86 | 62115,48 | 40895,1 | 13899,1 | 19472,78 | 28549 |
| DG 35:9 | 2757,08 | 1545,07 | 1354,69 | 14768,26 | 16500,27 | 7941,68 | 6776,12 | 3876,17 | 6940 |
| DG 36:1|DG 18:0_18:1 | 8430,46 | 6852,96 | 4685,62 | 43783,44 | 36774,27 | 19845,3 | 24935,2 | 15339,74 | 20081 |
| DG 36:2|DG 18:1_18:1 | 29174,86 | 23522,4 | 26888,36 | 127320,1 | 207556,2 | 97561,9 | 75980,5 | 81024,88 | 83629 |
| DG 36:3|DG 18:1_18:2 | 8536,6 | 5636,04 | 10165,17 | 43306,98 | 75417,96 | 28123,2 | 31457 | 23382,59 | 28253 |
| DG 36:4|DG 18:1_18:3 | 2486,26 | 1819,87 | 2315,93 | 15963,91 | 25965,22 | 7979,25 | 9321,11 | 4293,43 | 8768 |
| DG 36:5|DG 16:0_20:5 | 446,42 | 256,13 | 417,2 | 2709,43 | 4236,3 | 1506,37 | 1183,57 | 766,37 | 1440 |
| DG 36:5|DG 18:2_18:3 | 433,91 | 264,52 | 619,62 | 2590,41 | 3780,97 | 1590,48 | 2212,75 | 840,22 | 1542 |

| DG 36:7 | 3529,38 | 1959,5 | 2091,8 | 39770,57 | 13962,01 | 8844,55 | 2394,12 | 4111,06 | 9583 |
| --- | --- | --- | --- | --- | --- | --- | --- | --- | --- |
| DG 36:8 | 484,22 | 155,88 | 181,92 | 2160,09 | 1644,76 | 982,8 | 217,41 | 329,98 | 770 |
| DG 37:9 | 5962,79 | 3020,14 | 2766,14 | 28501,64 | 29401,24 | 13625,9 | 9586,15 | 7884,51 | 12594 |
| DG 38:5|DG 16:0_22:5 | 2023,92 | 1449,3 | 4258,27 | 18543,55 | 39675,75 | 13296,2 | 13940,9 | 7727,91 | 12614 |
| DG 38:6 | 300,29 | 164,86 | 133,01 | 2483,98 | 1941,19 | 927,92 | 234,28 | 431,73 | 827 |
| DG 40:6|DG 18:1_22:5 | 872,68 | 453,04 | 820,91 | 10551,36 | 6725,24 | 2832,38 | 1742,14 | 2278,01 | 3284 |
| DG 40:8 | 625,95 | 250,25 | 252,05 | 2117,3 | 2197,19 | 1440,07 | 403,61 | 494,08 | 973 |
| DG 41:10 | 1899,06 | 862,74 | 433,86 | 28144,64 | 6244,33 | 2850,82 | 592,2 | 882,41 | 5239 |
| DG 41:11 | 559,99 | 307,89 | 229,78 | 4883,11 | 1989,55 | 1335,17 | 286,81 | 526,71 | 1265 |
| DG 41:5 | 3820,09 | 1579,09 | 1157,88 | 20369,14 | 11243,44 | 4910,2 | 1277,08 | 2876,41 | 5904 |
| DG 41:6 | 32653,44 | 23000 | 8308,65 | 1495297,85 | 37915,33 | 31366,9 | 9927,23 | 19998,26 | 207308 |
| DG 43:11 | 1448,82 | 851,11 | 330,74 | 16310,8 | 2821,77 | 2523,19 | 557,54 | 870,09 | 3214 |
| DG 43:3 | 677,84 | 459,44 | 321,75 | 15295,98 | 2548,56 | 1498,84 | 410,23 | 681,19 | 2737 |
| DG 43:4 | 3184,3 | 1878,25 | 1171,57 | 90700,84 | 7172,56 | 4853,35 | 1882,06 | 2841,27 | 14211 |
| DG 43:6 | 27655,38 | 14759 | 8107,02 | 982347,82 | 28060,66 | 31665,6 | 5683,66 | 16087,98 | 139296 |
| DG 43:7 | 33296,04 | 20730,1 | 8480,47 | 1596758,84 | 37064,48 | 29916,7 | 16353,2 | 29143,06 | 221468 |
| DG 44:7 | 723,98 | 598,93 | 908,33 | 3232,09 | 2383,7 | 1586,84 | 1596,22 | 1370,16 | 1550 |
| DG 44:9 | 687,39 | 1018,3 | 1993,38 | 2833,03 | 1964,47 | 1532,66 | 1587,04 | 2671,41 | 1786 |
| DG 46:6 | 3585,27 | 5778,91 | 5420,56 | 5940,22 | 3891,04 | 4075,53 | 4111,69 | 3876,22 | 4585 |
| DG 47:7 | 20923,5 | 11998,3 | 7337,81 | 1156341,04 | 39759,63 | 20839,7 | 15940,1 | 11566,42 | 160588 |
| DG 47:8 | 14047,9 | 7502,67 | 4079,88 | 551789,84 | 21224,86 | 12544,4 | 7688,4 | 7301,59 | 78272 |
| DG 51:7 | 2757,94 | 1906,86 | 2233,16 | 49270,09 | 13465,39 | 5493,32 | 2392,09 | 4094,99 | 10202 |
| DG 51:8 | 1263,17 | 831,89 | 1286,47 | 16462,41 | 7380,98 | 2891,98 | 1561,14 | 1874,61 | 4194 |
| **Free Fatty Acids** |  |  |  |  |  |  |  |  |  |
| FA 12:0 | 658,94 | 285,37 | 645,04 | 833,24 | 1437,84 | 1112,34 | 633,57 | 462,47 | 759 |
| FA 14:0 | 2947,45 | 3002,37 | 9248,32 | 7767,44 | 5866,77 | 4662,07 | 4720,47 | 3432,54 | 5206 |
| FA 14:1 | 263,91 | 167,12 | 2385,98 | 2015,93 | 1203,27 | 763,16 | 428,08 | 409,9 | 955 |
| FA 15:0 | 469,05 | 288,07 | 800,87 | 660,31 | 1212,33 | 993,84 | 481,87 | 425,59 | 666 |
| FA 15:4 | 3675,11 | 1761,34 | 1340,88 | 1211,69 | 6537,46 | 4391,51 | 1389,37 | 2218,72 | 2816 |
| FA 16:0 | 271059,9 | 244445 | 311202,4 | 318652,6 | 423431,95 | 468674 | 242860 | 267747,65 | 318509 |
| FA 16:0;(2OH) | 445,2 | 301,55 | 379,97 | 338,28 | 455,59 | 598,91 | 244,96 | 541,13 | 413 |
| FA 16:0;O | 1385,07 | 1432,28 | 2599,72 | 3421,5 | 4788,59 | 2265,27 | 1124,01 | 1962,07 | 2372 |
| FA 16:1 | 1849,72 | 1667,83 | 18246,71 | 25890,19 | 10510,29 | 5451,82 | 3855,8 | 4540,91 | 9002 |
| FA 16:1;O | 1847,68 | 1919,75 | 2257,96 | 2037,1 | 3641,92 | 2729,4 | 1241,01 | 2218,71 | 2237 |
| FA 17:0 | 718,53 | 542,47 | 1385,15 | 943,83 | 1545,7 | 1780,54 | 664,1 | 764,42 | 1043 |
| FA 17:1 | 76,2 | 44,52 | 589,49 | 402,26 | 439 | 315,52 | 165,33 | 303,02 | 292 |
| FA 17:4;O | 116,16 | 247,65 | 459,5 | 320,62 | 509,34 | 594,54 | 178,91 | 447,12 | 359 |
| FA 18:0 | 302694,3 | 268674 | 336114,65 | 278328,35 | 451966,15 | 511838 | 248870 | 285043,7 | 335441 |
| FA 18:0;(2OH) | 2455,96 | 1859,66 | 2300,55 | 2457,61 | 3109,49 | 2009,75 | 1187,8 | 2106,42 | 2186 |
| FA 18:0;O | 2643,83 | 1955,2 | 4349,08 | 9244,72 | 13478,35 | 4809,96 | 1729,33 | 4178,03 | 5299 |
| FA 18:1 | 20469,48 | 16192,1 | 60476,4 | 56248,09 | 85826,92 | 58268,5 | 32080,7 | 50632,86 | 47524 |
| FA 18:1;2O | 346,94 | 348,68 | 603,78 | 979,68 | 2437,82 | 667,39 | 214,64 | 535,35 | 767 |
| FA 18:1;O | 1370,87 | 1215,12 | 3099,62 | 6740,84 | 9569,69 | 2904,48 | 1157,78 | 2223,63 | 3535 |
| FA 18:2 | 1288,72 | 1008,32 | 3651,47 | 4017,36 | 4157,51 | 2694,91 | 2389,58 | 2287,27 | 2687 |
| FA 18:2;O | 931,22 | 1258,54 | 2001,16 | 2465,61 | 3725,33 | 1707,98 | 809,06 | 1409,37 | 1789 |
| FA 18:3 | 1120,31 | 980,3 | 4494,62 | 7058,43 | 5430,96 | 1698,17 | 2386,74 | 1545,47 | 3089 |

| FA 18:3;4O | 865,13 | 595,09 | 614,16 | 568,67 | 714,76 | 825,86 | 386,3 | 693,92 | 658 |
| --- | --- | --- | --- | --- | --- | --- | --- | --- | --- |
| FA 18:3;O | 146,28 | 149,28 | 248,99 | 417,56 | 468,54 | 202 | 93,23 | 225,92 | 244 |
| FA 19:0 | 137,39 | 182,49 | 284,14 | 190,36 | 295,22 | 469,97 | 131,69 | 163,3 | 232 |
| FA 19:0;(2OH) | 1007,59 | 1089,7 | 1254,23 | 712,26 | 821,61 | 942,96 | 788,22 | 1054,59 | 959 |
| FA 19:1 | 32,58 | 39,12 | 381,59 | 256,86 | 244,94 | 264,71 | 110,11 | 96,01 | 178 |
| FA 19:1;2O | 1108,63 | 424,85 | 348,75 | 253,86 | 1125,29 | 1021,25 | 222,44 | 421,01 | 616 |
| FA 19:1;O | 233,47 | 117,78 | 673,29 | 1804,22 | 1938,67 | 395,51 | 168,02 | 477,01 | 726 |
| FA 20:0 | 3482,9 | 3489,19 | 4259,42 | 3716,74 | 5177,2 | 4994 | 2915,43 | 2795,44 | 3854 |
| FA 20:1 | 100,64 | 133,96 | 792,32 | 558,15 | 261,55 | 417,8 | 359,36 | 408,9 | 379 |
| FA 20:3 | 95,48 | 38,2 | 354,8 | 280,74 | 316,1 | 378,34 | 272,67 | 336,88 | 259 |
| FA 20:4 | 345,82 | 206,85 | 2585,12 | 1996,27 | 1835,11 | 1169,24 | 1173,47 | 2324,07 | 1454 |
| FA 20:5 | 1114,81 | 352,56 | 511,92 | 821,7 | 599,16 | 518,66 | 328,9 | 1150,76 | 675 |
| FA 21:0 | 380,79 | 444,58 | 354,58 | 365,78 | 540,17 | 406,14 | 319,35 | 263,28 | 384 |
| FA 21:1;2O | 152,34 | 166,2 | 266,73 | 257,62 | 377,18 | 164,06 | 153,87 | 200,4 | 217 |
| FA 22:0 | 1156,09 | 1121,25 | 1821,86 | 1350,91 | 1839,03 | 2435,55 | 1184,86 | 1145,41 | 1507 |
| FA 22:1 | 122,05 | 48,82 | 173,24 | 94,89 | 340,83 | 199,38 | 93,01 | 127,25 | 150 |
| FA 22:5 | 258,87 | 225,51 | 1138,44 | 652,28 | 1368,97 | 1529,26 | 700,97 | 1233,28 | 888 |
| FA 22:6 | 723,33 | 780,26 | 1621,83 | 1276,49 | 1237,27 | 960,19 | 626,28 | 1263,75 | 1061 |
| FA 23:0 | 663,67 | 1112,39 | 909,7 | 634,11 | 1286,99 | 1565,76 | 743,48 | 764,17 | 960 |
| FA 23:1 | 12,3 | 72,16 | 188,93 | 136,06 | 289,36 | 220,91 | 180,45 | 98,94 | 150 |
| FA 24:0 | 4357,17 | 5685,69 | 6854,23 | 5080,55 | 9217,81 | 9869,78 | 4931,89 | 5101,36 | 6387 |
| FA 25:0 | 2340 | 2632,84 | 2451,63 | 2058,52 | 4379,87 | 3719,49 | 1613,6 | 1748,73 | 2618 |
| FA 26:0 | 7136,42 | 9835,62 | 10565,87 | 10723,35 | 19995,99 | 16851,8 | 8822,61 | 9480,66 | 11677 |
| FA 26:1;O | 315,15 | 823,31 | 849,13 | 750,03 | 826,27 | 673,15 | 474,85 | 904,2 | 702 |
| FA 27:0 | 2118,78 | 2378,67 | 3454,53 | 5765,45 | 6652,8 | 5153,32 | 4619,26 | 3107,73 | 4156 |
| FA 27:1;O | 150,64 | 239,72 | 312,58 | 266,77 | 240,12 | 233,41 | 177,16 | 377,13 | 250 |
| FA 28:0 | 10830,6 | 12000,4 | 14378,17 | 16783,8 | 25522,22 | 19236,9 | 12240,8 | 11679,78 | 15334 |
| FA 29:0 | 2634,93 | 2630,1 | 3809,72 | 3951,63 | 6937,27 | 4758,25 | 2942,59 | 3469,27 | 3892 |
| FA 30:0 | 8673,16 | 9260,65 | 19373,79 | 27267,09 | 36982,21 | 26657 | 18807,1 | 19774,86 | 20849 |
| FA 31:0 | 1174,48 | 1501,08 | 2179,71 | 2741,75 | 3479,94 | 3412,29 | 2059 | 2032,95 | 2323 |
| FA 32:0 | 2740,8 | 2602,95 | 6413,21 | 7461,86 | 12539,53 | 9123,98 | 5670,65 | 6355,26 | 6614 |
| FA 33:0 | 376,78 | 280,64 | 699,16 | 950,28 | 797,88 | 786,61 | 503,21 | 466,9 | 608 |
| FA 34:0 | 738,1 | 430,82 | 1164,81 | 1524,19 | 2504,79 | 1426,82 | 1138,05 | 795,88 | 1215 |
| FA 34:8 | 110,88 | 163,11 | 1094,2 | 1687,62 | 1418,16 | 517,97 | 1340,92 | 1116,82 | 931 |
| FA 42:5 | 529726,6 | 621766 | 525511,5 | 486638,45 | 671814,35 | 780636 | 440821 | 776514,25 | 604179 |
| FA 42:6 | 430,63 | 751,14 | 749,44 | 591,58 | 685,79 | 862,84 | 433,9 | 874,99 | 673 |
| FA 44:5 | 157275,8 | 174431 | 152331,6 | 183130,1 | 242400,25 | 219259 | 155609 | 227060,8 | 188937 |
| FA 44:6 | 262,83 | 431,01 | 466,41 | 583,83 | 542,71 | 577,86 | 311,66 | 582,86 | 470 |
| **Dihexosylceramides** |  |  |  |  |  |  |  |  |  |
| Hex2Cer 32:1;2O|Hex2Cer 16:1;2O/16:0 | 135,23 | 98,43 | 154,74 | 1398,62 | 394,14 | 254,67 | 271,75 | 230,84 | 367 |
| Hex2Cer 33:1;2O|Hex2Cer 17:1;2O/16:0 | 106,92 | 101,52 | 136,18 | 1732,34 | 326,67 | 280,44 | 280,51 | 257,99 | 403 |
| Hex2Cer 34:0;2O | 208,3 | 216,54 | 583,23 | 2040,57 | 1940,13 | 989,82 | 1076,6 | 1064,17 | 1015 |
| Hex2Cer 34:1;2O|Hex2Cer 18:1;2O/16:0 | 1131,26 | 1396,55 | 4753,22 | 6842,61 | 14814,08 | 5613,46 | 8399,68 | 9392,44 | 6543 |
| Hex2Cer 36:1;2O|Hex2Cer 18:1;2O/18:0 | 81,79 | 60,48 | 148,97 | 953,05 | 457,34 | 138,05 | 258,54 | 294,96 | 299 |
| Hex2Cer 38:1;2O|Hex2Cer 16:1;2O/22:0 | 376,75 | 339,07 | 448,82 | 1500,67 | 1144,34 | 719,73 | 1536,21 | 933,78 | 875 |

| Hex2Cer 39:0;2O|Hex2Cer 17:0;2O/22:0 | 379,92 | 321,81 | 498,54 | 2139,71 | 1006,43 | 784,13 | 1350,54 | 990,66 | 934 |
| --- | --- | --- | --- | --- | --- | --- | --- | --- | --- |
| Hex2Cer 39:1;2O|Hex2Cer 16:1;2O/23:0 | 440,01 | 395,23 | 643,77 | 1850,6 | 1703,59 | 700,84 | 1530,21 | 1428,89 | 1087 |
| Hex2Cer 40:1;2O|Hex2Cer 18:1;2O/22:0 | 940 | 889,43 | 1361,11 | 5335,69 | 4784,16 | 2192,01 | 2907,1 | 3134,72 | 2693 |
| Hex2Cer 40:2;2O|Hex2Cer 16:1;2O/24:1 | 42,47 | 38,62 | 66,31 | 74,75 | 185,81 | 69,83 | 220,02 | 108,6 | 101 |
| Hex2Cer 41:1;2O|Hex2Cer 18:1;2O/23:0 | 529,95 | 515,57 | 603,78 | 6711,23 | 1582,06 | 1561,03 | 1551,86 | 2004,13 | 1882 |
| Hex2Cer 42:1;2O|Hex2Cer 18:1;2O/24:0 | 611,58 | 591,4 | 500,13 | 4447,04 | 1774,27 | 1465,92 | 903,33 | 1188,49 | 1435 |
| Hex2Cer 42:2;2O|Hex2Cer 18:1;2O/24:1 | 97,04 | 68,04 | 216,08 | 989,62 | 727,95 | 325,37 | 338,78 | 403,92 | 396 |
|  |  |  |  |  |  |  |  |  |  |
| **Hexosylceramides** |  |  |  |  |  |  |  |  |  |
|  |  |  |  |  |  |  |  |  |  |
| HexCer 33:1;2O|HexCer 17:1;2O/16:0 | 228,57 | 233,42 | 503,56 | 1027,21 | 1334,06 | 848,63 | 545,86 | 519,03 | 655 |
| HexCer 34:0;2O|HexCer 18:0;2O/16:0 | 131,28 | 130,75 | 243,34 | 623,08 | 1077,9 | 336,97 | 333,64 | 268,85 | 393 |
| HexCer 34:1;2O | 755,09 | 568,3 | 1195,9 | 2462,61 | 2524,98 | 1176,95 | 956,58 | 703,37 | 1293 |
| HexCer 34:1;2O|HexCer 18:1;2O/16:0 | 817,22 | 900,43 | 1445,32 | 4269,67 | 4628,06 | 1733,89 | 1555,43 | 1531,08 | 2110 |
| HexCer 34:1;3O|HexCer 18:1;2O/16:0;O | 380,31 | 361,26 | 545,64 | 1285,32 | 1982,1 | 1004,98 | 518,79 | 731,03 | 851 |
| HexCer 38:1;2O | 104,41 | 164,08 | 209,07 | 474,13 | 146,54 | 119,59 | 371,7 | 299,85 | 236 |
| HexCer 39:1;3O | 918,19 | 791,28 | 763,66 | 1401,94 | 967,66 | 922,54 | 666,54 | 1034,34 | 933 |
| HexCer 40:0;3O | 112,65 | 150,73 | 344,98 | 1194,32 | 325,79 | 279,84 | 470,11 | 416,69 | 412 |
| HexCer 40:1;2O | 678,97 | 556,52 | 2256,1 | 8594,78 | 4149,99 | 1738,94 | 3714,42 | 2617,55 | 3038 |
| HexCer 40:1;2O|HexCer 18:1;2O/22:0 | 558,04 | 715,08 | 413,8 | 4338,02 | 1532,57 | 703,66 | 369,93 | 746,99 | 1172 |
| HexCer 40:1;3O | 1496,2 | 1444,19 | 1488,12 | 2883,06 | 2230,31 | 2851,64 | 1673,81 | 1831,24 | 1987 |
| HexCer 41:1;2O | 811,07 | 707,75 | 1801,88 | 4315,2 | 4195,85 | 1717,47 | 2396,07 | 1895,19 | 2230 |
| HexCer 41:1;2O|HexCer 18:1;2O/23:0 | 3422,83 | 2486,17 | 3069,28 | 18318,27 | 17590,31 | 8411,47 | 4590,01 | 5128,12 | 7877 |
| HexCer 41:1;3O | 1531,26 | 1480,41 | 2350,39 | 8677,55 | 5604,32 | 3878 | 2573,65 | 3229,41 | 3666 |
| HexCer 42:1;2O | 681,77 | 560,35 | 3795,55 | 9693,57 | 7924,03 | 2844,94 | 4644,74 | 3831,51 | 4247 |
| HexCer 42:1;3O | 1777,9 | 1365,42 | 1924,13 | 5652,29 | 4421,97 | 3158,19 | 1777,16 | 2390,13 | 2808 |
| HexCer 43:1;3O | 234 | 214,56 | 506,19 | 1171,36 | 761,12 | 527,51 | 581,48 | 749,99 | 593 |
| HexCer 49:5;4O | 1095,02 | 877,96 | 1157,08 | 2473,11 | 2858 | 1466,61 | 2543,98 | 1074,44 | 1693 |
| HexCer 51:9;3O | 187,3 | 185,48 | 312,27 | 1137,96 | 247,24 | 499,22 | 809,68 | 666,14 | 506 |
| HexCer 53:9;3O | 290,08 | 293,67 | 273,56 | 3208,82 | 1048,67 | 471,94 | 662,31 | 563,22 | 852 |
| **Lysophophatidylcholines** |  |  |  |  |  |  |  |  |  |
| LPC 14:0 | 1343,82 | 677,38 | 1017,95 | 3314,78 | 2139,73 | 1620,6 | 2074,86 | 1251,88 | 1680 |
| LPC 15:0 | 1148,35 | 698,74 | 1151,53 | 3389,92 | 1868,42 | 1090,47 | 1245,48 | 1552,64 | 1518 |
| LPC 16:0 | 5929,12 | 4064,96 | 8139,56 | 18456,92 | 14787,62 | 14371,9 | 13763,2 | 11551,52 | 11383 |
| LPC 17:0 | 287,4 | 174,45 | 216,19 | 2032,21 | 611,73 | 487,26 | 346,54 | 357,92 | 564 |
| LPC 18:0 | 1173,21 | 699,25 | 832 | 3280,35 | 2461,56 | 2398,84 | 2252,91 | 2311,62 | 1926 |
| LPC 18:1 | 1746,99 | 1322,53 | 4054,86 | 6822,36 | 4989,57 | 4799,78 | 3413,44 | 5850,19 | 4125 |
| LPC 18:2 | 1460,32 | 680,61 | 1111,2 | 11377,37 | 2798,49 | 1655,24 | 1088,23 | 1555,07 | 2716 |
| LPC 26:0 | 356,29 | 389,77 | 385,57 | 822,58 | 717,32 | 600,83 | 559,55 | 519,96 | 544 |
| LPC 28:7 | 445,16 | 72,83 | 98,51 | 2127,21 | 902,77 | 474,73 | 134,77 | 193,13 | 556 |
| **Lysophosphatidylethanolamines** |  |  |  |  |  |  |  |  |  |
| LPE 16:0 | 1037,16 | 1166,31 | 1280,62 | 2649,84 | 2948,5 | 1866,86 | 2120,4 | 1595,64 | 1833 |
| LPE 16:1 | 200,48 | 133,9 | 220,23 | 864,73 | 876,92 | 207,57 | 138,06 | 185,52 | 353 |
| LPE 18:0 | 249,56 | 153,73 | 222,16 | 4362,6 | 620,26 | 398,2 | 594,34 | 627,98 | 904 |
| LPE 18:1 | 4716,69 | 3807,66 | 6513,84 | 9458,15 | 10420,58 | 8086,58 | 6185,46 | 9751,5 | 7368 |
| LPE 18:2 | 1342,27 | 1171,08 | 1433,35 | 2774,52 | 2566,55 | 1862,44 | 1392,87 | 1698,83 | 1780 |
| LPE 18:3 | 356,58 | 295,47 | 339,28 | 744,74 | 954,44 | 589,13 | 334,94 | 295,08 | 489 |
| LPE 20:4 | 194,42 | 125,12 | 219,8 | 206,82 | 306,73 | 171,42 | 112,43 | 311,88 | 206 |

| LPI 18:0 | 1809,24 | 7457,26 | 3971,74 | 2175,23 | 5445,13 | 1837,43 | 11626,5 | 6214,45 | 5067 |
| --- | --- | --- | --- | --- | --- | --- | --- | --- | --- |
| LPI 18:1 | 1682,19 | 6870,12 | 3741,05 | 2718,51 | 6451,34 | 2009,16 | 7584,47 | 5002,77 | 4507 |
| LPI 18:2 | 344,27 | 745,42 | 447,9 | 341,13 | 861,24 | 496,35 | 838,51 | 633,18 | 589 |
| **Monoacylglycerols** |  |  |  |  |  |  |  |  |  |
| MG 15:0 | 237,26 | 270,1 | 334,45 | 2418,39 | 821,29 | 2234,32 | 706,95 | 640,59 | 958 |
| MG 17:3 | 3009,35 | 1684,19 | 763,94 | 24649,32 | 4821,48 | 3232,12 | 1082,39 | 2247,47 | 5186 |
| MG 17:4 | 4487,99 | 2119,3 | 2356,36 | 11407,6 | 9123,94 | 3040,71 | 1694,97 | 4448,76 | 4835 |
| MG 18:0 | 3838,95 | 1986,06 | 1465,41 | 4318,72 | 7348,81 | 5705,6 | 2020,05 | 3473,69 | 3770 |
| MG 19:3 | 884,27 | 323,37 | 281,31 | 10549,87 | 1476,7 | 964,58 | 311,72 | 821,97 | 1952 |
| MG 21:4 | 874,85 | 560,38 | 1017,12 | 3345,66 | 6673,46 | 3760,21 | 1994,14 | 3075,22 | 2663 |
| MG 22:1 | 848,81 | 411,59 | 504,44 | 17119 | 2676,35 | 1980,72 | 639,46 | 991,15 | 3146 |
| MG 24:2 | 249,51 | 233,95 | 311,4 | 1267,94 | 711,29 | 491,84 | 157,19 | 325,53 | 469 |
| MG 30:7 | 4551,58 | 2319,05 | 3565,95 | 17735,13 | 11671,51 | 9541,87 | 6202,54 | 8197,09 | 7973 |
| MG 32:8 | 617,71 | 410,87 | 552,61 | 12992,2 | 2954,19 | 1829,57 | 940,18 | 1492,05 | 2724 |
| MG 34:8 | 1591,13 | 1015,85 | 980,46 | 15215,85 | 7826,45 | 4805,08 | 1629,1 | 2989,07 | 4507 |
| MG 36:6 | 246,56 | 217,2 | 499,64 | 1657,38 | 2326,51 | 1901,29 | 1241,38 | 1167,67 | 1157 |
| **N-acyl ethanolamines** |  |  |  |  |  |  |  |  |  |
| NAE 12:0 | 3306,45 | 2180,58 | 3669,38 | 6822,26 | 7043,41 | 5400,91 | 2246,86 | 4092,84 | 4345 |
| NAE 13:1 | 9989,19 | 3296,87 | 4254,03 | 8345,9 | 18168,1 | 6874,35 | 3455,54 | 8682 | 7883 |
| NAE 14:1 | 13567,4 | 5337,7 | 7856,7 | 16156,93 | 28655,05 | 9669,54 | 6232,05 | 13508,23 | 12623 |
| NAE 15:1 | 1773,3 | 888,02 | 1264,84 | 2194,8 | 3889,94 | 1741,34 | 915,68 | 2145,09 | 1852 |
| NAE 16:1 | 426143,6 | 228577 | 384568,7 | 451784,05 | 870965,05 | 343531 | 235427 | 563845,6 | 438105 |
| NAE 16:2 | 917285,5 | 521899 | 857651,85 | 1205025,65 | 2096362 | 830310 | 517772 | 1261957,5 | 1026033 |
| NAE 16:3 | 559286,1 | 313657 | 540353,55 | 624904,2 | 1050788,4 | 552955 | 342837 | 767709,35 | 594061 |
| NAE 16:4 | 42207,35 | 22506 | 37832,13 | 81624,54 | 111826,11 | 38524,9 | 21630 | 53607,46 | 51220 |
| NAE 17:4 | 6904,92 | 3310,18 | 3657,32 | 5887,48 | 12389,67 | 6022,08 | 3091,62 | 5774,59 | 5880 |
| NAE 18:1 | 12999,89 | 8915,83 | 16424,14 | 21612,96 | 30551,27 | 10723,3 | 9093,67 | 20648,49 | 16371 |
| NAE 18:2 | 19745,73 | 12687,4 | 24785,47 | 31780,04 | 44648,5 | 16206,4 | 11768,5 | 33416,26 | 24380 |
| NAE 18:3 | 3687,83 | 2082,02 | 3031,58 | 3844,53 | 7769,69 | 4362,55 | 1819,77 | 4453,09 | 3881 |
| NAE 18:4 | 101926,3 | 64208,8 | 80306,56 | 108731,76 | 247712 | 110043 | 54140 | 119721,05 | 110849 |
| NAE 18:5 | 163512,2 | 90020,2 | 121537,25 | 236695,5 | 371403,25 | 227935 | 87207,4 | 185875,9 | 185523 |
| NAE 20:1 | 1767,53 | 1228,69 | 1656,93 | 3286,77 | 4402,93 | 4112,03 | 1269,29 | 2318,36 | 2505 |
| NAE 20:2 | 3894,09 | 2978,19 | 5170,05 | 9415,05 | 9926,93 | 6670,32 | 3007,21 | 7733,1 | 6099 |
| NAE 20:4 | 3651,28 | 3510,6 | 4740,87 | 3867,3 | 8538,26 | 4196,78 | 2957,71 | 5123,13 | 4573 |
| NAE 20:5 | 3197,73 | 2231,73 | 3600,79 | 8825,58 | 8970,09 | 3666,92 | 1918,29 | 4632,76 | 4630 |
| NAE 22:3 | 1449,2 | 989,69 | 1628,56 | 4766,79 | 2757,53 | 1891,83 | 1136,85 | 1728,73 | 2044 |
| NAE 22:4 | 846,05 | 639,68 | 1081 | 1715,79 | 2192,43 | 2135,97 | 823,2 | 1455,67 | 1361 |
| NAE 22:5 | 1056,75 | 644,56 | 1091,62 | 5587,79 | 2841,35 | 1903,96 | 864,41 | 1514,68 | 1938 |
| NAE 24:4 | 1823,27 | 745,34 | 751,58 | 1915,3 | 2723 | 1399,95 | 780,23 | 1509,53 | 1456 |
| NAE 5:0 | 13181,07 | 2429,08 | 1322,85 | 23960,05 | 15143,49 | 4505,86 | 2549,88 | 4803,28 | 8487 |
| NAE 6:0 | 58795,99 | 10110,3 | 8763,65 | 110201,25 | 83679,97 | 23225 | 10565,1 | 15288,52 | 40079 |
| NAE 7:0 | 383,11 | 177,82 | 380,36 | 305,43 | 1145,53 | 605,3 | 98,55 | 456,67 | 444 |
| **Phosphatidylcolines** |  |  |  |  |  |  |  |  |  |
| PC 24:0 | 288,87 | 270,89 | 213,55 | 1140,39 | 446,67 | 561,43 | 1564,66 | 510,21 | 625 |
| PC 26:0|PC 12:0_14:0 | 974,96 | 779,69 | 751,04 | 4303,25 | 1780,93 | 1590,57 | 6739,67 | 1179,29 | 2262 |

| PC 27:0|PC 12:0_15:0 | 222,83 | 184,35 | 208,19 | 1840,83 | 568,98 | 358,21 | 810,62 | 237,67 | 554 |
| --- | --- | --- | --- | --- | --- | --- | --- | --- | --- |
| PC 28:0 | 409,71 | 406,15 | 363,03 | 2402,95 | 1014,51 | 746,03 | 1459,34 | 579,47 | 923 |
| PC 28:0|PC 14:0_14:0 | 16812,97 | 17937,7 | 20033,39 | 29742,57 | 37146,89 | 24590,1 | 99163,6 | 23783,62 | 33651 |
| PC 28:1 | 254,7 | 225,64 | 190,01 | 3214,79 | 814,35 | 438,74 | 752,99 | 281,61 | 772 |
| PC 29:0|PC 14:0_15:0 | 3428,66 | 3193,4 | 4670,85 | 8648,15 | 8682,24 | 5616,85 | 12276,8 | 5570,2 | 6511 |
| PC 30:0|PC 14:0_16:0 | 288038,2 | 289963 | 377609,35 | 494896,7 | 772134,35 | 533924 | 786286 | 507679,75 | 506316 |
| PC 30:1|PC 14:0_16:1 | 2549,53 | 2377,7 | 3206,85 | 14351,97 | 11376,17 | 5165,02 | 9164,78 | 4487 | 6585 |
| PC 30:3 | 420,38 | 322,37 | 311,23 | 2253,82 | 841,86 | 665,56 | 734,33 | 609,31 | 770 |
| PC 31:0|PC 15:0_16:0 | 22678,37 | 23928,6 | 21934,91 | 40575,12 | 40078,09 | 37755,9 | 30842,8 | 26525,71 | 30540 |
| PC 31:1 | 961,55 | 883,06 | 1331,49 | 5069,7 | 3215,74 | 1795,1 | 2825,2 | 2351,61 | 2304 |
| PC 31:1|PC 15:0_16:1 | 804,27 | 732,08 | 1073,19 | 5920,13 | 3706,07 | 1843,26 | 1736,65 | 1665,99 | 2185 |
| PC 32:0 | 6352,63 | 7917,03 | 7992,53 | 15391,66 | 14979,71 | 15458,3 | 12520,1 | 11413,51 | 11503 |
| PC 32:0|PC 16:0_16:0 | 318928,6 | 277352 | 336764,1 | 599550,25 | 889030,2 | 774940 | 593504 | 503059,05 | 536641 |
| PC 32:1 | 1123,11 | 1236,83 | 1627,43 | 5289,19 | 4945,48 | 2685,87 | 1939,85 | 2678,84 | 2691 |
| PC 32:1|PC 14:0_18:1 | 73451,85 | 72193,6 | 126465,95 | 279563,4 | 390048,8 | 186368 | 191185 | 205985,9 | 190658 |
| PC 32:2|PC 16:1_16:1 | 3631,36 | 3522,28 | 4664,65 | 12473,81 | 10409,55 | 5863,98 | 13384,6 | 6243,41 | 7524 |
| PC 32:3|PC 14:0_18:3 | 619,79 | 532,99 | 441,37 | 3190,22 | 1547,42 | 909,84 | 1618,26 | 695,46 | 1194 |
| PC 32:3|PC 14:1_18:2 | 4513,93 | 5920,36 | 9837,72 | 9625,98 | 11810,74 | 7912,23 | 20025 | 10247,89 | 9987 |
| PC 33:0|PC 16:0_17:0 | 9447,28 | 8630,17 | 7374 | 22527,57 | 19505,17 | 15196,9 | 7909,72 | 12641,55 | 12904 |
| PC 33:1|PC 15:0_18:1 | 13260,07 | 11601,3 | 19130,73 | 41950,56 | 33945,02 | 30572,2 | 26507 | 32391,45 | 26170 |
| PC 33:2 | 804,52 | 1347,38 | 1431,37 | 5576,34 | 3503,63 | 2474,26 | 2035,54 | 2606,23 | 2472 |
| PC 34:0|PC 16:0_18:0 | 46635,45 | 45990,8 | 51328,21 | 64681,8 | 85107,29 | 108900 | 96943,3 | 95495,67 | 74385 |
| PC 34:1 | 14049,82 | 13425,5 | 14801,03 | 47035,83 | 30920,11 | 26958,7 | 24932,7 | 35171,91 | 25912 |
| PC 34:1|PC 16:0_18:1 | 566932,7 | 467897 | 685287 | 1562984,5 | 1638208 | 1247128 | 997408 | 1377145,5 | 1067874 |
| PC 34:2 | 3374,22 | 2378,19 | 3543,69 | 5211,35 | 6074,33 | 5669,75 | 3772,23 | 5199,86 | 4403 |
| PC 34:2|PC 15:1_19:1 | 835,17 | 706,48 | 936,96 | 8934,3 | 2224,05 | 1801,11 | 1133,63 | 1895,7 | 2308 |
| PC 34:2|PC 16:0_18:2 | 124329,8 | 119946 | 129838,15 | 290166,7 | 288085,75 | 216823 | 184730 | 154581 | 188563 |
| PC 34:3|PC 16:0_18:3 | 18008,33 | 20419,3 | 22151,11 | 66266,65 | 82288,93 | 37714,1 | 54159,3 | 25927,08 | 40867 |
| PC 34:4|PC 17:2_17:2 | 258,66 | 250,8 | 237,9 | 3254,92 | 1477,48 | 604,29 | 511,39 | 413,21 | 876 |
| PC 35:0|PC 17:0_18:0 | 1715,54 | 1337,4 | 1282,51 | 4889,55 | 3278,93 | 3337,58 | 2427,59 | 2877,25 | 2643 |
| PC 35:1|PC 17:0_18:1 | 6682,09 | 6093,54 | 7144,88 | 12822,63 | 12355,08 | 11787,3 | 7344,36 | 12263,52 | 9562 |
| PC 35:2|PC 17:1_18:1 | 3141,62 | 2798,86 | 5457,24 | 11468,01 | 9421,48 | 8539,93 | 7006,38 | 9614,13 | 7181 |
| PC 35:3 | 633,85 | 587,4 | 923,24 | 2087,64 | 2600,25 | 1566,91 | 1321,1 | 1376,55 | 1387 |
| PC 36:0|PC 18:0_18:0 | 1585,01 | 1516,91 | 1447,04 | 5171,01 | 2879,18 | 2961,67 | 2488,69 | 2781,78 | 2604 |
| PC 36:1 | 699,35 | 514,7 | 774,05 | 982,67 | 863,4 | 1044,96 | 984,87 | 1448,02 | 914 |
| PC 36:1|PC 18:0_18:1 | 84934,08 | 72981,9 | 128490,3 | 166026,5 | 195792,9 | 206408 | 203324 | 266540,45 | 165562 |
| PC 36:2 | 1837,49 | 1867,74 | 1478,83 | 6507,56 | 2856,73 | 3268,23 | 1087,73 | 3902,29 | 2851 |
| PC 36:2|PC 18:1_18:1 | 112280 | 99999,8 | 98689,98 | 246010,6 | 165766,4 | 182246 | 78994,3 | 272323 | 157039 |
| PC 36:3 | 1127,18 | 1239,65 | 1348,2 | 5940,53 | 2096,58 | 2102,81 | 1381,08 | 1961,82 | 2150 |
| PC 36:3|PC 18:1_18:2 | 48405,14 | 43636,1 | 55870,98 | 131032,14 | 106453,75 | 103793 | 87151,1 | 98852,84 | 84399 |
| PC 36:4 | 517,37 | 510,41 | 632,68 | 4261,93 | 1579,78 | 950,19 | 873,11 | 867,49 | 1274 |
| PC 36:4|PC 18:2_18:2 | 16013,08 | 15043,8 | 22473,16 | 85266,25 | 61001,57 | 36869,4 | 39975,4 | 41167,19 | 39726 |
| PC 36:5|PC 16:0_20:5 | 2499,8 | 2450,15 | 3349,79 | 18725,17 | 8968,82 | 7073,89 | 4691,11 | 3662,8 | 6428 |
| PC 36:5|PC 18:2_18:3 | 1296,26 | 1138,74 | 942,52 | 13366,01 | 3830,49 | 2355,62 | 2238,72 | 1562,94 | 3341 |
| PC 36:6 | 159,67 | 117,7 | 83,2 | 3086,11 | 500,61 | 251,88 | 181,78 | 147,71 | 566 |
| PC 37:1|PC 19:0_18:1 | 600,59 | 402,92 | 507,37 | 4417,61 | 657,09 | 892,06 | 402,75 | 672,94 | 1069 |

| PC 37:2|PC 18:1_19:1 | 724,55 | 646,9 | 1003,07 | 4498,25 | 1922,17 | 1696,61 | 1263,58 | 1653,26 | 1676 |
| --- | --- | --- | --- | --- | --- | --- | --- | --- | --- |
| PC 38:1|PC 20:0_18:1 | 992,57 | 895,17 | 1079,31 | 3609,41 | 1821,2 | 2337,33 | 896,4 | 2016,28 | 1706 |
| PC 38:2 | 939,69 | 695,05 | 794,56 | 3338,79 | 1919,47 | 1630,73 | 1013,27 | 1592,48 | 1491 |
| PC 38:2|PC 20:0_18:2 | 2974,56 | 2446,72 | 2363,66 | 6033,03 | 4096,76 | 4902,21 | 2049,35 | 4053,76 | 3615 |
| PC 38:3 | 1248,52 | 1118,63 | 1436,87 | 4720,03 | 2870,49 | 3027,43 | 2320,28 | 2327,24 | 2384 |
| PC 38:3|PC 20:0_18:3 | 839,88 | 714,49 | 1085,94 | 2994,37 | 1766,25 | 2049,78 | 1259,32 | 1447,35 | 1520 |
| PC 38:4 | 1219,03 | 1094,75 | 1673,15 | 6180,81 | 3445,18 | 4091,62 | 2253,4 | 2769,1 | 2841 |
| PC 38:4|PC 18:1_20:3 | 1362,92 | 1250,85 | 2705,89 | 6894,38 | 4620,69 | 5258,03 | 3996,77 | 5485,78 | 3947 |
| PC 38:5|PC 16:0_22:5 | 3778,21 | 3663,11 | 8512,1 | 15559,52 | 14330,61 | 10887,3 | 8468,71 | 13372,5 | 9822 |
| PC 38:6|PC 19:3_19:3 | 838,4 | 918,92 | 1817,08 | 4614,98 | 3246,19 | 3393,82 | 2303,01 | 2462,81 | 2449 |
| PC 38:7 | 306,42 | 255,65 | 272,68 | 3124,92 | 938,92 | 744,22 | 337,78 | 453,16 | 804 |
| PC 40:3|PC 25:1_15:2 | 337,48 | 346,55 | 430,05 | 920,8 | 657,78 | 918,77 | 366,82 | 788,57 | 596 |
| PC 40:5|PC 18:0_22:5 | 639,17 | 592,97 | 920,49 | 4256,32 | 1885,73 | 1994,26 | 1332,09 | 1991,05 | 1702 |
| PC 40:6 | 815,67 | 720,04 | 1417,46 | 5020,56 | 2961,09 | 2568,15 | 2067,15 | 3452,94 | 2378 |
| PC O-29:1 | 310,48 | 362,72 | 422,33 | 1476,07 | 884,08 | 497,49 | 593,8 | 628,24 | 647 |
| PC O-31:0 | 3349,82 | 4135,44 | 6737,95 | 7438,34 | 16012,87 | 10305,4 | 6187,05 | 10389,66 | 8070 |
| PC O-31:1 | 1416,53 | 1240,02 | 1819,68 | 3901,3 | 3574,05 | 2717,94 | 2081,43 | 2974,52 | 2466 |
| PC O-31:6 | 132,34 | 82,54 | 58,5 | 771,32 | 312,01 | 183,52 | 177,6 | 132,52 | 231 |
| PC O-33:2 | 1808,76 | 1585,07 | 2583,03 | 5381,61 | 3853,35 | 3268,85 | 2465,97 | 5332,88 | 3285 |
| PC O-33:3 | 1224,95 | 1368,52 | 1129,94 | 4996,38 | 2673,74 | 1564,51 | 1440,5 | 2195,08 | 2074 |
| PC O-33:4 | 2851 | 4344 | 8637 | 7123 | 8812,71 | 5582,63 | 5888,57 | 10035,38 | 6659 |
| PC O-33:6 | 300,51 | 315,55 | 290,73 | 2038,55 | 935,47 | 608,65 | 573,46 | 487,93 | 694 |
| PC O-35:6 | 697,35 | 574,13 | 812,65 | 3052,45 | 2376,7 | 1590,67 | 1356,55 | 1027,85 | 1436 |
| PC O-35:9 | 836,78 | 1091,64 | 1777,59 | 2966,37 | 2200,51 | 1774,83 | 2849,14 | 2031,89 | 1941 |
| PC O-37:6 | 234,48 | 207,48 | 219,65 | 1689,47 | 628,47 | 466,83 | 414,46 | 453,79 | 539 |
| PC O-38:10 | 993,06 | 724,68 | 545,05 | 6075,49 | 2948,36 | 1304,69 | 1451,12 | 1092,34 | 1892 |
| PC O-39:0 | 1269,34 | 966,31 | 639,21 | 4610,93 | 1277,33 | 1130,81 | 483,14 | 1062,59 | 1430 |
| PC O-39:10 | 2369,59 | 2113,51 | 2390,13 | 5577,23 | 4176,29 | 3944,85 | 2714,35 | 5204,2 | 3561 |
| **Phosphatidylethanolamines** |  |  |  |  |  |  |  |  |  |
| PE 28:0|PE 14:0_14:0 | 1141,37 | 697,42 | 771,41 | 1420,22 | 1072,86 | 893,74 | 1233,9 | 1042,87 | 1034 |
| PE 30:0|PE 14:0_16:0 | 4003,87 | 3604,88 | 2906,51 | 3600,62 | 3339 | 6285,66 | 3004,94 | 3291,6 | 3755 |
| PE 30:1|PE 12:0_18:1 | 890,76 | 707,03 | 990,72 | 2694,72 | 2162,64 | 1416,31 | 1917,2 | 1406,8 | 1523 |
| PE 31:0|PE 15:0_16:0 | 634,28 | 557,69 | 472,97 | 594,11 | 640,96 | 1000,27 | 510,44 | 661,48 | 634 |
| PE 31:1 | 184,95 | 144,09 | 237,67 | 2320,7 | 954,68 | 429,21 | 282,28 | 311,02 | 608 |
| PE 31:1|PE 15:0_16:1 | 103,62 | 135,37 | 145,51 | 488,72 | 246,03 | 370,03 | 143,65 | 183,87 | 227 |
| PE 32:0|PE 16:0_16:0 | 7901,53 | 6122,03 | 4760,16 | 7465,71 | 8286,04 | 16126,4 | 3714,92 | 7268,22 | 7706 |
| PE 32:1|PE 14:0_18:1 | 17784 | 12422,2 | 17204,33 | 51511,34 | 47399,78 | 39550,8 | 16758,7 | 17594,91 | 27528 |
| PE 32:1|PE 16:0_16:1 | 3527,2 | 3428,89 | 4839,45 | 21064,22 | 16109,47 | 7198,15 | 6446,38 | 5961,55 | 8572 |
| PE 32:2 | 431,24 | 331,07 | 326,08 | 3507,44 | 1548,92 | 807,06 | 674,58 | 527,24 | 1019 |
| PE 32:2|PE 14:0_18:2 | 2189,09 | 1778,66 | 2176,95 | 6535,32 | 5904,64 | 4234,72 | 4176,73 | 2448,79 | 3681 |
| PE 32:3|PE 14:0_18:3 | 113,3 | 77,15 | 121,01 | 543,03 | 209,68 | 211,98 | 382,86 | 114,55 | 222 |
| PE 33:0|PE 16:0_17:0 | 1000,66 | 771,76 | 664,9 | 814,48 | 871,13 | 1120,79 | 696,72 | 1041,75 | 873 |
| PE 33:1|PE 15:0_18:1 | 5212,75 | 3889,09 | 4434,91 | 8155,74 | 8551,65 | 9371,86 | 3807,52 | 5292,9 | 6090 |
| PE 34:0|PE 16:0_18:0 | 4077,18 | 2791,92 | 2778,85 | 3484,92 | 2785,28 | 7075,65 | 2290,95 | 5707,11 | 3874 |
| PE 34:1 | 3222,05 | 2546,54 | 2811,05 | 18774,78 | 10869,68 | 5825,62 | 3452,62 | 4600,43 | 6513 |

| PE 34:1|PE 16:0_18:1 | 167481 | 118668 | 157204,05 | 304430,5 | 305671,1 | 329361 | 125099 | 215936,25 | 215481 |
| --- | --- | --- | --- | --- | --- | --- | --- | --- | --- |
| PE 34:2 | 895,53 | 748,58 | 767,05 | 6192,4 | 3117,22 | 1698,57 | 1203,4 | 1016,49 | 1955 |
| PE 34:2|PE 16:0_18:2 | 73743,7 | 50434,1 | 57921,94 | 153421,55 | 155723,25 | 137946 | 69301,1 | 63169,54 | 95208 |
| PE 34:3|PE 16:0_18:3 | 10956,8 | 7528,2 | 5643,74 | 23127,22 | 26134,45 | 16387 | 9990,68 | 5361,35 | 13141 |
| PE 34:3|PE 16:1_18:2 | 1900,2 | 1194,4 | 1668,83 | 8220,38 | 7274,77 | 4871,13 | 1714,19 | 1311,89 | 3519 |
| PE 34:4|PE 14:0_20:4 | 252,7 | 203,9 | 349,09 | 1105,01 | 574,23 | 476,93 | 697,44 | 388,74 | 506 |
| PE 34:4|PE 16:1_18:3 | 131,7 | 184,2 | 213,08 | 2259,87 | 1754,31 | 657,62 | 351,54 | 199,56 | 719 |
| PE 35:0|PE 17:0_18:0 | 290,4 | 245,1 | 227,78 | 252,21 | 154,05 | 448,69 | 220,44 | 556,55 | 299 |
| PE 35:1|PE 17:0_18:1 | 8796,96 | 6251,68 | 9170,38 | 10962,69 | 10948,07 | 16234,4 | 8312,79 | 14068,54 | 10593 |
| PE 35:2|PE 17:1_18:1 | 6633,13 | 4300,44 | 5678,97 | 11211,87 | 11235,12 | 16016,6 | 6069,03 | 9315,13 | 8808 |
| PE 35:3|PE 17:1_18:2 | 991,04 | 479,15 | 658,26 | 1861,67 | 1359,72 | 1385,97 | 943,79 | 873,32 | 1069 |
| PE 36:0|PE 18:0_18:0 | 237,81 | 238,76 | 314,28 | 532,94 | 501,51 | 724,32 | 475,35 | 529,41 | 444 |
| PE 36:1|PE 18:0_18:1 | 138265 | 92761 | 140074,05 | 165501,6 | 149604,85 | 264569 | 157368 | 245530,95 | 169209 |
| PE 36:2 | 1478,41 | 1210,88 | 1620,34 | 6817,43 | 4688,26 | 2968,83 | 3431,7 | 3183,64 | 3175 |
| PE 36:2|PE 18:1_18:1 | 396103 | 273095 | 373853,1 | 550851,2 | 579827,2 | 808177 | 345981 | 614865 | 492844 |
| PE 36:3 | 1416 | 1139 | 1178,91 | 5571,49 | 3662,88 | 2872,07 | 1935,7 | 2384,63 | 2520 |
| PE 36:3;2O|PE 14:0_22:3;2O | 757 | 1075 | 1236,32 | 1555,55 | 2066,79 | 1545,43 | 857,28 | 1395,98 | 1311 |
| PE 36:3|PE 18:1_18:2 | 127219,8 | 81453,9 | 83942,61 | 159707,55 | 188888,15 | 224953 | 110620 | 122201,3 | 137373 |
| PE 36:4|PE 16:0_20:4 | 10351,04 | 6903,8 | 8798,37 | 21728,51 | 22262,92 | 21435,3 | 10418,2 | 11388,1 | 14161 |
| PE 36:4|PE 18:1_18:3 | 30495,52 | 19604,8 | 17922,94 | 52653,26 | 52251,53 | 54317,9 | 30369,5 | 23596,83 | 35152 |
| PE 36:5|PE 16:0_20:5 | 3521,63 | 2158,46 | 1670,14 | 5016,54 | 5139,25 | 4882,45 | 1898,24 | 1299,23 | 3198 |
| PE 36:5|PE 18:2_18:3 | 1673,94 | 999,19 | 923,74 | 3880,9 | 3358,35 | 2693,34 | 2342,03 | 1293,25 | 2146 |
| PE 37:1|PE 18:0_19:1 | 747,33 | 715,06 | 1440,22 | 1400,68 | 1051,57 | 1887,38 | 1016,38 | 1549,51 | 1226 |
| PE 37:2|PE 18:1_19:1 | 2786,16 | 1880,95 | 3541,32 | 4273,88 | 4063,86 | 4775,98 | 3109,89 | 4713,55 | 3643 |
| PE 37:3|PE 18:1_19:2 | 642,12 | 539,36 | 700,79 | 1129,3 | 756,15 | 853,5 | 846,91 | 833,57 | 788 |
| PE 38:1|PE 20:0_18:1 | 1261,02 | 899,46 | 2185,66 | 1964,63 | 1430,2 | 3019,89 | 2466,81 | 2759,44 | 1998 |
| PE 38:2|PE 18:1_20:1 | 3983,35 | 2297,63 | 3580,14 | 4710,4 | 4097,44 | 5521,82 | 2663,41 | 5173,77 | 4003 |
| PE 38:3|PE 18:0_20:3 | 5486,42 | 3666,72 | 5751,97 | 8486,45 | 9048,61 | 13706,7 | 9215,5 | 9600,54 | 8120 |
| PE 38:3|PE 18:1_20:2 | 1543,96 | 1119,6 | 1380,61 | 2132,35 | 2568,1 | 3166,28 | 2152,05 | 2514,53 | 2072 |
| PE 38:4|PE 18:0_20:4 | 7220,02 | 5627,93 | 8751,58 | 13482,8 | 13733,3 | 18324,2 | 13319,6 | 13943,78 | 11800 |
| PE 38:4|PE 18:1_20:3 | 6280,15 | 4843,79 | 5991,72 | 10227,55 | 11728,48 | 18217,1 | 6994,7 | 10525,19 | 9351 |
| PE 38:5|PE 16:0_22:5 | 13185,31 | 9130,11 | 13209,93 | 28071,24 | 28991,02 | 31496,9 | 15584,5 | 23127,8 | 20350 |
| PE 38:5|PE 18:1_20:4 | 2237,65 | 2101,66 | 4244,7 | 11067,28 | 10024,85 | 6391,87 | 6463,45 | 9207,72 | 6467 |
| PE 38:6 | 776,31 | 679,86 | 870,29 | 4071,24 | 2777,39 | 2034,68 | 1033,01 | 1649,49 | 1737 |
| PE 39:1|PE 21:0_18:1 | 304,81 | 194,39 | 635,87 | 613,07 | 615,62 | 800,02 | 666,43 | 477,68 | 538 |
| PE 39:2|PE 18:1_21:1 | 500 | 301,49 | 811,32 | 1017,43 | 809,65 | 1269,32 | 616,48 | 840,55 | 771 |
| PE 40:1|PE 22:0_18:1 | 448,24 | 416,16 | 2146,17 | 2794,4 | 2490,28 | 2667,91 | 2212,99 | 1417,28 | 1824 |
| PE 40:2|PE 18:1_22:1 | 225,9 | 180,09 | 545,94 | 885,97 | 858,76 | 1065,41 | 637,61 | 642,97 | 630 |
| PE 40:4|PE 18:0_22:4 | 545,8 | 422,81 | 1045,56 | 1976,26 | 1664,55 | 1313,13 | 1706,91 | 2551,8 | 1403 |
| PE 40:5|PE 18:0_22:5 | 6951,98 | 4423,41 | 7844 | 13249,15 | 13710,38 | 15331,3 | 12696,3 | 16723 | 11366 |
| PE 40:6|PE 18:1_22:5 | 2388,75 | 1914,39 | 3519,78 | 9010,76 | 5931,46 | 4358,01 | 4897,27 | 9347,57 | 5171 |
| PE 41:1|PE 23:0_18:1 | 122,92 | 166,1 | 650,25 | 567,21 | 326,4 | 743,64 | 463,59 | 453,25 | 437 |
| PE 44:10|PE 22:5_22:5 | 1312,45 | 1569,17 | 2056,28 | 4645,45 | 6200,57 | 3619,85 | 2879,36 | 3815,22 | 3262 |
| PE O-19:0_18:1 | 109 | 136 | 395 | 635 | 31,61 | 243,53 | 435,47 | 396,22 | 298 |
| PE O-30:1|PE O-14:1_16:0 | 263,44 | 261,48 | 291,17 | 192,73 | 283,14 | 180,78 | 133,51 | 476,75 | 260 |

| PE O-31:1|PE O-15:1_16:0 | 1221,76 | 1018,11 | 1047,38 | 1047,46 | 967,76 | 1612,95 | 408,73 | 1701,31 | 1128 |
| --- | --- | --- | --- | --- | --- | --- | --- | --- | --- |
| PE O-31:2|PE O-15:1_16:1 | 554,26 | 391,69 | 515,63 | 1437,63 | 997,86 | 1160,82 | 379,52 | 663,29 | 763 |
| PE O-32:1|PE O-16:1_16:0 | 211,84 | 188,73 | 367,86 | 259,79 | 207,85 | 457,92 | 218,31 | 537,71 | 306 |
| PE O-32:2|PE O-14:1_18:1 | 2962,52 | 1682,78 | 2787,81 | 4702,12 | 5123,22 | 4411,99 | 2196,61 | 3742,05 | 3451 |
| PE O-32:3|PE O-14:1_18:2 | 1531,69 | 894,85 | 1053,27 | 1672,66 | 1432,37 | 1617,46 | 1047,93 | 972,13 | 1278 |
| PE O-33:2|PE O-15:1_18:1 | 6352,06 | 4176,61 | 6222,2 | 8356,56 | 7781,27 | 8594,17 | 4956,93 | 8833,11 | 6909 |
| PE O-33:3|PE O-15:1_18:2 | 4077,89 | 2610,34 | 3090,11 | 4073,37 | 3882,06 | 4629,53 | 2804,66 | 3573,69 | 3593 |
| PE O-33:4|PE O-15:1_18:3 | 644,34 | 394,99 | 447,41 | 912,47 | 761,77 | 658,46 | 502,81 | 424,22 | 593 |
| PE O-34:2|PE O-16:1_18:1 | 14902,83 | 10425 | 20780,15 | 24732,62 | 21340,99 | 28548,8 | 15447,5 | 27102,46 | 20410 |
| PE O-34:3|PE O-16:1_18:2 | 9628,03 | 7003,91 | 8905,76 | 14073,04 | 12984,63 | 17046,6 | 9915,36 | 11351,64 | 11364 |
| PE O-34:4|PE O-16:1_18:3 | 2360,7 | 1594,67 | 1965,58 | 3947,33 | 3554,56 | 3610,76 | 2327,15 | 2142,16 | 2688 |
| PE O-35:1 | 1925,94 | 1605,26 | 3032,5 | 12845,33 | 12397,6 | 7551,54 | 4649,07 | 5111,86 | 6140 |
| PE O-35:2|PE O-17:1_18:1 | 1582,1 | 1155,5 | 3199,9 | 3219,57 | 2427,4 | 3665,48 | 2285,15 | 3537,98 | 2634 |
| PE O-35:3|PE O-17:1_18:2 | 1648,21 | 953,92 | 1766,51 | 2000,06 | 2331,22 | 2119,18 | 1422,58 | 1632,4 | 1734 |
| PE O-35:3|PE O-17:2_18:1 | 253,26 | 412,47 | 507,4 | 827,51 | 535,73 | 446 | 502,64 | 887,12 | 547 |
| PE O-35:5|PE O-15:1_20:4 | 717,36 | 564,7 | 780,23 | 1037,66 | 837,26 | 989,53 | 518,79 | 1396,12 | 855 |
| PE O-35:6|PE O-15:1_20:5 | 399,93 | 224,83 | 281,23 | 433,2 | 213,97 | 460,16 | 151,15 | 330,06 | 312 |
| PE O-36:2|PE O-18:0_18:2 | 452,06 | 278,74 | 1282,66 | 762,7 | 4710,94 | 1479,14 | 1841,42 | 791,63 | 1450 |
| PE O-36:2|PE O-18:1_18:1 | 5311,56 | 3634,39 | 6016,47 | 5440,64 | 4467,28 | 9125,14 | 3705,27 | 6998,09 | 5587 |
| PE O-36:3|PE O-18:1_18:2 | 317,76 | 235,72 | 954,03 | 546,36 | 785,15 | 417,21 | 999,65 | 951,69 | 651 |
| PE O-36:3|PE O-18:2_18:1 | 5883,33 | 4547,53 | 5941,91 | 6728,5 | 6629,18 | 8908,09 | 5917,21 | 6934,13 | 6436 |
| PE O-36:4|PE O-18:2_18:2 | 2931,78 | 2141,41 | 2394,78 | 2993,61 | 3390,74 | 5300,34 | 2458,84 | 3198,2 | 3101 |
| PE O-36:5|PE O-16:1_20:4 | 1687,06 | 1202,51 | 2710,95 | 2928,87 | 3032,71 | 4627,79 | 1847,43 | 4148,37 | 2773 |
| PE O-36:5|PE O-18:2_18:3 | 558,89 | 406,95 | 407,42 | 743,18 | 671,64 | 1055,95 | 551,19 | 569,57 | 621 |
| PE O-36:6|PE O-16:1_20:5 | 875,39 | 649,62 | 1147,54 | 1322,8 | 1213,35 | 2065,73 | 687,86 | 983,57 | 1118 |
| PE O-38:4|PE O-18:1_20:3 | 267,79 | 210,2 | 595,8 | 598,35 | 626,41 | 729,95 | 500,2 | 818,1 | 543 |
| PE O-38:5|PE O-18:1_20:4 | 692,44 | 506,8 | 1626,37 | 1120,58 | 1046,87 | 1967,07 | 1053,44 | 1598,12 | 1201 |
| PE O-38:6|PE O-16:1_22:5 | 1820,24 | 1156,49 | 3470,6 | 2926,18 | 3484,68 | 4186,79 | 2056,46 | 4287,82 | 2924 |
| PE O-40:6|PE O-18:1_22:5 | 448,04 | 374,86 | 1484,34 | 850,32 | 1082,21 | 1618,79 | 1178,18 | 1656,63 | 1087 |
| PE P-30:1|PE P-14:0_16:1 | 183,97 | 181,21 | 249,18 | 1442,5 | 1222,11 | 640,99 | 269,01 | 482,18 | 584 |
| PE P-31:0|PE P-15:0_16:0 | 552,65 | 425,71 | 438,15 | 3127,01 | 1528,23 | 1165,6 | 366,85 | 896,63 | 1063 |
| PE P-32:1|PE P-14:0_18:1 | 930,35 | 637,68 | 1028,54 | 6752,81 | 4472,48 | 2850,9 | 1152,56 | 1778,5 | 2450 |
| PE P-32:2|PE P-14:0_18:2 | 502,84 | 339,86 | 587,35 | 4108,74 | 1343,48 | 854,94 | 709,99 | 1036,92 | 1186 |
| PE P-33:1|PE P-15:0_18:1 | 1148,17 | 991,18 | 1314,13 | 7358,4 | 3279,4 | 1532,1 | 1087,18 | 2573,38 | 2410 |
| PE P-33:2|PE P-15:0_18:2 | 697,65 | 701,34 | 817,01 | 2654,07 | 1523,25 | 1122,78 | 995,67 | 1051,38 | 1195 |
| PE P-33:3|PE P-15:0_18:3 | 395,19 | 422,09 | 539,68 | 1650,09 | 1487,08 | 732,1 | 767,16 | 733,39 | 841 |
| PE P-34:1|PE P-16:0_18:1 | 3291,58 | 2840,97 | 7263,35 | 19868,12 | 14827,26 | 11309,1 | 6757,07 | 10736,85 | 9612 |
| PE P-34:2|PE P-16:0_18:2 | 1964,44 | 1664,29 | 2383,63 | 7334,12 | 4768,44 | 3822,11 | 3278,59 | 3767,3 | 3623 |
| PE P-34:3|PE P-16:0_18:3 | 591,76 | 494,76 | 660,66 | 2735,69 | 1637,15 | 1042,02 | 1098,76 | 914,94 | 1147 |
| PE P-36:1|PE P-18:0_18:1 | 3035,32 | 1992,03 | 3112,4 | 13679,15 | 12413,56 | 9344,01 | 4650,87 | 6368,68 | 6825 |
| PE P-36:2|PE P-18:0_18:2 | 1321,67 | 1205,67 | 1644,73 | 5921,08 | 4780,41 | 3474,74 | 1855,45 | 2418,94 | 2828 |
| PE P-36:2|PE P-18:1_18:1 | 1177,95 | 927,46 | 1260,06 | 6229,37 | 3680,7 | 2395,63 | 1804,95 | 2463,14 | 2492 |
| PE P-36:3|PE P-16:0_20:3 | 699,12 | 521,34 | 762,97 | 2682,29 | 2352,07 | 1301,1 | 959,71 | 1175,09 | 1307 |
| PE P-36:3|PE P-18:1_18:2 | 666,6 | 599,89 | 794,88 | 2543,71 | 1917,13 | 1095,71 | 705,64 | 991,72 | 1164 |
| PE P-36:4|PE P-16:0_20:4 | 412,93 | 368,32 | 672,1 | 2588,23 | 1551,02 | 1179,77 | 600,66 | 1354,91 | 1091 |
|  |  |  |  |  |  |  |  |  |  |
| **Phosphatidylinositols** |  |  |  |  |  |  |  |  |  |
|  |  |  |  |  |  |  |  |  |  |

| PI 30:0 | 406,45 | 797,36 | 779,68 | 1842,7 | 2333,99 | 888,63 | 2082,69 | 992,64 | 1266 |
| --- | --- | --- | --- | --- | --- | --- | --- | --- | --- |
| PI 30:0|PI 14:0_16:0 | 421,99 | 850,08 | 824,9 | 1893,97 | 2444,18 | 918,45 | 2124,44 | 1022,15 | 1313 |
| PI 32:0|PI 16:0_16:0 | 2624,05 | 4826,59 | 5291,45 | 12117,44 | 20190,91 | 6524,55 | 7087,6 | 6452,83 | 8139 |
| PI 32:1|PI 14:0_18:1 | 1952,99 | 3021,15 | 3532,8 | 6935,93 | 11268,82 | 4477,54 | 5152,41 | 3570,75 | 4989 |
| PI 33:1 | 351,36 | 722,46 | 811,91 | 1590,2 | 2050,4 | 989,9 | 1234,4 | 1080,19 | 1104 |
| PI 34:0|PI 16:0_18:0 | 3409,29 | 4238,66 | 5027,71 | 9782,62 | 17846,98 | 7325,57 | 8769,15 | 10895,92 | 8412 |
| PI 34:1|PI 16:0_18:1 | 23937,13 | 29062,8 | 36108,08 | 59669,17 | 91262,71 | 43903,5 | 43713,1 | 39878,15 | 45942 |
| PI 34:2|PI 16:1_18:1 | 5360,41 | 6723,8 | 7637,69 | 15068,73 | 22571,01 | 9909,11 | 10433 | 8473,86 | 10772 |
| PI 34:3 | 257,21 | 526,29 | 484,54 | 1255,76 | 1465,28 | 415,84 | 922,47 | 415,57 | 718 |
| PI 35:2 | 653,55 | 809,14 | 1017,54 | 1221,6 | 1936,88 | 928,79 | 968,47 | 967,92 | 1063 |
| PI 36:0 | 684,76 | 789,36 | 1217,95 | 2911,23 | 4172,05 | 1275,91 | 2627,51 | 1791,73 | 1934 |
| PI 36:1|PI 18:0_18:1 | 75693,78 | 87294,4 | 102112,55 | 143440,25 | 239416,4 | 137225 | 199948 | 181603,55 | 145842 |
| PI 36:2|PI 18:1_18:1 | 49992,87 | 57549 | 54229,72 | 80273,39 | 132234,15 | 73577,9 | 100951 | 81163,37 | 78746 |
| PI 36:3|PI 18:1_18:2 | 10297,93 | 12536,8 | 11256,38 | 20140,69 | 31819,97 | 16872,6 | 21841,7 | 14421,22 | 17398 |
| PI 36:4|PI 16:0_20:4 | 2071,16 | 3020,37 | 3756,27 | 4509,42 | 6790,39 | 3251,84 | 3181,88 | 5659,86 | 4030 |
| PI 36:4|PI 18:2_18:2 | 585,31 | 1096,54 | 1064,22 | 1963,76 | 2709,41 | 1446,46 | 2084,87 | 1185,86 | 1517 |
| PI 36:5 | 955,28 | 854,32 | 1165,88 | 1682,49 | 1446,41 | 1585,26 | 1095,54 | 1537,11 | 1290 |
| PI 37:1 | 534,4 | 905,96 | 1432,2 | 2064,98 | 3013,49 | 1481,96 | 2875,25 | 2191,51 | 1812 |
| PI 37:2 | 767,69 | 1013,77 | 1461,3 | 1504,59 | 1900,17 | 1896,02 | 1846,31 | 1964,62 | 1544 |
| PI 38:1 | 878,28 | 1124,17 | 2172,87 | 2595,78 | 3293,47 | 1908,48 | 3572,49 | 2365,56 | 2239 |
| PI 38:2 | 2680,74 | 3252,04 | 4516,89 | 5078,25 | 8129,59 | 6162,12 | 7631,98 | 6511,71 | 5495 |
| PI 38:3|PI 18:0_20:3 | 11503,26 | 11532,1 | 13260,73 | 11310,06 | 17585,05 | 19338,3 | 20322,2 | 27245,72 | 16512 |
| PI 38:4|PI 18:0_20:4 | 15456,12 | 16127,1 | 18869,66 | 16429,75 | 30155,55 | 22724 | 20828,4 | 41066,34 | 22707 |
| PI 38:4|PI 18:1_20:3 | 3128,87 | 4036,18 | 5137,75 | 4716,67 | 8493,96 | 8390,01 | 4960,1 | 9166,04 | 6004 |
| PI 38:5|PI 18:0_20:5 | 7179,53 | 8566,21 | 7693,8 | 9038,12 | 13590,99 | 12067,6 | 9832,64 | 15761,14 | 10466 |
| PI 38:5|PI 18:1_20:4 | 5384,36 | 5878,7 | 7568,43 | 8516,91 | 11342,12 | 9992,9 | 6270,32 | 15538,54 | 8812 |
| PI 38:6 | 1038,5 | 1610,28 | 1514,23 | 1931,43 | 2517,46 | 2380,49 | 1403,22 | 2256,12 | 1831 |
| PI 40:3 | 3190,25 | 3451,93 | 4423,65 | 4187,99 | 6125,32 | 8682,3 | 3328,61 | 4744,28 | 4767 |
| PI 40:6 | 142,61 | 184,61 | 636,64 | 624,07 | 689,63 | 475,42 | 492,54 | 570,93 | 477 |
| PI 42:10 | 2842,78 | 2607,72 | 2173,74 | 3305,87 | 6661,31 | 3700,04 | 4267,13 | 3845,15 | 3675 |
| **Phosphatidylserines** |  |  |  |  |  |  |  |  |  |
| PS 32:0 | 331,72 | 380,36 | 275,57 | 1808,28 | 1270,95 | 731,72 | 360,49 | 623,93 | 723 |
| PS 32:1|PS 16:0_16:1 | 432,77 | 692,3 | 1102,2 | 4260,1 | 5346,34 | 743,99 | 500,31 | 588,96 | 1708 |
| PS 34:1|PS 16:0_18:1 | 13368,61 | 16268,3 | 17678,34 | 31357,42 | 54923,14 | 21636,9 | 10547,1 | 15162,88 | 22618 |
| PS 34:2|PS 16:0_18:2 | 5138,56 | 6317,7 | 6923,73 | 15269,56 | 21599,4 | 6774,58 | 4830,9 | 5148,43 | 9000 |
| PS 34:3|PS 16:0_18:3 | 563,49 | 690,81 | 669,04 | 2118,31 | 2803,64 | 407,02 | 550 | 271,15 | 1009 |
| PS 35:1|PS 18:0_17:1 | 3169,64 | 3869,77 | 5758,61 | 5678,55 | 8400,13 | 5994,31 | 4774,91 | 8132,59 | 5722 |
| PS 35:2|PS 17:0_18:2 | 287,33 | 305,3 | 420,45 | 3346,24 | 1524,01 | 794,18 | 406,68 | 517,15 | 950 |
| PS 36:0|PS 18:0_18:0 | 21654,6 | 20520,1 | 29872,45 | 27033,44 | 42585,62 | 33758,5 | 26358 | 42732,53 | 30564 |
| PS 36:1|PS 18:0_18:1 | 132548,8 | 136232 | 202332 | 164574,6 | 279236,9 | 228835 | 200620 | 340721,3 | 210638 |
| PS 36:2|PS 18:1_18:1 | 89959 | 98146 | 112769,68 | 111812 | 181069,55 | 130021 | 139869 | 165281,5 | 128616 |
| PS 36:3|PS 18:0_18:3 | 2930 | 2639 | 3540,25 | 5997,66 | 10165,1 | 6247,61 | 6094,46 | 6193,24 | 5476 |
| PS 36:3|PS 18:1_18:2 | 17967 | 21393 | 20225,01 | 27700,4 | 43828,5 | 24555,2 | 31837,2 | 24568,45 | 26509 |
| PS 36:4|PS 18:1_18:3 | 1887 | 2316 | 2008,76 | 3646,84 | 3816,01 | 2024,33 | 3231,55 | 1926,96 | 2607 |
| PS 36:4|PS 18:2_18:2 | 347 | 207 | 235,19 | 1416,14 | 763,52 | 447,14 | 444,94 | 360,81 | 528 |

| PS 37:0 | 70,2 | 56,1 | 95,14 | 944,08 | 247,82 | 140,38 | 99,13 | 117,05 | 221 |
| --- | --- | --- | --- | --- | --- | --- | --- | --- | --- |
| PS 37:2 | 161,4 | 156,41 | 222,65 | 1130,78 | 464,37 | 351,25 | 286,67 | 292,71 | 383 |
| PS 38:1|PS 20:0_18:1 | 3958,29 | 5354,19 | 9286,59 | 6014,88 | 8724,42 | 7461,43 | 7127,95 | 12838,86 | 7596 |
| PS 38:2|PS 18:1_20:1 | 3324,73 | 3805,7 | 5244,21 | 4201,96 | 7060,57 | 5624 | 4381,97 | 5993,12 | 4955 |
| PS 38:3 | 12918,47 | 14790 | 33736,1 | 70513,97 | 49667,78 | 23814,3 | 30886,9 | 26302,52 | 32829 |
| PS 38:3|PS 18:0_20:3 | 9614 | 10167 | 14989 | 16414 | 22859,12 | 21515 | 18127,7 | 25940,37 | 17453 |
| PS 38:4|PS 18:1_20:3 | 1943 | 2219 | 2321 | 2987 | 4102,3 | 4460,02 | 2650,18 | 3392,76 | 3009 |
| PS 38:5|PS 16:0_22:5 | 1069 | 1182 | 2316 | 4463 | 5604,7 | 1792,03 | 1222,63 | 1949,44 | 2450 |
| PS 39:1|PS 21:0_18:1 | 3405 | 3274 | 5855 | 4177 | 5920,95 | 5770,74 | 4868,09 | 8437,85 | 5214 |
| PS 39:2|PS 18:1_21:1 | 2465 | 2884 | 4093 | 3629 | 5673,49 | 4276,03 | 2667,6 | 3730,07 | 3677 |
| PS 40:1|PS 22:0_18:1 | 4163 | 5025 | 8804 | 4661 | 8097,95 | 9298,85 | 6252,53 | 12448,56 | 7344 |
| PS 40:2 | 2141 | 2877 | 3496 | 3046 | 3754,44 | 4363,69 | 3212,62 | 4329,97 | 3403 |
| PS 40:2|PS 22:0_18:2 | 2399 | 3038 | 3838 | 2913 | 3526,08 | 4170,04 | 3159,03 | 3759,92 | 3351 |
| PS 40:3 | 804 | 1043 | 2306 | 5181 | 3083,12 | 1464,02 | 2733,46 | 2386,31 | 2375 |
| PS 40:4|PS 18:0_22:4 | 2196 | 1940 | 4743 | 5421 | 8833,08 | 3308,1 | 5250,06 | 10730,13 | 5303 |
| PS 40:5|PS 18:0_22:5 | 23024 | 21238 | 32016 | 35837 | 51623,66 | 38220,7 | 41627,8 | 71835,24 | 39428 |
| PS 40:6|PS 18:1_22:5 | 3655 | 3980 | 5045 | 7027 | 9336,32 | 5712,89 | 6728,91 | 9188,12 | 6334 |
| PS 41:2 | 701 | 1042 | 1718 | 1621 | 1802,84 | 1674,62 | 903,25 | 1921,78 | 1423 |
| PS 42:1|PS 24:0_18:1 | 600 | 720 | 1277 | 1079 | 1416,18 | 1554,55 | 1112,71 | 2240,62 | 1250 |
| **Sulfatides** |  |  |  |  |  |  |  |  |  |
| SHexCer 35:0;3O | 2374 | 2867 | 2443 | 2404 | 4574,64 | 3826,24 | 2307,57 | 3178,19 | 2997 |
| SHexCer 36:4;2O | 3213 | 2236 | 1792 | 2256 | 5432,67 | 5267,05 | 1924,53 | 3487 | 3201 |
| SHexCer 38:2;2O | 189 | 214 | 270 | 486 | 369,44 | 409,43 | 680,61 | 332,9 | 369 |
| SHexCer 38:7;3O | 288 | 436 | 354 | 419 | 741,12 | 1075,7 | 496,97 | 703,79 | 564 |
| SHexCer 39:5;3O | 1274 | 1332 | 1335 | 966 | 2851,35 | 2417,48 | 1412,37 | 2837,45 | 1803 |
| SHexCer 39:6;3O | 423 | 721 | 697 | 636 | 1337 | 855,37 | 882,81 | 1264,66 | 852 |
| SHexCer 39:7;3O | 6225 | 4828 | 4303 | 6594 | 11636,5 | 9804,81 | 3772,54 | 7253,79 | 6802 |
| SHexCer 39:8;3O | 2371 | 1700 | 1745 | 3065 | 5144,02 | 3565,56 | 1744,81 | 2383,24 | 2715 |
| SHexCer 40:2;2O | 2455 | 2552 | 2813 | 7788 | 11760,16 | 4889,55 | 5517,44 | 3717,49 | 5187 |
| SHexCer 40:3;3O | 1776 | 1063 | 1279 | 1688 | 2583,7 | 3765,66 | 1985,16 | 2284,03 | 2053 |
| SHexCer 40:8;3O | 263 | 388 | 301 | 268 | 696,68 | 455,73 | 486,88 | 770,26 | 454 |
| SHexCer 40:9;3O | 1265 | 984 | 887 | 658 | 1660,69 | 1544,24 | 962,39 | 1994,79 | 1245 |
| SHexCer 41:8;3O | 18458 | 12152 | 10085 | 10653 | 23060,86 | 22509,6 | 8745,68 | 12827,61 | 14811 |
| SHexCer 42:2;2O | 119 | 123 | 136 | 494 | 575,97 | 124,85 | 336,35 | 346,49 | 282 |
| SHexCer 42:4;3O | 2948 | 2132 | 3648 | 3775 | 5533,62 | 6415,03 | 4786,13 | 6555,85 | 4474 |
| SHexCer 43:0;3O | 379 | 423 | 2060 | 4105 | 2536,77 | 622,67 | 1741,89 | 1420,64 | 1661 |
| SHexCer 44:2;2O | 1372 | 1455 | 1164 | 2884 | 3070,67 | 2554,72 | 3639,67 | 2003,66 | 2268 |
| SHexCer 46:2;2O | 5087 | 4896 | 5592 | 13506 | 16735,72 | 8680,39 | 10433 | 7846,23 | 9097 |
| **Sphingomyelins** |  |  |  |  |  |  |  |  |  |
| SM 30:0;2O|SM 22:0;2O/8:0 | 840 | 718 | 754 | 1287 | 1033,54 | 914,51 | 1091,35 | 1729,29 | 1046 |
| SM 30:1;2O|SM 16:1;2O/14:0 | 1019 | 1020 | 921 | 2779 | 1384,73 | 1892,32 | 1206,35 | 1842,25 | 1508 |
| SM 31:0;2O|SM 19:0;2O/12:0 | 200 | 210 | 231 | 473 | 446,88 | 308,67 | 307,01 | 429,12 | 326 |
| SM 31:1;2O|SM 17:1;2O/14:0 | 753 | 632 | 731 | 3658 | 1315,56 | 1226,97 | 984,46 | 1445,5 | 1343 |
| SM 32:0;2O | 634 | 616 | 728 | 2095 | 1340,92 | 1112,54 | 801,07 | 1177,72 | 1063 |
| SM 32:0;2O|SM 22:0;2O/10:0 | 18493 | 18107 | 30173 | 33401 | 37795,13 | 31738,3 | 31809,6 | 46974,22 | 31061 |
| SM 32:1;2O | 488 | 406 | 425 | 521 | 725,66 | 688,84 | 364,8 | 739,17 | 545 |

| SM 32:1;2O|SM 16:1;2O/16:0 | 41287 | 38511 | 60509 | 62604 | 72388,31 | 71680 | 60345,1 | 99472,1 | 63349 |
| --- | --- | --- | --- | --- | --- | --- | --- | --- | --- |
| SM 33:0;2O | 2299 | 2307 | 4058 | 7560 | 6263,96 | 4298,92 | 4059,27 | 5856,09 | 4588 |
| SM 33:1;2O | 542 | 518 | 770 | 1442 | 1383,03 | 1113,89 | 559,38 | 1169,18 | 937 |
| SM 33:1;2O|SM 17:1;2O/16:0 | 19582 | 19627 | 39217 | 45600 | 51655,83 | 39745,5 | 33044,6 | 55716,01 | 38024 |
| SM 34:0;2O | 19541 | 20022 | 31550 | 66840 | 74917,5 | 41919,4 | 22710 | 39238,71 | 39592 |
| SM 34:1;2O | 6964 | 12171 | 26648 | 12103 | 23856,55 | 16623,2 | 17688 | 29924,45 | 18247 |
| SM 34:1;2O|SM 18:1;2O/16:0 | 237401 | 247280 | 428615 | 518165 | 790900,4 | 514146 | 390865 | 639405,05 | 470847 |
| SM 34:1;3O | 586 | 548 | 844 | 2097 | 1422,96 | 1118,81 | 585,26 | 1330,71 | 1066 |
| SM 34:2;2O|SM 25:2;2O/9:0 | 1992 | 1958 | 3692 | 9352 | 5973,63 | 4104,93 | 4194,27 | 5224,85 | 4561 |
| SM 35:0;2O | 426 | 416 | 690 | 957 | 1176,92 | 948,67 | 714,77 | 1536,02 | 858 |
| SM 35:1;2O|SM 16:1;2O/19:0 | 4122 | 4572 | 9411 | 10313 | 10453,99 | 9699,35 | 5672,38 | 18019,68 | 9033 |
| SM 35:1;2O|SM 18:1;2O/17:0 | 2870 | 2748 | 3664 | 7739 | 5650,44 | 5252,4 | 3470,79 | 7059,64 | 4807 |
| SM 35:2;2O | 430 | 342 | 524 | 2838 | 1027,35 | 764,93 | 393,4 | 1083,35 | 925 |
| SM 35:2;2O|SM 21:1;2O/14:1 | 332 | 234 | 311 | 2809 | 829,61 | 563,72 | 285,29 | 527,1 | 737 |
| SM 35:2;3O | 721 | 543 | 867 | 4447 | 2282,12 | 1848,52 | 1086,32 | 1718,77 | 1689 |
| SM 36:0;2O | 1355 | 1226 | 1022 | 4600 | 3567,13 | 1748,81 | 984,32 | 2470,08 | 2122 |
| SM 36:1;2O|SM 15:0;2O/21:1 | 6746 | 6842 | 13827 | 15259 | 17988,15 | 11215,1 | 14498,4 | 24115,86 | 13811 |
| SM 36:2;2O|SM 19:1;2O/17:1 | 1388 | 1379 | 3456 | 4725 | 5265,07 | 3088,28 | 3800,53 | 5627,64 | 3591 |
| SM 37:0;2O | 1044 | 949 | 1114 | 2264 | 2124,08 | 1633,95 | 1461,06 | 1996,56 | 1573 |
| SM 37:1;2O | 551 | 578 | 644 | 1376 | 981,95 | 1018,62 | 764,41 | 1611,95 | 941 |
| SM 38:0;2O|SM 16:0;2O/22:0 | 13194 | 12725 | 9906 | 21324 | 14680,77 | 15213,7 | 8904,73 | 16795,76 | 14093 |
| SM 38:1;2O|SM 16:1;2O/22:0 | 76342 | 78275 | 97900 | 66399 | 92615,23 | 98366,6 | 87129 | 146105,65 | 92891 |
| SM 38:2;2O|SM 19:2;2O/19:0 | 631 | 638 | 692 | 2150 | 1447,25 | 1047,6 | 687,23 | 1011,98 | 1038 |
| SM 39:0;2O | 27995 | 31061 | 28380 | 37638 | 31409,52 | 36018,6 | 26054,2 | 48362,68 | 33365 |
| SM 39:1;2O|SM 17:1;2O/22:0 | 1233 | 798 | 1843 | 5077 | 2911,72 | 2186,46 | 1860,5 | 2708,01 | 2327 |
| SM 39:1;3O | 7110 | 4443 | 5357 | 10104 | 7923,03 | 5727,59 | 3163,77 | 7302,89 | 6391 |
| SM 39:2;2O|SM 19:1;2O/20:1 | 1120 | 1275 | 1163 | 4813 | 2261,96 | 1915,99 | 1032,85 | 1567,75 | 1894 |
| SM 40:0;2O | 18274 | 12996 | 7878 | 28909 | 12732,12 | 15524,4 | 5878,53 | 13455,43 | 14456 |
| SM 40:1;2O|SM 16:1;2O/24:0 | 201288 | 182136 | 196616 | 213577 | 246985,5 | 240010 | 132476 | 305625,25 | 214839 |
| SM 40:1;2O|SM 18:1;2O/22:0 | 2537 | 2229 | 1986 | 5230 | 4161,62 | 2915,35 | 1913,44 | 2730,82 | 2963 |
| SM 40:1;3O | 1356 | 1385 | 883 | 2328 | 1875,87 | 1670,26 | 502,76 | 1366,1 | 1421 |
| SM 40:2;2O|SM 18:1;2O/22:1 | 10272 | 10317 | 14018 | 15617 | 13649,89 | 12807,8 | 12474,1 | 17359,07 | 13314 |
| SM 40:8;2O | 633 | 304 | 415 | 4050 | 1907,58 | 1407,37 | 772,7 | 510,97 | 1250 |
| SM 41:0;2O | 5024 | 4728 | 2719 | 13917 | 6295,31 | 5338,43 | 2681,19 | 4285,97 | 5624 |
| SM 41:1;2O|SM 18:1;2O/23:0 | 37067 | 38524 | 23093 | 114773 | 46248,69 | 47696,2 | 14128,1 | 49342,07 | 46359 |
| SM 41:2;2O|SM 18:1;2O/23:1 | 5012 | 4814 | 4716 | 13702 | 9103,39 | 7398,82 | 3977,38 | 5066,76 | 6724 |
| SM 42:1;2O|SM 18:1;2O/24:0 | 77145 | 74539 | 46555 | 117815 | 55568,77 | 80497,3 | 27127,8 | 66319,17 | 68196 |
| SM 42:2;2O | 397 | 337 | 376 | 1331 | 462,32 | 408,4 | 221,01 | 462,57 | 499 |
| SM 42:2;2O|SM 18:1;2O/24:1 | 11147 | 10278 | 13887 | 28950 | 22472,1 | 14319 | 8571,7 | 18355,09 | 15998 |
| SM 42:2;3O | 159 | 147 | 255 | 580 | 323,47 | 316,56 | 296,78 | 406,62 | 310 |
| SM 42:3;2O | 532 | 422 | 552 | 1809 | 1309,01 | 882,19 | 738,56 | 631,66 | 859 |
| SM 43:2;2O|SM 18:1;2O/25:1 | 1733 | 1601 | 700 | 6092 | 1162,85 | 2029,08 | 580,04 | 1022,43 | 1865 |
| SM 44:8;2O | 406 | 312 | 66 | 5205 | 912,24 | 232,17 | 50,05 | 91,29 | 909 |
| **Triacylglycerols** |  |  |  |  |  |  |  |  |  |
| TG 10:0_10:0_20:5 | 33388 | 20657 | 19889 | 105422 | 189918,85 | 138477 | 34969,8 | 43369,06 | 73261 |
| TG 46:0|TG 14:0_16:0_16:0 | 9195 | 10989 | 15432 | 140149 | 23191,54 | 31803 | 13442,1 | 16219,6 | 32553 |

| TG 48:1|TG 16:0_16:0_16:1 | 35098 | 48201 | 67230 | 464438 | 119142,9 | 111114 | 57663,4 | 109093,04 | 126498 |
| --- | --- | --- | --- | --- | --- | --- | --- | --- | --- |
| TG 49:3|TG 15:0_16:0_18:3 | 6072 | 9052 | 45574 | 34917 | 94342,88 | 36574,1 | 22539,2 | 43878,91 | 36619 |
| TG 50:2|TG 16:0_16:0_18:2 | 21676 | 47951 | 71804 | 366479 | 150902,1 | 109277 | 57702,3 | 162207,9 | 123500 |
| TG 51:2|TG 15:0_18:1_18:1 | 5334 | 5680 | 12592 | 50193 | 30968,52 | 25831,4 | 9982,76 | 35164,79 | 21968 |
| TG 51:4|TG 15:0_16:0_20:4 | 4776 | 6563 | 22647 | 23121 | 83586,22 | 19184,5 | 17025,1 | 34022,11 | 26366 |
| TG 52:2|TG 16:0_18:1_18:1 | 24960 | 39341 | 39160 | 416460 | 243517,1 | 154685 | 40677,3 | 144462,81 | 137908 |
| TG 52:3|TG 16:0_18:1_18:2 | 528578 | 673720 | 1258496 | 3279022 | 4282306 | 2624927 | 980968 | 2829452,5 | 2057184 |
| TG 53:4|TG 17:1_18:1_18:2 | 4088 | 8034 | 17204 | 28040 | 52192,47 | 16989 | 21432 | 48997,63 | 24622 |
| TG 54:6|TG 18:1_18:2_18:3 | 7756 | 15981 | 30303 | 76034 | 134667,42 | 55994,1 | 37015,2 | 65266,56 | 52877 |
| TG 56:6|TG 16:0_18:1_22:5 | 4523 | 9285 | 33653 | 41405 | 93490,46 | 45359,9 | 33649,4 | 128703,2 | 48759 |
| TG 57:6|TG 17:0_18:1_22:5 | 329 | 379 | 1260 | 1696 | 3337,95 | 2245,08 | 1325,85 | 4587,81 | 1895 |
| TG 8:0_8:0_16:4 | 768 | 459 | 470 | 3389 | 5027,07 | 2340,49 | 1224,92 | 858,24 | 1817 |
| TG O-42:5|TG O-20:5_11:0_11:0 | 1139 | 1401 | 1002 | 62418 | 2766,98 | 2387,74 | 591 | 2716,53 | 9303 |
| TG O-49:6|TG O-15:4_17:1_17:1 | 1690 | 2537 | 2819 | 37153 | 4952,23 | 6099,23 | 2128,16 | 3940,63 | 7665 |
| TG O-51:6|TG O-15:4_17:0_19:2 | 821 | 1698 | 1717 | 25420 | 2672,88 | 3129,36 | 1192,56 | 2402,3 | 4882 |
| TG O-51:7|TG O-15:4_15:0_21:3 | 5588 | 6223 | 7116 | 94226 | 14544,37 | 12200,4 | 7121,33 | 11411,32 | 19804 |
| TG O-52:7|TG O-18:4_16:0_18:3 | 885 | 1030 | 1729 | 11346 | 3254,77 | 2922,45 | 1056,08 | 2961,63 | 3148 |
| TG O-54:8|TG O-15:4_18:1_21:3 | 903 | 919 | 1425 | 11070 | 3594,63 | 3323,72 | 1247,64 | 4187,25 | 3334 |
| TG O-55:9|TG O-13:1_21:4_21:4 | 2148 | 2605 | 3164 | 43113 | 8151,42 | 8049,16 | 2445,92 | 9184,27 | 9858 |
| TG O-57:10|TG O-15:0_21:5_21:5 | 1270 | 1685 | 1382 | 17710 | 4509,76 | 3605,23 | 1782,46 | 5344 | 4661 |
| TG O-57:9|TG O-15:1_21:4_21:4 | 1142 | 1670 | 1696 | 16918 | 6909,91 | 4219,07 | 2412,98 | 7036,46 | 5250 |
| **Others** |  |  |  |  |  |  |  |  |  |
| CoQ8 | 804 | 739 | 895 | 2729 | 2372,34 | 1141,95 | 645,92 | 1089,15 | 1302 |
| DGCC 17:2_22:6 | 1169 | 1089 | 1595 | 4612 | 2037,57 | 2082,08 | 2033,36 | 3443,74 | 2258 |
| DGGA 36:2|DGGA 18:1_18:1 | 2228,59 | 1540,18 | 1198,13 | 1821,75 | 3854,45 | 3433,03 | 1474,24 | 1940,24 | 2186 |
| DGTS 21:0 | 292,9 | 129,89 | 138,66 | 4635,68 | 1222,29 | 940,12 | 345,85 | 345,3 | 1006 |
| LPA 16:3 | 1866,82 | 1232,8 | 1342,01 | 1554,77 | 2714,22 | 2399,48 | 1145,31 | 1197,94 | 1682 |
| LPE O-19:4 | 720 | 417,97 | 264,67 | 22053,64 | 2079,25 | 1450,93 | 443,29 | 812 | 3530 |
| LPE-N (FA)33:2|LPE-N (FA 15:0)18:2 | 705,72 | 506,01 | 600,67 | 1646,83 | 1392,41 | 1055,26 | 790,07 | 645,39 | 918 |
| LPG O-13:0 | 554,62 | 392,59 | 440,68 | 494,88 | 952,73 | 766,54 | 448 | 577,27 | 578 |
| NAGly 9:0;O | 1166,04 | 762,55 | 623,43 | 982,88 | 3143,33 | 1148 | 344,91 | 1004,68 | 1147 |
| Pentaerythritol tetrakis(3,5-di-tert-butyl-4- |  |  |  |  |  |  |  |  |  |
| hydroxyhydrocinnamate) | 7491,94 | 6770,31 | 7423,67 | 5680,28 | 12302,83 | 15571,3 | 3535,6 | 4904,85 | 7960 |
| PI O-16:0 | 1620,64 | 4677,53 | 2865,62 | 3700,77 | 5204,65 | 1313,86 | 5060,01 | 2792,04 | 3404 |
| PI-Cer 38:3;2O | 385,52 | 352,79 | 478,39 | 1065,82 | 1309,42 | 977,4 | 578,24 | 1083,64 | 779 |
| PMeOH 40:4|PMeOH 20:2_20:2 | 2,01 | 11,03 | 320,58 | 700,67 | 1811,05 | 522,53 | 534,77 | 447,33 | 544 |
| ST 27:1;O;S - Cholesterol sulfate | 1187 | 1534 | 3396 | 1255 | 1800,19 | 1323,21 | 1163,33 | 2141,28 | 1725 |
| SL 12:0;O/26:0 | 3434 | 3514 | 4716 | 13676 | 10116,08 | 6794,55 | 4724,44 | 6397,9 | 6672 |
| SL 12:1;O/32:0 | 2375 | 6265 | 5737 | 11560 | 9192,57 | 9922,05 | 4135,91 | 6471,62 | 6958 |
| MG 40:1;2O | 4177 | 4440 | 4280 | 7108 | 3260,13 | 3561,84 | 2149,35 | 5401,4 | 4297 |
| **EO Treated T8** |  |  |  |  |  |  |  |  |  |
| **Metabolyte name** | **t8_T1** | **t8_T2** | **t8_T3** | **t8_T4** | **t8_T5** | **t8_T6** | **t8_T7** | **t8_T8** | **average value** |
| **Acyl sterol glycosides** |  |  |  |  |  |  |  |  |  |

| ASG 27:1;O;Hex;FA 2:0 | 628,03 | 367,07 | 639,77 | 687,69 | 869,55 | 718,81 | 554,69 | 405,75 | 608,92 |
| --- | --- | --- | --- | --- | --- | --- | --- | --- | --- |
| ASG 28:1;O;Hex;FA 14:0 | 932,84 | 449,62 | 276,02 | 881,81 | 337,89 | 1620,69 | 457,01 | 143,18 | 637,3825 |
| ASG 28:1;O;Hex;FA 16:0 | 511,56 | 207,86 | 446,81 | 1203,34 | 460,64 | 1175,52 | 443,93 | 64 | 564,2075 |
| ASG 28:1;O;Hex;FA 18:0 | 432,56 | 43,62 | 115,93 | 752,81 | 428,58 | 1070,39 | 252,84 | 68,25 | 395,6225 |
| ASG 28:1;O;Hex;FA 20:1 | 528,41 | 142,28 | 394,44 | 1153,72 | 374,68 | 1066,91 | 277,99 | 162,3 | 512,59125 |
| ASG 28:2;O;Hex;FA 14:0 | 1338,01 | 181 | 324,9 | 2280,12 | 917,26 | 2787,26 | 794,9 | 165,31 | 1098,595 |
| ASG 28:2;O;Hex;FA 16:0 | 866,52 | 276,11 | 416,01 | 1224,81 | 450,99 | 1213,94 | 428,95 | 102,38 | 622,46375 |
| ASG 28:2;O;Hex;FA 16:1 | 427,05 | 95,81 | 205,98 | 528,07 | 269,28 | 840,32 | 241,62 | 2,04 | 326,27125 |
| ASG 28:2;O;Hex;FA 20:1 | 267,39 | 80,35 | 115,31 | 548,22 | 159,58 | 556,23 | 191,13 | 61,43 | 247,455 |
| ASG 29:2;O;Hex;FA 20:5 | 896,05 | 1061,8 | 1718,85 | 1040,75 | 1170,25 | 1574,29 | 442,98 | 1091,37 | 1124,5425 |
| **Carnitines** |  |  |  |  |  |  |  |  |  |
| CAR 13:0 | 664,94 | 1858,59 | 609,39 | 1121,71 | 814,18 | 1409,87 | 983,31 | 680,8 | 1017,84875 |
| CAR 21:2 | 3330,66 | 3639,76 | 2131,88 | 4239,4 | 3576,83 | 3849,01 | 3021,96 | 1529,08 | 3164,8225 |
| CAR 21:4 | 2287,29 | 2842,89 | 289,93 | 1154,94 | 6913,83 | 1311,86 | 926,6 | 1057,52 | 2098,1075 |
| CAR 4:0 | 3006,05 | 13324,6 | 5198,74 | 3638,71 | 18720,1 | 2617,03 | 4816,55 | 12754,24 | 8009,49875 |
| CAR 5:0 | 1654,04 | 6866,49 | 4439,11 | 5350,78 | 32118,81 | 3507,69 | 6796,23 | 11502,41 | 9029,445 |
| **Ceramides** |  |  |  |  |  |  |  |  |  |
| Cer 12:0;2O/25:0 | 2760,07 | 4737,44 | 5049,14 | 6801,62 | 4652,36 | 2855,05 | 3965,58 | 2751,22 | 4196,56 |
| Cer 12:2;2O/19:4 | 950,25 | 951,18 | 714,16 | 1474,17 | 781,82 | 754,34 | 1507,58 | 533,06 | 958,32 |
| Cer 13:2;2O/30:3 | 2686,62 | 1619 | 1818,06 | 2314,88 | 2326,49 | 3205,37 | 2763,16 | 1554,04 | 2285,9525 |
| Cer 20:2;4O|Cer 12:2;3O/8:0;(2OH) | 1469,45 | 7000,22 | 2736,79 | 1817,84 | 1743,61 | 1708,54 | 1102,85 | 4974,17 | 2819,18375 |
| Cer 24:3;3O|Cer 15:3;2O/9:0;O | 1162,12 | 2074,49 | 1627,68 | 1295,68 | 1355,76 | 1076,58 | 1127,09 | 1567,7 | 1410,8875 |
| Cer 32:0;2O|Cer 16:0;2O/16:0 | 470,55 | 687,68 | 580,02 | 758,34 | 584,54 | 690,08 | 468,36 | 455,25 | 586,8525 |
| Cer 32:1;2O|Cer 16:1;2O/16:0 | 760,32 | 499,03 | 615,6 | 580,69 | 458,82 | 648,12 | 537,28 | 175,74 | 534,45 |
| Cer 33:1;2O|Cer 17:1;2O/16:0 | 320,24 | 176,13 | 255,37 | 248,92 | 184,38 | 127,38 | 236,15 | 109,54 | 207,26375 |
| Cer 33:1;4O|Cer 18:0;3O/15:1;(2OH) | 1633,89 | 3085,58 | 1798,82 | 2693,75 | 2043,69 | 1913,15 | 1550,01 | 2298,47 | 2127,17 |
| Cer 34:1;2O|Cer 18:1;2O/16:0 | 1912,22 | 2166,74 | 3234,33 | 3372,88 | 3534 | 2700,56 | 2131,47 | 1187,39 | 2529,94875 |
| Cer 34:1;3O|Cer 19:0;2O/15:1;O | 2138,44 | 1939,76 | 2190,06 | 2906,98 | 1406,28 | 2032,17 | 1652,08 | 1784,97 | 2006,3425 |
| Cer 34:3;2O|Cer 12:2;2O/22:1 | 1418,02 | 2782,46 | 1337,58 | 2069,7 | 2592,27 | 1988,42 | 1858,31 | 1140,7 | 1898,4325 |
| Cer 36:0;3O|Cer 18:0;2O/18:0;O | 9462,13 | 15943,4 | 12644,53 | 13835,64 | 13567,52 | 11016,8 | 8143,64 | 13623,41 | 12279,625 |
| Cer 36:1;3O|Cer 19:0;2O/17:1;O | 457,92 | 487,5 | 528,86 | 760,08 | 421,99 | 482,42 | 384,43 | 574,36 | 512,195 |
| Cer 36:3;4O|Cer 19:2;3O/17:1;(2OH) | 930,09 | 1089,02 | 1125,74 | 2085,42 | 1292,77 | 971,42 | 1214,65 | 1143,83 | 1231,6175 |
| Cer 37:0;2O|Cer 19:0;2O/18:0 | 742,05 | 2137,54 | 1038,85 | 1616,6 | 1061,09 | 773,62 | 490,11 | 2392,36 | 1281,5275 |
| Cer 38:0;4O | 515,36 | 2576,03 | 1316,3 | 1718,92 | 1173,1 | 810,61 | 805,15 | 1859,02 | 1346,81125 |
| Cer 39:0;3O|Cer 18:0;2O/21:0;O | 2931,69 | 13533,2 | 6329,98 | 8939,07 | 9382,73 | 2430,76 | 2813,76 | 8385,14 | 6843,29 |
| Cer 40:0;2O|Cer 16:0;2O/24:0 | 718,17 | 1103,83 | 583,22 | 1172,46 | 740,43 | 766,66 | 562,97 | 847,98 | 811,965 |
| Cer 40:1;2O|Cer 16:1;2O/24:0 | 3443,64 | 4794,25 | 3400,08 | 4620,46 | 3419,5 | 2514 | 2676,11 | 2256,76 | 3390,6 |
| Cer 41:1;2O|Cer 18:1;2O/23:0 | 1180,17 | 1132,82 | 1275,25 | 1901,85 | 1388,21 | 752,3 | 826,78 | 1155,39 | 1201,59625 |
| Cer 42:1;2O|Cer 18:1;2O/24:0 | 3328,24 | 2978,06 | 3020,68 | 3537,4 | 3432,91 | 2482,54 | 1882,64 | 2491,7 | 2894,27125 |
| Cer 44:2;4O|Cer 28:1;3O(FA 16:0) | 1255,54 | 888,93 | 1041,67 | 1750,87 | 1341,63 | 1650,32 | 620,04 | 345,92 | 1111,865 |
| **Diacylglycerols** |  |  |  |  |  |  |  |  |  |
| DG 16:0 | 1557,39 | 1527,99 | 804,9 | 942,35 | 1190,74 | 779,3 | 1353,33 | 962,05 | 1139,75625 |
| DG 18:0 | 6224,34 | 4107,67 | 2299,06 | 3873,24 | 3777,21 | 2792,97 | 4682,32 | 1313,19 | 3633,75 |
| DG 20:0 | 30573,89 | 12254,02 | 8148,79 | 21012,81 | 18023,28 | 17824,76 | 16719,21 | 4880,78 | 16179,6925 |
| DG 21:0 | 617,07 | 766,28 | 397,73 | 563,28 | 638,91 | 384,52 | 570,94 | 399,03 | 542,22 |
| DG 22:0 | 19011,54 | 6954,76 | 4267,92 | 13509,28 | 12888,94 | 10203,69 | 12812,29 | 3128,91 | 10347,16625 |

| DG 22:0|DG 10:0_12:0 | 881,27 | 707,16 | 425,55 | 832,74 | 661,19 | 673,02 | 2045,43 | 377,69 | 825,50625 |
| --- | --- | --- | --- | --- | --- | --- | --- | --- | --- |
| DG 22:0|DG 8:0_14:0 | 1000,47 | 729,37 | 412,82 | 879,86 | 812,79 | 683,26 | 1985,81 | 437,89 | 867,78375 |
| DG 22:1 | 6085,92 | 4535,66 | 2687,96 | 4956,12 | 4436,29 | 2725,42 | 4057,08 | 1374,5 | 3857,36875 |
| DG 22:1|DG 8:0_14:1 | 7003,06 | 4091,5 | 1933,31 | 3887,35 | 4021,97 | 3025,41 | 3957,03 | 1222,42 | 3642,75625 |
| DG 24:0 | 11486,24 | 6445,85 | 4392,2 | 8277,24 | 7396,72 | 7430,95 | 38039,7 | 3516,48 | 10873,1763 |
| DG 24:0|DG 10:0_14:0 | 4662,33 | 2305,27 | 1706,42 | 3213,49 | 2829,52 | 3120,18 | 13281,4 | 1070,8 | 4023,67 |
| DG 24:0|DG 8:0_16:0 | 4069,91 | 2776,3 | 1474,94 | 4326,95 | 3391,24 | 3117,84 | 9589,85 | 1113,23 | 3732,5325 |
| DG 24:1 | 11242,66 | 5659,39 | 2784,89 | 7326,18 | 5795,98 | 5098,18 | 9820,37 | 2033,51 | 6220,145 |
| DG 24:1|DG 10:0_14:1 | 6553,31 | 2974,13 | 1653,5 | 3970,5 | 3298,22 | 2702,01 | 5208,02 | 1318,21 | 3459,7375 |
| DG 24:2 | 744,88 | 641,61 | 325,52 | 496,24 | 508,28 | 502,13 | 586,66 | 235,67 | 505,12375 |
| DG 24:3 | 443,64 | 530,9 | 187,97 | 425,47 | 403,52 | 260,09 | 453,85 | 230,86 | 367,0375 |
| DG 25:0|DG 10:0_15:0 | 800,97 | 494,34 | 420,88 | 934,06 | 729,38 | 568,57 | 1822,49 | 250,31 | 752,625 |
| DG 26:0|DG 12:0_14:0 | 9940,67 | 7921,85 | 6276,38 | 11275,37 | 11370,11 | 11449,1 | 31338,9 | 3556,16 | 11641,0575 |
| DG 26:1 | 8420,55 | 4897,96 | 2897,28 | 6626,99 | 6056,47 | 5916,38 | 14202 | 2064,24 | 6385,23125 |
| DG 26:1|DG 8:0_18:1 | 7512,88 | 3343,7 | 2349,46 | 4444,3 | 4390,75 | 4114,02 | 9942,58 | 1692,24 | 4723,74125 |
| DG 26:2 | 725,59 | 732,22 | 333,16 | 700,69 | 658,21 | 581,44 | 1030,41 | 369,96 | 641,46 |
| DG 26:2|DG 8:0_18:2 | 706,27 | 662,74 | 315,02 | 606,57 | 540,09 | 541,57 | 847,84 | 297,61 | 564,71375 |
| DG 26:3 | 304,96 | 279,35 | 184,04 | 285,24 | 337,24 | 203,34 | 614,87 | 112,77 | 290,22625 |
| DG 27:0 | 895,06 | 1411,73 | 1001,03 | 1589,11 | 1392,48 | 797,94 | 2647 | 832,42 | 1320,84625 |
| DG 27:5 | 882,6 | 1121,44 | 819,98 | 928,28 | 791,95 | 901,49 | 1633,48 | 812,37 | 986,44875 |
| DG 28:0|DG 12:0_16:0 | 30678,93 | 27092,7 | 22287,28 | 37486,96 | 34784,58 | 27558,2 | 87976,7 | 13830,26 | 35211,9475 |
| DG 28:1 | 10476,26 | 12842,2 | 9072,88 | 15366,53 | 14552,77 | 10137,3 | 20755,9 | 5289,33 | 12311,65 |
| DG 28:1|DG 10:0_18:1 | 20654,56 | 10706,5 | 8209,12 | 16223,74 | 15578,31 | 16701,9 | 39187,4 | 4300,33 | 16445,2288 |
| DG 28:2 | 3174,4 | 3688,1 | 2493,37 | 3980,69 | 3194,7 | 3345,77 | 60861,1 | 2094,58 | 10354,0875 |
| DG 28:2|DG 14:1_14:1 | 4719,56 | 3512,7 | 2193,25 | 4114,07 | 3068,3 | 3990,14 | 6319,86 | 1883,4 | 3725,16 |
| DG 28:3 | 619,96 | 699,98 | 298,87 | 770,42 | 541,99 | 529,92 | 1420,75 | 240,56 | 640,30625 |
| DG 28:3|DG 10:0_18:3 | 663,93 | 582,24 | 314,78 | 664,28 | 530,62 | 484,09 | 1468,43 | 235,22 | 617,94875 |
| DG 30:0 | 796,69 | 1024,83 | 583,42 | 1291,12 | 976,34 | 844,49 | 1208,59 | 397,21 | 890,33625 |
| DG 30:0|DG 14:0_16:0 | 75329,6 | 100916 | 88497,26 | 152746,7 | 157571,35 | 117046 | 148969 | 51658,54 | 111591,794 |
| DG 30:1|DG 16:0_14:1 | 32107,89 | 33259,2 | 25105,63 | 60388,71 | 48915,63 | 53631,6 | 99258,4 | 13147,41 | 45726,8075 |
| DG 30:2 | 1610,73 | 2687,03 | 1726,09 | 2682,55 | 2632,29 | 1789,06 | 4013,03 | 1136,13 | 2284,61375 |
| DG 30:2|DG 12:0_18:2 | 3899,58 | 2663,85 | 1731,03 | 3269,84 | 2763,05 | 3387,31 | 7613,02 | 1057,58 | 3298,1575 |
| DG 30:2|DG 14:1_16:1 | 4026,11 | 2850,83 | 1866,64 | 3620,45 | 3384,09 | 3650,47 | 7200,3 | 1000,73 | 3449,9525 |
| DG 30:3|DG 12:0_18:3 | 1198,53 | 782,77 | 481,43 | 1135,04 | 869,32 | 864,21 | 2765,61 | 364,03 | 1057,6175 |
| DG 30:5 | 164,24 | 819,58 | 417,44 | 502,47 | 502,03 | 223,98 | 10800,2 | 786,33 | 1777,03125 |
| DG 30:6 | 4474,35 | 6891,24 | 4657,52 | 5595,42 | 4314,43 | 3698,47 | 6214,39 | 3247,27 | 4886,63625 |
| DG 30:7 | 507,91 | 936,14 | 592,69 | 745,57 | 691,08 | 543,25 | 614,16 | 462,17 | 636,62125 |
| DG 31:7 | 26975,81 | 57580,8 | 28949,43 | 48885,9 | 40468,67 | 34032,5 | 29437,4 | 22968,2 | 36162,3475 |
| DG 31:8 | 1520,61 | 2243,44 | 1071,7 | 1914,89 | 1478,99 | 1420,65 | 2305,29 | 1022,89 | 1622,3075 |
| DG 32:1|DG 14:0_18:1 | 91761,55 | 80139,2 | 88644,35 | 169268,95 | 194395,75 | 126348 | 170146 | 41436,8 | 120267,579 |
| DG 32:2 | 5172,11 | 11511,3 | 6931,24 | 12363,07 | 10449,82 | 5260,21 | 10365,7 | 3995,45 | 8256,1175 |
| DG 32:2|DG 14:1_18:1 | 21849,74 | 15541,9 | 9800,35 | 24037,92 | 20511,92 | 19770,9 | 33182,7 | 4557,06 | 18656,555 |
| DG 32:3 | 887,46 | 2631,35 | 1242,82 | 2205,03 | 2180,83 | 1079,59 | 1814,82 | 1024,13 | 1633,25375 |
| DG 32:3|DG 14:0_18:3 | 3417 | 3559,09 | 1974,67 | 2985,17 | 3852,28 | 2985,78 | 6834,44 | 1406,48 | 3376,86375 |
| DG 32:6 | 4618,41 | 13163,7 | 7233,63 | 8848,03 | 8265,32 | 4971,24 | 7570,32 | 6353,5 | 7628,0225 |
| DG 32:7 | 1398,48 | 3312,72 | 1719,33 | 2166,29 | 2156,62 | 1509,67 | 1570,98 | 1375,91 | 1901,25 |

| DG 33:2 | 1183,41 | 1946,42 | 788,45 | 2281,36 | 1649,2 | 1180,43 | 833,37 | 1336,72 | 1399,92 |
| --- | --- | --- | --- | --- | --- | --- | --- | --- | --- |
| DG 33:3 | 496,73 | 1166,36 | 423,17 | 692,41 | 705,92 | 590,04 | 414,69 | 592,51 | 635,22875 |
| DG 33:6 | 25827,41 | 184790 | 87146,11 | 102258,92 | 96873,25 | 50537,5 | 42827,5 | 106654,55 | 87114,3538 |
| DG 33:8 | 4923,22 | 8153,59 | 3948,35 | 8351,74 | 6133,63 | 6051,05 | 5920,57 | 2937 | 5802,39375 |
| DG 33:9 | 971,64 | 1430,41 | 871,05 | 1428,88 | 666,75 | 799,28 | 1022,68 | 842,78 | 1004,18375 |
| DG 34:0 | 529,03 | 498,75 | 623,98 | 1127,79 | 745,48 | 750,34 | 572,54 | 461,76 | 663,70875 |
| DG 34:0|DG 16:0_18:0 | 4825,68 | 14561,6 | 12174,53 | 16751 | 13536,16 | 8259,4 | 10400,4 | 13494,01 | 11750,3575 |
| DG 34:1|DG 16:0_18:1 | 120781,3 | 187032 | 195175,3 | 304638,25 | 196870,95 | 136943 | 218577 | 130302,7 | 186290,031 |
| DG 34:2 | 262,85 | 885,97 | 606,75 | 335,58 | 402,95 | 267,78 | 269,38 | 575,16 | 450,8025 |
| DG 34:2|DG 16:0_18:2 | 51799,41 | 45562,9 | 33834,76 | 89804 | 81898,52 | 65844 | 88501,3 | 15939,96 | 59148,0963 |
| DG 34:3|DG 16:0_18:3 | 8001,26 | 10704,4 | 6628,06 | 17697,76 | 17264,74 | 11736,3 | 15005,5 | 3341,46 | 11297,4363 |
| DG 34:6 | 5197,11 | 16716,4 | 11609,64 | 11124,18 | 8039,52 | 6192,34 | 8177,69 | 10116,59 | 9646,68875 |
| DG 34:7 | 2461,49 | 5872,12 | 3214,31 | 4601,86 | 3201,47 | 3036,33 | 2836,63 | 2660,96 | 3485,64625 |
| DG 35:2 | 901,54 | 1497 | 646,09 | 1470,17 | 975,59 | 807,43 | 688 | 927,98 | 989,225 |
| DG 35:3 | 417,81 | 986,98 | 401,65 | 661,85 | 470,98 | 525,44 | 267,3 | 439,65 | 521,4575 |
| DG 35:8 | 12690,09 | 35026,4 | 14972,71 | 25324,54 | 21036,18 | 17050,4 | 13621,2 | 12873,22 | 19074,3513 |
| DG 35:9 | 3752,13 | 7216 | 2960,89 | 6437,47 | 4764,47 | 3177,39 | 7640,51 | 3453,17 | 4925,25375 |
| DG 36:1|DG 18:0_18:1 | 3186,89 | 12708,4 | 13639,65 | 15252,51 | 13856,95 | 7603,1 | 14336,3 | 9533,29 | 11264,6313 |
| DG 36:2|DG 18:1_18:1 | 28662,89 | 36807,6 | 52475,3 | 73968,93 | 57853,05 | 24556,5 | 58368,3 | 25957,52 | 44831,255 |
| DG 36:3|DG 18:1_18:2 | 14402,49 | 15704,6 | 13037,66 | 22052,31 | 20674,83 | 14670,3 | 20445,9 | 6191,84 | 15897,48 |
| DG 36:4|DG 18:1_18:3 | 3583,49 | 4639,57 | 3228,18 | 7170,85 | 5195,51 | 3574,96 | 5484,93 | 2342,23 | 4402,465 |
| DG 36:5|DG 16:0_20:5 | 909,74 | 1095,32 | 643,18 | 1450,23 | 1141,22 | 1061,29 | 1217,88 | 316,6 | 979,4325 |
| DG 36:5|DG 18:2_18:3 | 1030,67 | 1035,92 | 589,6 | 1295,65 | 1005,89 | 879,27 | 1489,47 | 445,01 | 971,435 |
| DG 36:7 | 3386,26 | 12804,3 | 6820,21 | 8015,8 | 6811,8 | 4645,72 | 3494,73 | 5171,47 | 6393,7875 |
| DG 36:8 | 269,1 | 946,92 | 381,87 | 558,93 | 561,53 | 391,37 | 267,98 | 512,81 | 486,31375 |
| DG 37:9 | 6334,5 | 13893,2 | 5983,19 | 10154,64 | 8768,21 | 6054,43 | 10726 | 5808,07 | 8465,2875 |
| DG 38:5|DG 16:0_22:5 | 5837,27 | 5641,11 | 5492,25 | 12383,39 | 9720,14 | 7415,8 | 10776,5 | 2696,39 | 7495,36125 |
| DG 38:6 | 295,45 | 642,4 | 461,33 | 822,19 | 599,01 | 394,69 | 353,86 | 438,53 | 500,9325 |
| DG 40:6|DG 18:1_22:5 | 1074,18 | 1668,32 | 1254,18 | 2172,27 | 1784,55 | 1625,98 | 1400,72 | 1032,56 | 1501,595 |
| DG 40:8 | 428,32 | 1575,04 | 674,29 | 918,35 | 996,65 | 560,45 | 407,1 | 926,77 | 810,87125 |
| DG 41:10 | 793,45 | 3697,78 | 1136,74 | 2771,35 | 2897,12 | 1118,47 | 644,62 | 1581,16 | 1830,08625 |
| DG 41:11 | 284,72 | 1062,16 | 470,08 | 955,01 | 699 | 431,45 | 343,11 | 664,15 | 613,71 |
| DG 41:5 | 2078,75 | 7978,7 | 2954,82 | 5541,28 | 4256,78 | 2134,6 | 954,1 | 3629,22 | 3691,03125 |
| DG 41:6 | 12968,16 | 57273,5 | 22384,41 | 33194,72 | 22833,2 | 8461,2 | 9458,05 | 39533,43 | 25763,3338 |
| DG 43:11 | 675,64 | 2776,44 | 1135,32 | 1966,34 | 1671,86 | 766,46 | 518,04 | 1304,29 | 1351,79875 |
| DG 43:3 | 488,57 | 1613,99 | 805,28 | 1348,09 | 1116,17 | 726,86 | 515,48 | 1179,21 | 974,20625 |
| DG 43:4 | 1542,2 | 6902,73 | 3149,4 | 3942,51 | 4185,29 | 2021,46 | 2051,73 | 5332,41 | 3640,96625 |
| DG 43:6 | 10081,7 | 51801,6 | 19503,04 | 31856,29 | 20536,49 | 10581,3 | 9105,55 | 35740,86 | 23650,8575 |
| DG 43:7 | 13222,71 | 34904,4 | 15965,72 | 19107,44 | 15821,65 | 8757,35 | 11755,4 | 22635,88 | 17771,3238 |
| DG 44:7 | 1508,59 | 1142,45 | 918,15 | 1171,84 | 1139,99 | 1035,07 | 1461,62 | 611,78 | 1123,68625 |
| DG 44:9 | 1327,72 | 627,56 | 970,96 | 1311,32 | 1465,51 | 2092,46 | 1069,43 | 994,56 | 1232,44 |
| DG 46:6 | 3883,3 | 4024,16 | 5046,46 | 4764,94 | 5236,89 | 4269,79 | 4686,07 | 5645,33 | 4694,6175 |
| DG 47:7 | 6329,89 | 22696,3 | 9783,18 | 14195,03 | 13928,36 | 7910,29 | 7530,05 | 14935,55 | 12163,5825 |
| DG 47:8 | 7335,42 | 21218,8 | 8878,84 | 11659,94 | 10630,41 | 6662,19 | 6295,53 | 12971,35 | 10706,555 |
| DG 51:7 | 3199,42 | 5979,07 | 3571,27 | 5652,21 | 5166,03 | 2876 | 1956,44 | 5816,4 | 4277,105 |
| DG 51:8 | 1412,97 | 2680,9 | 1838,79 | 3306,95 | 2605,02 | 1776,48 | 1463,66 | 2087,62 | 2146,54875 |
|  |  |  |  |  |  |  |  |  |  |
| **Free Fatty Acids** |  |  |  |  |  |  |  |  |  |
|  |  |  |  |  |  |  |  |  |  |

| FA 12:0 | 1012,72 | 1228,62 | 810,52 | 911,75 | 1029,76 | 695,94 | 968,59 | 921,37 | 947,40875 |
| --- | --- | --- | --- | --- | --- | --- | --- | --- | --- |
| FA 14:0 | 9346,48 | 5477,67 | 5457,62 | 5216,5 | 4502,03 | 5756,67 | 6090,12 | 4212,89 | 5757,4975 |
| FA 14:1 | 1576,32 | 490,13 | 832,98 | 434,92 | 621,25 | 1045,19 | 566,69 | 110,17 | 709,70625 |
| FA 15:0 | 743,84 | 970,94 | 869,78 | 682,81 | 698,95 | 535,19 | 597,86 | 1627,3 | 840,83375 |
| FA 15:4 | 1282,31 | 8154,08 | 4945,98 | 3848,01 | 4308,13 | 1958,65 | 1542,22 | 6450,5 | 4061,235 |
| FA 16:0 | 418672 | 530757 | 436200,2 | 563923,1 | 506751,25 | 441764 | 389880 | 556820,3 | 480595,85 |
| FA 16:0;(2OH) | 417,87 | 875,76 | 591,62 | 571,34 | 547,01 | 555,03 | 245,83 | 557,54 | 545,25 |
| FA 16:0;O | 2235,17 | 3137,93 | 3196,21 | 2982,57 | 2836 | 2351,74 | 1579,34 | 2810,76 | 2641,215 |
| FA 16:1 | 8756,42 | 3998,42 | 6842,69 | 4319,25 | 3632,35 | 9173,44 | 4674,04 | 2325,39 | 5465,25 |
| FA 16:1;O | 2045,24 | 3681,73 | 2916,42 | 3828,66 | 3338,17 | 2781,28 | 1684,11 | 2738,88 | 2876,81125 |
| FA 17:0 | 1253,81 | 1875,05 | 1420,67 | 1425,77 | 1060,72 | 1294,04 | 1050,9 | 2871,29 | 1531,53125 |
| FA 17:1 | 184,47 | 143,55 | 211,99 | 94,54 | 1,72 | 236,83 | 131,54 | 285,05 | 161,21125 |
| FA 17:4;O | 596,67 | 493,66 | 1154,66 | 901,31 | 1271,68 | 1365,04 | 517,69 | 606,85 | 863,445 |
| FA 18:0 | 427544,3 | 635713 | 495884,9 | 633081,8 | 563393,3 | 509285 | 404618 | 641306,7 | 538853,331 |
| FA 18:0;(2OH) | 2164,71 | 5435,97 | 5158,62 | 3895,98 | 2806,06 | 2962,13 | 1539,53 | 3878,3 | 3480,1625 |
| FA 18:0;O | 4583,98 | 8177,52 | 4256,16 | 4684,19 | 4555,46 | 3520,52 | 2109,5 | 4460,56 | 4543,48625 |
| FA 18:1 | 38497,17 | 45010,2 | 58318 | 26615,36 | 27540,76 | 43268,7 | 30065,2 | 21048,12 | 36295,4425 |
| FA 18:1;2O | 431,39 | 1100,78 | 1046,68 | 788,24 | 491,17 | 457,17 | 264,5 | 735,55 | 664,435 |
| FA 18:1;O | 2710,63 | 4490,18 | 3366,22 | 2546,77 | 1550,06 | 1935,06 | 1045,34 | 2456,41 | 2512,58375 |
| FA 18:2 | 2775,99 | 3062,38 | 3228,14 | 1847,83 | 1663,23 | 2384,67 | 2040,91 | 1600,02 | 2325,39625 |
| FA 18:2;O | 1923,03 | 2788,76 | 2805,24 | 3344,6 | 2538,4 | 2377,2 | 1243,42 | 2861,42 | 2485,25875 |
| FA 18:3 | 2733,17 | 1524,53 | 1874,39 | 976,2 | 856,91 | 1388,57 | 2020,28 | 247,27 | 1452,665 |
| FA 18:3;4O | 600,43 | 1356,35 | 1180,81 | 1218,32 | 817,29 | 754,79 | 376,41 | 1448,02 | 969,0525 |
| FA 18:3;O | 303,84 | 174,36 | 492,88 | 360,3 | 198,21 | 337,89 | 162,3 | 297,22 | 290,875 |
| FA 19:0 | 242,36 | 462,37 | 268,24 | 344,99 | 302,44 | 263,31 | 213,97 | 731,61 | 353,66125 |
| FA 19:0;(2OH) | 878,67 | 992,27 | 722,98 | 906,3 | 1119,89 | 1280,5 | 961,34 | 606,11 | 933,5075 |
| FA 19:1 | 173,69 | 151,53 | 319,68 | 2,29 | 61,8 | 91,32 | 116,31 | 54,76 | 121,4225 |
| FA 19:1;2O | 293,15 | 1125,71 | 1130,68 | 1004,67 | 766,59 | 547,34 | 347,39 | 1041,37 | 782,1125 |
| FA 19:1;O | 301,8 | 215,95 | 213,06 | 115,74 | 84,31 | 159,33 | 76,78 | 250,07 | 177,13 |
| FA 20:0 | 4512,97 | 6138,68 | 5637,21 | 5873,7 | 5388,55 | 4929,79 | 3958,8 | 8385 | 5603,0875 |
| FA 20:1 | 477,12 | 318,57 | 1170,83 | 370,15 | 290,18 | 597,41 | 336,26 | 332,52 | 486,63 |
| FA 20:3 | 142,14 | 175,6 | 193,69 | 110,92 | 242,33 | 247,18 | 168,46 | 188,21 | 183,56625 |
| FA 20:4 | 1671,06 | 445,18 | 1197,34 | 798,4 | 648,31 | 1666,94 | 842,07 | 519,59 | 973,61125 |
| FA 20:5 | 491,35 | 576,07 | 1397 | 595,94 | 375,22 | 373,55 | 254,42 | 1161,06 | 653,07625 |
| FA 21:0 | 603,24 | 302,63 | 687,14 | 816,17 | 368,35 | 612,06 | 379,12 | 409,94 | 522,33125 |
| FA 21:1;2O | 193,24 | 488,1 | 716,65 | 543,39 | 486,74 | 264,99 | 213,65 | 620,62 | 440,9225 |
| FA 22:0 | 1782,04 | 3032,82 | 2287,72 | 2211,47 | 1605,78 | 2320,27 | 1293,61 | 3749,44 | 2285,39375 |
| FA 22:1 | 110,34 | 217,78 | 142,08 | 37,66 | 156,89 | 110,75 | 56,28 | 316,58 | 143,545 |
| FA 22:5 | 727,84 | 708,77 | 904,87 | 563,91 | 717,75 | 1286,68 | 612,13 | 959,38 | 810,16625 |
| FA 22:6 | 894,11 | 1674,77 | 1839,8 | 1179,63 | 1044,22 | 1202,65 | 722,28 | 1138,55 | 1212,00125 |
| FA 23:0 | 1133,98 | 1732,39 | 1800,56 | 1839,11 | 821,37 | 1059,58 | 693,27 | 2433,9 | 1439,27 |
| FA 23:1 | 92,81 | 0,64 | 88,02 | 62,68 | 95,78 | 134,91 | 75,68 | 0,64 | 68,895 |
| FA 24:0 | 7309,78 | 10720,6 | 10832,19 | 10773,66 | 6434,72 | 6940,77 | 5098,04 | 13872,41 | 8997,77625 |
| FA 25:0 | 2572,67 | 6399,44 | 5004,88 | 3909,56 | 2860,93 | 3095,19 | 1945,96 | 8251 | 4254,95375 |
| FA 26:0 | 14147,04 | 25131 | 21496,67 | 23004,98 | 14566,67 | 16529,5 | 9923,48 | 30374,42 | 19396,7188 |

| FA 26:1;O | 889,63 | 1205,03 | 1814,22 | 1525,49 | 1135,69 | 1547,92 | 659,62 | 1483,49 | 1282,63625 |
| --- | --- | --- | --- | --- | --- | --- | --- | --- | --- |
| FA 27:0 | 5260,81 | 7109,74 | 6170,48 | 6884,85 | 3946,85 | 5215,13 | 3584,37 | 9790,16 | 5995,29875 |
| FA 27:1;O | 300,66 | 280,75 | 465,97 | 446,38 | 153,8 | 403,96 | 163,25 | 375,31 | 323,76 |
| FA 28:0 | 20531,94 | 38285,9 | 28013,97 | 32402,84 | 17515,73 | 22123,4 | 12603,9 | 46941,62 | 27302,4 |
| FA 29:0 | 5716,66 | 8139,92 | 7395,74 | 8319,04 | 4682,13 | 5154,78 | 3555,62 | 13179,6 | 7017,93625 |
| FA 30:0 | 32542,77 | 36067,2 | 30076,21 | 54650,85 | 20155,52 | 34268,2 | 17861,2 | 52766,94 | 34798,6138 |
| FA 31:0 | 3483,02 | 6704,21 | 4895,58 | 9021,1 | 3105,93 | 3597,95 | 2404,72 | 10932,02 | 5518,06625 |
| FA 32:0 | 8746,57 | 15849,8 | 12223,94 | 26214,27 | 8165,59 | 10415,3 | 6068,08 | 21777,19 | 13682,5825 |
| FA 33:0 | 1113,84 | 1957,39 | 1491,42 | 3555,18 | 980,33 | 1308,5 | 724,4 | 4080,28 | 1901,4175 |
| FA 34:0 | 2012,5 | 4423,97 | 3216,95 | 8000,35 | 2637,02 | 2393,27 | 1341,23 | 8314,96 | 4042,53125 |
| FA 34:8 | 1989,29 | 638,95 | 704,75 | 1110,2 | 461,84 | 2099,23 | 1227,25 | 219,16 | 1056,33375 |
| FA 42:5 | 676402,2 | 2700972 | 1792027 | 2303877,5 | 1456408,5 | 1202451 | 295972 | 3288372,5 | 1714560,29 |
| FA 42:6 | 849,84 | 2211,09 | 1764,54 | 2095,36 | 1327,73 | 1242,72 | 647,38 | 2779,18 | 1614,73 |
| FA 44:5 | 239273,6 | 805526 | 519092,9 | 661654,5 | 445586,6 | 362409 | 90232,3 | 966610,6 | 511298,163 |
| FA 44:6 | 700,67 | 1071,94 | 1269,13 | 1402,63 | 786,91 | 790,12 | 404,04 | 2103,62 | 1066,1325 |
| **Dihexosylceramides** |  |  |  |  |  |  |  |  |  |
| Hex2Cer 32:1;2O|Hex2Cer 16:1;2O/16:0 | 139,91 | 136,24 | 147,21 | 291,37 | 179,72 | 254,07 | 252,79 | 144,64 | 193,24375 |
| Hex2Cer 33:1;2O|Hex2Cer 17:1;2O/16:0 | 102,53 | 79,44 | 151,8 | 254 | 149,54 | 174,92 | 176,92 | 114,44 | 150,44875 |
| Hex2Cer 34:0;2O | 414,17 | 239,88 | 569,68 | 1223,67 | 750,78 | 756,69 | 827,95 | 317,09 | 637,48875 |
| Hex2Cer 34:1;2O|Hex2Cer 18:1;2O/16:0 | 2501,07 | 1212,81 | 3283,44 | 8789,7 | 5541,68 | 6280,48 | 5791,29 | 1662,31 | 4382,8475 |
| Hex2Cer 36:1;2O|Hex2Cer 18:1;2O/18:0 | 54,93 | 127,75 | 107,56 | 202,91 | 164,61 | 66,92 | 162,42 | 100,51 | 123,45125 |
| Hex2Cer 38:1;2O|Hex2Cer 16:1;2O/22:0 | 505,08 | 452,36 | 509,5 | 1053,02 | 617,8 | 663,2 | 873,9 | 435,91 | 638,84625 |
| Hex2Cer 39:0;2O|Hex2Cer 17:0;2O/22:0 | 478 | 452,85 | 561,16 | 1323,14 | 719,58 | 719,58 | 890,88 | 579,33 | 715,565 |
| Hex2Cer 39:1;2O|Hex2Cer 16:1;2O/23:0 | 687,66 | 467,36 | 845,96 | 1863,32 | 860,35 | 1266,99 | 1201,35 | 650,83 | 980,4775 |
| Hex2Cer 40:1;2O|Hex2Cer 18:1;2O/22:0 | 1092,94 | 1256,31 | 1442,44 | 3707,61 | 2487,12 | 2026 | 1781,11 | 1304,55 | 1887,26 |
| Hex2Cer 40:2;2O|Hex2Cer 16:1;2O/24:1 | 64,67 | 60,57 | 67,47 | 105,08 | 93,41 | 84,64 | 125,16 | 35,86 | 79,6075 |
| Hex2Cer 41:1;2O|Hex2Cer 18:1;2O/23:0 | 546,79 | 779,09 | 1194,73 | 2893,08 | 1640,75 | 1061,42 | 847,04 | 1284,49 | 1280,92375 |
| Hex2Cer 42:1;2O|Hex2Cer 18:1;2O/24:0 | 518,29 | 795,16 | 1228,98 | 2965,03 | 2084,05 | 928,9 | 1002,89 | 1070,88 | 1324,2725 |
| Hex2Cer 42:2;2O|Hex2Cer 18:1;2O/24:1 | 79,71 | 130,29 | 226,23 | 461,52 | 264,59 | 255,23 | 206,84 | 175,28 | 224,96125 |
| HexCer 33:1;2O|HexCer 17:1;2O/16:0 | 414,4 | 408,17 | 438,9 | 634,51 | 510,9 | 623,8 | 528,65 | 343,31 | 487,83 |
| HexCer 34:0;2O|HexCer 18:0;2O/16:0 | 214,86 | 229,43 | 126,45 | 506,73 | 443,73 | 320,25 | 284,27 | 161,11 | 285,85375 |
| HexCer 34:1;2O | 792,95 | 579,89 | 1004,66 | 1529,11 | 1356,85 | 1304,43 | 1146,04 | 425,7 | 1017,45375 |
| HexCer 34:1;2O|HexCer 18:1;2O/16:0 | 1321,17 | 1052,82 | 1103,66 | 1965,72 | 2050,03 | 2190,57 | 1443,73 | 1071,98 | 1524,96 |
| HexCer 34:1;3O|HexCer 18:1;2O/16:0;O | 492,01 | 433,48 | 523,74 | 646,58 | 667,63 | 689,88 | 356,68 | 357,34 | 520,9175 |
| HexCer 38:1;2O | 294,33 | 73,52 | 111,99 | 145,74 | 244,81 | 239,09 | 496,93 | 48,27 | 206,835 |
| HexCer 39:1;3O | 1449,75 | 1114,66 | 474,84 | 957,67 | 691,63 | 1324,21 | 577,3 | 817,1 | 925,895 |
| HexCer 40:0;3O | 466,3 | 109,42 | 208,2 | 168,55 | 233,05 | 369,36 | 208,41 | 121,07 | 235,545 |
| HexCer 40:1;2O | 2672,18 | 548,84 | 820,55 | 1844 | 2098,34 | 2653,92 | 2544,01 | 692,31 | 1734,26875 |
| HexCer 40:1;2O|HexCer 18:1;2O/22:0 | 402,65 | 1098,38 | 532,71 | 1464,52 | 1031,58 | 763,08 | 498,31 | 904,22 | 836,93125 |
| HexCer 40:1;3O | 2409,93 | 1548,22 | 1037,49 | 1847,99 | 1534,55 | 2431,56 | 1002,59 | 924,9 | 1592,15375 |
| HexCer 41:1;2O | 2100,43 | 1180,31 | 1031,94 | 1470,02 | 1753,03 | 2037,5 | 2141,93 | 1181,13 | 1612,03625 |
| HexCer 41:1;2O|HexCer 18:1;2O/23:0 | 3942,58 | 7765,64 | 5165,05 | 10124,42 | 9056,64 | 5110,75 | 4739,55 | 5147,44 | 6381,50875 |
| HexCer 41:1;3O | 3753,32 | 1290,64 | 987,31 | 2583,91 | 2344,03 | 4147,65 | 1327,24 | 1675,92 | 2263,7525 |
| HexCer 42:1;2O | 4512,33 | 1739,3 | 1167,51 | 2827,85 | 2727,47 | 4220,27 | 3864,81 | 912,09 | 2746,45375 |
| HexCer 42:1;3O | 2429,59 | 1609,05 | 944,52 | 2641,45 | 2469,38 | 3792,64 | 1037,52 | 1607 | 2066,39375 |
| HexCer 43:1;3O | 1241,8 | 75,57 | 153,4 | 368,94 | 380,4 | 1114,14 | 310,22 | 203,27 | 480,9675 |

| HexCer 49:5;4O | 1348,19 | 907,33 | 1182,91 | 1642,72 | 1560,37 | 1766,65 | 2909,52 | 750,43 | 1508,515 |
| --- | --- | --- | --- | --- | --- | --- | --- | --- | --- |
| HexCer 51:9;3O | 395,12 | 292,88 | 235,88 | 1182,03 | 609,32 | 589,18 | 740,13 | 254,48 | 537,3775 |
| HexCer 53:9;3O | 308,99 | 457,61 | 420,23 | 1323,91 | 918,61 | 528,77 | 426,97 | 345,16 | 591,28125 |
| **Lysophophatidylcholines** |  |  |  |  |  |  |  |  |  |
| LPC 14:0 | 1885,82 | 2173 | 771,02 | 1050,03 | 1825,52 | 1378,92 | 2961,85 | 1715,39 | 1720,19375 |
| LPC 15:0 | 1221,56 | 1395,35 | 838,2 | 700,41 | 952,62 | 957,55 | 1307,93 | 1454,9 | 1103,565 |
| LPC 16:0 | 16884,36 | 9130,35 | 3265,03 | 7439,46 | 10121,01 | 11767,4 | 19693,3 | 7971 | 10783,9825 |
| LPC 17:0 | 358,58 | 393,85 | 185,95 | 208,35 | 350,64 | 323,56 | 444,4 | 385,44 | 331,34625 |
| LPC 18:0 | 1583,92 | 1313,54 | 665,9 | 973,58 | 883,05 | 1511,31 | 2773,41 | 1343,68 | 1381,04875 |
| LPC 18:1 | 3853,16 | 2732,3 | 2707,51 | 1986,01 | 2561,8 | 3276,86 | 3610,94 | 2580,65 | 2913,65375 |
| LPC 18:2 | 1445,58 | 2014,12 | 991,59 | 964,26 | 1631,43 | 1140,47 | 1280,73 | 1465,48 | 1366,7075 |
| LPC 26:0 | 400,36 | 441,17 | 392,1 | 484,46 | 419,42 | 470,3 | 503,88 | 443,95 | 444,455 |
| LPC 28:7 | 131,85 | 605,55 | 210,85 | 247,29 | 445,21 | 205,56 | 146,55 | 472,34 | 308,15 |
| **Lysophosphatidylethanolamines** |  |  |  |  |  |  |  |  |  |
| LPE 16:0 | 3001,24 | 2346,46 | 724,6 | 2171,6 | 2482,75 | 2078,7 | 2550,51 | 1712,16 | 2133,5025 |
| LPE 16:1 | 258,45 | 107,01 | 194,72 | 347,56 | 217,73 | 289,23 | 167,93 | 174,18 | 219,60125 |
| LPE 18:0 | 1729,45 | 1128,62 | 755,78 | 1087,85 | 1216,22 | 1652,7 | 2897,05 | 1000,91 | 1433,5725 |
| LPE 18:1 | 8865,85 | 7304,81 | 4616,57 | 5467,39 | 5236,44 | 7030,23 | 5429,2 | 4798,65 | 6093,6425 |
| LPE 18:2 | 2713,37 | 2327,6 | 1335,91 | 1566,68 | 1473,29 | 1684,32 | 1767,08 | 1276,98 | 1768,15375 |
| LPE 18:3 | 618 | 829,92 | 338,28 | 542,75 | 332,68 | 362,71 | 410,67 | 433,87 | 483,61 |
| LPE 20:4 | 187,48 | 143,77 | 218,15 | 191,65 | 127,71 | 219,16 | 177,01 | 344,51 | 201,18 |
| **Lysophosphatidylinositols** |  |  |  |  |  |  |  |  |  |
| LPI 18:0 | 3866,01 | 2233,98 | 894,12 | 1990,84 | 942,61 | 2367,66 | 3618,94 | 2136,67 | 2256,35375 |
| LPI 18:1 | 3605,93 | 1839,14 | 750,69 | 1659,51 | 1441,19 | 2429,91 | 2956,09 | 1454,87 | 2017,16625 |
| LPI 18:2 | 423,82 | 429,2 | 219,92 | 360,95 | 256,19 | 290,09 | 394,78 | 500,6 | 359,44375 |
| **Monoacylglycerols** |  |  |  |  |  |  |  |  |  |
| MG 15:0 | 609,37 | 1606,83 | 416,76 | 863,73 | 616,25 | 1092,7 | 669,09 | 496,84 | 796,44625 |
| MG 17:3 | 967,99 | 7412,67 | 1999,39 | 1339,58 | 3942,17 | 994,36 | 1646,44 | 3159,36 | 2682,745 |
| MG 17:4 | 1389,85 | 7385,07 | 4012,58 | 2157,02 | 2035,14 | 1494,85 | 1571,63 | 4985,2 | 3128,9175 |
| MG 18:0 | 1928,37 | 9069,44 | 4291,03 | 4045,53 | 4276,29 | 1985,28 | 2338,4 | 5385,6 | 4164,9925 |
| MG 19:3 | 363,15 | 2086,97 | 641,26 | 571,59 | 1171,94 | 381,73 | 460,99 | 1083,6 | 845,15375 |
| MG 21:4 | 1627,68 | 1698,53 | 1413,65 | 2439,15 | 1737,87 | 1717,78 | 1955,42 | 982,91 | 1696,62375 |
| MG 22:1 | 658,12 | 1627,11 | 1222,66 | 1174,93 | 1121,58 | 713,41 | 558,82 | 1093,51 | 1021,2675 |
| MG 24:2 | 360,66 | 530,01 | 497,58 | 742,32 | 394,39 | 405,45 | 290,25 | 473,71 | 461,79625 |
| MG 30:7 | 7610,83 | 11513,8 | 6518,78 | 11141,49 | 6529,02 | 5221,33 | 4742,95 | 3355,41 | 7079,2025 |
| MG 32:8 | 860,92 | 1297,87 | 751,24 | 1413,41 | 1004,15 | 931,97 | 1048,41 | 806,58 | 1014,31875 |
| MG 34:8 | 1767,87 | 4273,03 | 2273,81 | 3450,21 | 2561,86 | 2553,34 | 2309,13 | 1659,66 | 2606,11375 |
| MG 36:6 | 1120,58 | 588,49 | 483,14 | 1207,29 | 809,24 | 1210,31 | 1162,33 | 580,29 | 895,20875 |
| **N-acyl ethanolamines** |  |  |  |  |  |  |  |  |  |
| NAE 12:0 | 3799,86 | 5586,95 | 3785,66 | 4668,03 | 4747,99 | 3296,3 | 3169,13 | 5215,81 | 4283,71625 |
| NAE 13:1 | 4305,58 | 12896,5 | 5614,28 | 5446,06 | 7040,78 | 3368,41 | 3476,85 | 9755,59 | 6488,01 |
| NAE 14:1 | 7299,45 | 18341,2 | 11721,84 | 8290,1 | 12123,52 | 6322,18 | 6305,73 | 16158,66 | 10820,34 |
| NAE 15:1 | 1116,73 | 2624,34 | 1805,68 | 1133,39 | 1274,66 | 866,42 | 976,11 | 2215,43 | 1501,595 |
| NAE 16:1 | 336340,8 | 637642 | 517830,4 | 297425,4 | 328128,55 | 254827 | 241555 | 545348,6 | 394887,156 |
| NAE 16:2 | 751956,5 | 1561132 | 1294462 | 726182,55 | 721195,45 | 572046 | 537948 | 1419607,5 | 948066,213 |
| NAE 16:3 | 459985,7 | 836058 | 647246,5 | 525589,2 | 597818,35 | 405633 | 405865 | 719944,1 | 574767,569 |

| NAE 16:4 | 26240,46 | 66700,5 | 47341,9 | 31117,19 | 31062,08 | 21080,2 | 21039 | 59766,5 | 38043,4825 |
| --- | --- | --- | --- | --- | --- | --- | --- | --- | --- |
| NAE 17:4 | 3721,51 | 7679,25 | 5494,27 | 4866,53 | 6283,41 | 3345,11 | 4273,17 | 5127,43 | 5098,835 |
| NAE 18:1 | 13618,94 | 21477,5 | 20572,86 | 11959,3 | 10128,25 | 9770,02 | 9217,56 | 17505,17 | 14281,205 |
| NAE 18:2 | 19937,3 | 33249,7 | 30387,46 | 15708 | 15704,51 | 13145,9 | 12732,5 | 30479,9 | 21418,1563 |
| NAE 18:3 | 2862,28 | 5244,67 | 3873,01 | 3101,33 | 4197,09 | 2564,72 | 2740,56 | 4479,53 | 3632,89875 |
| NAE 18:4 | 78279,5 | 162057 | 129146,9 | 83363,56 | 88448,73 | 59984,1 | 67464,4 | 147273,3 | 102002,169 |
| NAE 18:5 | 121249,6 | 287578 | 209362,75 | 157199,78 | 196044,2 | 120572 | 119822 | 275835,95 | 185958,015 |
| NAE 20:1 | 1430,18 | 4643,3 | 2420,19 | 1958,29 | 2155,48 | 1751,61 | 1280,33 | 3086,16 | 2340,6925 |
| NAE 20:2 | 4514,74 | 6836,63 | 5882,38 | 4551,44 | 3738,09 | 4022,97 | 3359,04 | 5241,28 | 4768,32125 |
| NAE 20:4 | 3054,38 | 6314,06 | 6096,65 | 4121,47 | 3885,75 | 2854,76 | 3213,12 | 6102,54 | 4455,34125 |
| NAE 20:5 | 2915,86 | 5890,17 | 4648,36 | 3086,67 | 3067 | 2198,5 | 2495,02 | 4782,75 | 3635,54125 |
| NAE 22:3 | 1651,1 | 2196,81 | 1722,1 | 2160,27 | 2327,02 | 1664,72 | 1823,33 | 1921,17 | 1933,315 |
| NAE 22:4 | 1037,97 | 2247,05 | 1164,19 | 1038,24 | 710,11 | 847,18 | 970,74 | 1037,75 | 1131,65375 |
| NAE 22:5 | 1084,22 | 2350,77 | 1632,94 | 1294,29 | 1240,5 | 998 | 781,49 | 1714,09 | 1387,0375 |
| NAE 24:4 | 917,19 | 2092,32 | 1389,42 | 1136,89 | 1564,22 | 898,33 | 776,52 | 1878,4 | 1331,66125 |
| NAE 5:0 | 4123,34 | 18620,8 | 2798,53 | 2857,61 | 9497,78 | 2442,4 | 2781,64 | 7440,2 | 6320,28375 |
| NAE 6:0 | 18258,81 | 77926,8 | 15005,56 | 18821,66 | 89732,25 | 10971,6 | 13627,2 | 22493,82 | 33354,6963 |
| NAE 7:0 | 297,61 | 797,96 | 299,13 | 393 | 1210,36 | 310,48 | 372,37 | 818,17 | 562,385 |
| **Phosphatidylcholines** |  |  |  |  |  |  |  |  |  |
| PC 24:0 | 616,51 | 397,76 | 154,98 | 367,04 | 399,01 | 336,63 | 1482,59 | 555,37 | 538,73625 |
| PC 26:0|PC 12:0_14:0 | 2435,77 | 1718,04 | 659,24 | 2041,24 | 1563,05 | 1284,66 | 4052,26 | 1222,63 | 1872,11125 |
| PC 27:0|PC 12:0_15:0 | 416,99 | 414,38 | 193,75 | 364,41 | 347,22 | 307,44 | 754,73 | 278,31 | 384,65375 |
| PC 28:0 | 798,48 | 769,74 | 438,73 | 560,94 | 648,15 | 558,45 | 1188,01 | 559,55 | 690,25625 |
| PC 28:0|PC 14:0_14:0 | 48547,63 | 26333,7 | 11872,28 | 25913,8 | 25893,97 | 29567,4 | 90780,3 | 18314,28 | 34652,9225 |
| PC 28:1 | 474,02 | 555,75 | 177,07 | 397,98 | 484,48 | 391,12 | 687,4 | 414,52 | 447,7925 |
| PC 29:0|PC 14:0_15:0 | 8784,18 | 5636,53 | 2939,38 | 4228,81 | 4270,82 | 5380,95 | 10232,3 | 3572,77 | 5630,7125 |
| PC 30:0|PC 14:0_16:0 | 637877,3 | 452446 | 266114,7 | 438690,5 | 426797,9 | 511875 | 688948 | 308500,15 | 466406,088 |
| PC 30:1|PC 14:0_16:1 | 7662,04 | 4161,5 | 2298,72 | 5811,97 | 6047,67 | 6444,56 | 8822,79 | 2944,39 | 5524,205 |
| PC 30:3 | 461,67 | 661,55 | 323,74 | 450,63 | 370,94 | 401,67 | 464,79 | 512,89 | 455,985 |
| PC 31:0|PC 15:0_16:0 | 31380,08 | 31319,7 | 21878,16 | 22801,29 | 22703,55 | 32577 | 24035,6 | 22797,65 | 26186,62 |
| PC 31:1 | 1830,37 | 1437,87 | 1163,4 | 1571,61 | 1256,3 | 1582,49 | 2201,72 | 953,94 | 1499,7125 |
| PC 31:1|PC 15:0_16:1 | 1652,33 | 1344,82 | 1026,01 | 1747,87 | 1739,5 | 1836,65 | 1565,01 | 912,69 | 1478,11 |
| PC 32:0 | 11832,12 | 7486,8 | 6105,78 | 9848,85 | 9330,47 | 14267,3 | 10475,4 | 6585,04 | 9491,4725 |
| PC 32:0|PC 16:0_16:0 | 538147,4 | 467529 | 325948,3 | 481194,2 | 484587,25 | 629331 | 482813 | 331929,65 | 467685,075 |
| PC 32:1 | 2085,28 | 1658,69 | 1328,48 | 2109,62 | 1977,36 | 2255,46 | 1536,18 | 1459,14 | 1801,27625 |
| PC 32:1|PC 14:0_18:1 | 176579,3 | 109986 | 92571,18 | 189793,7 | 168314,9 | 190712 | 142567 | 90510,63 | 145129,351 |
| PC 32:2|PC 16:1_16:1 | 10429,43 | 5920,83 | 3170,57 | 6174,32 | 5577,41 | 5700,4 | 12081,4 | 3668,24 | 6590,325 |
| PC 32:3|PC 14:0_18:3 | 964,93 | 896,55 | 335,6 | 751,81 | 782,08 | 636,76 | 1308,49 | 602,78 | 784,875 |
| PC 32:3|PC 14:1_18:2 | 15570,1 | 6114,95 | 4127,34 | 6358,72 | 6597,03 | 10107,8 | 14575,8 | 4840,44 | 8536,51875 |
| PC 33:0|PC 16:0_17:0 | 8154,23 | 11105,4 | 9666,74 | 9433,02 | 10163,54 | 9949,8 | 7574,32 | 9049,51 | 9387,07125 |
| PC 33:1|PC 15:0_18:1 | 26140,4 | 17601,7 | 17703,34 | 19540,48 | 19211,59 | 23364,2 | 21085 | 17180,35 | 20228,3825 |
| PC 33:2 | 1898,31 | 1664,94 | 1461,91 | 1817,97 | 1603,78 | 1868,47 | 2449,04 | 1089,71 | 1731,76625 |
| PC 34:0|PC 16:0_18:0 | 49707,61 | 44061,4 | 47869,39 | 37140,16 | 35882,22 | 64970,9 | 58803 | 40179,04 | 47326,7175 |
| PC 34:1 | 20324,69 | 14900,5 | 18176,87 | 22035,67 | 19578,85 | 21142,7 | 20801,8 | 18144,52 | 19388,195 |
| PC 34:1|PC 16:0_18:1 | 858778,2 | 806122 | 775022,8 | 1032064 | 922287,05 | 909585 | 853645 | 755330,4 | 864104,281 |
| PC 34:2 | 4111,93 | 3089,57 | 4254,44 | 3641,86 | 3674,17 | 5043,68 | 4348,87 | 3402,8 | 3945,915 |

| PC 34:2|PC 15:1_19:1 | 1110,76 | 1357,78 | 1049,82 | 1529,44 | 987,89 | 1069,32 | 1033,76 | 1177 | 1164,47125 |
| --- | --- | --- | --- | --- | --- | --- | --- | --- | --- |
| PC 34:2|PC 16:0_18:2 | 178713,1 | 172793 | 145450,15 | 182754,1 | 179187,25 | 174771 | 153998 | 110002,4 | 162208,588 |
| PC 34:3|PC 16:0_18:3 | 40385,09 | 19885,9 | 15184,58 | 36099,57 | 32417,6 | 34411,8 | 45794,3 | 11911,02 | 29511,2375 |
| PC 34:4|PC 17:2_17:2 | 361,09 | 355,17 | 231,94 | 602,09 | 498,63 | 597,42 | 556,79 | 258,68 | 432,72625 |
| PC 35:0|PC 17:0_18:0 | 1211,96 | 1921,16 | 1978,7 | 1743,73 | 1442,98 | 1710,11 | 1806,46 | 1651,43 | 1683,31625 |
| PC 35:1|PC 17:0_18:1 | 5345,28 | 7206,75 | 10485,95 | 7724,42 | 7272,67 | 8873,46 | 7966,62 | 9029,78 | 7988,11625 |
| PC 35:2|PC 17:1_18:1 | 5903,26 | 4203,23 | 4313,55 | 4451,93 | 3953,63 | 6388,29 | 5164,83 | 3706,21 | 4760,61625 |
| PC 35:3 | 1117,45 | 1104,28 | 874,33 | 1018,16 | 872,57 | 1335,44 | 981,9 | 619,41 | 990,4425 |
| PC 36:0|PC 18:0_18:0 | 905,72 | 1510,92 | 1687,74 | 1380,92 | 1061,78 | 1166,1 | 1523,88 | 1513,76 | 1343,8525 |
| PC 36:1 | 751,95 | 451,78 | 847,91 | 708,68 | 696,62 | 951,16 | 1113,88 | 668,91 | 773,86125 |
| PC 36:1|PC 18:0_18:1 | 112659,7 | 90570,9 | 135751,05 | 112907,7 | 99133,37 | 136517 | 175086 | 116231,3 | 122357,16 |
| PC 36:2 | 1293,02 | 1777,02 | 1889,21 | 1923,77 | 1782,76 | 1683,17 | 860,67 | 2175,09 | 1673,08875 |
| PC 36:2|PC 18:1_18:1 | 83793,08 | 116674 | 127632 | 113334,8 | 107927 | 122311 | 64383 | 169328,55 | 113172,966 |
| PC 36:3 | 1521,56 | 1353,02 | 1381,1 | 1438,14 | 1106,74 | 1383,25 | 1286,34 | 1200,25 | 1333,8 |
| PC 36:3|PC 18:1_18:2 | 83662,49 | 65327,3 | 56975,88 | 61783,94 | 56998,64 | 78841,2 | 69670,2 | 49060,54 | 65290,02 |
| PC 36:4 | 746,49 | 713,7 | 506,25 | 787,81 | 528,04 | 662,14 | 912,33 | 534,35 | 673,88875 |
| PC 36:4|PC 18:2_18:2 | 26792,69 | 19180,7 | 15328,01 | 19581,91 | 18222,63 | 27677,6 | 29241,4 | 12270,18 | 21036,8838 |
| PC 36:5|PC 16:0_20:5 | 5498,05 | 4082,04 | 3742,87 | 4745,57 | 3721,05 | 6362,61 | 3938,13 | 2316,29 | 4300,82625 |
| PC 36:5|PC 18:2_18:3 | 2065,2 | 1926,65 | 1074,24 | 1462,95 | 1496,74 | 1632,65 | 1961,82 | 1192,46 | 1601,58875 |
| PC 36:6 | 157,49 | 192,03 | 95,68 | 126,69 | 150,79 | 138 | 174,38 | 111,76 | 143,3525 |
| PC 37:1|PC 19:0_18:1 | 499,55 | 619,71 | 958,05 | 795,78 | 568,34 | 655,5 | 486,95 | 885,11 | 683,62375 |
| PC 37:2|PC 18:1_19:1 | 813,47 | 1185,25 | 1284,63 | 962,28 | 966,3 | 1006,86 | 883,16 | 1090,25 | 1024,025 |
| PC 38:1|PC 20:0_18:1 | 637,44 | 1353,98 | 1284,92 | 1323,14 | 1367,8 | 1370,5 | 1042,98 | 1534,2 | 1239,37 |
| PC 38:2 | 719,47 | 1033,34 | 1109,11 | 1051,13 | 863,68 | 748,74 | 894,52 | 1115,23 | 941,9025 |
| PC 38:2|PC 20:0_18:2 | 1653,97 | 1996,68 | 3594,12 | 2689,16 | 2375,5 | 3217,82 | 2300,93 | 2471,02 | 2537,4 |
| PC 38:3 | 912,7 | 1444,9 | 2884,64 | 1233,42 | 1401,43 | 1868,34 | 2808,26 | 1616,47 | 1771,27 |
| PC 38:3|PC 20:0_18:3 | 818,62 | 819,82 | 1150,02 | 1179,64 | 851,22 | 1126,81 | 1094,37 | 950,6 | 998,8875 |
| PC 38:4 | 1039,32 | 1600,83 | 3439,61 | 1862,04 | 1765,38 | 2065,97 | 1637,2 | 1928,9 | 1917,40625 |
| PC 38:4|PC 18:1_20:3 | 2270,43 | 1942,84 | 3065,13 | 2433,86 | 2058,84 | 3626,28 | 3149,83 | 2327,18 | 2609,29875 |
| PC 38:5|PC 16:0_22:5 | 5353,71 | 4867,73 | 9023,16 | 8285,6 | 8071,43 | 9694,57 | 7346,7 | 7349,85 | 7499,09375 |
| PC 38:6|PC 19:3_19:3 | 1773,99 | 1496,86 | 1827,59 | 1563,72 | 1590,98 | 2435,24 | 1790,51 | 1266,86 | 1718,21875 |
| PC 38:7 | 360,31 | 469,52 | 305,99 | 431,02 | 398,04 | 438,98 | 346,37 | 363,95 | 389,2725 |
| PC 40:3|PC 25:1_15:2 | 218,45 | 289,28 | 521,74 | 359,03 | 447,86 | 569,31 | 384,1 | 450,11 | 404,985 |
| PC 40:5|PC 18:0_22:5 | 372,69 | 876,99 | 1576,05 | 749,8 | 975,96 | 1205 | 1227,11 | 1331,29 | 1039,36125 |
| PC 40:6 | 1243,9 | 1152,67 | 1645,13 | 1535,76 | 1568,66 | 1597,78 | 1303,12 | 1383,3 | 1428,79 |
| PC O-29:1 | 635,75 | 573,98 | 315,46 | 485,52 | 440,77 | 424,47 | 437,14 | 428,84 | 467,74125 |
| PC O-31:0 | 4427,96 | 3943,75 | 5268,75 | 6915,16 | 5756,56 | 6603,21 | 5780,58 | 4041,12 | 5342,13625 |
| PC O-31:1 | 2244,43 | 1667,03 | 1589,44 | 1931,17 | 1694,41 | 2047,7 | 1718,71 | 1660,97 | 1819,2325 |
| PC O-31:6 | 157,66 | 186,74 | 13,01 | 160,35 | 212,54 | 104,85 | 102,1 | 87,32 | 128,07125 |
| PC O-33:2 | 1955,32 | 2694,6 | 2166,38 | 2670,8 | 2228,98 | 2185,7 | 2213,14 | 2880,84 | 2374,47 |
| PC O-33:3 | 1470,66 | 1644,25 | 1205,99 | 1959,11 | 1464,75 | 1330,46 | 1385,84 | 1308,12 | 1471,1475 |
| PC O-33:4 | 6371,47 | 2311,89 | 4317,69 | 5199,35 | 4988,78 | 8219,02 | 3853,61 | 3434,79 | 4837,075 |
| PC O-33:6 | 426,65 | 484,17 | 275 | 565,22 | 554,78 | 394,58 | 416,44 | 316,4 | 429,155 |
| PC O-35:6 | 1221,78 | 1228,27 | 661,97 | 1553,18 | 1174,94 | 1403,03 | 1300,15 | 813,81 | 1169,64125 |
| PC O-35:9 | 2491,75 | 1113,4 | 851,82 | 1105,87 | 1310,7 | 1878,44 | 2155,54 | 1011,5 | 1489,8775 |

| PC O-37:6 | 239,34 | 331,18 | 209,39 | 294,27 | 307,49 | 266,94 | 297,06 | 304,02 | 281,21125 |
| --- | --- | --- | --- | --- | --- | --- | --- | --- | --- |
| PC O-38:10 | 942,61 | 1225,09 | 1130,07 | 1034,92 | 786 | 1290,05 | 1077,57 | 680,13 | 1020,805 |
| PC O-39:0 | 562,83 | 1222,81 | 1178,06 | 1234,44 | 1277,95 | 853,12 | 352,74 | 1651,53 | 1041,685 |
| PC O-39:10 | 2685,61 | 2318,67 | 2742,87 | 3293,55 | 3060,74 | 2815,39 | 2606,81 | 2918,66 | 2805,2875 |
| **Phosphatidylethanolamines** |  |  |  |  |  |  |  |  |  |
| PE 28:0|PE 14:0_14:0 | 1513,88 | 1438,58 | 847,2 | 1818,21 | 1294,9 | 1169,35 | 1351,73 | 1124,56 | 1319,80125 |
| PE 30:0|PE 14:0_16:0 | 4668,52 | 8996,36 | 3735,63 | 4217,14 | 4847,41 | 4491,7 | 2819,52 | 3511,47 | 4660,96875 |
| PE 30:1|PE 12:0_18:1 | 1592,59 | 2146,36 | 897,92 | 2147,75 | 1989 | 1522,35 | 2217,86 | 1256,74 | 1721,32125 |
| PE 31:0|PE 15:0_16:0 | 862,04 | 1210,86 | 626,85 | 701,54 | 698,27 | 794,92 | 312,83 | 550,01 | 719,665 |
| PE 31:1 | 382,21 | 319,25 | 242,25 | 388,47 | 347,06 | 343,52 | 245,53 | 192,98 | 307,65875 |
| PE 31:1|PE 15:0_16:1 | 133,75 | 239,66 | 60,52 | 388,88 | 107,16 | 191,21 | 150,82 | 99,69 | 171,46125 |
| PE 32:0|PE 16:0_16:0 | 7991,93 | 14517,7 | 8323,64 | 8917,81 | 8653,7 | 9398,13 | 3293,07 | 7840,64 | 8617,075 |
| PE 32:1|PE 14:0_18:1 | 20954,98 | 37147,2 | 21607,6 | 44174,96 | 51247,01 | 31273,5 | 22832,3 | 21797,98 | 31379,4388 |
| PE 32:1|PE 16:0_16:1 | 6995,72 | 6936,42 | 3545,46 | 10352 | 10153,72 | 8682,71 | 6095,98 | 4427,6 | 7148,70125 |
| PE 32:2 | 525,98 | 656,76 | 335,58 | 675,76 | 490,14 | 573,31 | 663,69 | 404,8 | 540,7525 |
| PE 32:2|PE 14:0_18:2 | 3782,7 | 5037,89 | 2142,01 | 4876,67 | 4334,82 | 3823,38 | 5622,43 | 2976,4 | 4074,5375 |
| PE 32:3|PE 14:0_18:3 | 363,5 | 230,95 | 122,6 | 209,57 | 404,92 | 278,61 | 438,19 | 157,87 | 275,77625 |
| PE 33:0|PE 16:0_17:0 | 862,91 | 1240,54 | 841,85 | 822,09 | 1014,48 | 944,52 | 406,77 | 969,36 | 887,815 |
| PE 33:1|PE 15:0_18:1 | 4811,81 | 8695,06 | 5872,27 | 7809,62 | 9088,24 | 6495,66 | 4023,36 | 5411,45 | 6525,93375 |
| PE 34:0|PE 16:0_18:0 | 3026,69 | 4358,7 | 4487,14 | 2727,34 | 2886,54 | 3842,64 | 1604,76 | 3243,88 | 3272,21125 |
| PE 34:1 | 4014,05 | 5327,8 | 3423,17 | 6363,53 | 5395,61 | 4259,85 | 3482,07 | 4418,12 | 4585,525 |
| PE 34:1|PE 16:0_18:1 | 179803,8 | 299376 | 216349,85 | 315035,55 | 309199,65 | 213194 | 130528 | 242941,3 | 238303,538 |
| PE 34:2 | 1232,31 | 1361,26 | 698,29 | 1723,87 | 1652,53 | 1361,48 | 1094,21 | 1048,43 | 1271,5475 |
| PE 34:2|PE 16:0_18:2 | 85622,07 | 133908 | 74555,64 | 145777,9 | 155149,65 | 102497 | 89483,5 | 85120,99 | 109014,371 |
| PE 34:3|PE 16:0_18:3 | 9353,25 | 19129,2 | 7603,18 | 20894,6 | 21351,35 | 10524,1 | 12083 | 9105,34 | 13755,51 |
| PE 34:3|PE 16:1_18:2 | 2314,4 | 3880,4 | 1729,22 | 6208,45 | 7709,92 | 4542,23 | 2483,42 | 1826,31 | 3836,79375 |
| PE 34:4|PE 14:0_20:4 | 518,39 | 521,96 | 230,64 | 652,44 | 829,71 | 626,93 | 923,55 | 646,84 | 618,8075 |
| PE 34:4|PE 16:1_18:3 | 359,69 | 348,28 | 135,21 | 1281,87 | 1030,36 | 781,34 | 336,78 | 233 | 563,31625 |
| PE 35:0|PE 17:0_18:0 | 302,38 | 423,49 | 338,52 | 131,23 | 78,71 | 250,38 | 158,4 | 277,37 | 245,06 |
| PE 35:1|PE 17:0_18:1 | 9070,35 | 12217 | 12466,29 | 11553,66 | 11391,7 | 10402,9 | 8061,85 | 10669,56 | 10729,165 |
| PE 35:2|PE 17:1_18:1 | 6479,89 | 9405,38 | 8523,92 | 10595,1 | 11335,67 | 9843,63 | 6854,53 | 8882,04 | 8990,02 |
| PE 35:3|PE 17:1_18:2 | 836,29 | 824,25 | 620,68 | 1193,9 | 1234,98 | 1090,11 | 1043,52 | 843,22 | 960,86875 |
| PE 36:0|PE 18:0_18:0 | 361,87 | 156,61 | 391,13 | 212,5 | 169,87 | 375,07 | 423,53 | 420,12 | 313,8375 |
| PE 36:1|PE 18:0_18:1 | 120439,7 | 203736 | 189254,25 | 155857,85 | 159490,65 | 156883 | 141949 | 209799,2 | 167176,138 |
| PE 36:2 | 2130,47 | 1802,19 | 1588,86 | 2210,97 | 1974,85 | 1998,87 | 2794,64 | 1697,32 | 2024,77125 |
| PE 36:2|PE 18:1_18:1 | 359650,7 | 610930 | 489486,3 | 541293,9 | 550015,15 | 486196 | 347971 | 583631,45 | 496146,8 |
| PE 36:3 | 1811,57 | 2380,92 | 1080,15 | 2024,71 | 1776,46 | 1845,28 | 1775,05 | 1532,5 | 1778,33 |
| PE 36:3;2O|PE 14:0_22:3;2O | 957,71 | 1288,14 | 1058,37 | 822,19 | 962,27 | 1008,38 | 630,77 | 708,83 | 929,5825 |
| PE 36:3|PE 18:1_18:2 | 114982,7 | 192481 | 112348,95 | 154240,7 | 166922,4 | 141175 | 129686 | 132816,6 | 143081,681 |
| PE 36:4|PE 16:0_20:4 | 11900,68 | 19656,2 | 12222,71 | 21643,65 | 25070,87 | 14762,7 | 14838,2 | 15060,31 | 16894,4113 |
| PE 36:4|PE 18:1_18:3 | 26655,44 | 47632,5 | 23709,81 | 39288,3 | 41245,73 | 32922,5 | 35603,2 | 26343,31 | 34175,09 |
| PE 36:5|PE 16:0_20:5 | 3287,96 | 5662,16 | 2441,37 | 5169,1 | 5595,27 | 4046,68 | 2437,89 | 2530,74 | 3896,39625 |
| PE 36:5|PE 18:2_18:3 | 1472,77 | 1587,59 | 909,08 | 1931,27 | 2119,11 | 1881,65 | 2337,7 | 1050,33 | 1661,1875 |
| PE 37:1|PE 18:0_19:1 | 970,02 | 902,34 | 1419,02 | 796,07 | 684,55 | 1019,52 | 1214,3 | 836,16 | 980,2475 |
| PE 37:2|PE 18:1_19:1 | 3114,04 | 3105,68 | 3678,79 | 3234,9 | 3172,1 | 3334,58 | 2816,45 | 3918,4 | 3296,8675 |
| PE 37:3|PE 18:1_19:2 | 742,34 | 1043,31 | 600 | 892,63 | 1062,1 | 788,39 | 831,8 | 719,77 | 835,0425 |

| PE 38:1|PE 20:0_18:1 | 1616,65 | 1326,33 | 2425,25 | 1382,97 | 1655,99 | 1679,99 | 1928,61 | 1298,58 | 1664,29625 |
| --- | --- | --- | --- | --- | --- | --- | --- | --- | --- |
| PE 38:2|PE 18:1_20:1 | 3395,42 | 4850,85 | 5093,22 | 3674,84 | 4014,21 | 3961,59 | 3109,76 | 4621,56 | 4090,18125 |
| PE 38:3|PE 18:0_20:3 | 5977,36 | 7581,25 | 8004,42 | 7515,42 | 7673,47 | 8085,27 | 9969,5 | 9252,48 | 8007,39625 |
| PE 38:3|PE 18:1_20:2 | 1384,47 | 1913,61 | 2344,97 | 2156,1 | 1954,76 | 1892,51 | 1789,85 | 2228,01 | 1958,035 |
| PE 38:4|PE 18:0_20:4 | 6885,68 | 11433,9 | 14506,9 | 12135,25 | 14266,22 | 11142,7 | 13906,5 | 16162,36 | 12554,93 |
| PE 38:4|PE 18:1_20:3 | 6066,95 | 8635,58 | 8528,55 | 10081,54 | 10158,13 | 9683,76 | 8475,24 | 9909,83 | 8942,4475 |
| PE 38:5|PE 16:0_22:5 | 12971,79 | 21555,7 | 17901,91 | 25996,67 | 28904,32 | 20753,8 | 18539,4 | 23626,64 | 21281,2688 |
| PE 38:5|PE 18:1_20:4 | 5126,52 | 3350,13 | 2833,66 | 5106,11 | 4824,42 | 5018,24 | 4961,33 | 3694,02 | 4364,30375 |
| PE 38:6 | 1380,26 | 1344,36 | 859,66 | 1495,71 | 1324,83 | 1622,52 | 930,92 | 848,3 | 1225,82 |
| PE 39:1|PE 21:0_18:1 | 375,35 | 413,76 | 623,31 | 406,89 | 494,12 | 794,17 | 568,05 | 486,03 | 520,21 |
| PE 39:2|PE 18:1_21:1 | 580,95 | 977,1 | 840,9 | 626,71 | 491,32 | 702,02 | 541,62 | 397,66 | 644,785 |
| PE 40:1|PE 22:0_18:1 | 1455,14 | 921,21 | 1488,29 | 1080,17 | 1242,29 | 1733,02 | 1807,58 | 734,59 | 1307,78625 |
| PE 40:2|PE 18:1_22:1 | 484,74 | 334,29 | 317,38 | 249,4 | 330,04 | 609,36 | 575,37 | 346,77 | 405,91875 |
| PE 40:4|PE 18:0_22:4 | 552,2 | 580,89 | 1293,65 | 1548,04 | 1231,35 | 793,55 | 1675,27 | 1876,94 | 1193,98625 |
| PE 40:5|PE 18:0_22:5 | 6200,8 | 7566,86 | 11910,87 | 10866,84 | 11578,83 | 9230,03 | 13077,8 | 12376,71 | 10351,0863 |
| PE 40:6|PE 18:1_22:5 | 4021,56 | 2784,2 | 2664,89 | 3090,59 | 2815,18 | 3774,37 | 3168,68 | 3568,96 | 3236,05375 |
| PE 41:1|PE 23:0_18:1 | 459,58 | 427,49 | 424,17 | 112,88 | 77,29 | 624,44 | 424,49 | 98,51 | 331,10625 |
| PE 44:10|PE 22:5_22:5 | 1403,77 | 2008,03 | 2416,77 | 3421,48 | 3669,83 | 2746,39 | 2081,04 | 2599,47 | 2543,3475 |
| PE O-19:0_18:1 | 540,57 | 372,76 | 89,13 | 407,84 | 310,92 | 512,02 | 233,05 | 238,44 | 338,09125 |
| PE O-30:1|PE O-14:1_16:0 | 330,86 | 311,85 | 219,79 | 67,56 | 236,11 | 312,24 | 86,63 | 226,63 | 223,95875 |
| PE O-31:1|PE O-15:1_16:0 | 1423,48 | 2020,6 | 958,54 | 1351,73 | 872,74 | 1331,49 | 408,86 | 1242,43 | 1201,23375 |
| PE O-31:2|PE O-15:1_16:1 | 559,22 | 582,6 | 438,1 | 733,93 | 1113,55 | 643,47 | 476,92 | 407,3 | 619,38625 |
| PE O-32:1|PE O-16:1_16:0 | 297,2 | 660,54 | 648,13 | 228,44 | 254,15 | 411,73 | 179,34 | 276,65 | 369,5225 |
| PE O-32:2|PE O-14:1_18:1 | 2389,21 | 3403,73 | 3170,91 | 4039,66 | 4238,09 | 3306,58 | 2806,85 | 3825,7 | 3397,59125 |
| PE O-32:3|PE O-14:1_18:2 | 1447,63 | 1861,12 | 1430,64 | 1191,32 | 1325,82 | 1030,15 | 1138,63 | 731,5 | 1269,60125 |
| PE O-33:2|PE O-15:1_18:1 | 5752,65 | 6038,53 | 6940,28 | 6629,45 | 7189,84 | 5678,82 | 5398,04 | 9159,95 | 6598,445 |
| PE O-33:3|PE O-15:1_18:2 | 4221,53 | 5712,01 | 3470,4 | 3758,98 | 4408,22 | 3423,09 | 3297,88 | 3245,51 | 3942,2025 |
| PE O-33:4|PE O-15:1_18:3 | 653,96 | 961,17 | 490,6 | 834,73 | 959,75 | 482,78 | 634,96 | 312,26 | 666,27625 |
| PE O-34:2|PE O-16:1_18:1 | 14411,85 | 20808,1 | 26486,52 | 20267,75 | 20764,37 | 19079,8 | 16434,7 | 26429,14 | 20585,2775 |
| PE O-34:3|PE O-16:1_18:2 | 10014,05 | 15399,4 | 14019,29 | 11315,96 | 11809,7 | 12786,4 | 11568,5 | 11381,31 | 12286,8225 |
| PE O-34:4|PE O-16:1_18:3 | 2399,48 | 2799,83 | 2251,84 | 3213,57 | 3382,18 | 2793,98 | 2984,21 | 1411,42 | 2654,56375 |
| PE O-35:1 | 3689,95 | 5557,01 | 4004,95 | 6066,1 | 4924,67 | 5317,45 | 4724,55 | 4269,28 | 4819,245 |
| PE O-35:2|PE O-17:1_18:1 | 1747,63 | 2624,24 | 3123,88 | 2669,73 | 2725,58 | 2501,33 | 2179,21 | 2671,29 | 2530,36125 |
| PE O-35:3|PE O-17:1_18:2 | 1238,19 | 1786,38 | 1964,22 | 1843,59 | 1680,74 | 1664,16 | 1578,8 | 1292,71 | 1631,09875 |
| PE O-35:3|PE O-17:2_18:1 | 458,94 | 356,3 | 830,56 | 478,05 | 578,8 | 438,53 | 423,99 | 512,56 | 509,71625 |
| PE O-35:5|PE O-15:1_20:4 | 723,86 | 1088,74 | 940,08 | 554,79 | 816,36 | 722,43 | 548,94 | 1138,73 | 816,74125 |
| PE O-35:6|PE O-15:1_20:5 | 410,42 | 247,55 | 224,17 | 240,86 | 162,92 | 417,87 | 227,37 | 230,59 | 270,21875 |
| PE O-36:2|PE O-18:0_18:2 | 338,58 | 821,51 | 1991,85 | 335,5 | 1960,01 | 942,3 | 1784,93 | 851,32 | 1128,25 |
| PE O-36:2|PE O-18:1_18:1 | 4894,73 | 5810,21 | 6685,1 | 4662,39 | 4200 | 5450,64 | 3667,82 | 6357,32 | 5216,02625 |
| PE O-36:3|PE O-18:1_18:2 | 383,63 | 457,57 | 1128,07 | 507,82 | 467,41 | 841,99 | 1418,33 | 678,98 | 735,475 |
| PE O-36:3|PE O-18:2_18:1 | 6529,21 | 8585,63 | 6563,9 | 5023,63 | 5532,6 | 6664,76 | 5463,07 | 6051,54 | 6301,7925 |
| PE O-36:4|PE O-18:2_18:2 | 2783,78 | 3870,87 | 3639,44 | 2681,53 | 2650,92 | 3294,03 | 2882,12 | 2936,95 | 3092,455 |
| PE O-36:5|PE O-16:1_20:4 | 1480,79 | 2020,85 | 4669,25 | 2191,02 | 2485,66 | 3026,21 | 2202,7 | 2963,68 | 2630,02 |
| PE O-36:5|PE O-18:2_18:3 | 581,55 | 807,48 | 494,85 | 375,5 | 558,9 | 551,54 | 538,68 | 341,01 | 531,18875 |
| PE O-36:6|PE O-16:1_20:5 | 990,72 | 471,06 | 1805,23 | 589,53 | 776,25 | 1586,71 | 655,81 | 732,81 | 951,015 |

| PE O-38:4|PE O-18:1_20:3 | 501,25 | 361,54 | 729,85 | 272,19 | 517,32 | 571,6 | 514,57 | 605,12 | 509,18 |
| --- | --- | --- | --- | --- | --- | --- | --- | --- | --- |
| PE O-38:5|PE O-18:1_20:4 | 704,44 | 865,49 | 2251,13 | 532,96 | 1099,58 | 1482,38 | 1187,29 | 1323,32 | 1180,82375 |
| PE O-38:6|PE O-16:1_22:5 | 1395,53 | 1516,37 | 5583,64 | 2052,48 | 1909,54 | 2487,03 | 2231,8 | 3136,5 | 2539,11125 |
| PE O-40:6|PE O-18:1_22:5 | 526,99 | 626,72 | 1931,45 | 357,42 | 620,07 | 982,68 | 842,22 | 439,13 | 790,835 |
| PE P-30:1|PE P-14:0_16:1 | 319,74 | 343,02 | 304,56 | 443,7 | 418,82 | 328,91 | 314,87 | 258,47 | 341,51125 |
| PE P-31:0|PE P-15:0_16:0 | 653,42 | 965,65 | 538,02 | 716,92 | 613,06 | 634,87 | 295,09 | 684,88 | 637,73875 |
| PE P-32:1|PE P-14:0_18:1 | 1432,12 | 1831,56 | 1234,76 | 2324,93 | 1831,64 | 1717,13 | 1193,89 | 1246,18 | 1601,52625 |
| PE P-32:2|PE P-14:0_18:2 | 747,73 | 654,59 | 484,44 | 628,13 | 562,21 | 617,03 | 710,01 | 456,42 | 607,57 |
| PE P-33:1|PE P-15:0_18:1 | 1344,41 | 1388,59 | 1294,15 | 2056,86 | 1476,74 | 1454,46 | 1346,34 | 1795,41 | 1519,62 |
| PE P-33:2|PE P-15:0_18:2 | 1269,3 | 1284,34 | 675,75 | 1063,58 | 809,31 | 813,21 | 811,18 | 695,28 | 927,74375 |
| PE P-33:3|PE P-15:0_18:3 | 893,32 | 488,27 | 411,96 | 604,22 | 379,48 | 636,43 | 618,9 | 262,66 | 536,905 |
| PE P-34:1|PE P-16:0_18:1 | 5911,6 | 5732,08 | 6459,92 | 7429,95 | 6056,74 | 7024,29 | 6020,72 | 5523,81 | 6269,88875 |
| PE P-34:2|PE P-16:0_18:2 | 3288,4 | 2970,6 | 2288,4 | 2918,74 | 2659,07 | 3261,39 | 2872,74 | 1911,19 | 2771,31625 |
| PE P-34:3|PE P-16:0_18:3 | 849,77 | 830,26 | 540,08 | 834,23 | 785,95 | 721,11 | 759,45 | 573,19 | 736,755 |
| PE P-36:1|PE P-18:0_18:1 | 3296,97 | 5936,21 | 4330,48 | 5959,83 | 5081,07 | 4798,26 | 3862,37 | 4018,49 | 4660,46 |
| PE P-36:2|PE P-18:0_18:2 | 1631,96 | 2117,45 | 1470,74 | 2062,77 | 2053,84 | 2258,74 | 1501,33 | 1530,57 | 1828,425 |
| PE P-36:2|PE P-18:1_18:1 | 1490,86 | 1677,69 | 1081,44 | 1648,49 | 1485,17 | 1565,98 | 1124,55 | 1449,96 | 1440,5175 |
| PE P-36:3|PE P-16:0_20:3 | 705,77 | 1107,82 | 690,53 | 873,73 | 816,79 | 984,97 | 986,04 | 668,62 | 854,28375 |
| PE P-36:3|PE P-18:1_18:2 | 607,56 | 1146,84 | 772,95 | 837,98 | 849,35 | 1073,44 | 965,66 | 784,77 | 879,81875 |
| PE P-36:4|PE P-16:0_20:4 | 691,13 | 730,46 | 1006,98 | 570,14 | 574,91 | 886,76 | 628,25 | 780,78 | 733,67625 |
| **Phosphatidylinositols** |  |  |  |  |  |  |  |  |  |
| PI 30:0 | 1105,11 | 522,73 | 696,91 | 1178,02 | 777,37 | 1146,8 | 1213,71 | 623,81 | 908,0575 |
| PI 30:0|PI 14:0_16:0 | 1218,9 | 520,55 | 683,91 | 1217,29 | 792,8 | 1168,69 | 1269,31 | 695,44 | 945,86125 |
| PI 32:0|PI 16:0_16:0 | 5793,8 | 6329,39 | 4798,44 | 9284,08 | 7460,37 | 6816,59 | 4870,02 | 5622,69 | 6371,9225 |
| PI 32:1|PI 14:0_18:1 | 3715,45 | 2708,5 | 2539,34 | 4194,75 | 3322,77 | 5036,95 | 2864,15 | 2429,73 | 3351,455 |
| PI 33:1 | 882,43 | 428,38 | 630,37 | 864,9 | 433,63 | 1071,99 | 527,89 | 423,65 | 657,905 |
| PI 34:0|PI 16:0_18:0 | 4486,75 | 5588,63 | 5681,17 | 8297,89 | 7194,67 | 5884,5 | 5609,51 | 6630,01 | 6171,64125 |
| PI 34:1|PI 16:0_18:1 | 36455,49 | 39497,5 | 29539,86 | 46159,54 | 34105,92 | 39485,3 | 25464,7 | 32341,19 | 35381,1775 |
| PI 34:2|PI 16:1_18:1 | 8415,28 | 8703,93 | 5588,2 | 8681,58 | 6093,29 | 8941,37 | 6312,22 | 6259,4 | 7374,40875 |
| PI 34:3 | 504,29 | 243,07 | 229,9 | 448,07 | 344,69 | 561,04 | 503,47 | 272,68 | 388,40125 |
| PI 35:2 | 1248,15 | 435,39 | 683,41 | 723,65 | 458,77 | 1183,67 | 545,22 | 388,15 | 708,30125 |
| PI 36:0 | 749,59 | 972,42 | 787,84 | 1332,3 | 1212,45 | 1186,87 | 1121,32 | 990,84 | 1044,20375 |
| PI 36:1|PI 18:0_18:1 | 109408,9 | 101299 | 85558,7 | 108012,45 | 87321,62 | 96381,5 | 108625 | 100506,01 | 99639,1425 |
| PI 36:2|PI 18:1_18:1 | 65886,17 | 71871 | 46459,39 | 55099,41 | 44661,58 | 53959 | 55173,5 | 51943,34 | 55631,6675 |
| PI 36:3|PI 18:1_18:2 | 16092,3 | 13650,6 | 9282,97 | 10785,31 | 8013,13 | 12378,1 | 10531,7 | 7991,29 | 11090,675 |
| PI 36:4|PI 16:0_20:4 | 3290,76 | 2681,5 | 2879,76 | 2775,74 | 2185,41 | 2918,11 | 1779,31 | 3218,43 | 2716,1275 |
| PI 36:4|PI 18:2_18:2 | 1378,93 | 896,76 | 468,24 | 1052,74 | 706,18 | 1350,27 | 1240,82 | 456,06 | 943,75 |
| PI 36:5 | 1589,37 | 1108,61 | 991,73 | 1141,8 | 1093,63 | 1523,2 | 953,23 | 657,76 | 1132,41625 |
| PI 37:1 | 1815,59 | 1338,13 | 1290,68 | 1483,41 | 894,68 | 1340,99 | 1754,24 | 973,12 | 1361,355 |
| PI 37:2 | 1433,37 | 1390,68 | 1271,77 | 1225,61 | 703,52 | 1360,81 | 1127,7 | 1080,79 | 1199,28125 |
| PI 38:1 | 1804,08 | 1772,68 | 1293,43 | 1568,09 | 1219,48 | 1556,74 | 1612,28 | 1192,67 | 1502,43125 |
| PI 38:2 | 4505,37 | 3991,7 | 3122,46 | 3821,6 | 2879,59 | 3874,26 | 3328,88 | 3084,01 | 3575,98375 |
| PI 38:3|PI 18:0_20:3 | 14047,46 | 11513,1 | 11063,29 | 7482,15 | 6647,29 | 11492,1 | 10950,8 | 12007,8 | 10650,495 |
| PI 38:4|PI 18:0_20:4 | 15399,91 | 16049,3 | 18729,48 | 11800,61 | 10102,94 | 13759,8 | 11492,3 | 17701,26 | 14379,445 |
| PI 38:4|PI 18:1_20:3 | 5603,07 | 4516,53 | 4739,56 | 3361,04 | 3170,54 | 6031,03 | 3243,01 | 3992,83 | 4332,20125 |

| PI 38:5|PI 18:0_20:5 | 10464,1 | 9818,78 | 8081,93 | 5564,22 | 5991,86 | 8769,43 | 6077,87 | 6861,15 | 7703,6675 |
| --- | --- | --- | --- | --- | --- | --- | --- | --- | --- |
| PI 38:5|PI 18:1_20:4 | 7933,87 | 6465,46 | 7186,96 | 5587,25 | 4686,42 | 8225,48 | 3444,94 | 6786,3 | 6289,585 |
| PI 38:6 | 2355,24 | 1732,16 | 1114,27 | 1274,64 | 986,66 | 2399,4 | 805,46 | 999,47 | 1458,4125 |
| PI 40:3 | 6937,99 | 7001,17 | 10311,11 | 10666,55 | 12888,71 | 9946,92 | 9547,24 | 7899,73 | 9399,9275 |
| PI 40:6 | 440,43 | 397,83 | 285,69 | 220,09 | 214,91 | 483,48 | 283,83 | 224,86 | 318,89 |
| PI 42:10 | 3260,83 | 3245,74 | 1997,87 | 2491,91 | 2193,44 | 2712,28 | 2833,45 | 2379,09 | 2639,32625 |
| **Phosphatidylserines** |  |  |  |  |  |  |  |  |  |
| PS 32:0 | 508,31 | 597,08 | 384,93 | 454,46 | 365,26 | 539,98 | 340,26 | 377,7 | 445,9975 |
| PS 32:1|PS 16:0_16:1 | 1054,11 | 456,35 | 342,97 | 1628,62 | 1229,05 | 1169,13 | 354,54 | 582 | 852,09625 |
| PS 34:1|PS 16:0_18:1 | 14832,17 | 24657,3 | 16214,45 | 24309,47 | 20939,86 | 15074,5 | 7096,69 | 18191,95 | 17664,5525 |
| PS 34:2|PS 16:0_18:2 | 6509,04 | 7370,41 | 4803,46 | 8275,13 | 6363,31 | 5304,86 | 3481,95 | 3830,27 | 5742,30375 |
| PS 34:3|PS 16:0_18:3 | 740,25 | 627,87 | 234,05 | 767,76 | 505,81 | 458,51 | 275,33 | 154,8 | 470,5475 |
| PS 35:1|PS 18:0_17:1 | 4587,48 | 5153,27 | 4408,04 | 3505,11 | 2835,54 | 5129,28 | 2589,82 | 3748,62 | 3994,645 |
| PS 35:2|PS 17:0_18:2 | 441,53 | 440,42 | 450,81 | 460,12 | 396,98 | 517,34 | 457,2 | 414,29 | 447,33625 |
| PS 36:0|PS 18:0_18:0 | 20638,55 | 24199,7 | 22742,9 | 19796,16 | 17588,61 | 20071,2 | 16365 | 25911,54 | 20914,1988 |
| PS 36:1|PS 18:0_18:1 | 139246,9 | 169939 | 166665,45 | 137039,85 | 107155,95 | 144092 | 108781 | 189479,9 | 145300,031 |
| PS 36:2|PS 18:1_18:1 | 97929,85 | 113449 | 96289,14 | 77351,7 | 63663,2 | 89291,8 | 83981,6 | 91947,47 | 89237,995 |
| PS 36:3|PS 18:0_18:3 | 5017,91 | 2865,8 | 2996,52 | 3403,93 | 2504,94 | 3868,9 | 4178,16 | 2157,75 | 3374,23875 |
| PS 36:3|PS 18:1_18:2 | 21032,36 | 21735,2 | 15414,6 | 19156,53 | 13812,2 | 17035,5 | 18933,1 | 13452,54 | 17571,505 |
| PS 36:4|PS 18:1_18:3 | 1962,44 | 1676 | 788,38 | 1325,12 | 1008,27 | 1443,05 | 1771,03 | 813,88 | 1348,52125 |
| PS 36:4|PS 18:2_18:2 | 347,5 | 353,84 | 215,16 | 314,21 | 249,97 | 317,43 | 322 | 221,34 | 292,68125 |
| PS 37:0 | 81,03 | 128,48 | 110,71 | 99,76 | 94,39 | 110,07 | 109,92 | 95,55 | 103,73875 |
| PS 37:2 | 241,76 | 191,53 | 249,55 | 240,49 | 203,41 | 188,94 | 223,15 | 230,37 | 221,15 |
| PS 38:1|PS 20:0_18:1 | 5292,5 | 5955,35 | 7089,83 | 4315,67 | 3118,56 | 5392,47 | 3527,04 | 5614,56 | 5038,2475 |
| PS 38:2|PS 18:1_20:1 | 3979,23 | 4503,9 | 4250,42 | 3105,07 | 2031,56 | 3218,7 | 2468,65 | 3430,96 | 3373,56125 |
| PS 38:3 | 19212,16 | 13746 | 17464,38 | 26706,73 | 27254,32 | 26824,6 | 21417,7 | 11914,8 | 20567,5838 |
| PS 38:3|PS 18:0_20:3 | 12607,94 | 12311,8 | 12303,7 | 10930,82 | 9049,49 | 14366,4 | 11501,6 | 13900,62 | 12121,5463 |
| PS 38:4|PS 18:1_20:3 | 2501,21 | 3295,55 | 2256,05 | 2424,02 | 1749,78 | 2903,97 | 2379,95 | 2380,49 | 2486,3775 |
| PS 38:5|PS 16:0_22:5 | 1591,79 | 1163,57 | 1318,87 | 2349,94 | 1727,86 | 1397,62 | 909,56 | 925,47 | 1423,085 |
| PS 39:1|PS 21:0_18:1 | 3126,73 | 4097,01 | 4813,49 | 3307,35 | 2399,95 | 4209,59 | 2960,12 | 3916 | 3603,78 |
| PS 39:2|PS 18:1_21:1 | 2827,34 | 3343,95 | 3350,19 | 2697,34 | 1739,46 | 3005,99 | 1874,59 | 3153,92 | 2749,0975 |
| PS 40:1|PS 22:0_18:1 | 5279,51 | 5132,48 | 6457,62 | 3622,53 | 2961,71 | 5215 | 2951,7 | 5391,35 | 4626,4875 |
| PS 40:2 | 2578,17 | 2513,19 | 2618,96 | 1730,81 | 1176,34 | 2469,71 | 1586,83 | 2131,47 | 2100,685 |
| PS 40:2|PS 22:0_18:2 | 2702,03 | 3000,96 | 2454,82 | 1646,95 | 1204,6 | 2263,77 | 1426,22 | 1908,82 | 2076,02125 |
| PS 40:3 | 1978,7 | 764,56 | 1006,12 | 1401,31 | 1285,67 | 1707,19 | 1834,73 | 855,48 | 1354,22 |
| PS 40:4|PS 18:0_22:4 | 2071,45 | 2446,93 | 3478,38 | 3106,01 | 2924,37 | 1664,57 | 3007,3 | 4832,78 | 2941,47375 |
| PS 40:5|PS 18:0_22:5 | 22561,6 | 23050,8 | 27367,89 | 26330,54 | 22634 | 23862,3 | 24639,6 | 35475,46 | 25740,2663 |
| PS 40:6|PS 18:1_22:5 | 4038,22 | 3459,17 | 3371,83 | 3174,71 | 2595,77 | 4019,69 | 3812,95 | 4298,42 | 3596,345 |
| PS 41:2 | 1003,22 | 1121,22 | 859,09 | 640,92 | 685 | 1147,32 | 786,28 | 637,93 | 860,1225 |
| PS 42:1|PS 24:0_18:1 | 949,27 | 799,77 | 691,78 | 714,36 | 481,86 | 852,91 | 451,21 | 893,18 | 729,2925 |
| **Sulfatides** |  |  |  |  |  |  |  |  |  |
| SHexCer 35:0;3O | 1886,23 | 4844,18 | 3630,24 | 2374,86 | 2117,53 | 3150,65 | 1509,9 | 3132,83 | 2830,8025 |
| SHexCer 36:4;2O | 1989,99 | 6755,51 | 4347,74 | 4122,71 | 3994,15 | 3689,7 | 1725,48 | 5238,4 | 3982,96 |
| SHexCer 38:2;2O | 184,67 | 182,65 | 218,28 | 285,52 | 369,31 | 474,66 | 443 | 150,07 | 288,52 |
| SHexCer 38:7;3O | 259,94 | 461,82 | 476,39 | 413,4 | 340,38 | 370,47 | 258,34 | 156,78 | 342,19 |
| SHexCer 39:5;3O | 894,41 | 2112,78 | 2186,38 | 1781,99 | 1576,33 | 1382,14 | 687,65 | 2399,21 | 1627,61125 |

| SHexCer 39:6;3O | 461,86 | 778,3 | 648,06 | 453,52 | 489,22 | 575,89 | 307,72 | 444,97 | 519,9425 |
| --- | --- | --- | --- | --- | --- | --- | --- | --- | --- |
| SHexCer 39:7;3O | 4842,99 | 11299,1 | 8577,48 | 12226,85 | 9918,28 | 7448,35 | 3415,87 | 10310,9 | 8504,98125 |
| SHexCer 39:8;3O | 2363,87 | 4439,83 | 2859,63 | 4128,39 | 4133,79 | 3028,03 | 1779,42 | 3150,14 | 3235,3875 |
| SHexCer 40:2;2O | 1370,62 | 5290,58 | 4437,34 | 7366,71 | 6185,28 | 5154,27 | 3115,8 | 3543,39 | 4557,99875 |
| SHexCer 40:3;3O | 1099,52 | 2094,75 | 2145,57 | 1630,05 | 1466,13 | 2053,26 | 1240,4 | 2119,2 | 1731,11 |
| SHexCer 40:8;3O | 221,49 | 416,17 | 478,66 | 299,61 | 271,78 | 350,34 | 370,11 | 441,79 | 356,24375 |
| SHexCer 40:9;3O | 629,78 | 1541,58 | 1004,66 | 1019,59 | 1072,16 | 1157,33 | 650,73 | 2029,82 | 1138,20625 |
| SHexCer 41:8;3O | 8498,35 | 25342,4 | 17509,86 | 17835,5 | 16526,89 | 14368,1 | 6973,5 | 26019,66 | 16634,2838 |
| SHexCer 42:2;2O | 106,36 | 243,17 | 196,83 | 376,42 | 326,3 | 237,24 | 202,41 | 225,95 | 239,335 |
| SHexCer 42:4;3O | 2573,12 | 4607,85 | 6527,38 | 4290,88 | 3965,46 | 4707,85 | 3485,06 | 6484,27 | 4580,23375 |
| SHexCer 43:0;3O | 935,83 | 271,35 | 559,88 | 1056,67 | 1021,09 | 1531,72 | 1016,4 | 329,05 | 840,24875 |
| SHexCer 44:2;2O | 1330,88 | 2153,01 | 1946,59 | 2160,39 | 2010,96 | 2103,41 | 2075,11 | 1846,31 | 1953,3325 |
| SHexCer 46:2;2O | 3092,38 | 6817,5 | 9591,72 | 11061,85 | 11235,72 | 7789,3 | 6698,83 | 8374,71 | 8082,75125 |
| **Sphingomyelin** |  |  |  |  |  |  |  |  |  |
| SM 30:0;2O|SM 22:0;2O/8:0 | 994,31 | 793,16 | 509,61 | 427,68 | 561,58 | 801,09 | 958,12 | 965,02 | 751,32125 |
| SM 30:1;2O|SM 16:1;2O/14:0 | 1350,18 | 1303,48 | 665,47 | 740,12 | 846,56 | 901,96 | 1237,29 | 1121 | 1020,7575 |
| SM 31:0;2O|SM 19:0;2O/12:0 | 254,05 | 204,97 | 195,06 | 196,42 | 175,77 | 231,96 | 249,39 | 217,69 | 215,66375 |
| SM 31:1;2O|SM 17:1;2O/14:0 | 974,96 | 871,4 | 681,96 | 823,24 | 599,88 | 790,3 | 855,7 | 830,92 | 803,545 |
| SM 32:0;2O | 737,29 | 700,7 | 632,09 | 810,42 | 634,98 | 820,65 | 620,46 | 678,84 | 704,42875 |
| SM 32:0;2O|SM 22:0;2O/10:0 | 30816,94 | 15593,5 | 15868,36 | 19535,22 | 19191,31 | 32218,6 | 21602,9 | 16932,82 | 21469,9625 |
| SM 32:1;2O | 418,22 | 614,2 | 599,36 | 368,21 | 360,62 | 527,78 | 213,29 | 286,11 | 423,47375 |
| SM 32:1;2O|SM 16:1;2O/16:0 | 70392,99 | 38566 | 35025,32 | 36507,33 | 39556,54 | 67615,6 | 44793,7 | 38029,61 | 46310,8975 |
| SM 33:0;2O | 3129,65 | 2344,52 | 2207,39 | 2393,11 | 2503,58 | 3868,44 | 2385,53 | 2192,54 | 2628,095 |
| SM 33:1;2O | 553,01 | 524,47 | 725,88 | 711,75 | 577,76 | 754,72 | 427,31 | 646,99 | 615,23625 |
| SM 33:1;2O|SM 17:1;2O/16:0 | 34595,43 | 18413,1 | 24176,66 | 24246,57 | 23819,49 | 38342,4 | 22244 | 19842,49 | 25710,0063 |
| SM 34:0;2O | 19839,12 | 18570,5 | 23571,64 | 42820,53 | 38079,35 | 37725,9 | 16731,1 | 23835,09 | 27646,6488 |
| SM 34:1;2O | 18984,14 | 5499,3 | 10722,08 | 14817,44 | 13518,23 | 24643 | 11081,6 | 8588,54 | 13481,7813 |
| SM 34:1;2O|SM 18:1;2O/16:0 | 374997,5 | 218387 | 314645,15 | 411393,5 | 394934,7 | 478194 | 294096 | 302043,15 | 348586,369 |
| SM 34:1;3O | 792,94 | 718,28 | 677,36 | 934,54 | 735,51 | 858,54 | 462,53 | 700,84 | 735,0675 |
| SM 34:2;2O|SM 25:2;2O/9:0 | 3106,15 | 2470,43 | 2722,11 | 3365,83 | 3432,7 | 3759,79 | 3371,17 | 2253,19 | 3060,17125 |
| SM 35:0;2O | 727,59 | 448,52 | 630,87 | 556,11 | 628,65 | 942,37 | 493,52 | 534,64 | 620,28375 |
| SM 35:1;2O|SM 16:1;2O/19:0 | 6930,21 | 3978,95 | 5618,2 | 5465,58 | 5118,12 | 9317,8 | 3891,92 | 4754,06 | 5634,355 |
| SM 35:1;2O|SM 18:1;2O/17:0 | 3027,5 | 3264,49 | 3505,77 | 3124,82 | 2826,44 | 3680,06 | 2705,76 | 3334,97 | 3183,72625 |
| SM 35:2;2O | 442,7 | 599,42 | 363,49 | 395,69 | 459,53 | 438,09 | 328,85 | 527,81 | 444,4475 |
| SM 35:2;2O|SM 21:1;2O/14:1 | 356,21 | 421,94 | 282,55 | 425,82 | 386,59 | 383,34 | 300,67 | 376,94 | 366,7575 |
| SM 35:2;3O | 946,27 | 1119,26 | 816,73 | 1194,27 | 1237,06 | 976,19 | 877,42 | 1006,53 | 1021,71625 |
| SM 36:0;2O | 1527,15 | 1600,75 | 1675,29 | 2309,3 | 2282,64 | 1446,57 | 933,97 | 1733,77 | 1688,68 |
| SM 36:1;2O|SM 15:0;2O/21:1 | 11939,2 | 6592,87 | 10138,99 | 10670,82 | 10702,52 | 11235,1 | 8717,22 | 9362,9 | 9919,9475 |
| SM 36:2;2O|SM 19:1;2O/17:1 | 2675,87 | 1663,59 | 2373,31 | 2354,15 | 2177,7 | 3157,51 | 2278,95 | 1595,95 | 2284,62875 |
| SM 37:0;2O | 1272,19 | 1467,47 | 908,38 | 1420,36 | 1505,74 | 1063,19 | 1128,58 | 1360,32 | 1265,77875 |
| SM 37:1;2O | 664,58 | 800,79 | 748,38 | 694,94 | 570,3 | 662,84 | 528 | 1047,19 | 714,6275 |
| SM 38:0;2O|SM 16:0;2O/22:0 | 9292,83 | 15057,1 | 13838,19 | 12378,12 | 12657,37 | 11307,6 | 7946,73 | 16034,9 | 12314,1025 |
| SM 38:1;2O|SM 16:1;2O/22:0 | 101747,9 | 79009,1 | 78867,9 | 59543,73 | 61122,36 | 79551,6 | 55808,9 | 70084,88 | 73217,0563 |
| SM 38:2;2O|SM 19:2;2O/19:0 | 792,93 | 884,91 | 757,12 | 877,26 | 831,16 | 617,13 | 654,8 | 817,11 | 779,0525 |
| SM 39:0;2O | 28093,87 | 35227,9 | 39861,05 | 25102,25 | 20284,59 | 27723,7 | 22949,6 | 36553,32 | 29474,535 |
| SM 39:1;2O|SM 17:1;2O/22:0 | 1508,75 | 1401,86 | 1380,74 | 1499,56 | 1482,16 | 1499,23 | 1576,1 | 1507,92 | 1482,04 |

| SM 39:1;3O | 4336,53 | 4943,34 | 5628,38 | 5595,59 | 4361,27 | 5173,89 | 2239,52 | 5652,88 | 4741,425 |
| --- | --- | --- | --- | --- | --- | --- | --- | --- | --- |
| SM 39:2;2O|SM 19:1;2O/20:1 | 1047,42 | 1515,56 | 1119,1 | 1346,32 | 1354,46 | 1449,59 | 747,95 | 1233,44 | 1226,73 |
| SM 40:0;2O | 7598,62 | 17266,4 | 12403,39 | 14067,66 | 13555,28 | 9502,51 | 5565,78 | 19485,09 | 12430,585 |
| SM 40:1;2O | 2693,83 | 3505,73 | 3561,57 | 2699,04 | 2715,64 | 2768,07 | 1483,53 | 3847,83 | 2909,405 |
| SM 40:1;2O|SM 16:1;2O/24:0 | 161034,3 | 204313 | 202376,5 | 186159,15 | 165002,35 | 187856 | 97921,6 | 196900,75 | 175195,43 |
| SM 40:1;2O|SM 18:1;2O/22:0 | 1784,14 | 2419,94 | 2545,19 | 2717,01 | 2584,67 | 2348,91 | 1585,84 | 2582,85 | 2321,06875 |
| SM 40:1;3O | 1200,39 | 1617,56 | 1185,02 | 1086,89 | 1103,32 | 1248,65 | 248,52 | 1419,83 | 1138,7725 |
| SM 40:2;2O|SM 18:1;2O/22:1 | 13400,32 | 10065,8 | 9349 | 8919,66 | 8451,92 | 10394,3 | 8311,63 | 7923,38 | 9601,99625 |
| SM 40:8;2O | 482,7 | 1513,53 | 837,76 | 1495,68 | 1144,46 | 601,48 | 542,96 | 1070,84 | 961,17625 |
| SM 41:0;2O | 2618,2 | 5077,24 | 5926,17 | 5225,4 | 4581,47 | 2621,38 | 2378,6 | 8933,45 | 4670,23875 |
| SM 41:1;2O|SM 18:1;2O/23:0 | 16211,77 | 54548,9 | 52655,49 | 44503,64 | 38423,25 | 29872,1 | 14785,7 | 104424,65 | 44428,185 |
| SM 41:2;2O|SM 18:1;2O/23:1 | 4265,94 | 5337,57 | 7350,98 | 7180,63 | 7078,46 | 5323,98 | 3412,33 | 7096,26 | 5880,76875 |
| SM 42:1;2O|SM 18:1;2O/24:0 | 33089,78 | 49992,2 | 78900,73 | 69771,58 | 60921,11 | 34999,5 | 21493,9 | 92892,3 | 55257,6275 |
| SM 42:2;2O | 202,6 | 315,59 | 466,55 | 451,55 | 381,35 | 306,8 | 216,91 | 428,9 | 346,28125 |
| SM 42:2;2O|SM 18:1;2O/24:1 | 7733,75 | 9533,32 | 15312,75 | 15628,38 | 12914,99 | 12973 | 7474,85 | 13218,49 | 11848,6875 |
| SM 42:2;3O | 226,24 | 298,12 | 310,3 | 250,35 | 208,01 | 297,5 | 248,26 | 314,63 | 269,17625 |
| SM 42:3;2O | 462,55 | 601,35 | 731,18 | 787 | 777,45 | 557,24 | 617,76 | 616,93 | 643,9325 |
| SM 43:2;2O|SM 18:1;2O/25:1 | 816,36 | 1401,77 | 1323,93 | 1363,9 | 1552,36 | 825,78 | 498,98 | 1640,48 | 1177,945 |
| SM 44:8;2O | 83,48 | 1059,19 | 328,38 | 489,29 | 470,05 | 187 | 95,54 | 468,57 | 397,6875 |
| **Triacylglycerols** |  |  |  |  |  |  |  |  |  |
| TG 10:0_10:0_20:5 | 47649,86 | 65963 | 32428,59 | 98376,28 | 63755,41 | 43544,6 | 29081,2 | 22959,1 | 50469,76 |
| TG 46:0|TG 14:0_16:0_16:0 | 19259,73 | 38728,7 | 59442,29 | 53220,5 | 17758,66 | 14452,9 | 17494,9 | 64166,74 | 35565,5525 |
| TG 48:1|TG 16:0_16:0_16:1 | 72559,61 | 96317,8 | 128893,45 | 118875,2 | 107694,52 | 70495,6 | 92759,8 | 137241,25 | 103104,65 |
| TG 49:3|TG 15:0_16:0_18:3 | 24973,43 | 14969,8 | 49997,43 | 30648,66 | 10921,82 | 23993,4 | 39596,9 | 21824,91 | 27115,785 |
| TG 50:2|TG 16:0_16:0_18:2 | 69598,12 | 104722 | 159057,95 | 126952,75 | 130898,49 | 88460 | 80988 | 185417,55 | 118261,906 |
| TG 51:2|TG 15:0_18:1_18:1 | 12869 | 20248,2 | 38025,69 | 15883,46 | 15038,12 | 12080,6 | 18156,6 | 24812,73 | 19639,2988 |
| TG 51:4|TG 15:0_16:0_20:4 | 15661,54 | 8564,05 | 39149,97 | 17194,09 | 9925,24 | 11048,7 | 31931,2 | 13362,79 | 18354,695 |
| TG 52:2|TG 16:0_18:1_18:1 | 34778,35 | 81992,8 | 145541,61 | 55032,22 | 41714,08 | 28646,2 | 76120,5 | 132534,2 | 74544,9975 |
| TG 52:3|TG 16:0_18:1_18:2 | 1352489 | 1987101 | 3113385 | 2160060 | 1902553,5 | 1130660 | 2267476 | 3097074,5 | 2126349,69 |
| TG 53:4|TG 17:1_18:1_18:2 | 14502,11 | 11758,4 | 19876,9 | 15046,12 | 12891,03 | 15158,7 | 18029,4 | 18212,86 | 15684,44 |
| TG 54:6|TG 18:1_18:2_18:3 | 24129,76 | 15774,6 | 55345,08 | 42566,5 | 26918,69 | 14632,9 | 41918,8 | 32473,53 | 31719,9913 |
| TG 56:6|TG 16:0_18:1_22:5 | 29367,37 | 23215,6 | 56952,28 | 44893,15 | 42198,36 | 28408,7 | 32438,3 | 67240,54 | 40589,2888 |
| TG 57:6|TG 17:0_18:1_22:5 | 766,42 | 1144,62 | 3850,59 | 1047,68 | 1203,75 | 924,69 | 1771,04 | 2435,87 | 1643,0825 |
| TG 8:0_8:0_16:4 | 1203,08 | 2045,46 | 821,27 | 1471,63 | 1264,74 | 1158,1 | 1030,17 | 799,09 | 1224,1925 |
| TG O-42:5|TG O-20:5_11:0_11:0 | 793,36 | 4813,33 | 6600,28 | 2090,91 | 2432,17 | 814,13 | 2102,35 | 8037,77 | 3460,5375 |
| TG O-49:6|TG O-15:4_17:1_17:1 | 3463,62 | 10101,6 | 14383,12 | 9796,62 | 5005,86 | 3078,71 | 3797,56 | 20827,7 | 8806,85 |
| TG O-51:6|TG O-15:4_17:0_19:2 | 1937,63 | 6102,21 | 10800,98 | 8628,14 | 4215,57 | 2430,24 | 2394,97 | 13318,1 | 6228,48 |
| TG O-51:7|TG O-15:4_15:0_21:3 | 8197,24 | 14067,6 | 15926,69 | 13723,97 | 15015,76 | 8172,43 | 10913,3 | 20005,45 | 13252,805 |
| TG O-52:7|TG O-18:4_16:0_18:3 | 1637,47 | 5024,89 | 7323,98 | 3285,56 | 3491,04 | 1585,75 | 2369,62 | 7071,27 | 3973,6975 |
| TG O-54:8|TG O-15:4_18:1_21:3 | 1557,68 | 4880,95 | 6909,84 | 1835,48 | 2601,67 | 1737,32 | 2437,63 | 5572,12 | 3441,58625 |
| TG O-55:9|TG O-13:1_21:4_21:4 | 3657,7 | 9520,19 | 10786,37 | 6974,94 | 5941,76 | 2158,38 | 6597,99 | 15093,16 | 7591,31125 |
| TG O-57:10|TG O-15:0_21:5_21:5 | 1347,1 | 4709,82 | 5316,76 | 2052,13 | 2657,6 | 1326,98 | 3654,09 | 6744,77 | 3476,15625 |
| TG O-57:9|TG O-15:1_21:4_21:4 | 1526,62 | 3430,47 | 5303,1 | 1551,09 | 1773,22 | 855,84 | 3808,68 | 5955,81 | 3025,60375 |
|  |  |  |  |  |  |  |  |  |  |
| **Others** |  |  |  |  |  |  |  |  |  |

| CoQ8 | 514,74 | 943,7 | 860,44 | 1522,51 | 1065,43 | 1079,24 | 351,56 | 864,26 | 900,235 |
| --- | --- | --- | --- | --- | --- | --- | --- | --- | --- |
| DGCC 17:2_22:6 | 1211,56 | 1350,71 | 1864,34 | 1677,66 | 1346,15 | 1488,73 | 1827,15 | 1798,02 | 1570,54 |
| DGGA 36:2|DGGA 18:1_18:1 | 1758,82 | 4662,03 | 2809,84 | 3249,51 | 2748,94 | 3395,9 | 1854,03 | 3502 | 2997,63375 |
| DGTS 21:0 | 359,26 | 713,06 | 280,65 | 693,48 | 551,18 | 348,46 | 295,83 | 392,92 | 454,355 |
| LPA 16:3 | 1789,18 | 3651,06 | 2916,42 | 3027,56 | 2344,97 | 1690,06 | 1417,74 | 2595,79 | 2429,0975 |
| LPE O-19:4 | 448,57 | 1641,03 | 829,47 | 912,28 | 1144,77 | 527,09 | 502,39 | 1320 | 915,7 |
| LPE-N (FA)33:2|LPE-N (FA 15:0)18:2 | 888,31 | 1359,48 | 604,81 | 1592,66 | 1290,07 | 970,23 | 1063,9 | 673,81 | 1055,40875 |
| LPG O-13:0 | 843,62 | 940,47 | 777,7 | 1170,31 | 1066,96 | 1012,27 | 977,86 | 797,07 | 948,2825 |
| NAGly 9:0;O | 423,01 | 1509,62 | 1540,72 | 753,11 | 1226,11 | 490,31 | 468,61 | 2060,93 | 1059,0525 |
| Pentaerythritol tetrakis(3,5-di-tert-butyl-4-hydro | 9612,53 | 22454,4 | 23662,33 | 21261,57 | 33391,2 | 18016,7 | 14178,7 | 19100,38 | 20209,7363 |
| PI O-16:0 | 3220,39 | 1945,51 | 793,38 | 3031,52 | 2138,46 | 2112,99 | 2712,72 | 1729,29 | 2210,5325 |
| PI-Cer 38:3;2O | 542,72 | 540,58 | 402,49 | 650,9 | 526,13 | 627,55 | 645,97 | 553,48 | 561,2275 |
| PMeOH 40:4|PMeOH 20:2_20:2 | 366,77 | 117,36 | 59,24 | 682,02 | 289,11 | 725,34 | 318,21 | 67,59 | 328,205 |
| ST 27:1;O;S - Cholesterol sulfate | 1393,96 | 1392,9 | 2242,82 | 1152,98 | 950,82 | 1543,71 | 801,83 | 2869,67 | 1543,58625 |
| SL 12:0;O/26:0 | 4435,18 | 3737,96 | 3825,12 | 4339,19 | 4021,88 | 4924,19 | 5073,13 | 4329,18 | 4335,72875 |
| SL 12:1;O/32:0 | 2966,46 | 5457,82 | 6577,59 | 4477,71 | 4472,87 | 3905,25 | 4245,93 | 6513,54 | 4827,14625 |
| **EO Treated T21** |  |  |  |  |  |  |  |  |  |
| **Metabolyte name** | **t21_T1** | **t21_T2** | **t21_T3** | **t21_T4** | **t21_T5** | **t21_T6** | **t21_T7** | **t21_T8** | **average value** |
| **Acyl sterol glycoside** |  |  |  |  |  |  |  |  |  |
| ASG 27:1;O;Hex;FA 2:0 | 974,08 | 736,4 | 1346,31 | 1667,59 | 1795,54 | 1385,8 | 1672,4 | 1218,64 | 1349,595 |
| ASG 28:1;O;Hex;FA 14:0 | 755,56 | 357,52 | 994,84 | 3567,02 | 2003,74 | 3455,49 | 462,09 | 499,13 | 1511,92375 |
| ASG 28:1;O;Hex;FA 16:0 | 476,54 | 215,51 | 733,87 | 2542,62 | 1967,52 | 3153,47 | 417,12 | 499,85 | 1250,8125 |
| ASG 28:1;O;Hex;FA 18:0 | 327,38 | 128,44 | 509,47 | 1635,39 | 1669,55 | 3195,25 | 339,29 | 329,82 | 1016,82375 |
| ASG 28:1;O;Hex;FA 20:1 | 452,77 | 178,06 | 618,21 | 1888,35 | 1043,58 | 1895,95 | 201,43 | 592,32 | 858,83375 |
| ASG 28:2;O;Hex;FA 14:0 | 1821,61 | 642,01 | 1833,3 | 7082,99 | 3870,26 | 6471,9 | 813,4 | 1354,44 | 2986,23875 |
| ASG 28:2;O;Hex;FA 16:0 | 875,63 | 301,5 | 1105,47 | 3196,54 | 2015,03 | 3477,62 | 490,41 | 619,67 | 1510,23375 |
| ASG 28:2;O;Hex;FA 16:1 | 522,38 | 236,47 | 639,81 | 1963,02 | 1362,47 | 1938,32 | 140,58 | 299,6 | 887,83125 |
| ASG 28:2;O;Hex;FA 20:1 | 399,28 | 162,19 | 385,43 | 1299,71 | 1062,05 | 1367,33 | 152,98 | 349,07 | 647,255 |
| ASG 29:2;O;Hex;FA 20:5 | 958,22 | 357,52 | 671,63 | 1134,06 | 993,03 | 973,85 | 868,48 | 946,49 | 862,91 |
| **Carnitines** |  |  |  |  |  |  |  |  |  |
| CAR 13:0 | 699,11 | 305,55 | 4605,93 | 591,71 | 1252,98 | 2566,77 | 243,13 | 1160,04 | 1428,1525 |
| CAR 21:2 | 4165,54 | 1702,28 | 3657,26 | 9165,38 | 7113,01 | 11745,1 | 1445,45 | 3353,49 | 5293,43375 |
| CAR 21:4 | 8137,03 | 1789,02 | 1746,02 | 5934,46 | 7497,57 | 7658,31 | 1215,59 | 4976,99 | 4869,37375 |
| CAR 4:0 | 9383,97 | 1774,18 | 8532,06 | 16680,11 | 20616,68 | 14502,7 | 2535,7 | 17431 | 11432,0538 |
| CAR 5:0 | 4259,72 | 911,28 | 5689,47 | 15647,08 | 27198,81 | 18289,5 | 3000,7 | 10742,18 | 10717,3375 |
| **Ceramides** |  |  |  |  |  |  |  |  |  |
| Cer 12:0;2O/25:0 | 4489,94 | 2070 | 5267,5 | 7428,38 | 6294,26 | 9576,18 | 3063,2 | 4248,67 | 5304,76625 |
| Cer 12:2;2O/19:4 | 1264,57 | 416,18 | 1339,49 | 2468,49 | 2494,26 | 3420,43 | 546,79 | 1058,42 | 1626,07875 |
| Cer 13:2;2O/30:3 | 3566,84 | 1747,75 | 2307,3 | 4745,52 | 3969,59 | 3946,18 | 1966,59 | 3440,42 | 3211,27375 |
| Cer 20:2;4O|Cer 12:2;3O/8:0;(2OH) | 2181,76 | 785,04 | 3808,43 | 4776,45 | 3292,81 | 4869,6 | 625,92 | 2861,78 | 2900,22375 |
| Cer 24:3;3O|Cer 15:3;2O/9:0;O | 2035,94 | 1203,23 | 4743,76 | 2516,75 | 2793,08 | 4751,47 | 1254,29 | 2200,91 | 2687,42875 |
| Cer 32:0;2O|Cer 16:0;2O/16:0 | 818,53 | 335,83 | 753,16 | 997,75 | 1014,12 | 1274,58 | 322,38 | 603,64 | 764,99875 |
| Cer 32:1;2O|Cer 16:1;2O/16:0 | 600,09 | 381,98 | 493,38 | 779,23 | 726,53 | 673,8 | 561,59 | 419,93 | 579,56625 |
| Cer 33:1;2O|Cer 17:1;2O/16:0 | 301,9 | 90,36 | 204,68 | 412,49 | 494,5 | 585,33 | 207,49 | 149,21 | 305,745 |

| Cer 33:1;4O|Cer 18:0;3O/15:1;(2OH) | 2337,45 | 780,52 | 2238,76 | 3260,97 | 2812,43 | 5567,41 | 618,7 | 1530,79 | 2393,37875 |
| --- | --- | --- | --- | --- | --- | --- | --- | --- | --- |
| Cer 34:1;2O|Cer 18:1;2O/16:0 | 3963,93 | 1776,9 | 2662,06 | 4578,2 | 6148,38 | 6920,76 | 2922,69 | 2633,11 | 3950,75375 |
| Cer 34:1;3O|Cer 19:0;2O/15:1;O | 2497,26 | 2201,71 | 2439,28 | 3400,56 | 2749,98 | 2412,2 | 1276,88 | 1970,05 | 2368,49 |
| Cer 34:3;2O|Cer 12:2;2O/22:1 | 1667,78 | 378,85 | 1527,31 | 2982,7 | 2091,25 | 3743,02 | 1301,6 | 1532,2 | 1903,08875 |
| Cer 36:0;3O|Cer 18:0;2O/18:0;O | 15797,11 | 9693,57 | 13428,23 | 20075,09 | 17569,66 | 23883,5 | 5820,59 | 12659,52 | 14865,9125 |
| Cer 36:1;3O|Cer 19:0;2O/17:1;O | 744,63 | 574,27 | 755,71 | 822,87 | 784,44 | 1023,66 | 299,51 | 532,3 | 692,17375 |
| Cer 36:3;4O|Cer 19:2;3O/17:1;(2OH) | 1925,46 | 1533,13 | 1676,57 | 2364,32 | 2513,16 | 2790,67 | 875,26 | 1928,3 | 1950,85875 |
| Cer 37:0;2O|Cer 19:0;2O/18:0 | 2180,11 | 531,09 | 2897,72 | 3132,88 | 1680,24 | 2632,78 | 399,43 | 1361,19 | 1851,93 |
| Cer 38:0;4O | 779,84 | 316,93 | 949,85 | 1376,02 | 574,78 | 1599,95 | 220,83 | 750,18 | 821,0475 |
| Cer 39:0;3O|Cer 18:0;2O/21:0;O | 4733,62 | 1337,43 | 4050,92 | 5531,92 | 3776 | 4906,28 | 800,16 | 2449,36 | 3448,21125 |
| Cer 40:0;2O|Cer 16:0;2O/24:0 | 997,35 | 554,04 | 940,77 | 1354,7 | 1556,55 | 1938,1 | 574,21 | 926,43 | 1105,26875 |
| Cer 40:1;2O|Cer 16:1;2O/24:0 | 3487,708 | 4541,34 | 2068,37 | 2604,78 | 3663,36 | 4409,92 | 3969,07 | 1729,93 | 3309,30972 |
| Cer 41:1;2O|Cer 18:1;2O/23:0 | 1699,28 | 723,65 | 1036,46 | 1623,6 | 2425,2 | 2310,42 | 594,18 | 1049,91 | 1432,8375 |
| Cer 42:1;2O|Cer 18:1;2O/24:0 | 5367,09 | 2403,7 | 2965,02 | 5782,96 | 7568,68 | 8993,18 | 2801,11 | 2867,72 | 4843,6825 |
| Cer 44:2;4O|Cer 28:1;3O(FA 16:0) | 1732,67 | 727,73 | 1020,24 | 2685,53 | 2664,58 | 3125,41 | 1145,96 | 818,53 | 1740,08125 |
| **Diacylglycerols** |  |  |  |  |  |  |  |  |  |
| DG 16:0 | 2106,05 | 507 | 858,91 | 2364,01 | 2086,46 | 2595,18 | 875,41 | 750,51 | 1517,94125 |
| DG 18:0 | 9044,97 | 2886,73 | 2063,89 | 7442,2 | 6418,05 | 7039,62 | 2132,46 | 2472,29 | 4937,52625 |
| DG 20:0 | 33598,84 | 20328,9 | 10992,9 | 41277,31 | 53656,8 | 53886,5 | 15786 | 13975,31 | 30437,8325 |
| DG 21:0 | 932,51 | 245,65 | 446,86 | 1148,22 | 1224,67 | 1627,66 | 333,02 | 521,72 | 810,03875 |
| DG 22:0 | 17243,33 | 7730,29 | 6594,62 | 23871,19 | 36868,55 | 38097,6 | 13451,4 | 8444,5 | 19037,6863 |
| DG 22:0|DG 10:0_12:0 | 838,15 | 368,17 | 596,42 | 1734,58 | 1951,19 | 1992,93 | 3550,91 | 729,47 | 1470,2275 |
| DG 22:0|DG 8:0_14:0 | 964,93 | 476,46 | 593,25 | 1760,22 | 1935,06 | 2234,98 | 2852,53 | 722,07 | 1442,4375 |
| DG 22:1 | 8614,84 | 2106,06 | 2654,72 | 6664,33 | 9640,36 | 8905,99 | 2791,84 | 3856,93 | 5654,38375 |
| DG 22:1|DG 8:0_14:1 | 8419,71 | 2862,31 | 2769,8 | 9112,09 | 10324,06 | 10148,9 | 3548,86 | 4635,47 | 6477,65 |
| DG 24:0 | 9271,67 | 3532,85 | 6782,78 | 17895,26 | 20125,85 | 24431,2 | 37186,2 | 6181,29 | 15675,8788 |
| DG 24:0|DG 10:0_14:0 | 3908,74 | 1699,48 | 2926,24 | 7983,7 | 8769,57 | 11237 | 18140,9 | 2593,24 | 7157,36375 |
| DG 24:0|DG 8:0_16:0 | 3473,51 | 1875,38 | 2665,43 | 7232,34 | 11307,44 | 13048,9 | 9862,27 | 2451,89 | 6489,64625 |
| DG 24:1 | 15694,25 | 5703,92 | 5150,33 | 17627,31 | 25105,12 | 26626,7 | 10203,3 | 7122,02 | 14154,1113 |
| DG 24:1|DG 10:0_14:1 | 8183,73 | 3412,95 | 2716,3 | 9271,75 | 13538,12 | 14677 | 5353,8 | 3663,51 | 7602,14125 |
| DG 24:2 | 1234,67 | 412,95 | 362,26 | 1373,62 | 2128,07 | 2073,22 | 623,94 | 582,79 | 1098,94 |
| DG 24:3 | 504,82 | 223,42 | 201,68 | 525,57 | 653,96 | 719,62 | 315,71 | 261,23 | 425,75125 |
| DG 25:0|DG 10:0_15:0 | 1027,68 | 528,17 | 864,45 | 1999,45 | 2198,35 | 3236,42 | 1261,58 | 713,82 | 1478,74 |
| DG 26:0|DG 12:0_14:0 | 8461,84 | 3206,12 | 7398,1 | 19117,28 | 22771,87 | 28876,9 | 31886,1 | 5730,15 | 15931,055 |
| DG 26:1 | 9016,4 | 3337,39 | 5378,43 | 17285,36 | 21614,59 | 23310,4 | 13149,6 | 5080,66 | 12271,6025 |
| DG 26:1|DG 8:0_18:1 | 7020,3 | 3288,83 | 4037,24 | 14089,22 | 18604,92 | 21435,3 | 13521,2 | 4215,78 | 10776,6063 |
| DG 26:2 | 935,66 | 321,4 | 425,99 | 1165,16 | 1846,16 | 2597,66 | 828,09 | 509,31 | 1078,67875 |
| DG 26:2|DG 8:0_18:2 | 787,14 | 340,3 | 435,74 | 1474,06 | 2181,46 | 2588,21 | 743,89 | 473,9 | 1128,0875 |
| DG 26:3 | 496,39 | 141,83 | 84,81 | 735,38 | 936,23 | 922,99 | 609,41 | 244,65 | 521,46125 |
| DG 27:0 | 942,06 | 309,12 | 1188,13 | 2094,22 | 2104,3 | 3208,75 | 735,61 | 921,41 | 1437,95 |
| DG 27:5 | 1183,26 | 348,19 | 1285,62 | 2366,97 | 1541,95 | 2272,9 | 753,03 | 1016,51 | 1346,05375 |
| DG 28:0|DG 12:0_16:0 | 20368,76 | 8402,49 | 23410,9 | 53447,05 | 58440,74 | 89134,1 | 57483,3 | 14034,45 | 40590,225 |
| DG 28:1 | 9771,33 | 2647,06 | 9555,93 | 18796,88 | 19098,27 | 29595,2 | 12300,3 | 6974,51 | 13592,4338 |
| DG 28:1|DG 10:0_18:1 | 20880,02 | 8022,87 | 13145,3 | 39857,2 | 46542,35 | 67738 | 47464 | 11497,95 | 31893,47 |
| DG 28:2 | 5359,05 | 1249,62 | 3029,25 | 7945,38 | 9504,45 | 12140,7 | 1962,99 | 2960,87 | 5519,03375 |
| DG 28:2|DG 14:1_14:1 | 6343,64 | 2340,06 | 3050,84 | 12060,4 | 15620,13 | 18669,9 | 5846,88 | 3236,66 | 8396,0675 |

| DG 28:3 | 770,25 | 270,6 | 476,3 | 1210,62 | 1512,07 | 1926,16 | 1128,22 | 389,95 | 960,52125 |
| --- | --- | --- | --- | --- | --- | --- | --- | --- | --- |
| DG 28:3|DG 10:0_18:3 | 796,75 | 378,58 | 437,16 | 1316,66 | 1682,89 | 1901,7 | 1318,7 | 480,23 | 1039,08375 |
| DG 30:0 | 1117,36 | 322,88 | 562,36 | 1719,55 | 1346,78 | 2204,35 | 763,73 | 390,38 | 1053,42375 |
| DG 30:0|DG 14:0_16:0 | 79851,8 | 25345 | 73766,81 | 172396,25 | 174093,2 | 240105 | 95397,2 | 48351,39 | 113663,333 |
| DG 30:1|DG 16:0_14:1 | 31553,59 | 16983,7 | 40782,56 | 97685,3 | 184121,4 | 168631 | 81032,9 | 26369,03 | 80894,8725 |
| DG 30:2 | 2775,09 | 640,75 | 2453,01 | 5080,76 | 6440,45 | 8971,86 | 1098,14 | 1675,76 | 3641,9775 |
| DG 30:2|DG 12:0_18:2 | 4936,76 | 1687,46 | 3142,76 | 10330,4 | 13560,73 | 18819 | 5696,33 | 2338,16 | 7563,9475 |
| DG 30:2|DG 14:1_16:1 | 3815,16 | 1426,22 | 2974,7 | 8377,94 | 15765,39 | 17405,2 | 5563,94 | 2009,96 | 7167,31 |
| DG 30:3|DG 12:0_18:3 | 1201,59 | 532,44 | 807,03 | 1916,69 | 2672,11 | 3598,98 | 2336,55 | 830,07 | 1736,9325 |
| DG 30:5 | 332,95 | 71,52 | 270,68 | 461,81 | 338,11 | 671,82 | 13,75 | 298,84 | 307,435 |
| DG 30:6 | 5818,44 | 1494,94 | 5432,67 | 9324,7 | 6371,53 | 12559,4 | 1518,96 | 4407,5 | 5866,01375 |
| DG 30:7 | 729,42 | 176,52 | 682,02 | 1455,61 | 1258,22 | 1684,45 | 115,15 | 437,78 | 817,39625 |
| DG 31:7 | 48050,72 | 13918,1 | 42412,75 | 82263,53 | 35436,16 | 52277,1 | 7504,75 | 31862,73 | 39215,7275 |
| DG 31:8 | 4414,92 | 730,17 | 1783,8 | 4753,05 | 3709,3 | 6525,36 | 479,75 | 1529,53 | 2990,735 |
| DG 32:1|DG 14:0_18:1 | 76859,51 | 36094,5 | 93408,12 | 285806,85 | 251933,95 | 288877 | 128818 | 62041,03 | 152979,919 |
| DG 32:2 | 7374,65 | 1842,51 | 7045,04 | 12752,87 | 14241,32 | 20819,8 | 4825,65 | 4700,34 | 9200,27375 |
| DG 32:2|DG 14:1_18:1 | 25190,93 | 9355,14 | 16911,6 | 51767,84 | 79832 | 105196 | 25525,9 | 11521,23 | 40662,5575 |
| DG 32:3 | 1569,36 | 339,89 | 1084,25 | 2228,2 | 2764,57 | 3568,36 | 595,31 | 918,77 | 1633,58875 |
| DG 32:3|DG 14:0_18:3 | 4948,83 | 1682,1 | 2786,21 | 7406,61 | 8414,47 | 12606,7 | 3864,58 | 1950,1 | 5457,44875 |
| DG 32:6 | 5169,17 | 1467,29 | 6912,65 | 9019,72 | 7627,46 | 12568,4 | 1809,25 | 5519,36 | 6261,65625 |
| DG 32:7 | 1950,57 | 452,01 | 2223,34 | 3498,97 | 2236,23 | 5323,84 | 233,96 | 1174,1 | 2136,6275 |
| DG 33:2 | 3775,21 | 4689,12 | 4365,61 | 3784,36 | 5788,37 | 9538,99 | 4477,95 | 2582,05 | 4875,2075 |
| DG 33:3 | 3671,72 | 3759,38 | 3050,7 | 2581,91 | 3066,81 | 5789,44 | 2200,35 | 2848,16 | 3371,05875 |
| DG 33:6 | 54217,85 | 9446,38 | 47800,19 | 63547,6 | 49566,72 | 93539,3 | 9766,84 | 53739,64 | 47703,065 |
| DG 33:8 | 8712,8 | 2037,34 | 6043,6 | 14963,2 | 10388,64 | 20192,4 | 1199,34 | 4136,34 | 8459,20625 |
| DG 33:9 | 1163,27 | 216,11 | 480,85 | 883,56 | 881,53 | 1084,03 | 372,05 | 517,99 | 699,92375 |
| DG 34:0 | 723,27 | 293,44 | 759,03 | 1088,56 | 1012,3 | 1635,3 | 173,54 | 746,24 | 803,96 |
| DG 34:0|DG 16:0_18:0 | 7021,16 | 2631,05 | 7120,88 | 15349,68 | 12838,03 | 17888,1 | 9028,57 | 6921,21 | 9849,82875 |
| DG 34:1|DG 16:0_18:1 | 134056,2 | 46903 | 160673 | 343410,35 | 336765,8 | 385917 | 145012 | 95696,25 | 206054,219 |
| DG 34:2 | 298,67 | 135,42 | 429,89 | 538,61 | 318,67 | 787,3 | 112,04 | 358,81 | 372,42625 |
| DG 34:2|DG 16:0_18:2 | 63947,85 | 22453,8 | 49165,76 | 130118,4 | 175745,05 | 242864 | 69550,3 | 35204,7 | 98631,2138 |
| DG 34:3|DG 16:0_18:3 | 10571,53 | 2802,33 | 6210,58 | 17100,19 | 29944,14 | 45020,7 | 7497,5 | 5432,57 | 15572,445 |
| DG 34:6 | 5646,62 | 1834,99 | 8753,44 | 10740,21 | 7281,43 | 12198,6 | 2375,83 | 5208,92 | 6755,00125 |
| DG 34:7 | 3585,07 | 945,54 | 4328,86 | 7110,03 | 4292,98 | 8563,17 | 697,78 | 2905,75 | 4053,6475 |
| DG 35:2 | 2087,93 | 2084,92 | 2496,29 | 2162,45 | 2846,44 | 4555 | 3034,95 | 1992,28 | 2657,5325 |
| DG 35:3 | 1928,18 | 1404,75 | 1564,84 | 1640,27 | 2072,42 | 3501,99 | 1282,37 | 1662,75 | 1882,19625 |
| DG 35:8 | 23043,17 | 6290,54 | 19223,81 | 37353,38 | 22693,81 | 38122,3 | 3216,81 | 14651,78 | 20574,4438 |
| DG 35:9 | 6541,3 | 1194,77 | 4506,3 | 9070,47 | 5839,23 | 13370,7 | 869,78 | 2967,81 | 5545,05 |
| DG 36:1|DG 18:0_18:1 | 10997,75 | 4726,93 | 12952,97 | 15818,02 | 10727,53 | 11247,6 | 9877,73 | 10627,78 | 10872,0425 |
| DG 36:2|DG 18:1_18:1 | 46414,32 | 23246,6 | 55623,87 | 107028,19 | 77602,82 | 110594 | 52044,6 | 59161,03 | 66464,37 |
| DG 36:3|DG 18:1_18:2 | 33038,85 | 9633,82 | 19646,77 | 53275,08 | 52494,83 | 72398,5 | 20638,2 | 20027,75 | 35144,225 |
| DG 36:4|DG 18:1_18:3 | 6321,29 | 1932,61 | 4261,51 | 10638,4 | 9538,09 | 15736,3 | 4302,31 | 3663,91 | 7049,30625 |
| DG 36:5|DG 16:0_20:5 | 1152,54 | 363,27 | 647,18 | 2051,47 | 2106,76 | 3462,98 | 656,39 | 548,27 | 1373,6075 |
| DG 36:5|DG 18:2_18:3 | 1497,93 | 546,49 | 1052,55 | 2670,62 | 2792,31 | 4489,95 | 1569,3 | 940,19 | 1944,9175 |
| DG 36:7 | 4151,58 | 1376 | 5836,63 | 7262 | 5679,47 | 9712,02 | 972,15 | 4067,13 | 4882,1225 |
| DG 36:8 | 1438,36 | 1068,5 | 1638,37 | 1404,04 | 1367,27 | 2704,09 | 617,64 | 1287,1 | 1440,67125 |

| DG 37:9 | 9455,06 | 1946,18 | 6848,58 | 12971,07 | 8170,42 | 15779,8 | 1901,46 | 5450,04 | 7815,3225 |
| --- | --- | --- | --- | --- | --- | --- | --- | --- | --- |
| DG 38:5|DG 16:0_22:5 | 5538,52 | 2589,1 | 7173,33 | 14866,75 | 16827,25 | 26430,3 | 9137,7 | 6201,67 | 11095,58 |
| DG 38:6 | 452,22 | 98,68 | 385,9 | 765,26 | 559,36 | 861,8 | 52,42 | 360,46 | 442,0125 |
| DG 40:6|DG 18:1_22:5 | 1691,8 | 635,66 | 1688,84 | 3756,81 | 3231,49 | 4203,29 | 852,46 | 1637,49 | 2212,23 |
| DG 40:8 | 1047,11 | 699,34 | 1577,47 | 1312,54 | 1438,8 | 2425,08 | 540,78 | 1034,09 | 1259,40125 |
| DG 41:10 | 1151,02 | 315,96 | 835,27 | 1557,92 | 1425,48 | 1911,67 | 125,97 | 645,98 | 996,15875 |
| DG 41:11 | 496,39 | 166,51 | 461,01 | 812,82 | 400,13 | 690,81 | 118,05 | 321,35 | 433,38375 |
| DG 41:5 | 2510,49 | 861,65 | 2169,03 | 4080,76 | 3021,55 | 5685,6 | 430,32 | 1924,88 | 2585,535 |
| DG 41:6 | 21299,07 | 6915,21 | 12571,41 | 34948,71 | 21531,08 | 31878,9 | 7111,44 | 10116,79 | 18296,5738 |
| DG 43:11 | 973,21 | 320,71 | 1137,02 | 1456,82 | 797,43 | 1444,13 | 176,68 | 780,15 | 885,76875 |
| DG 43:3 | 633,18 | 217,79 | 635,8 | 956,77 | 840,59 | 1272,92 | 149,69 | 589,9 | 662,08 |
| DG 43:4 | 2561,39 | 657,75 | 2371,26 | 3236,3 | 2536,33 | 4163,12 | 747,59 | 1959,91 | 2279,20625 |
| DG 43:6 | 11683,33 | 5494,24 | 13238,35 | 20177,87 | 13136,01 | 24352,3 | 4562,49 | 9919,18 | 12820,4675 |
| DG 43:7 | 19714,31 | 7981,64 | 18572,04 | 28601,27 | 19464,46 | 27413,2 | 6869,71 | 17229,02 | 18230,705 |
| DG 44:7 | 1513,07 | 696,51 | 883,13 | 1427,23 | 1747,76 | 1448,5 | 1157,76 | 1001,11 | 1234,38375 |
| DG 44:9 | 1185,11 | 935,37 | 922,83 | 1520,01 | 1975,81 | 1610,08 | 1318,97 | 1300,71 | 1346,11125 |
| DG 46:6 | 4304,75 | 4677,59 | 4045,16 | 3612,96 | 4815,24 | 3351,62 | 3996,54 | 4716,68 | 4190,0675 |
| DG 47:7 | 9820,35 | 3605,17 | 12526,59 | 12197,47 | 15745,12 | 18595,7 | 3416,14 | 10957,45 | 10858,0025 |
| DG 47:8 | 9612,48 | 3425,9 | 10583,02 | 11416,84 | 11488,4 | 18794,6 | 3206,6 | 8218,3 | 9593,27125 |
| DG 51:7 | 3887,09 | 1053,52 | 3464,23 | 6356,58 | 5458,72 | 9618,46 | 770,97 | 2571,8 | 4147,67125 |
| DG 51:8 | 1928,19 | 765,66 | 1991,19 | 3102,36 | 3269,04 | 4943,37 | 592,2 | 1571,63 | 2270,455 |
| **Free Fatty Acids** |  |  |  |  |  |  |  |  |  |
| FA 12:0 | 915,52 | 1214,68 | 888,1 | 884,53 | 923,43 | 930,89 | 1081,64 | 948,4 | 973,39875 |
| FA 14:0 | 8839,68 | 23220 | 8191,34 | 9018,54 | 11201,28 | 8558,72 | 13084,6 | 6419,6 | 11066,7175 |
| FA 14:1 | 1275,94 | 1979,58 | 789,85 | 1289,68 | 3301,57 | 1609,27 | 1319,77 | 401,59 | 1495,90625 |
| FA 15:0 | 914,99 | 933,81 | 667,75 | 694,69 | 1095,25 | 922,1 | 790,21 | 675,76 | 836,82 |
| FA 15:4 | 1536,81 | 868,96 | 2112,5 | 2353,3 | 2356,24 | 2427,71 | 1330,6 | 1743,09 | 1841,15125 |
| FA 16:0 | 617212,8 | 719749 | 710999,35 | 752302,55 | 793220,2 | 777994 | 767250 | 574137,8 | 714108,194 |
| FA 16:0;(2OH) | 339,59 | 571,11 | 511,4 | 634,18 | 812,75 | 556,13 | 481,13 | 604,34 | 563,82875 |
| FA 16:0;O | 2017,7 | 2403,64 | 2161,84 | 2975,05 | 4119,96 | 2834,59 | 2500,79 | 2356,91 | 2671,31 |
| FA 16:1 | 5951,1 | 13265,4 | 6950,89 | 10785,75 | 19466,35 | 11901,4 | 12341,1 | 2983,73 | 10455,7 |
| FA 16:1;O | 2412,97 | 2591,88 | 3056,42 | 3346,45 | 4715,09 | 3722,79 | 2781,49 | 2811,27 | 3179,795 |
| FA 17:0 | 1603,73 | 1959,32 | 2241,66 | 1940,71 | 2062,14 | 1816,64 | 1941,16 | 1515,98 | 1885,1675 |
| FA 17:1 | 205,94 | 131,93 | 225,12 | 191,09 | 261,32 | 388,09 | 181,79 | 155,03 | 217,53875 |
| FA 17:4;O | 546,02 | 731,04 | 759,84 | 952,46 | 1079,87 | 902,56 | 757,38 | 1802,97 | 941,5175 |
| FA 18:0 | 675501,7 | 697389 | 862828,3 | 872074,35 | 837243,3 | 781276 | 888516 | 588841,95 | 775458,738 |
| FA 18:0;(2OH) | 2140,01 | 2736,68 | 3571,35 | 3921,2 | 4316,81 | 3645,32 | 3176,14 | 2172,3 | 3209,97625 |
| FA 18:0;O | 2121,74 | 1905,54 | 2264,22 | 2305,08 | 3477,77 | 2846,58 | 2307,33 | 1929,13 | 2394,67375 |
| FA 18:1 | 39544,11 | 28915,1 | 38551,8 | 52387,62 | 43560,8 | 38037,9 | 47784,6 | 26407,05 | 39398,6213 |
| FA 18:1;2O | 350,89 | 311,93 | 638,92 | 465,94 | 556,43 | 603,45 | 266,98 | 322,46 | 439,625 |
| FA 18:1;O | 1267,56 | 1227,58 | 1798,63 | 1185,78 | 2071,74 | 1953,32 | 1101,28 | 1453,13 | 1507,3775 |
| FA 18:2 | 2409,18 | 1762,4 | 1904,13 | 2871,06 | 2850,8 | 2534,75 | 3629,36 | 1324,06 | 2410,7175 |
| FA 18:2;O | 1768,53 | 2231,23 | 2445,58 | 2311,33 | 3252,39 | 2391,86 | 1945,94 | 2006,22 | 2294,135 |
| FA 18:3 | 2324,31 | 4215,6 | 1457,48 | 1817,79 | 2816,45 | 1732,73 | 5739,2 | 911,65 | 2626,90125 |
| FA 18:3;4O | 532,66 | 394,86 | 970,02 | 834,11 | 777,03 | 708,77 | 746,96 | 673,5 | 704,73875 |
| FA 18:3;O | 345,72 | 416,18 | 474,67 | 537,34 | 486,12 | 408,18 | 290,66 | 356,13 | 414,375 |

| FA 19:0 | 395,25 | 542,79 | 685,01 | 581,11 | 427,82 | 558,35 | 647,26 | 365,51 | 525,3875 |
| --- | --- | --- | --- | --- | --- | --- | --- | --- | --- |
| FA 19:0;(2OH) | 906,81 | 970,7 | 808,8 | 856,83 | 1148,78 | 830,64 | 751,09 | 1014,8 | 911,05625 |
| FA 19:1 | 118,76 | 180,76 | 111,26 | 213,78 | 135,59 | 124,2 | 213,58 | 85,4 | 147,91625 |
| FA 19:1;2O | 415,04 | 308,77 | 695,89 | 638,31 | 777,81 | 778,32 | 633,14 | 480,75 | 591,00375 |
| FA 19:1;O | 273,94 | 182,9 | 131,26 | 170,58 | 227,91 | 124 | 175,08 | 130,21 | 176,985 |
| FA 20:0 | 6144,01 | 7680,3 | 9484,79 | 9923,92 | 8187,42 | 10054,1 | 9890,62 | 6553,7 | 8489,8625 |
| FA 20:1 | 379,46 | 339,81 | 479,23 | 658,76 | 572,43 | 586,4 | 742,12 | 320,83 | 509,88 |
| FA 20:3 | 98,89 | 98,55 | 239,21 | 305,79 | 169,56 | 83,99 | 304,87 | 181,36 | 185,2775 |
| FA 20:4 | 1067,99 | 2222,63 | 1962,25 | 2152,63 | 2624,11 | 1411,39 | 3063,94 | 2206,99 | 2088,99125 |
| FA 20:5 | 370,69 | 654,76 | 504,47 | 736,74 | 718,1 | 1024,98 | 553,75 | 471 | 629,31125 |
| FA 21:0 | 563,84 | 968,6 | 1431,95 | 1008,88 | 971,35 | 1235,04 | 845,64 | 603,05 | 953,54375 |
| FA 21:1;2O | 218,67 | 286,16 | 570,93 | 396,69 | 483,78 | 303,6 | 323,45 | 324,35 | 363,45375 |
| FA 22:0 | 2298,5 | 2495,67 | 4119,54 | 3658,94 | 2988,55 | 3126,76 | 2955,98 | 2010,76 | 2956,8375 |
| FA 22:1 | 109,47 | 80,69 | 304,69 | 207,02 | 231,19 | 180,34 | 224,12 | 182,63 | 190,01875 |
| FA 22:5 | 674,27 | 368,85 | 1314,7 | 1727,84 | 1488,18 | 1360,41 | 1583,41 | 1301,01 | 1227,33375 |
| FA 22:6 | 746,15 | 1145,11 | 1637,66 | 1890,65 | 2121,13 | 1540,91 | 1926,81 | 1269,39 | 1534,72625 |
| FA 23:0 | 1450,74 | 1258,16 | 2313,93 | 1776,53 | 1614,87 | 1632,43 | 1431,14 | 1285,44 | 1595,405 |
| FA 23:1 | 137,52 | 141 | 155,29 | 443,57 | 262,1 | 217,98 | 388,52 | 70,83 | 227,10125 |
| FA 24:0 | 8116,46 | 7783,86 | 14433,77 | 13105,16 | 9223,42 | 13467,3 | 8605,86 | 7314,95 | 10256,35 |
| FA 25:0 | 3169,49 | 3197,07 | 5423,51 | 5500,29 | 3978,95 | 5972,72 | 5695,97 | 2857,16 | 4474,395 |
| FA 26:0 | 18808,16 | 18233,8 | 35155,97 | 31258,13 | 19919,36 | 30206,4 | 18139,2 | 16633,32 | 23544,2938 |
| FA 26:1;O | 1012,53 | 1320,84 | 1921,96 | 2308,99 | 1918,2 | 1832,59 | 2049,03 | 1225,67 | 1698,72625 |
| FA 27:0 | 5885,15 | 6476,05 | 12210,1 | 12998,7 | 11300,3 | 15541,9 | 9213,34 | 4977,02 | 9825,3225 |
| FA 27:1;O | 345,81 | 357,32 | 708,04 | 832,43 | 588,92 | 572,35 | 773,95 | 410,25 | 573,63375 |
| FA 28:0 | 29616 | 25950,3 | 53933,44 | 47723,77 | 26992,38 | 51363,3 | 23821 | 23047,54 | 35305,9675 |
| FA 29:0 | 7417,52 | 7559,02 | 14839,11 | 13057,37 | 8072,2 | 14479,9 | 6113,41 | 6714,01 | 9781,5725 |
| FA 30:0 | 52445,19 | 45153,9 | 82836,06 | 89403,34 | 55500,61 | 98932,1 | 31886,6 | 37315,67 | 61684,1963 |
| FA 31:0 | 6446,34 | 5840,61 | 13595,82 | 9625,83 | 6282,37 | 11848,6 | 4568,06 | 5273,86 | 7935,1825 |
| FA 32:0 | 21457,28 | 17622,6 | 38628,21 | 31247,39 | 18502,24 | 40189,9 | 12732 | 17174,42 | 24694,2488 |
| FA 33:0 | 2600,24 | 2669,96 | 5778,54 | 3860,46 | 2787,12 | 6157,75 | 1698,72 | 2321,43 | 3484,2775 |
| FA 34:0 | 5404,8 | 5480,75 | 12010,1 | 9222,46 | 5867,3 | 12391,2 | 3817,7 | 5340,98 | 7441,91375 |
| FA 34:8 | 2210,59 | 1664,69 | 2730,8 | 7497,76 | 2592,36 | 6736,4 | 2009,34 | 1485,63 | 3365,94625 |
| FA 42:5 | 1591750 | 980937 | 3218497,5 | 3160881 | 1991742 | 2892372 | 2223209 | 1592739 | 2206515,78 |
| FA 42:6 | 1284,84 | 1057,61 | 2519,41 | 3210,76 | 2163,7 | 2477,72 | 2685,04 | 1744,96 | 2143,005 |
| FA 44:5 | 485459,3 | 388695 | 956204,95 | 1242818 | 606923,1 | 1064677 | 669228 | 445158,6 | 732395,556 |
| FA 44:6 | 1033,56 | 754,76 | 1636,97 | 2755,35 | 1754,23 | 2134,16 | 1703,24 | 876,42 | 1581,08625 |
| **Dihexosylceramides** |  |  |  |  |  |  |  |  |  |
| Hex2Cer 32:1;2O|Hex2Cer 16:1;2O/16:0 | 171,44 | 102,63 | 131,88 | 267,23 | 320,71 | 294,59 | 306,9 | 192,18 | 223,445 |
| Hex2Cer 33:1;2O|Hex2Cer 17:1;2O/16:0 | 96,04 | 61,67 | 154,49 | 196,48 | 244,24 | 276,36 | 216,33 | 169,3 | 176,86375 |
| Hex2Cer 34:0;2O | 564,41 | 238,23 | 583,85 | 1089,98 | 1595,89 | 1455,76 | 960,86 | 801,11 | 911,26125 |
| Hex2Cer 34:1;2O|Hex2Cer 18:1;2O/16:0 | 4268,98 | 2142,18 | 4125,24 | 9326,87 | 14650,16 | 12079,1 | 7118,41 | 6278,8 | 7498,72125 |
| Hex2Cer 36:1;2O|Hex2Cer 18:1;2O/18:0 | 149,98 | 76,09 | 79,3 | 133,77 | 288,24 | 132,47 | 150,53 | 122,68 | 141,6325 |
| Hex2Cer 38:1;2O|Hex2Cer 16:1;2O/22:0 | 871,44 | 373,24 | 497,2 | 744,76 | 1075,12 | 953,13 | 1314,69 | 765,82 | 824,425 |
| Hex2Cer 39:0;2O|Hex2Cer 17:0;2O/22:0 | 542,67 | 280,11 | 554,93 | 750,29 | 1352,9 | 1193,18 | 1369,84 | 659,59 | 837,93875 |
| Hex2Cer 39:1;2O|Hex2Cer 16:1;2O/23:0 | 1407,66 | 563,26 | 579,35 | 1410,48 | 2266,1 | 1531,94 | 1617,54 | 962,07 | 1292,3 |
| Hex2Cer 40:1;2O|Hex2Cer 18:1;2O/22:0 | 1812,47 | 824,52 | 1472,94 | 2144,64 | 3387,34 | 3585,77 | 3681,84 | 2215,68 | 2390,65 |

| Hex2Cer 40:2;2O|Hex2Cer 16:1;2O/24:1 | 89,42 | 62,27 | 75,27 | 114,73 | 188,63 | 173,15 | 215,85 | 87,11 | 125,80375 |
| --- | --- | --- | --- | --- | --- | --- | --- | --- | --- |
| Hex2Cer 41:1;2O|Hex2Cer 18:1;2O/23:0 | 771,27 | 410,99 | 834,29 | 1661,05 | 1665,69 | 1148,02 | 1493,24 | 1504,16 | 1186,08875 |
| Hex2Cer 42:1;2O|Hex2Cer 18:1;2O/24:0 | 713,77 | 275,05 | 570,76 | 1023,45 | 1323,91 | 1172,05 | 1281,98 | 1243,47 | 950,555 |
| Hex2Cer 42:2;2O|Hex2Cer 18:1;2O/24:1 | 128,88 | 73,35 | 148,67 | 196,46 | 350,15 | 289,87 | 318,34 | 167,88 | 209,2 |
| HexCer 33:1;2O|HexCer 17:1;2O/16:0 | 567,51 | 302,54 | 448,41 | 760,08 | 1068,45 | 1028,41 | 325,78 | 484,5 | 623,21 |
| HexCer 34:0;2O|HexCer 18:0;2O/16:0 | 208,84 | 133,77 | 151,09 | 370,18 | 658,83 | 810,64 | 291,58 | 225,54 | 356,30875 |
| HexCer 34:1;2O | 691,9 | 502,67 | 577,93 | 872,66 | 2635,51 | 1734,35 | 1253,73 | 941,34 | 1151,26125 |
| HexCer 34:1;2O|HexCer 18:1;2O/16:0 | 2456,79 | 1321,26 | 2035,75 | 3522,74 | 5859,82 | 5993,14 | 3354,2 | 2459,54 | 3375,405 |
| HexCer 34:1;3O|HexCer 18:1;2O/16:0;O | 720,57 | 334,97 | 535,96 | 1065,9 | 1111,59 | 727,98 | 335,28 | 487,43 | 664,96 |
| HexCer 38:1;2O | 289,17 | 288,2 | 124,19 | 284,21 | 381,73 | 157,77 | 705,68 | 211,77 | 305,34 |
| HexCer 39:1;3O | 1912,11 | 1378,5 | 846,53 | 1871,09 | 1955,48 | 1537,92 | 1114,66 | 1008,28 | 1453,07125 |
| HexCer 40:0;3O | 812,9 | 368,42 | 262,63 | 803,49 | 1111,61 | 463,4 | 586,06 | 395,65 | 600,52 |
| HexCer 40:1;2O | 2877,01 | 1361,96 | 1665,19 | 3525,72 | 8865,3 | 4974,19 | 4549,28 | 2429,83 | 3781,06 |
| HexCer 40:1;2O|HexCer 18:1;2O/22:0 | 780,69 | 395,17 | 732,48 | 1042,32 | 2096,2 | 1652,01 | 673,29 | 1002,91 | 1046,88375 |
| HexCer 40:1;3O | 2616,89 | 1708,66 | 1326,93 | 3139,71 | 3008,58 | 2186,83 | 1562,45 | 2264,79 | 2226,855 |
| HexCer 41:1;2O | 1761,58 | 933,45 | 1337,06 | 1921,64 | 4916,11 | 2910,86 | 2355,95 | 1656,48 | 2224,14125 |
| HexCer 41:1;2O|HexCer 18:1;2O/23:0 | 3898,43 | 1822,28 | 5045,92 | 6400,77 | 8818,35 | 10037,6 | 2779,07 | 4173,09 | 5371,9425 |
| 9,12 | 6199,71 | 2716,81 | 2355,53 | 7488,23 | 9781,61 | 6961,6 | 2942,92 | 3329,34 | 5221,96875 |
| HexCer 42:1;2O | 5010,65 | 2276,79 | 2392,39 | 5339,51 | 10992,73 | 9599,69 | 4367,74 | 3606,52 | 5448,2525 |
| HexCer 42:1;3O | 5169,06 | 2361,31 | 2029,78 | 5655,97 | 6766,28 | 5520,17 | 1825,07 | 3259,84 | 4073,435 |
| HexCer 43:1;3O | 1452,33 | 739,92 | 523,11 | 1828,57 | 1486,35 | 1501,7 | 403,5 | 1069,88 | 1125,67 |
| HexCer 49:5;4O | 1751,69 | 1010,02 | 1233,7 | 2060,01 | 2584,44 | 2802,64 | 3980,27 | 2306,72 | 2216,18625 |
| HexCer 51:9;3O | 682,8 | 242,71 | 373,13 | 947,19 | 1362,09 | 963,28 | 1059,36 | 752,6 | 797,895 |
| HexCer 53:9;3O | 475,4 | 261,41 | 420,97 | 588,85 | 1060,54 | 1124,46 | 705,18 | 629,98 | 658,34875 |
| **Lysophophatidylcholine** |  |  |  |  |  |  |  |  |  |
| LPC 14:0 | 1386,72 | 533,01 | 1434,04 | 2388,53 | 2740,27 | 2381,86 | 2306,1 | 1011,51 | 1772,755 |
| LPC 15:0 | 1227,13 | 801,4 | 1267,28 | 1999,84 | 1770,65 | 2003,18 | 994,62 | 1300,9 | 1420,625 |
| LPC 16:0 | 8184,92 | 3888,63 | 6927,41 | 19879,31 | 23390,51 | 17924,6 | 19254,7 | 7362,04 | 13351,5188 |
| LPC 17:0 | 325,74 | 186,58 | 240,43 | 518,44 | 456,42 | 488,9 | 356,94 | 268,98 | 355,30375 |
| LPC 18:0 | 1621,91 | 385,21 | 1059,53 | 3172,89 | 1964,01 | 1719,19 | 2866,61 | 1475,66 | 1783,12625 |
| LPC 18:1 | 4113,44 | 1596,89 | 3223,18 | 6450,67 | 4740,53 | 4448,28 | 3496,2 | 3712,63 | 3972,7275 |
| LPC 18:2 | 1954,8 | 782,68 | 1280,7 | 1969,9 | 2265,68 | 2418,57 | 926,91 | 1335,12 | 1616,795 |
| LPC 26:0 | 535,26 | 414,91 | 444,28 | 622,01 | 712,14 | 738,24 | 381,49 | 496,49 | 543,1025 |
| LPC 28:7 | 381,26 | 248,93 | 250,2 | 1188,59 | 791,46 | 550,12 | 694,36 | 318,76 | 552,96 |
| **Lysophosphatidylethanolamine** |  |  |  |  |  |  |  |  |  |
| LPE 16:0 | 1600,17 | 887,36 | 1244,92 | 2053,14 | 3116,45 | 2446,5 | 2749,14 | 1362,44 | 1932,515 |
| LPE 16:1 | 158,14 | 151,14 | 176,61 | 311,49 | 521,18 | 379,58 | 183,11 | 123,75 | 250,625 |
| LPE 18:0 | 1491,18 | 967,58 | 1079,52 | 1829,39 | 1169,4 | 1331,56 | 5121,53 | 1503,24 | 1811,675 |
| LPE 18:1 | 9536,77 | 4878,15 | 7086,87 | 9337,87 | 7865,03 | 7610,69 | 7545,47 | 8332,53 | 7774,1725 |
| LPE 18:2 | 2584,16 | 1671,1 | 1463,47 | 1798,44 | 2463,88 | 2025,51 | 1894,4 | 1575,8 | 1934,595 |
| LPE 18:3 | 1314,71 | 267,01 | 1092,23 | 1863,26 | 1316,74 | 2057,98 | 524,05 | 1237,29 | 1209,15875 |
| LPE 20:4 | 325,23 | 255,79 | 210,53 | 223,96 | 222 | 145,71 | 160,29 | 305,6 | 231,13875 |
| **Lysophosphatidylinositol** |  |  |  |  |  |  |  |  |  |
| LPI 18:0 | 842,14 | 834,7 | 1049,25 | 1099,02 | 829,86 | 1580,88 | 1778,29 | 702,97 | 1089,63875 |
| LPI 18:1 | 642,47 | 736,78 | 779,63 | 1864,69 | 1825,67 | 1950,44 | 1033,43 | 755,49 | 1198,575 |

| LPI 18:2 | 175,76 | 187,81 | 200,48 | 347,28 | 280,78 | 562,3 | 102,99 | 190,28 | 255,96 |
| --- | --- | --- | --- | --- | --- | --- | --- | --- | --- |
| **Monoacylglyceros** |  |  |  |  |  |  |  |  |  |
| MG 15:0 | 573,4 | 203,07 | 3072,03 | 506,59 | 826,68 | 2098,42 | 206,95 | 994,34 | 1060,185 |
| MG 17:3 | 1774,16 | 682,12 | 2107,05 | 2685,83 | 2222,16 | 3362,26 | 648,85 | 1938,88 | 1927,66375 |
| MG 17:4 | 2715,91 | 588,11 | 4213,55 | 5767,41 | 3143,57 | 4423,29 | 407,24 | 2795,45 | 3006,81625 |
| MG 18:0 | 3181,7 | 944,58 | 3458,98 | 4242,84 | 3154,5 | 6275,41 | 1092,05 | 2342,73 | 3086,59875 |
| MG 19:3 | 683,38 | 289,37 | 1112,28 | 809,41 | 797,81 | 1274,92 | 297,79 | 732,98 | 749,7425 |
| MG 21:4 | 2453,42 | 827,89 | 1936,02 | 4458,66 | 2456,89 | 5489,73 | 906,14 | 2226,41 | 2594,395 |
| MG 22:1 | 939,61 | 233,94 | 1169,21 | 1494,59 | 872,77 | 1264,12 | 163,18 | 716,8 | 856,7775 |
| MG 24:2 | 580,15 | 266,74 | 506,08 | 666,46 | 399,46 | 590,61 | 131,29 | 376,5 | 439,66125 |
| MG 30:7 | 7374,05 | 3516,09 | 6628,27 | 11888,07 | 10735,44 | 20587 | 2752,76 | 5518,65 | 8625,03625 |
| MG 32:8 | 1545,07 | 434,1 | 1201,46 | 2426,35 | 1952,78 | 2942,15 | 439,43 | 950,06 | 1486,425 |
| MG 34:8 | 3565,15 | 764,14 | 3027,72 | 4739,16 | 2941,3 | 4766,14 | 653,72 | 2175,06 | 2829,04875 |
| MG 36:6 | 800,4 | 337,4 | 646,12 | 1819,83 | 1374,28 | 1923,02 | 530,11 | 657,35 | 1011,06375 |
| **N-acyl ethanolamines** |  |  |  |  |  |  |  |  |  |
| NAE 12:0 | 6121,62 | 2884,9 | 4947,48 | 7824,93 | 6974,1 | 8526,89 | 1883,21 | 6404,26 | 5695,92375 |
| NAE 13:1 | 12247,15 | 3884,72 | 11197,15 | 14725,26 | 12398,1 | 16429,1 | 3734,84 | 10373,62 | 10623,7475 |
| NAE 14:1 | 16637,69 | 6031,91 | 19126,74 | 21662,6 | 14593,75 | 24985,6 | 5303,74 | 15491,63 | 15479,2113 |
| NAE 15:1 | 2479,94 | 1095,77 | 2308,46 | 3017,92 | 2422,24 | 4136,19 | 873,69 | 2501,38 | 2354,44875 |
| NAE 16:1 | 610365,1 | 321393 | 774158,8 | 918517,5 | 661123,05 | 1053518 | 196970 | 609417,4 | 643182,825 |
| NAE 16:2 | 1342677 | 672369 | 1763923 | 1962940 | 1432296,5 | 2293137 | 362505 | 1408999,5 | 1404855,83 |
| NAE 16:3 | 832897 | 408199 | 838760,95 | 1168787 | 1065807 | 1221895 | 253867 | 818057,15 | 826033,675 |
| NAE 16:4 | 47168,62 | 21884,4 | 54311,49 | 61739,65 | 56234,76 | 71575,8 | 10284,6 | 49902,37 | 46637,705 |
| NAE 17:4 | 9650,06 | 4259,58 | 9646,4 | 12120,8 | 9605,92 | 14499,2 | 4021,32 | 7684,23 | 8935,935 |
| NAE 18:1 | 23369,7 | 15103,5 | 33520,58 | 37669,58 | 26116,3 | 43816,3 | 7431,92 | 24183,43 | 26401,4138 |
| NAE 18:2 | 36024,92 | 23250,6 | 51304,32 | 56035,97 | 38802,84 | 65295,7 | 10032 | 38635,64 | 39922,7463 |
| NAE 18:3 | 5122,5 | 2951,64 | 4979,18 | 7332,12 | 6803,34 | 7917,48 | 1924,17 | 4796,32 | 5228,34375 |
| NAE 18:4 | 143012,2 | 57257,2 | 155527,95 | 195437,85 | 159708,95 | 230759 | 44975,1 | 132870,15 | 139943,49 |
| NAE 18:5 | 212232 | 79628,6 | 228042,25 | 289599,85 | 229682,75 | 316471 | 66854,8 | 201580,7 | 203011,498 |
| NAE 20:1 | 3051,65 | 1414,32 | 7419,8 | 2629,34 | 2635,83 | 7683,75 | 1023,84 | 3537,35 | 3674,485 |
| NAE 20:2 | 6811,3 | 4039,06 | 24150,96 | 9717,75 | 8896,39 | 20648,7 | 2226,06 | 7128,51 | 10452,34 |
| NAE 20:4 | 6650,39 | 4029,01 | 8633 | 9261,5 | 6505,3 | 10643,6 | 2003,7 | 5714,58 | 6680,13125 |
| NAE 20:5 | 5275,56 | 3073,11 | 7023,4 | 7611,96 | 6116,37 | 8607,63 | 1564,75 | 5329,3 | 5575,26 |
| NAE 22:3 | 3458,2 | 2414,45 | 2643,24 | 4399,84 | 4577,63 | 4286,8 | 2837,23 | 3448,4 | 3508,22375 |
| NAE 22:4 | 1566,58 | 803,22 | 5619,1 | 1988,19 | 2219,08 | 4176,36 | 560,33 | 1469,15 | 2300,25125 |
| NAE 22:5 | 1638,62 | 1354,16 | 4337,36 | 2348,87 | 1987,15 | 3866,65 | 483,17 | 1628,31 | 2205,53625 |
| NAE 24:4 | 2070,89 | 727,84 | 2074,53 | 2560,65 | 2258,24 | 3034,21 | 777,38 | 1670,24 | 1896,7475 |
| NAE 5:0 | 5246,3 | 1402,08 | 3209,09 | 13946 | 8644,71 | 9129,72 | 1652,03 | 6525,48 | 6219,42625 |
| NAE 6:0 | 34618,5 | 7530,25 | 31228,06 | 97524,7 | 77925,83 | 71233,8 | 8361,28 | 38117,8 | 45817,5213 |
| NAE 7:0 | 1874,67 | 430,22 | 1161,01 | 2951,2 | 2852,89 | 2126,71 | 501,38 | 1677,81 | 1696,98625 |
| **Phosphatidylcholines** |  |  |  |  |  |  |  |  |  |
| PC 24:0 | 174,12 | 111,95 | 167,74 | 452,21 | 588,8 | 839,17 | 1869,2 | 425,26 | 578,55625 |
| PC 26:0|PC 12:0_14:0 | 792,46 | 490,22 | 662,73 | 1949,52 | 3121,48 | 3390,46 | 8564,57 | 1076,09 | 2505,94125 |
| PC 27:0|PC 12:0_15:0 | 265,05 | 120,03 | 240,54 | 400,5 | 586,16 | 716,83 | 981,07 | 283,31 | 449,18625 |
| PC 28:0 | 561,72 | 318,23 | 429,08 | 682,96 | 812,57 | 997,62 | 1884,14 | 624,49 | 788,85125 |
| PC 28:0|PC 14:0_14:0 | 24673,59 | 15163 | 14681,55 | 29851,01 | 49345,11 | 51237,1 | 131170 | 29288,47 | 43176,1825 |

| PC 28:1 | 330,05 | 175,89 | 202,27 | 510,36 | 786,14 | 707,83 | 943,68 | 344,3 | 500,065 |
| --- | --- | --- | --- | --- | --- | --- | --- | --- | --- |
| PC 29:0|PC 14:0_15:0 | 6503,99 | 3786,49 | 3182,99 | 6197,64 | 9524,32 | 9094,63 | 16293,8 | 6669,24 | 7656,63625 |
| PC 30:0|PC 14:0_16:0 | 491873,4 | 306072 | 279618,5 | 570512,05 | 692300,95 | 748175 | 672936 | 445298,15 | 525848,288 |
| PC 30:1|PC 14:0_16:1 | 4452,43 | 2826,31 | 2582,86 | 6240,35 | 14718,18 | 13490,5 | 13669,9 | 4386,28 | 7795,84 |
| PC 30:3 | 511,75 | 290,56 | 281,9 | 547,67 | 477,68 | 589,59 | 452,91 | 499,33 | 456,42375 |
| PC 31:0|PC 15:0_16:0 | 23339,35 | 13885,3 | 16258 | 26582,43 | 27173,61 | 23071,7 | 34715,4 | 25993,16 | 23877,3675 |
| PC 31:1 | 1880,6 | 971,06 | 1100,55 | 1991,27 | 2380,28 | 2515,34 | 1674,98 | 2024,2 | 1817,285 |
| PC 31:1|PC 15:0_16:1 | 1430,24 | 951,19 | 1140,62 | 1679,34 | 3674,45 | 3947,23 | 1943,96 | 1424,17 | 2023,9 |
| PC 32:0 | 9169,07 | 4857,13 | 4180,2 | 9727,24 | 12606,76 | 10996,9 | 6750,27 | 8947,18 | 8404,3425 |
| PC 32:0|PC 16:0_16:0 | 477631,5 | 254324 | 264871,1 | 653245,85 | 650692,5 | 685933 | 426096 | 411783,75 | 478072,294 |
| PC 32:1 | 1605,56 | 845,75 | 822,07 | 1535,14 | 2512,87 | 2440,25 | 1636,31 | 1681,78 | 1634,96625 |
| PC 32:1|PC 14:0_18:1 | 140372,3 | 86847,5 | 69088,29 | 141821,75 | 253899,35 | 231537 | 165641 | 129922,55 | 152391,218 |
| PC 32:2|PC 16:1_16:1 | 7883,94 | 4760,2 | 3548,46 | 6270,32 | 11918,47 | 10215,8 | 16720,8 | 5667,09 | 8373,125 |
| PC 32:3|PC 14:0_18:3 | 838,3 | 465,03 | 280,38 | 853,13 | 1238,66 | 1107,22 | 1449,88 | 587,66 | 852,5325 |
| PC 32:3|PC 14:1_18:2 | 8118,3 | 6900,4 | 4033,97 | 7525,76 | 12542,48 | 10639,4 | 15383,7 | 7894,71 | 9129,83125 |
| PC 33:0|PC 16:0_17:0 | 11059,14 | 5454,53 | 7239,62 | 11160,25 | 11606,13 | 10964,7 | 6189,77 | 10354,22 | 9253,545 |
| PC 33:1|PC 15:0_18:1 | 22774,52 | 13116,1 | 15468,89 | 23695,52 | 25599,05 | 26192,7 | 26438,9 | 25545,36 | 22353,8813 |
| PC 33:2 | 2570,82 | 1296,03 | 1221,42 | 2886,84 | 3093,65 | 2947,4 | 1653,82 | 1987,98 | 2207,245 |
| PC 34:0|PC 16:0_18:0 | 66247,25 | 33974,5 | 41079,5 | 94946,32 | 45042,61 | 43190,8 | 83869 | 84893,33 | 61655,415 |
| PC 34:1 | 21414,18 | 8068,49 | 12585,34 | 22408,43 | 21819,99 | 23381,9 | 10606,6 | 21058,51 | 17667,935 |
| PC 34:1|PC 16:0_18:1 | 952381,1 | 434909 | 678126,95 | 1133224 | 1105009 | 1305739 | 653886 | 906160,1 | 896179,294 |
| PC 34:2 | 1956,65 | 1178,2 | 1366,73 | 1546,76 | 2320,91 | 1995,03 | 1797,38 | 1581,81 | 1717,93375 |
| PC 34:2|PC 15:1_19:1 | 1263,49 | 685,27 | 1034,87 | 1607,32 | 1580,82 | 1832,13 | 727,07 | 1358,66 | 1261,20375 |
| PC 34:2|PC 16:0_18:2 | 143564,5 | 91277,2 | 76683,15 | 113410,75 | 170308,4 | 150559 | 170453 | 114675,9 | 128866,384 |
| PC 34:3|PC 16:0_18:3 | 31943,5 | 22477,2 | 12331,97 | 30703,2 | 53541,44 | 53065,7 | 41719,7 | 16290,3 | 32759,1238 |
| PC 34:4|PC 17:2_17:2 | 355,72 | 240,16 | 233,1 | 545 | 892,08 | 831,58 | 431,35 | 284,33 | 476,665 |
| PC 35:0|PC 17:0_18:0 | 2003,51 | 726,75 | 1443,93 | 2105,84 | 1549,02 | 1538,24 | 1193,69 | 2168,24 | 1591,1525 |
| PC 35:1|PC 17:0_18:1 | 7485,29 | 4508,77 | 6800,86 | 8379,99 | 6930,97 | 8658,01 | 7865,01 | 11464,51 | 7761,67625 |
| PC 35:2|PC 17:1_18:1 | 6697,9 | 3841,44 | 3832,68 | 6914,56 | 6208,29 | 5941,19 | 6323,06 | 6418,51 | 5772,20375 |
| PC 35:3 | 1391,77 | 677,53 | 770,39 | 1376,25 | 1152,53 | 1539,76 | 960,25 | 912,99 | 1097,68375 |
| PC 36:0|PC 18:0_18:0 | 1900,96 | 925,71 | 1605,52 | 2518,07 | 1272,53 | 1712,39 | 2871,16 | 2676,69 | 1935,37875 |
| PC 36:1|PC 18:0_18:1 | 148984,2 | 78756,8 | 115512,5 | 202055,75 | 137874,1 | 132038 | 184964 | 216655,35 | 152105,05 |
| PC 36:2 | 1954,59 | 816,49 | 1234,99 | 2081,59 | 1006,97 | 544,25 | 841,75 | 2243,42 | 1340,50625 |
| PC 36:2|PC 18:1_18:1 | 1245,59 | 776,32 | 807 | 1507,48 | 1008,66 | 684,27 | 1011,44 | 1442,12 | 1060,36 |
| PC 36:2|PC 18:1_18:1 | 127104,2 | 67924,1 | 84322,5 | 137582 | 92267,26 | 84363,9 | 63271,9 | 175626,05 | 104057,726 |
| PC 36:3 | 1655,84 | 1085,13 | 954,48 | 1425,07 | 1407,7 | 1326,26 | 1560,04 | 1708,29 | 1390,35125 |
| PC 36:3|PC 18:1_18:2 | 85554,47 | 56580,4 | 42680 | 78059,25 | 63688,82 | 74836,2 | 85574,6 | 72790,52 | 69970,5325 |
| PC 36:4 | 824,5 | 455,29 | 391,37 | 645,99 | 903,48 | 750,61 | 584,1 | 651,84 | 650,8975 |
| PC 36:4|PC 18:2_18:2 | 33758,73 | 18175 | 14091,62 | 35587,93 | 33234,56 | 32999,5 | 24235,3 | 22889,8 | 26871,5538 |
| PC 36:5|PC 16:0_20:5 | 4404,76 | 2826,79 | 2341,36 | 5767,11 | 6345,41 | 5621 | 5089,33 | 2976,78 | 4421,5675 |
| PC 36:5|PC 18:2_18:3 | 2044,16 | 1125,61 | 944,1 | 1761,28 | 1959,23 | 2422,54 | 1812,9 | 1190,16 | 1657,4975 |
| PC 36:6 | 149,26 | 75,19 | 66,11 | 158,21 | 185,49 | 181,41 | 120,74 | 77,83 | 126,78 |
| PC 37:1|PC 19:0_18:1 | 379,43 | 270,86 | 559,86 | 750,48 | 443,24 | 604,28 | 529,07 | 736,78 | 534,25 |
| PC 37:2|PC 18:1_19:1 | 1038,62 | 568,9 | 1013,5 | 1222,19 | 920,81 | 1274,14 | 864,12 | 1125,42 | 1003,4625 |
| PC 38:1|PC 20:0_18:1 | 1053,46 | 520,93 | 993,07 | 1537,67 | 1029,56 | 1283,17 | 546,9 | 1501,98 | 1058,3425 |
| PC 38:2 | 851,52 | 409,13 | 758,86 | 722,89 | 849,72 | 977,87 | 628,07 | 999,99 | 774,75625 |

| PC 38:2|PC 20:0_18:2 | 2736,13 | 1453,06 | 2319,41 | 2832,67 | 1989,04 | 2389,72 | 1852,41 | 2934,42 | 2313,3575 |
| --- | --- | --- | --- | --- | --- | --- | --- | --- | --- |
| PC 38:3 | 1733,95 | 894,02 | 1603,59 | 1593,56 | 1718,16 | 1196,06 | 2968,7 | 2088,2 | 1724,53 |
| PC 38:3|PC 20:0_18:3 | 1163,39 | 606,6 | 906,73 | 1674,62 | 1059,01 | 1255,7 | 900,38 | 1065,17 | 1078,95 |
| PC 38:4 | 1577,94 | 784,69 | 1634,77 | 1625,53 | 1427,76 | 744,48 | 1553,79 | 2528,32 | 1484,66 |
| PC 38:4|PC 18:1_20:3 | 2836,27 | 1520,27 | 1509,71 | 4008,17 | 3271,04 | 2907,35 | 3096,65 | 3703,51 | 2856,62125 |
| PC 38:5|PC 16:0_22:5 | 5733,96 | 4109,83 | 4398,34 | 7816,01 | 7120,35 | 6814,99 | 9101,61 | 9773,12 | 6858,52625 |
| PC 38:6|PC 19:3_19:3 | 2450,9 | 1403,34 | 1397,15 | 3556,91 | 2371,79 | 2403,45 | 1840,81 | 1998,39 | 2177,8425 |
| PC 38:7 | 383,47 | 238,16 | 192,01 | 446,39 | 456,79 | 348,95 | 301,76 | 377,12 | 343,08125 |
| PC 40:3|PC 25:1_15:2 | 365,33 | 197,8 | 318,58 | 521,61 | 369,64 | 266,99 | 269,61 | 585,27 | 361,85375 |
| PC 40:5|PC 18:0_22:5 | 1055,91 | 503,94 | 965,33 | 1424,77 | 855,48 | 935,47 | 910,95 | 1564,23 | 1027,01 |
| PC 40:6 | 1419,26 | 744,01 | 1179,1 | 1838,98 | 1703,83 | 1985,36 | 1633,32 | 2500,31 | 1625,52125 |
| PC O-29:1 | 590,09 | 432,18 | 422,02 | 567,78 | 686,45 | 705,05 | 547,44 | 556,97 | 563,4975 |
| PC O-31:0 | 5936,81 | 3156,03 | 4484,47 | 8112,92 | 7559,3 | 8591,15 | 5294,89 | 6558,8 | 6211,79625 |
| PC O-31:1 | 2034,68 | 1128,04 | 1381,39 | 1965,69 | 1896,85 | 2016,86 | 1814,61 | 2030,13 | 1783,53125 |
| PC O-31:6 | 83,05 | 39,01 | 45,93 | 114,67 | 247,72 | 259,53 | 144,74 | 89,05 | 127,9625 |
| PC O-33:2 | 3119,73 | 1615,08 | 1931,27 | 2267,69 | 3293,36 | 3054,77 | 1602,68 | 3206,96 | 2511,4425 |
| PC O-33:3 | 1823,41 | 908,82 | 1056,31 | 952,26 | 1382,99 | 1488,14 | 975,5 | 1373,56 | 1245,12375 |
| PC O-33:4 | 4830,69 | 4170,37 | 3390,19 | 5377,03 | 8952,82 | 6878,85 | 6190,42 | 6132,97 | 5740,4175 |
[truncated: 100,446 more chars]
