# Supplementary figures and images for: The effects of Thymus capitatus essential oil topical application on milk quality: a systems biology approach
Source: Sci Rep. 2025 Feb 7;15:4627. doi: 10.1038/s41598-025-88168-0 (PMC11805959; doi:10.1038/s41598-025-88168-0)

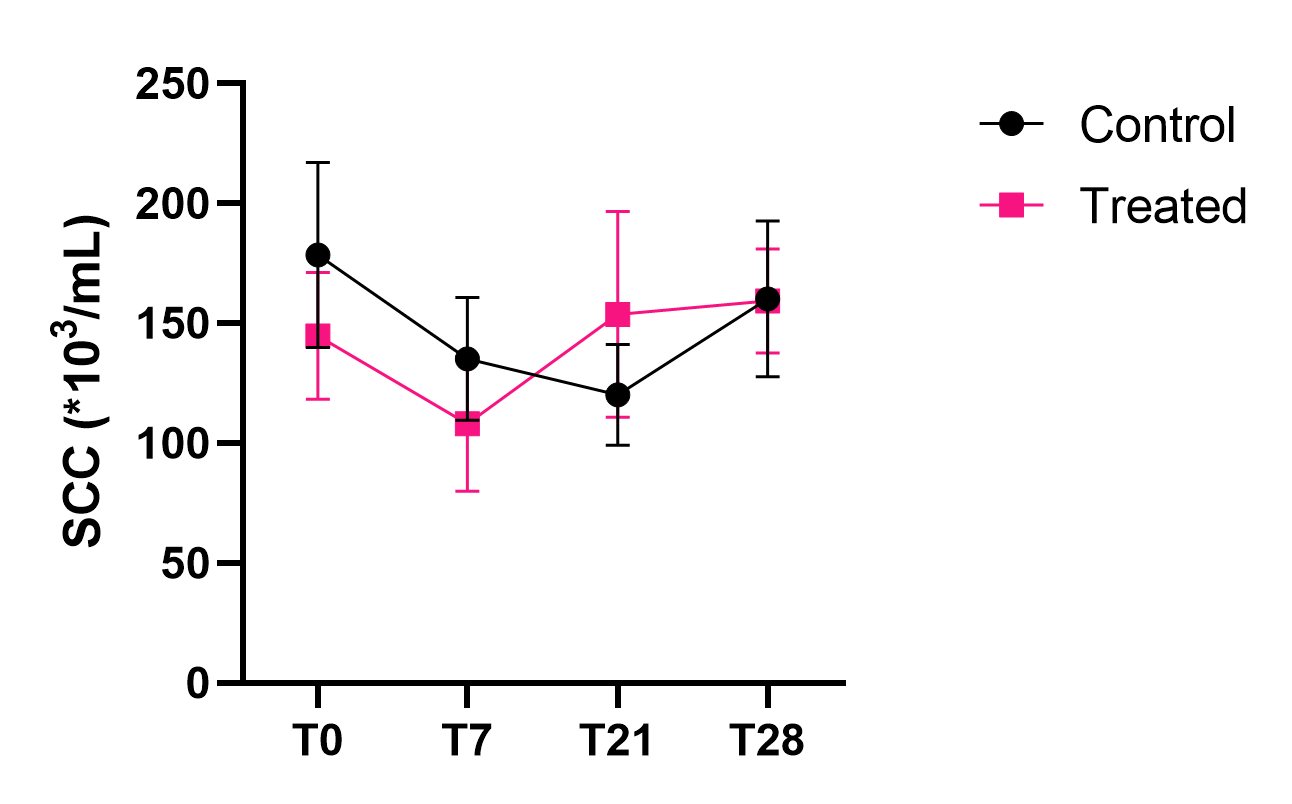

Supplement: Supplementary file 8 — Supplementary Material 8 [file 41598_2025_88168_MOESM8_ESM.tif]
